# Supplementary material for: HEPATOKIN1 is a biochemistry-based model of liver metabolism for applications in medicine and pharmacology
Source: Nat Commun. 2018 Jun 19;9:2386. doi: 10.1038/s41467-018-04720-9 (PMC6008457; doi:10.1038/s41467-018-04720-9)
Supplement: Supplementary file 1 — Supplementary Information [file 41467_2018_4720_MOESM1_ESM.pdf]

Supplementary Information to

A Biochemistry-Based Model of Liver Metabolism for Applications in Medicine and Pharmacology

N. Berndt et al.

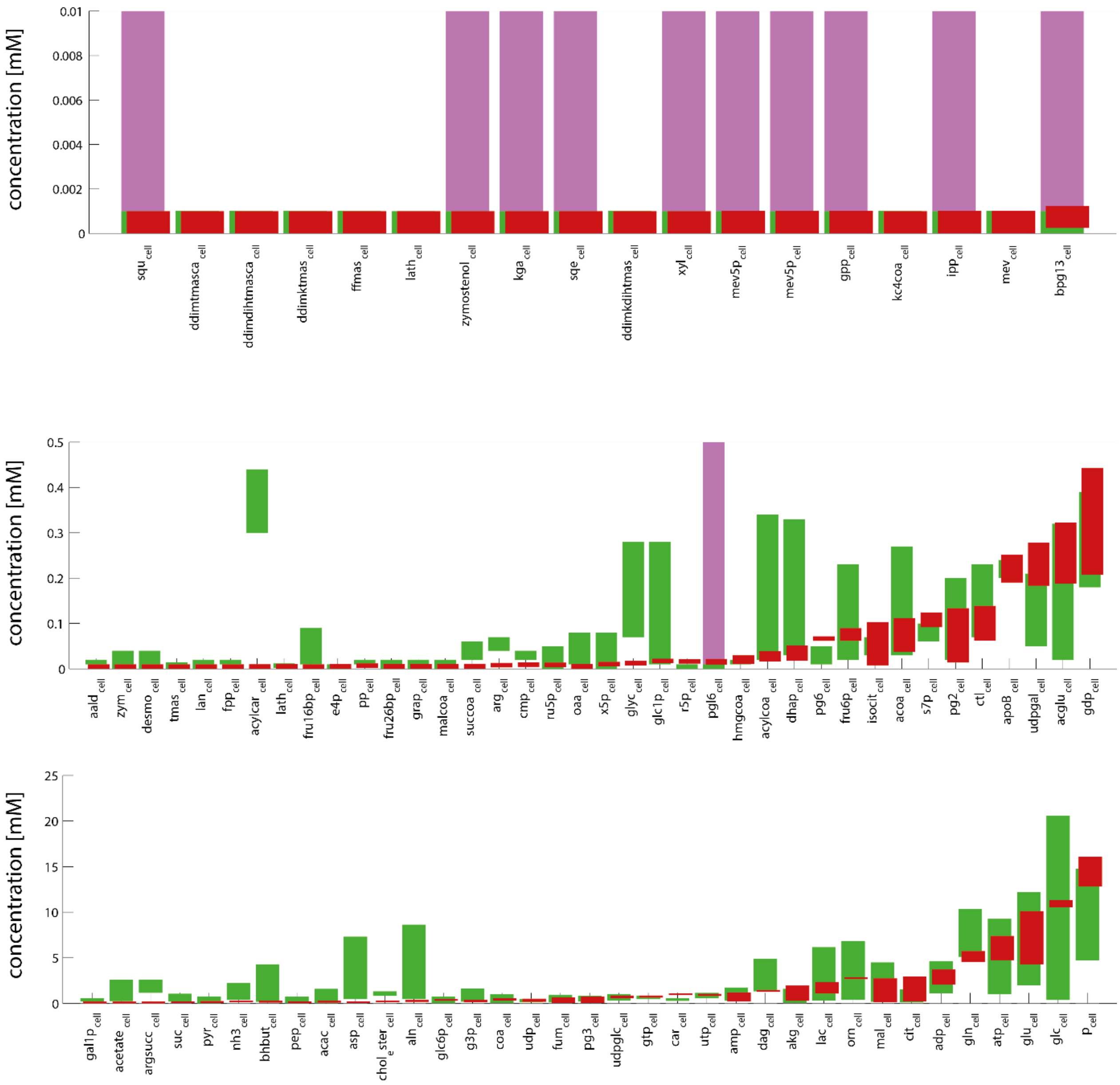

Supplementary Figure 1 *Comparison of computed and measured cellular metabolite concentrations*

Experimentally measured concentration ranges are indicated by green bars; simulated concentration ranges derived from the diurnal (24h) profile are depicted in red bars. Magenta bars indicate missing experimental values. Cellular concentrations are given as mM with respect to the cytosolic compartment. For the factors used for the conversion of experimental data from various sources and the numerical values of the computed and measured metabolite concentrations see Supplementary Data 1.

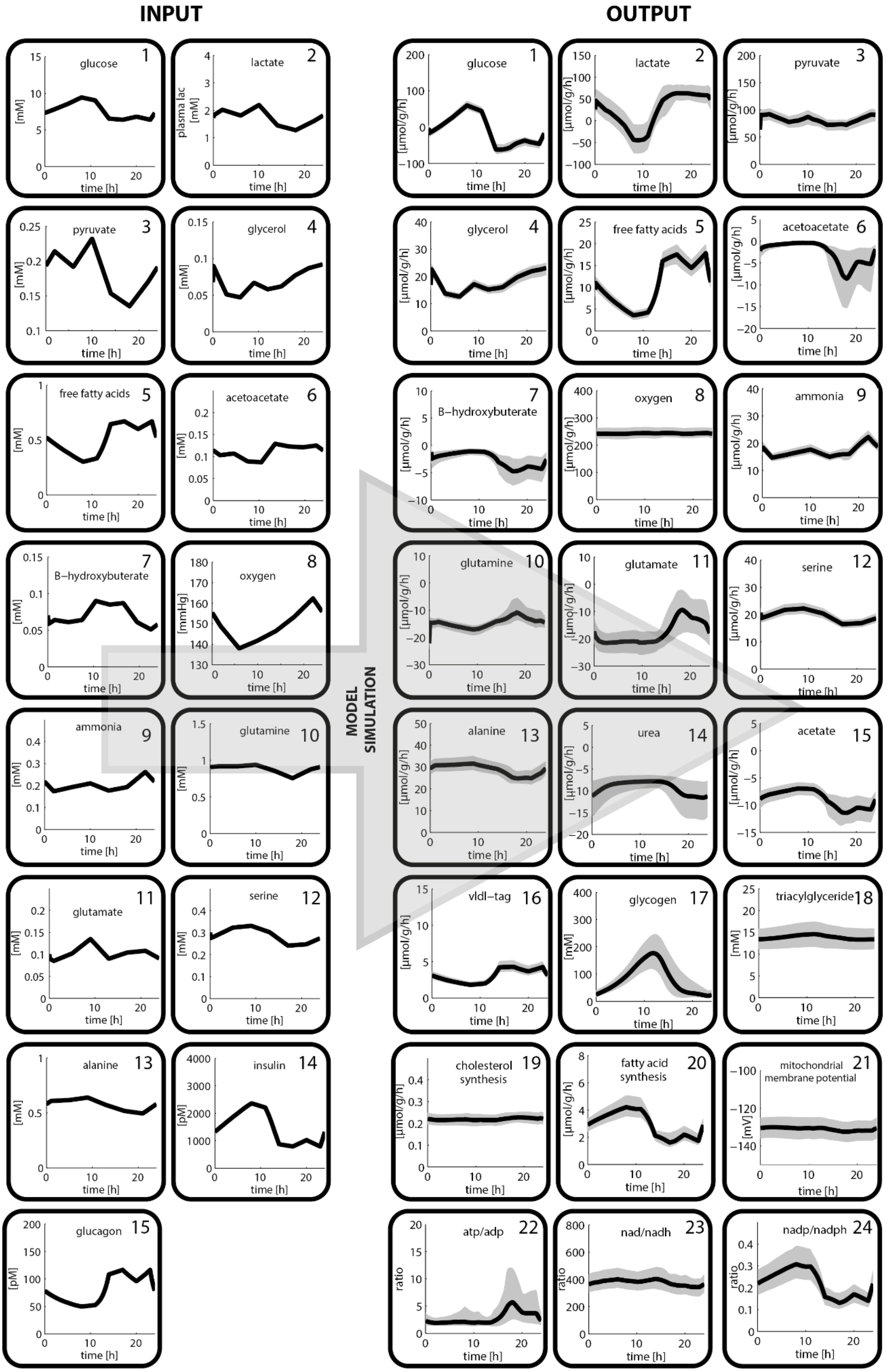

Supplementary Figure 2 *Simulated diurnal changes in the metabolic state of the liver*
Left panels: 24h plasma profile of hormones and metabolites of an ad libitum fed rat used as model input: 1) glucose, 2) lactate, 3) pyruvate, 4) glycerol, 5) free fatty acids, 6)

acetoacetate, 7) β-hydroxy butyrate, 8) oxygen, 9) ammonia, 10) glutamine, 11) glutamate, 12) serine, 13) alanine, 14) insulin, 15) glucagon.

Right panels: Simulated diurnal profile of 24 exchange fluxes and selected internal metabolites.
1) glucose exchange rate, 2) lactate exchange rate, 3) pyruvate exchange rate, 4) glycerol exchange rate, 5) fatty acid uptake rate, 6) acetoacetate secretion rate, 7) β-hydroxybuterate secretion rate, 8) oxygen uptake rate, 9) ammonia uptake rate, 10) glutamine exchange rate, 11) glutamate exchange rate, 12) serine exchange rate, 13) alanine exchange rate 14) urea secretion rate; 15) acetate exchange rate, 16) VLDL secretion rate, 17) glycogen storage, 18) cellular triglyceride concentration, 19) cholesterol synthesis rate, 20) fatty acid synthesis rate, 21) mitochondrial membrane potential, 22) ATP/ADP ratio, 23) NAD/NADH ratio (cytosolic), 24) NADP/NADPH ratio (cytosolic)

Grey areas encompass standard deviations of values obtained in 100 recurrent simulations where the Vmax value of each enzyme was randomly sampled from an interval between 90% and 110% of the reference value.

The complete table of infinitesimal and finite response is found in the Supplementary Data 4.

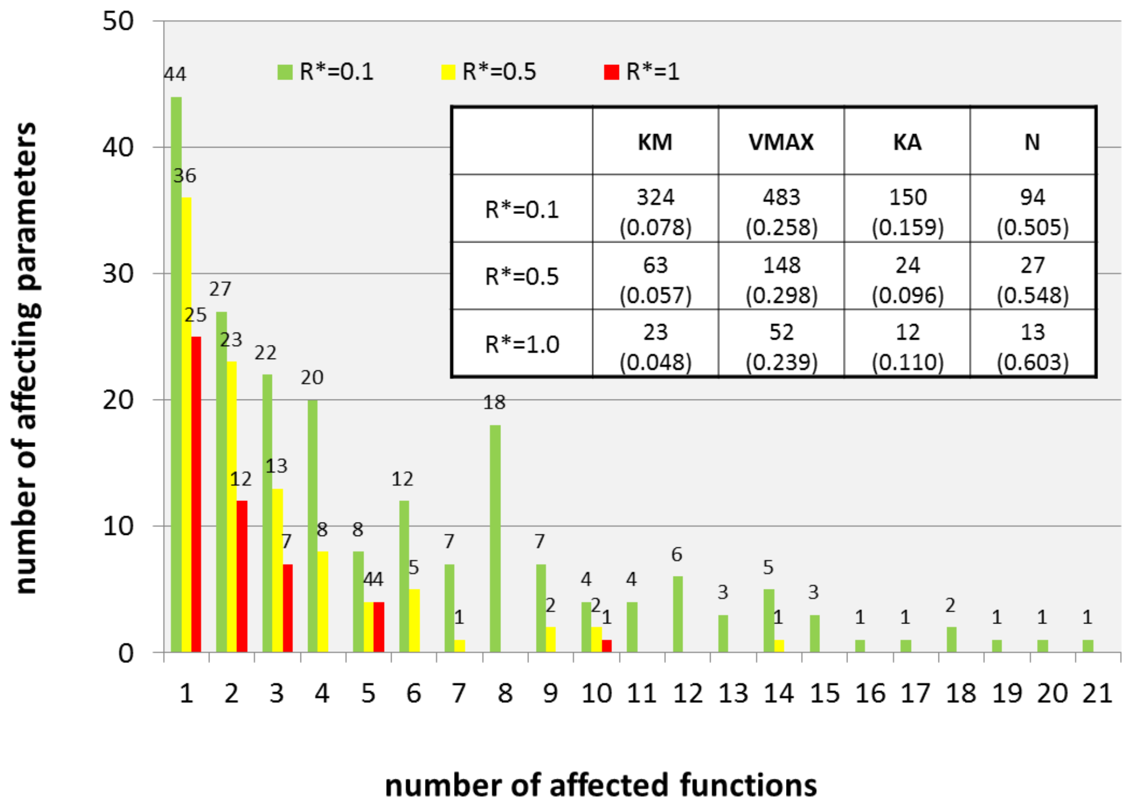

	KM	VMAX	KA	N
R*=0.1	324 (0.078)	483 (0.258)	150 (0.159)	94 (0.505)
R*=0.5	63 (0.057)	148 (0.298)	24 (0.096)	27 (0.548)
R*=1.0	23 (0.048)	52 (0.239)	12 (0.110)	13 (0.603)

.

Supplementary Figure 3 *Response to a finite parameter reduction by 50% (p→p/2)*
Number of parameters (y axis) affecting a given number of metabolic functions (x axis) with R >= R*.
The inserted Table shows the absolute and relative frequencies (in brackets) of parameter categories. The relative frequency is given by the absolute frequency divided by the total frequency of a parameter in a category (see Table 1, main text)

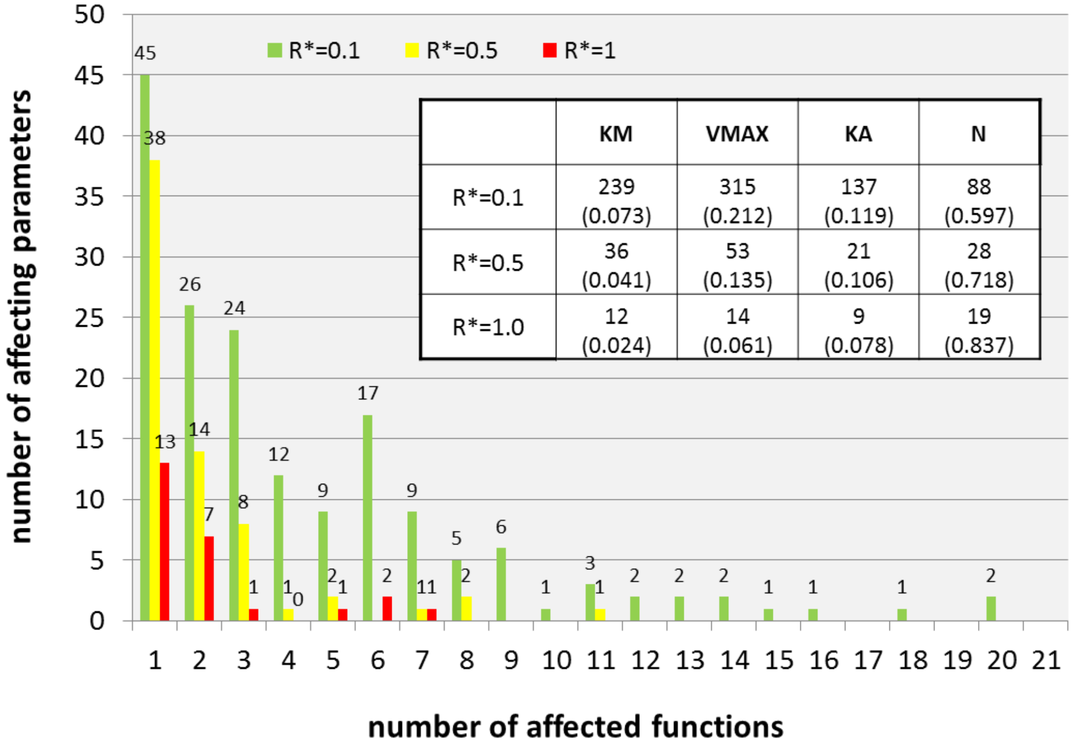

	KM	VMAX	KA	N
R*=0.1	239 (0.073)	315 (0.212)	137 (0.119)	88 (0.597)
R*=0.5	36 (0.041)	53 (0.135)	21 (0.106)	28 (0.718)
R*=1.0	12 (0.024)	14 (0.061)	9 (0.078)	19 (0.837)

Supplementary Figure 4 *Response to a finite parameter increase of 50% (p→1.5p)*
Number of parameters (y axis) affecting a given number of metabolic functions (x axis) with R >= R*.
The inserted table shows the absolute and relative frequencies (in brackets) of parameter categories. The relative frequency is given by the absolute frequency divided by the total

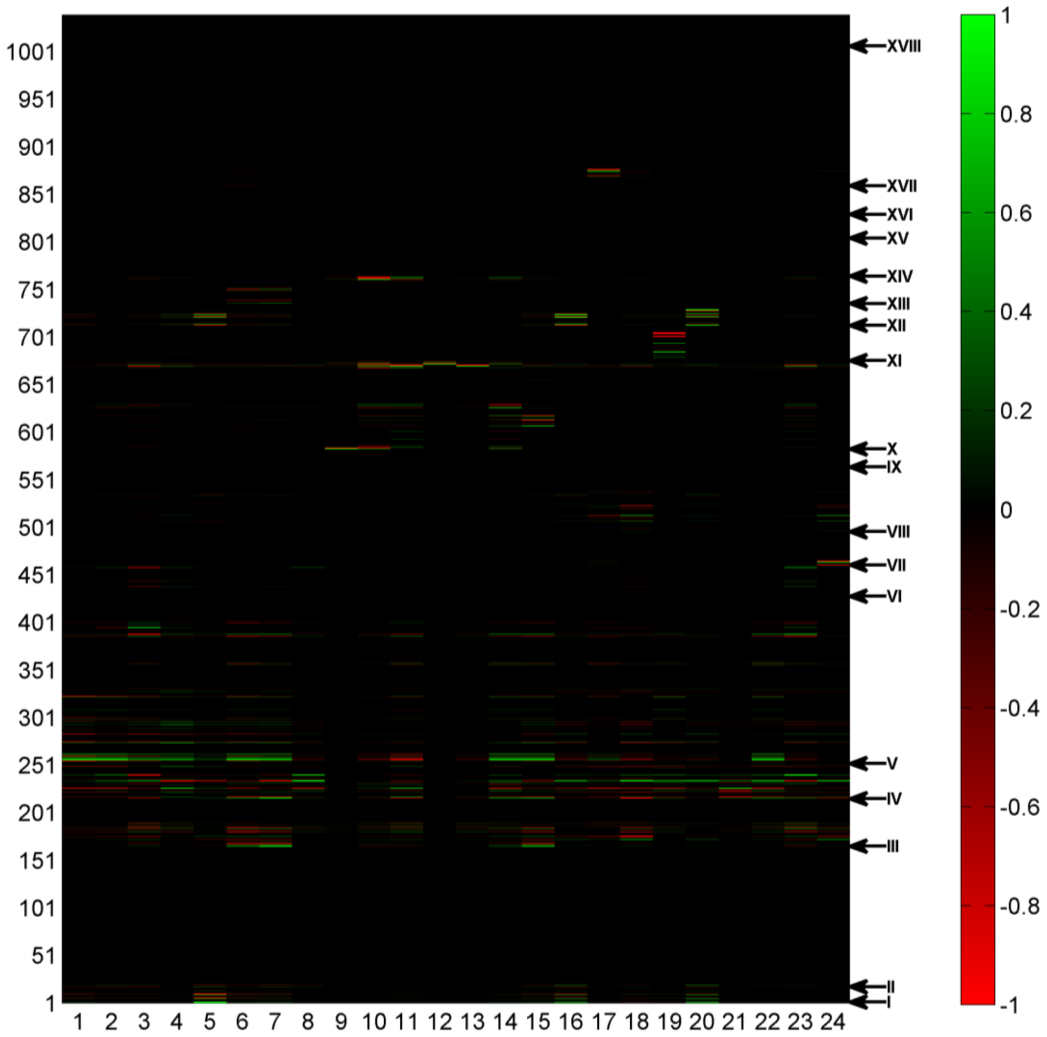

Supplementary Figure 5 *Heat map representation of infinitesimal response coefficients*

The infinitesimal response coefficients were calculated for a stationary metabolic state where the concentration of extracellular metabolites and hormones are set to their 24h mean. Numbers on the left margin refer to the number of the parameter given in the Supplementary Data 4. The numbers on the bottom margin indicate the metabolic function (see legend Fig.5, main text). The association of parameters with metabolic pathways is indicated by the roman numerals on the margin:

I: fatty acid uptake; II: β-oxidation; III: citric acid cycle; IV: oxidative phosphorylation; V: glycolysis/glyconeogenesis; VI: Malate-Aspartate Shuttle; VII: PPP; VIII: FAS; IX: TAG metabolism; X: urea cycle; XI: glycogen metabolism; XII: LD metabolism; XIII: ketone body metabolism; XIV: galactose metabolism; XV ethanol metabolism; XVI: fructose metabolism; XVII: cholesterol metabolism; XVIII: valproate metabolism.

Supplementary Table 1 Mostly affected metabolic functions at 50% parameter

#	Short name	Long name	EC/TCDB number	Estimated vmax value µmol/g/h
	Fatty acid uptake			
1	$v_{c16_{cyt}-uptake}^{carrier}$	Carrier mediated FATP	TCDB 4.C.1.1	1.56E+06
2	$v_{c16_{cyt}-uptake}^{diffusion}$	Diffusive uptake		2.33E+05
3	v_{ACSL1}	(Long-chain) acyl-coa synthetase 1	EC 6.2.1.3	9.92E+03
4	v_{ACSL4}	(Long-chain) acyl-coa synthetase 4	EC 6.2.1.3	1.03E+04
5	v_{ACSL5}	(Long-chain) acyl-coa synthetase 5	EC 6.2.1.3	2.81E+03
	Beta-oxidation			
6	v_{CPT1}	Carnitinpalmitoyltransferase I	EC 2.3.1.21	1.80E+01
7	v_{CACT}	Carnitin-Acylcarnitin translocase	TCDB 2.A.29.8	1.80E+11
8	v_{CPT2}	Carnitinpalmitoyltransferase 2	EC 2.3.1.21	2.70E+06
9	$v_{c4coa-scdh}$	Short chain acyl-coa dehydrogenase (c4)	EC 1.3.8.1	7.20E+06
	$v_{c5coa-scdh}$	short chain acyl-coa dehydrogenase (c5)	EC 1.3.8.1	3.60E+05
10	$v_{c6coa-mcdh}$	medium chain acyl-coa dehydrogenase (c6)	EC 1.3.8.7	3.78E+06
	$v_{c8coa-mcdh}$	medium chain acyl-coa dehydrogenase (c8)	EC 1.3.8.7	4.50E+06
	$v_{c10coa-mcdh}$	medium chain acyl-coa dehydrogenase (c10)	EC 1.3.8.7	8.64E+05
	$v_{c12coa-mcdh}$	medium chain acyl-coa dehydrogenase (c12)	EC 1.3.8.7	7.02E+05
11	$v_{c10coa-lcdh}$	long chain acyl-coa dehydrogenase (c10)	EC 1.3.8.8	8.64E+05
	$v_{c12coa-lcdh}$	long chain acyl-coa dehydrogenase (c12)	EC 1.3.8.8	7.02E+05
	$v_{c14coa-lcdh}$	long chain acyl-coa dehydrogenase (c14)	EC 1.3.8.8	3.78E+05
	$v_{c16coa-lcdh}$	long chain acyl-coa dehydrogenase (c16)	EC 1.3.8.8	8.64E+04
12	$v_{ehyd-ec4}$	Enoyl-coa hydratase (Crontonase) (ec4)	EC 4.2.1.17	1.80E+08
	$v_{ehyd-ec5}$	Enoyl-coa hydratase (Crontonase) (ec5)	EC 4.2.1.17	9.54E+10
	$v_{ehyd-ec6}$	Enoyl-coa hydratase (Crontonase) (ec6)	EC 4.2.1.17	9.54E+08
	$v_{ehyd-ec8}$	Enoyl-coa hydratase (Crontonase) (ec8)	EC 4.2.1.17	7.20E+08
	$v_{ehyd-ec10}$	Enoyl-coa hydratase (Crontonase) (ec10)	EC 4.2.1.17	6.48E+08
	$v_{ehyd-ec12}$	Enoyl-coa hydratase (Crontonase) (ec12)	EC 4.2.1.17	3.78E+08
	$v_{ehyd-ec14}$	Enoyl-coa hydratase (Crontonase) (ec14)	EC 4.2.1.17	2.68E+08
	$v_{ehyd-ec16}$	Enoyl-coa hydratase (Crontonase) (ec16)	EC 4.2.1.17	2.88E+09
13	$v_{3hdh-lc4}$	3-hydroxyacyl-coa dehydrogenase (lc4)	EC 1.1.1.35	4.14E+15
	$v_{3hdh-lc5}$	3-hydroxyacyl-coa dehydrogenase (lc5)	EC 1.1.1.35	4.14E+12
	$v_{3hdh-lc6}$	3-hydroxyacyl-coa dehydrogenase (lc6)	EC 1.1.1.35	4.14E+12
	$v_{3hdh-lc8}$	3-hydroxyacyl-coa dehydrogenase (lc8)	EC 1.1.1.35	4.14E+12
	$v_{3hdh-lc10}$	3-hydroxyacyl-coa dehydrogenase (lc10)	EC 1.1.1.35	4.14E+12

	$v_{3hdh-lc12}$	3-hydroxyacyl-coa dehydrogenase (lc12)	EC 1.1.1.35	4.14E+12
	$v_{3hdh-lc14}$	3-hydroxyacyl-coa dehydrogenase (lc14)	EC 1.1.1.35	4.14E+12
	$v_{3hdh-lc16}$	3-hydroxyacyl-coa dehydrogenase (lc16)	EC 1.1.1.35	4.14E+12
14	v_{3kt}^{kc4coa}	3-ketoacyl-coa thiolase (kc4)	EC 2.3.1.16	1.80E+10
15	v_{3kt}^{kc5coa}	3-ketoacyl-coa thiolase (kc5)	EC 2.3.1.16	1.62E+07
	v_{3kt}^{kc6coa}	3-ketoacyl-coa thiolase (kc6)	EC 2.3.1.16	1.60E+07
	v_{3kt}^{kc8coa}	3-ketoacyl-coa thiolase (kc8)	EC 2.3.1.16	1.68E+07
	$v_{3kt}^{kc10coa}$	3-ketoacyl-coa thiolase (kc10)	EC 2.3.1.16	1.66E+07
	$v_{3kt}^{kc12coa}$	3-ketoacyl-coa thiolase (kc12)	EC 2.3.1.16	1.54E+07
	$v_{3kt}^{kc14coa}$	3-ketoacyl-coa thiolase (kc14)	EC 2.3.1.16	9.00E+06
	$v_{3kt}^{kc16coa}$	3-ketoacyl-coa thiolase (kc16)	EC 2.3.1.16	8.37E+06
16	v_{pcc}	Propionyl-coa carboxylase	EC 6.4.1.3	1.80E+04
17	v_{mmrm}	Methylmalonyl-coa racemase	EC 5.1.99.1	1.80E+04
18	v_{mmm}	Methylmalonyl-coa mutase	EC 5.4.99.2	1.80E+04
19	$v_{ETF-FAD}$	ETF-FAD		1.80E+05
20	v_{ETF-QO}	ETF-QO		9.00E+07
	Citric acid cycle			
21	v_{pdhc}	Pyruvate dehydrogenase complex	EC 1.8.1.4; EC 1.2.4.1; EC 2.3.1.12	1.44E+04
22	v_{cs}	Citrate synthase	EC 2.3.3.1	1.44E+05
23	v_{ac}	Aconitase	EC 4.2.1.3	3.60E+07
24	v_{idh}	NAD-dependent isocitrate dehydrogenase	EC 1.1.1.41	1.08E+04
25	v_{kgdhc}	α-ketogluterate dehydrogenase	EC 1.2.4.2; EC 2.3.1.61; EC 1.8.1.4	1.08E+04
26	$v_{scs-atp}$	Succinyl-Coa Synthetase (ATP)	EC 6.2.1.4; EC 6.2.1.5	0
27	$v_{scs-gtp}$	Succinyl-Coa Synthetase (GTP)	EC 6.2.1.4; EC 6.2.1.5	8.28E+07
28	v_{succdh}	Succinate dehydrogenase	EC 1.3.5.1	1.08E+03
29	v_{fum}	Fumerase	EC 4.2.1.2	7.20E+07
30	$v_{mdh_{mito}}$	Malate dehydrogenase (mitochondrial)	EC 1.1.1.37	3.60E+07
31	v_{tdh}	Transdehydrogenase	EC 1.6.1.2	1.44E+06
	Mitochondrial electrophysiology and ATP synthesis			
32	$I_{cl_{ed}}$	Chloride electro diffusion		
33	I_{na}^{pump}	Sodium pump		5.90E-01
34	$I_{na_{ed}}$	Sodium electro diffusion		
35	I_{k}^{pump}	Potassium pump		8.86E-02

36	$I_{k_{ed}}$	Potassium electro diffusion		
37	v_{F0F1}	F0F1 synthetase	EC 3.6.3.14	2.95E-03
38	v_{nex}	ATP-ADP nucleotide exchanger	TCDB 2.A.29	3.54E-03
39	v_{P-ex}	Phosphate exchanger	TCDB 2.A.29.4	1.02E+01
40	v_{cxI}	Complex I	EC 1.6.5.3	4.13E-04
41	v_{cxIII}	Complex III	EC 1.10.2.2	8.86E-03
42	v_{cxIV}	Complex IV	EC 1.9.3.1	1.12E-03
43	$v_{ak_{cyt}}$	Adenylate kinase	EC 2.7.4.3	2.95E+07
44	v_{ppase}	Pyrophosphatase	EC 3.6.1.1	2.95E+02
45	$v_{atp-usage}$	ATP usage		2.21E+02
46	$v_{O_{2diff}}$	O2 diffusion		2.95E+05
	I_H^{pump}	Proton pump		
47	$I_{H_{ed}}$	Proton electro diffusion		
	Glycolysis			
48	v_{gluT2}	Glut2 glucose transporter (Glut2)	TCDB 2.A.1.1	8.10E+01
49	v_{Gk}	Glucokinase	EC 2.7.1.2	5.18E+04
50	v_{hkIII}	Hexokinase III	EC 2.7.1.1	1.62E+00
51	$v_{glc6pT_{er}}$	Glucose-6-phosphate transport to ER		3.24E+05
52	$v_{glc6pp_{er}}$	D-Glucose-6-phosphate phosphatase	EC 3.1.3.9	2.84E+03
53	$v_{glcT_{er}}$	Glucose transport to ER		1.62E+06
54	$v_{pT_{er}}$	phosphate transport to ER		3.24E+04
55	v_{gpi}	D-Glucose-6-phosphate isomerase (Gpi)	EC 5.3.1.9	4.86E+04
56	v_{pfk2}	Phosphofructokinase 2 (Pfk2)	EC 2.7.1.105; EC 3.1.3.46	3.97E+01
57	v_{fbp2}	Phosphofructokinase 2 (FBP2)	EC 2.7.1.105; EC 3.1.3.46	1.17E+03
58	v_{pfk1}	Phosphofructokinase 1 (Pfk1)	EC 2.7.1.11	8.10E+06
59	v_{fbp1}	Fructose-1,6-bisphosphatase (Fbp1)	EC 3.1.3.11	1.30E+03
60	v_{ald}	Aldolase (Ald)	EC 4.1.2.13	1.62E+06
61	v_{tpi}	Triosephosphate isomerase (Tpi)	EC 5.3.1.1	1.62E+06
62	v_{gapdh}	D-Glyceraldehyde-3-phosphate:NAD$^+$ oxidoreductase (Gapdh)	EC 1.2.1.12	2.43E+08
63	v_{pgk}	Phosphyglyceratekinase (Pgk)	EC 2.7.2.3	8.10E+08
64	v_{pgm}	2-Phospho-D-glycerate 2,3 phosphomutase (Pgm)	EC 5.4.2.1	8.10E+08
65	v_{eno}	2-Phospho-D-glycerate hydrolase (Eno)	EC 4.2.1.11	1.62E+06
66	v_{pk}	Pyruvate kinase (Pk)	EC 2.7.1.40	1.62E+04
67	v_{pepck}	Phosphoenolpyruvate carboxykinase (Pepck)	EC 4.1.1.32	1.62E+08

68	$v_{pepck_{mito}}$	mitochondrial phosphoenolpyruvate carboxykinase (pepck)	EC 4.1.1.32	1.62E+08
69	v_{pc}	Pyruvate carboxylase (Pc)	EC 6.4.1.1	2.43E+03
70	v_{ldh}	Lactate dehydrogenase (Ldh)	EC 1.1.1.27	1.30E+14
71	v_{lacT}	Lactate transport (LacT)	TCDB 2.A.1.13	6.69E+02
	v_{pyrT}	pyruvate transport (pyrT)	TCDB 2.A.1.13	1.02E+03
72	$v_{pyrT_{mito}}$	Mitochondrial pyruvate transport	TCDB 2.A.1.13	1.62E+08
73	v_{malT}	Mitochondrial malate-phosphate transport	TCDB 2.A.29.2.7	0
74	$v_{mal-pyrT}$	Malate-pyruvate antiport (MalPyrT)		1.62E+06
75	v_{mdh}	cytosolic malate dehydrogenase (Mdh)	EC 1.1.1.37	1.62E+06
76	v_{me}	NADP dependent malic enzyme (cytosol)	EC 1.1.1.40	1.62E+03
77	v_{pepT}	Phosphoenolpyruvate transporter		1.62E+06
78	$v_{ndk_{cyt}}$	Nudiki (cytosolic) (gdp)	EC 2.7.4.6	1.62E+05
	$v_{ndk_{cyt}}^{udp}$	Nudiki (cytosolic) (udp)	EC 2.7.4.6	4.32E+05
79	$v_{ndk_{mito}}$	Nudiki (mito) (gdp)	EC 2.7.4.6	1.62E+07
	Glycogen metabolism			
80	v_{gpm}	alpha-D-Glucose 1-phosphate 1,6-phosphomutase	EC 5.4.2.2	8.64E+07
81	v_{upgase}	UTP:Glucose-1-phosphate uridylyltransferase (UPGase)	EC 2.7.7.9	6.48E+04
82	v_{gs}	Glycogen synthase (GS)	EC 2.4.1.11	1.30E+02
83	v_{gp}	Glycogen-phosphorylase (GP)	EC 2.4.1.1	1.30E+02
	Mal-Asp shuttle			
84	$v_{asat_{mito}}$	aspartate –amino transferase (mitochondrial)	EC 2.6.1.1	3.60E+07
85	v_{asat}	aspartate –amino transferase (cytosolic)	EC 2.6.1.1	3.60E+07
86	v_{agc}	aspartate –glutamate carrier	TCDB 2.A.29.14	5.40E+04
87	v_{mac}	Malate – α-ketogluterate carrier	TCDB 2.A.29.2	3.60E+05
88	v_{g3pdh}	Glycerol-3-phosphate dehydrogenase (cytosolic)	EC 1.1.1.8	3.60E+10
89	$v_{g3pdh_{mito}}$	Glycerol-3-phosphate dehydrogenase (mitochondrial)	EC 1.1.5.3	1.80E-07
	PPP			
90	v_{g6pdh}	Glucose-6-phopshate dehydrogenase	EC 1.1.1.49	1.80E+02

91	v_{pgls}	6-Phosphogluconolactase	EC 3.1.1.31	1.08E+03
92	v_{pgdh}	6-Phosphogluconate dehydrogenase	EC 1.1.1.44	3.60E+06
93	v_{rpe}	Ribulose-phosphate-3-epimerase	EC 5.1.3.1	1.80E+05
94	v_{rpi}	Ribose-phosphate-isomerase	EC 5.3.1.6	3.60E+05
95	v_{taldo}	Transladolase	EC 2.2.1.2	3.60E+05
96	v_{tketo1}	Transketolase 1	EC 2.2.1.1	3.60E+09
97	v_{tketo2}	Transketolase 2	EC 2.2.1.1	3.60E+07
	Fatty acid synthesis			
98	$v_{cit-mal}$	Citrate-malate exchanger	TCDB 2.A.29.7	2.70E+04
99	$v_{cit-lys}$	ATP dependent citrate lyase	EC 2.3.3.8	9.00E+01
100	v_{acc1}	Acetyl-CoA carboxylase 1	EC 6.4.1.2	3.51E+01
101	v_{acc2}	Acetyl-CoA carboxylase 2	EC 6.4.1.2	3.96E-01
102	v_{mdc2}	Malonyl-CoA decarboxylase 2	EC 4.1.1.9	1.44E+01
103	v_{fas-c4}	Fatty acid synthase c2->c4	EC 2.3.1.85	1.08E+02
	v_{fas-c6}	Fatty acid synthase c4->c6	EC 2.3.1.85	2.16E+03
	v_{fas-c8}	Fatty acid synthase c6->c8	EC 2.3.1.85	2.16E+03
	$v_{fas-c10}$	Fatty acid synthase c8->c10	EC 2.3.1.85	2.16E+03
	$v_{fas-c12}$	Fatty acid synthase c10->c12	EC 2.3.1.85	2.16E+03
	$v_{fas-c14}$	Fatty acid synthase c12->c14	EC 2.3.1.85	2.16E+03
	$v_{fas-c16}$	Fatty acid synthase c14->c16	EC 2.3.1.85	2.16E+03
	TAG synthesis			
104	v_{glycT}	Glycerol-uptake		3.60E+02
105	v_{glycK}	Glycerol kinase	EC 2.7.1.30	1.73E+02
106	v_{gpat}	Glycerophosphate acyltransferase	EC 2.3.1.15	1.44E+02
107	v_{agpat}	Acetyl glycerol-3-phosphate acyltransferase	EC 2.3.1.51	7.20E+01
108	v_{pap}	Phosphatidic acid phosphatase	EC 3.1.3.4	4.32E+02
109	v_{dgat}	Diacylglycerol acyltransferase	EC 2.3.1.20	5.04E+02
	Urea synthesis			
110	$v_{nh_3-uptake}$	ammonia-uptake		2.70E+02
111	v_{nh_3-diff}	ammonia-diffusion		2.70E+02
112	$v_{glnT_{mito}}$	Mitochondrial glutamine transport	TCDB 2.A.18.6	9.00E+03
113	$v_{glnase-b}$	Glutaminase (bound to membrane)	EC 3.5.1.2	3.60E+01
	$v_{glnase-free}$	Glutaminase (free)	EC 3.5.1.2	1.80E+01
114	v_{gdh}	Glutamatedehydrogenase (nad - dependent)	EC 1.4.1.3	1.08E+03
115	$v_{gdh-nadp}$	Glutamatedehydrogenase (nadp – dependent)	E C 1.4.1.3	1.08E+03

116	v_{argT}	Arginine Transporter		9.00E+02
117	$v_{aglu-syn}$	Acetyl-glutamate synthesis	EC 2.3.1.1	5.40E+02
118	v_{acgluT}	Acetyl-glutamate transporter		9.00E+03
119	$v_{acglu-hyd}$	Acetyl-glutamate hydrolase	EC 3.5.1.14	4.50E+02
120	$v_{gluT_{mito}}$	Mitochondrial glutamate carrier	TCDB 2.A.29.14	4.50E+09
121	$v_{acoa-syn}$	Acetyl-CoA synthetase	EC 6.2.1.1	2.70E+00
122	v_{cps}	Carbamoyl-phosphate synthase	EC 6.3.4.16	7.43E+02
123	v_{otc}	Ornithine transcarbamylase	EC 2.1.3.3	2.70E+03
124	v_{ocT}	Ornithine Citrulline exchanger	TCDB 2.A.29.19	9.00E+08
125	v_{ass}	Argininosuccinate synthase	EC 6.3.4.5	1.62E+04
126	v_{asl}	Argininosuccinate lyase	EC 4.3.2.1	1.26E+03
127	v_{argase}	Argininase	EC 3.5.3.1	7.20E+03
128	v_{ureaT}	Urea transport	TCDB 1.A.28.1	9.00E+01
129	v_{fmT}	Fumerate-malate exchanger		9.00E+04
130	v_{aat}	Alanine amino transferase	EC 2.6.1.2	9.00E+06
131	v_{gluT}	glutamate-sodium-transporter	TCDB 2.A.3.8	3.24E+01
132	v_{alnT}	alanin-sodium-transporter	TCDB 2.A.18.6	2.82E+02
133	v_{serT}	serine-sodium-transporter	TCDB 2.A.18.6	3.38E+02
134	v_{sdh}	serinedehydrogenase	EC 1.4.1.7	3.60E+03
135	v_{glnT}	glutamine-sodium-transporter	TCDB 2.A.18.6	3.60E+01
136	$v_{gln-syn}$	Glutamine synthetase	EC 6.3.1.2	1.30E+02
	VLDL-LD synthesis			
137	$v_{LD-syn-tag}$	LD synthesis (tag)		5.40E+01
138	$v_{LD-syn-ce}$	LD synthesis (cholesterol ester)		1.08E+02
139	$v_{apoB-syn}$	ApoB synthesis		1.80E+03
140	$v_{apoB-deg}$	ApoB degradation		1.80E+04
141	v_{MTP}^{tag}	Microsomal transfer protein (tag)		9.00E+01
	v_{MTP}^{ce}	Microsomal transfer protein (cholesterol ester)		6.93E+01
142	$v_{VLDL-ex-tag}$	VLDL release (tag)		1.80E+05
	$v_{VLDL-ex-ce}$	VLDL release (cholesterol ester)		1.80E+05
143	v_{ATGL}^{tag}	ATGL	EC 3.1.1.3	3.60E+00
144	v_{HSL}^{dag}	Hormone sensitive lipase (HSL) (dag)	EC 3.1.1.79	1.80E+01
145	v_{magl}	Monoaclyglycerol lipase	EC 3.1.1.23	3.60E+01

146	v_{cee}	Cholesterol ester esterase	EC 3.1.1.13	1.08E+01
	Ketone body synthesis			
147	$v_{hmg-syn}$	Mitochondrial HMG-CoA synthase	EC 2.3.3.10	1.76E+03
148	$v_{hmg-lys}$	Mitochondrial HMG-CoA lyase	EC 4.1.3.4	2.52E+02
149	$v_{\beta hdh}$	B-Hydroxy butyrate dehydrogenase	EC 1.1.1.30	2.52E+09
150	v_{acacT}	Acetoacetate transport (mitochondrial)	TCDB 2.A.13.1	3.78E+02
151	$v_{\beta hbT}$	B-Hydroxy butyrate transport (mitochondrial)	TCDB 2.A.13.1	3.78E+02
152	$v_{acac-ex}$	Acetoacetate export (MCT1/MCT2)	TCDB 2.A.13.1	1.26E+02
153	$v_{\beta hb-ex}$	B-Hydroxy butyrate export (MCT1/MCT2)	TCDB 2.A.13.1	1.26E+02
	Cholesterol synthesis			
154	$v_{acaccoa-syn}$	Acetoacetyl-CoA synthetase	EC 6.2.1.1	4.94E-01
155	v_{3kt}^{kc4coa}	Thiolase (cytosolic, peroxisomal)	EC 2.3.1.9	2.74E+09
156	$v_{hmg-syn-cyt}$	Cytosolic HMG-CoA synthase	EC 2.3.3.10	8.23E+01
157	v_{hmgl}	HMG-CoA lyase	EC 4.1.3.4	2.74E-01
158	v_{hmgr}	HMG-CoA reductase	EC 1.1.1.34	2.88E+00
159	v_{mevk}	Mevalonate kinase	EC 2.7.1.36	2.74E+04
160	v_{pmevk}	Phopshomevalonate kinase	EC 2.7.4.2	2.74E+04
161	v_{mdp}	Mevalonate-5-pyrophopshatedecarboxylase	EC 4.1.1.33	2.74E+04
162	v_{ippi}	Isopentenylphyrophosphate isomerase	EC 5.3.3.2	2.74E+04
163	v_{gpps}	Geranyl pyrophosphate synthase	EC 2.5.1.29	2.74E+04
164	v_{fpps}	Farnesyl pyrophosphate synthase	EC 2.5.1.10	2.74E+04
165	$v_{sqs-nadph}$	Squalene synthase	EC 2.5.1.21	2.74E+04
	$v_{sqs-nadh}$	Squalene synthase	EC 2.5.1.21	2.74E+04
166	v_{sqe}	Squalene epoxidase	EC 1.14.13.132	2.74E+04
167	v_{osc}	Squalene epoxide cyclase	EC 5.4.99.7	2.74E+04
168	$v_{s14dm-lan}$	Sterol 14-Demethylase P450 (CYP51)	EC 1.14.13.70	7.19E+03
	$v_{s14dm-dihlan}$	Sterol 14-Demethylase P450 (CYP51)	EC 1.14.13.70	1.03E+04
169	$v_{s14r-ffmas}$	Sterol Δ14 reductase	EC 1.3.1.70	2.74E+04
	$v_{s14r-dihffmas}$	Sterol Δ14 reductase	EC 1.3.1.70	2.74E+04
170	$v_{mso-tmas}$	Methyl sterol oxidase (Methylsterol monooxygenase)	EC 1.14.13.72	2.74E+04
	$v_{mso-dmtmas}$	Methyl sterol oxidase (Methylsterol monooxygenase)	EC 1.14.13.72	2.74E+04

	$v_{mso-dihtmas}$	Methyl sterol oxidase (Methylsterol monooxygenase)	EC 1.14.13.72	2.74E+04
	$v_{mso-dmdihtmas}$	Methyl sterol oxidase (Methylsterol monooxygenase)	EC 1.14.13.72	2.74E+04
171	$v_{casdc-dmtmasca}$	4α-carboxylic acid sterol decarboxylase	EC 1.1.1.170	2.74E+04
	$v_{casdc-ddimtmasca}$	4α-carboxylic acid sterol decarboxylase	EC 1.1.1.170	2.74E+04
	$v_{casdc-dmdihtmasca}$	4α-carboxylic acid sterol decarboxylase	EC 1.1.1.170	2.74E+04
	$v_{casdc-ddimdihtmasca}$	4α-carboxylic acid sterol decarboxylase	EC 1.1.1.170	2.74E+04
172	$v_{3ksr-dmktmas}$	3-keto steroid reductase	EC 1.1.1.270	2.74E+04
	$v_{3ksr-ddimktmas}$	3-keto steroid reductase	EC 1.1.1.270	2.74E+04
	$v_{3ksr-ddimkdihtmas}$	3-keto steroid reductase	EC 1.1.1.270	2.74E+04
	$v_{3ksr-dmkdihtmas}$	3-keto steroid reductase	EC 1.1.1.270	2.74E+04
173	$v_{s78i-zym}$	Steroid7,8 isomerase (cholesterol Δ isomerase)	EC 5.3.3.5	2.74E+04
	$v_{s78i-zymostenol}$	Steroid7,8 isomerase (cholesterol Δ isomerase)	EC 5.3.3.5	2.74E+04
174	$v_{lathox-lath}$	Lathosterol oxidase (assumed to be member of cytochrome P450 superfamily)	EC 1.21.14.6	2.74E+04
	$v_{lathox-dhlath}$	Lathosterol oxidase (assumed to be member of cytochrome P450 superfamily)	EC 1.21.14.6	2.74E+04
175	$v_{s7r-dhchol}$	Sterol Δ7 reductase (cytochrome P450 oxidoreductase)	EC 1.3.1.21	2.74E+04
	$v_{s7r-dhdesm}$	Sterol Δ7 reductase (cytochrome P450 oxidoreductase)	EC 1.3.1.21	2.74E+04
176	$v_{s24r-lan}$	Sterol Δ24 reductase	EC 1.3.1.72	2.74E+04
	$v_{s24r-ffmas}$	Sterol Δ24 reductase	EC 1.3.1.72	2.74E+04
	$v_{s24r-tmas}$	Sterol Δ24 reductase	EC 1.3.1.72	2.74E+04
	$v_{s24r-zym}$	Sterol Δ24 reductase	EC 1.3.1.72	2.74E+04
	$v_{s24r-dhlath}$	Sterol Δ24 reductase	EC 1.3.1.72	2.74E+04
	$v_{s24r-dhdesmo}$	Sterol Δ24 reductase	EC 1.3.1.72	2.74E+04
	$v_{s24r-desmo}$	Sterol Δ24 reductase	EC 1.3.1.72	2.74E+04
177	v_{acat}	Acyl-CoA cholesterol Acyltransferase	EC 2.3.1.26	2.74E+04
	Alcohol metabolism			
178	v_{ethT}	Ethanol uptake		2.88E+02
179	v_{adh}	Alcohol dehydrogenase	EC 1.1.1.1	1.01E+02
180	v_{alddhI}	Aldehyde dehydrogenase I	EC 1.2.1.3; EC 1.2.1.5	1.58E-01
181	$v_{alddhII}$	Aldehyde dehydrogenase II	EC 1.2.1.3; EC 1.2.1.5	1.01E-01
182	v_{aaldT}	Acetaldehyde transport		1.44E+10
183	$v_{alddh_{mito}}$	Mitochondrial Aldehyde dehydrogenase	EC 1.2.1.3; EC	1.44E+03

			1.2.1.5	
184	v_{aceT}	Acetate transport	TCDB 2.A.1.13	1.44E+03
185	$v_{aceT\,mito}$	Acetate transport (mito)	TCDB 2.A.1.13	1.44E+03
	Galactose metabolism			
186	v_{galT}	Galactose uptake	TCDB 2.A.1.1	6.48E+01
187	v_{aldor}	Aldose reductase	EC 1.1.1.21	4.32E+01
188	v_{galolT}	Galactitol transport		4.32E+02
189	v_{galdh}	Galactosedehydrogenase	EC 1.1.1.48	4.32E+01
190	v_{galaT}	Galactonase transport		4.32E+02
191	v_{guldh}	Gulonate dehydrogenase	EC 1.1.1.45	4.32E+02
192	v_{galdc}	Decarboxylation β-keto galactonic acid		4.32E+02
193	v_{xylk}	Xylulose kinase	EC 2.7.1.17	4.32E+02
194	v_{galk}	Galactokinase	EC 2.7.1.6	7.86E+02
195	v_{galt}	Galactose-1-phosphate uridyltransferase	EC 2.7.7.12	3.24E+03
196	v_{gale}	Uridine diphosphate (UDP)-galactose-4-epimerase	EC 5.1.3.2	8.64E+02
197	v_{gal1pp}	Galactose-1-phosphatase		4.32E+02
	Fructose metabolism			
198	v_{fruT}	Fructose uptake	TCDB 2.A.1.1	2.16E+01
199	v_{fruk}	Fructokinase	EC 2.7.1.4	1.01E+03
200	v_{aldB}	Aldolase B	EC 4.1.2.13	2.88E+02
201	v_{triok}	Triokinase	EC 2.7.1.28	1.15E+03
202	v_{aldr}	Aldehyde reductase	EC 1.1.1.21	7.20E+03
203	$v_{alddh-gra}$	Aldehydedehydrogenase	EC 1.2.1.3; EC 1.2.1.5	5.76E+04
204	v_{sordh}	Sorbitoldehydrogenase	EC 1.1.1.14	7.20E+02
205	v_{gck}	Glycerate kinase	EC 2.7.1.31	5.76E+04
206	v_{graT}	Glyceraldehyde transport		7.20E+02
	Valproate metabolism			
207	$v_{val-diff}$	Valproate uptake		3.60E+00
208	v_{vcs}	valproylcoa synthetase	EC 6.2.1.3	3.60E+00
6	$v_{CPT1-val}$	Carnitinpalmitoyltransferase I	EC 2.3.1.21	1.80E+00
7	$v_{CACT-val}$	Carnitin-Acylcarnitin translocase	TCDB 2.A.29.8	3.60E+02
8	$v_{CPT2-val}$	Carnitinpalmitoyltransferase 2	EC 2.3.1.21	5.40E+04
209	$v_{valcoa-bcdh}$	branched chain acyl-coa dehydrogenase (valcoa)	EC 1.3.8.5	3.60E+01
12	$v_{ehyd-evalcoa}$	Enoyl-coa hydratase (Crontonase) (e-	EC 4.2.1.17	3.60E+05

		valcoa)		
13	$v_{3hdh-lvalcoa}$	3-hydroxyacyl-coa dehydrogenase (lvalcoa)	EC 1.1.1.35	3.60E+05
14	$v_{3kt}^{kvalcoa}$	3-ketoacyl-coa thiolase (kvalcoa)	EC 2.3.1.16	3.60E+03

The complete table of infinitesimal and finite response is found in the Supplementary Data 4.

parameter	type	enzyme	affected functions
Imax_CIV	VMAX	complex IV resp. chain	/1/2/3/6/7/11/14/17/22/23/
Imax_K_pump	VMAX	potassium pump	/1/3/6/22/23/
vmax_c1	VMAX	complex I	/1/2/6/17/22/
v0_GK	VMAX	glucokinase	/1/2/6/7/22/
ki_GK_c16_coa_cyt	KA	glucokinase	/1/2/6/7/22/
P_K	VMAX	potassium permeabilty	/6/7/22/
km_Imax_F0F1_syn_Vmm	KA	F0F1-ATPase	/6/17/22/
Imax_PEX	VMAX	phosphate exchanger	/1/2/17/
n_GK	N	glucokinase	/1/2/6/
ka_GK_glc_cyt	KA	glucokinase	/1/2/6/
ni_pfk1	N	phosphofructokinase1	/1/2/17/
vmax_PC	VMAX	pyruvate carboxylase	/6/7/22/

#	function	#	function
1	gluce EF	13	alanin EF
2	lactate EF	14	urea EF
3	pyruvate EF	15	acetate EF
4	glycerol EF	16	VLDL EF
5	fatty acid EF	17	glycogen (cellular)
6	acetoacetate EF	18	triglyceride (cellular)
7	ß-hydroxy butyrate EF	19	cholesterol synthesis
8	oxygen uptake	20	fatty acid synthesis
9	ammonia EF	21	membrane potential (mito)
10	glutamine EF	22	ATP/ADP
11	glutamate EF	23	NAD/NADH
12	serin EF	24	NADP/NADPH

Supplementary Table 2 Left panel: *Parameters affecting at least three different metabolic functions with a finite response coefficient >= 1.0 at a finite parameter reduction p->p/2.* For the numbering of metabolic functions see right panel and/or legend Fig.5, main text.

parameter	type	enzyme	affected functions
ka_GK_glc_cyt	KA	glucokinase	/1/2/6/7/11/14/22/
km_Imax_F0F1_syn_Vmm	KA	F0F1-ATPase	/1/2/7/14/17/22/
n_GK	N	glucokinase	/1/2/6/7/14/22/
n2_GK	N	glucokinase	/1/2/6/7/22/
k0_GK_glc_cyt	KM	glucokinase	/1/2/6/
vmax_PDHC_np	VMAX	pyruvate dehydrogenase	/6/7/
ka_gamma_PDHC_pyr_mito	KA	pyruvate dehydrogenase	/6/7/
k0_PDHC_np_nad_mito	KM	pyruvate dehydrogenase	/6/7/
Imax_K_pump	VMAX	potassium pump	/6/7/
v0_GK	VMAX	glucokinase	/1/2/
vmax_G6P_ER	VMAX	glucose-6-phosphatase	/1/6/
ki0_atp_pfk1	KA	phosphofructokinase 1	/1/2/

Supplementary Table 3 *Parameters affecting at least three different metabolic functions with an infinitesimal response coefficients R >= 1.0 at a finite parameter increase p->1.5p.* For the numbering of metabolic functions see right panel of Supplementary Table 2 and/or legend Fig.5, main text

Supplementary Note 1 – Kinetic Rate Laws

Fatty acid uptake

Fatty acid uptake:

Carrier mediated FATP

$$v_{c16_{cyt}-uptake}^{carrier} = V_{max-carrier}^{c16_{cyt}-uptake} \cdot \frac{(ffa_{ext} - c16_{cyt})}{1 + \frac{ffa_{ext}}{K_m^{ffa_{ext}}} + \frac{c16_{cyt}}{K_m^{c16_{cyt}}}}$$

$V_{max}^{ACSc16_{cyt}-uptake}$ for numerical value see Supplementary Table 1

$K_m^{ffa_{ext}} = 0.000083$ [1]

$K_m^{c16_{cyt}} = 0.004$ [2]

Diffusive uptake [1]

$$v_{c16_{cyt}-uptake}^{diffusion} = V_{max-diffusion}^{c16_{cyt}-uptake} \cdot (ffa_{ext} - c16_{cyt})$$

$V_{max-diffusion}^{c16_{cyt}-uptake}$ for numerical value see Supplementary Table 1

(Long-chain) acyl-coa synthetase

$$v_{ACSL1} = V_{max}^{ACSL1} \cdot \frac{c16_{cyt}}{c16_{cyt} + K_m^{c16_{cyt}}} \cdot \frac{atp_{cyt}}{atp_{cyt} + K_m^{atp_{cyt}}} \cdot \frac{coa_{cyt}}{coa_{cyt} + K_m^{coa_{cyt}}}$$

V_{max}^{ACSL1} for numerical value see Supplementary Table 1

$K_m^{c16_{cyt}}$ =0.005 [3]

$K_m^{atp_{cyt}}$ =0.649 [3]

$K_m^{coa_{cyt}}$ =0.0064 [3]

$$v_{ACSL4} = V_{max}^{ACSL4} \cdot \frac{c16_{cyt}}{c16_{cyt} + K_m^{c16_{cyt}}} \cdot \frac{atp_{cyt}}{atp_{cyt} + K_m^{atp_{cyt}}} \cdot \frac{coa_{cyt}}{coa_{cyt} + K_m^{coa_{cyt}}}$$

V_{max}^{ACSL4} for numerical value see Supplementary Table 1

$K_m^{c16_{cyt}}$ =0.0054 [3]

$K_m^{atp_{cyt}}$ =0.034 [3]

$K_m^{coa_{cyt}}$ =0.0041 [3]

$$v_{ACSL5} = V_{max}^{ACSL5} \cdot \frac{c16_{cyt}}{c16_{cyt} + K_m^{c16_{cyt}}} \cdot \frac{atp_{cyt}}{atp_{cyt} + K_m^{atp_{cyt}}} \cdot \frac{coa_{cyt}}{coa_{cyt} + K_m^{coa_{cyt}}}$$

V_{max}^{ACSL5} for numerical value see Supplementary Table 1

$K_m^{c16_{cyt}}$ =0.0086 [3]

$K_m^{atp_{cyt}}$ =0.666 [3]

$K_m^{coa_{cyt}}$ =0.0024 [3]

Beta-oxidation

Carnitinpalmitoyltransferase I

$$v_{CPT1} = V_{max}^{CPT1} \cdot \frac{c16coa_{cyt} \cdot car_{cyt}}{\left(c16coa_{cyt} + K_m^{c16coa_{cyt}}\right) \cdot \left(car_{cyt} + K_m^{car_{cyt}}\right)}$$

$V_{max}^{CPT1}$for numerical value see Supplementary Table 1

$n = 2.47$ [4]

$$K_m^{c16coa_{cyt}} = K_0^{c16coa_{cyt}} \cdot \left(1 + \frac{malcoa2_{imm}}{K_i^{malcoa2_{imm}}}\right) \cdot \left(1 + \frac{valcoa_{cyt}}{K_i^{valcoa_{cyt}}}\right)$$

$K_0^{c16coa_{cyt}}$ =0.03 [5]

$K_i^{malcoa2_{imm}} =$ 0.0025 [6]

$K_m^{car_{cyt}}$ =0.032 [7]

$K_i^{valcoa} = 0.057$ [8]

Carnitin-Acylcarnitin translocase

$$v_{CACT} = V_{max}^{CACT} \cdot \left(\frac{car_{mito} \cdot c16car_{cyt} - {}^{1}/_{K_{eq}^{CACT}} \cdot car_{cyt} \cdot c16car_{mito}}{\left(1 + \frac{car_{mito}}{K_m^{car_{mito}}}\right)\left(1 + \frac{c16car_{cyt}}{K_m^{c16coa_{cyt}}}\right) + \left(1 + \frac{car_{cyt}}{K_m^{car_{cyt}}}\right)\left(1 + \frac{c16car_{mito}}{K_m^{c16car_{mito}}}\right) - 1} \right)$$

V_{max}^{CACT} for numerical value see Supplementary Table 1

$K_{eq}^{CACT} = 1.6$ [9]

$K_m^{car_{mito}} = 5.8$ [10]
$K_m^{c16car_{cyt}}$ =0.0015 [11]

$K_m^{car_{cyt}} = 0.51$ [10]

$K_m^{c16car_{mito}} = 0.0051$ [12]

Carnitinpalmitoyltransferase 2

$$v_{CPT2} = V_{max}^{CPT2} \cdot \left(\frac{c16car_{mito} \cdot coa_{mito} - 1/K_{eq}^{CPT2} \cdot c16coa_{mito} \cdot car_{mito}}{\left(1 + \frac{c16car_{mito}}{K_m^{c16car_{mito}}}\right)\left(1 + \frac{coa_{mito}}{K_m^{coa_{mito}}}\right) + \left(1 + \frac{c16coa_{mito}}{K_m^{c16coa_{mito}}}\right)\left(1 + \frac{car_{mito}}{K_m^{car_{mito}}}\right) - 1} \right)$$

V_{max}^{CPT2} for numerical value see Supplementary Table 1

$K_{eq}^{CPT2} = 2$ [13]

$K_m^{c16car_{mito}} =0.12$ [6]

$K_m^{coa_{mito}} = 0.0055$ [14]

$K_m^{c16coa_{mito}} =0.191$ [15]

$K_m^{car_{mito}} =0.121$ [15]

Short chain acyl-coa dehydrogenase (c4)

$$v_{c4coa-scdh} = V_{max}^{c4coa-dh} \cdot \left(\frac{c4coa_{mito}}{c4coa_{mito} + K_m^{c4coa_{mito}}} \right) \cdot \left(\frac{etffad_{mito}}{etffad_{mito} + K_m^{etffad_{mito}}} \right)$$

$V_{max}^{c4coa-dh}$ for numerical value see Supplementary Table 1

$K_m^{c4coa_{mito}} = 0.0107$ [16]

$K_m^{etffad_{mito}} = 0.0038$ [16]

short chain acyl-coa dehydrogenase (c5)

$$v_{c5coa-scdh} = V_{max}^{c5coa-dh} \cdot \left(\frac{c5coa_{mito}}{c5coa_{mito} + K_m^{c5coa_{mito}}} \right) \cdot \left(\frac{etffad_{mito}}{etffad_{mito} + K_m^{etffad_{mito}}} \right)$$

$V_{max}^{c5coa-dh}$ for numerical value see Supplementary Table 1

$K_m^{c5coa_{mito}} = 0.01$

$K_m^{etffad_{mito}} = 0.0038$ [16]

medium chain acyl-coa dehydrogenase (c6)

$$v_{c6coa-mcdh} = V_{max}^{c6coa-dh} \cdot \left(\frac{c6coa_{mito}}{c6coa_{mito} + K_m^{c6coa_{mito}}}\right) \cdot \left(\frac{etffad_{mito}}{etffad_{mito} + K_m^{etffad_{mito}}}\right)$$

$V_{max}^{c6coa-dh}$ for numerical value see Supplementary Table 1

$K_m^{c6_{coa_{mito}}} = 0.0094$ [16]

$K_m^{etffad_{mito}} = 0.0045$ [16]

medium chain acyl-coa dehydrogenase (c8)

$$v_{c8coa-mcdh} = V_{max}^{c8coa-dh} \cdot \left(\frac{c8coa_{mito}}{c8coa_{mito} + K_m^{c8coa_{mito}}}\right) \cdot \left(\frac{etffad_{mito}}{etffad_{mito} + K_m^{etffad_{mito}}}\right)$$

$V_{max}^{c8coa-dh}$ for numerical value see Supplementary Table 1

$K_m^{c8coa_{mito}} = 0.004$ [16]

$K_m^{etffad_{mito}} = 0.0045$ [16]

medium chain acyl-coa dehydrogenase (c10)

$$v_{c10coa-mcdh} = V_{max}^{c10coa-dh} \cdot \left(\frac{c10coa_{mito}}{c10coa_{mito} + K_m^{c10coa_{mito}}}\right) \cdot \left(\frac{etffad_{mito}}{etffad_{mito} + K_m^{etffad_{mito}}}\right)$$

$V_{max}^{c10coa-dh}$ for numerical value see Supplementary Table 1

$K_m^{c10coa_{mito}} = 0.0054$ [16]

$K_m^{etffad_{mito}} = 0.0045$ [16]

medium chain acyl-coa dehydrogenase (c12)

$$v_{c12coa-mcdh} = V_{max}^{c12coa-dh} \cdot \left(\frac{c12coa_{mito}}{c12coa_{mito} + K_m^{c12coa_{mito}}}\right) \cdot \left(\frac{etffad_{mito}}{etffad_{mito} + K_m^{etffad_{mito}}}\right)$$

$V_{max}^{c12coa-dh}$ for numerical value see Supplementary Table 1

$K_m^{c12coa_{mito}} =0.0057$ [16]

$K_m^{etffad_{mito}} = 0.0045$ [16]

long chain acyl-coa dehydrogenase (c10)

$$v_{c10coa-lcdh} = V_{max}^{c10coa-dh} \cdot \left(\frac{c10coa_{mito}}{c10coa_{mito} + K_m^{c10coa_{mito}}}\right) \cdot \left(\frac{etffad_{mito}}{etffad_{mito} + K_m^{etffad_{mito}}}\right)$$

$V_{max}^{c10coa-dh}$ for numerical value see Supplementary Table 1

$$K_m^{c10coa_{mito}} = K_0^{c10coa_{mito}} \cdot \left(1 + \frac{kc16coa_{mito}}{K_i^{kc16coa_{mito}}}\right)$$

$K_i^{kc16coa_{mito}} = 0.00047$ [17]

$K_0^{c10coa_{mito}} = 0.0243$ [16]

$K_m^{etffad_{mito}} = 0.0083$ [16]

long chain acyl-coa dehydrogenase (c12)

$$v_{c12coa-lcdh} = V_{max}^{c12coa-dh} \cdot \left(\frac{c12coa_{mito}}{c12coa_{mito} + K_m^{c12coa_{mito}}}\right) \cdot \left(\frac{etffad_{mito}}{etffad_{mito} + K_m^{etffad_{mito}}}\right)$$

$V_{max}^{c12coa-dh}$ for numerical value see Supplementary Table 1

$$K_m^{c12coa_{mito}} = K_0^{c12coa_{mito}} \cdot \left(1 + \frac{kc16coa_{mito}}{K_i^{kc16coa_{mito}}}\right)$$

$K_i^{kc16coa_{mito}} = 0.00047$ [17]

$K_0^{c12coa_{mito}} = 0.009$ [16]

$K_m^{etffad_{mito}} = 0.0083$ [16]

long chain acyl-coa dehydrogenase (c14)

$$v_{c14coa-lcdh} = V_{max}^{c14coa-dh} \cdot \left(\frac{c14coa_{mito}}{c14coa_{mito} + K_m^{c14coa_{mito}}}\right) \cdot \left(\frac{etffad_{mito}}{etffad_{mito} + K_m^{etffad_{mito}}}\right)$$

$V_{max}^{c14coa-dh}$ for numerical value see Supplementary Table 1

$$K_m^{c14coa_{mito}} = K_0^{c14coa_{mito}} \cdot \left(1 + \frac{kc16coa_{mito}}{K_i^{kc16coa_{mito}}}\right)$$

$K_i^{kc16coa_{mito}} = 0.00047$ [17]

$K_0^{c14coa_{mito}} = 0.0074$ [16]

$K_m^{etffad_{mito}} = 0.0083$ [16]

long chain acyl-coa dehydrogenase (c16)

$$v_{c16coa-lcdh} = V_{max}^{c16coa-dh} \cdot \left(\frac{c16coa_{mito}}{c16coa_{mito} + K_m^{c16coa_{mito}}}\right) \cdot \left(\frac{etffad_{mito}}{etffad_{mito} + K_m^{etffad_{mito}}}\right)$$

$V_{max}^{c16coa-dh}$ for numerical value see Supplementary Table 1

$$K_m^{c16coa_{mito}} = K_0^{c16coa_{mito}} \cdot \left(1 + \frac{kc16coa_{mito}}{K_i^{kc16coa_{mito}}}\right)$$

$K_i^{kc16coa_{mito}} = 0.00047$ [17]

$K_0^{c16coa_{mito}} = 0.0025$ [16]

$K_m^{etffad_{mito}} = 0.0083$ [16]

Enoyl-coa hydratase (Crontonase) (ec4)

$$v_{ehyd-ec4} = V_{max}^{ehyd-ec4} \cdot \left(\frac{ec4coa_{mito} - {}^{1}\!/\!{}_{K_{eq}^{ehyd-ec4}} \cdot lc4coa_{mito}}{ec4coa_{mito} + K_m^{ec4coa_{mito}}} \right)$$

$V_{max}^{ehyd-ec4}$ for numerical value see Supplementary Table 1

$K_{eq}^{ehyd-ec4} = 0.25$ [18]

$$K_m^{ec4coa_{mito}} = K_0^{ec4coa_{mito}} \cdot \left(1 + \frac{kc4coa_{mito}}{K_i^{kc4coa_{mito}}}\right)$$

$K_0^{ec4coa_{mito}} = 0.04$ [19]

$K_i^{kc4coa_{mito}} = 0.025$ [19]

Enoyl-coa hydratase (Crontonase) (ec5)

$$v_{ehyd-ec5} = V_{max}^{ehyd-ec5} \cdot \left(\frac{ec5coa_{mito} \; {}^{1}\!/\!{}_{K_{eq}^{ehyd-ec5}} \cdot lc5coa_{mito}}{ec5coa_{mito} + K_m^{ec5coa_{mito}}} \right)$$

$V_{max}^{ehyd-ec5}$ for numerical value see Supplementary Table 1

$K_{eq}^{ehyd-ec5} = 2$ [18]

$$K_m^{ec5coa_{mito}} = K_0^{ec5coa_{mito}} \cdot \left(1 + \frac{kc4coa_{mito}}{K_i^{kc4coa_{mito}}}\right)$$

$K_0^{ec5coa_{mito}} = 0.025$

$K_i^{kc4coa_{mito}} = 0.025$ [19]

Enoyl-coa hydratase (Crontonase) (ec6)

$$v_{ehyd-ec6} = V_{max}^{ehyd-ec6} \cdot \left(\frac{ec6coa_{mito} - {}^{1}\!/_{K_{eq}^{ehyd-ec6}} \cdot lc6coa_{mito}}{ec6coa_{mito} + K_m^{ec6coa_{mito}}} \right)$$

$V_{max}^{ehyd-ec6}$ for numerical value see Supplementary Table 1

$K_{eq}^{ehyd-ec6} = 2$ [18]

$$K_m^{ec6coa_{mito}} = K_0^{ec6_{coa_{mito}}} \cdot \left(1 + \frac{kc4coa_{mito}}{K_i^{kc4coa_{mito}}}\right)$$

$K_0^{ec6coa_{mito}} = 0.025$ [19]

$K_i^{kc4coa_{mito}} = 0.025$ [19]

Enoyl-coa hydratase (Crontonase) (ec8)

$$v_{ehyd-ec8} = V_{max}^{ehyd-ec8} \cdot \left(\frac{ec8coa_{mito} - {}^{1}\!/_{K_{eq}^{ehyd-ec8}} \cdot lc8coa_{mito}}{ec8coa_{mito} + K_m^{ec8coa_{mito}}} \right)$$

$V_{max}^{ehyd-ec8}$ for numerical value see Supplementary Table 1

$K_{eq}^{ehyd-ec8} = 2$ [18]

$$K_m^{ec8coa_{mito}} = K_0^{ec8coa_{mito}} \cdot \left(1 + \frac{kc4coa_{mito}}{K_i^{kc4coa_{mito}}}\right)$$

$K_0^{ec8coa_{mito}} = 0.025$ [19]

$K_i^{kc4coa_{mito}} = 0.025$ [19]

Enoyl-coa hydratase (Crontonase) (ec10)

$$v_{ehyd-ec10} = V_{max}^{ehyd-ec10} \cdot \left(\frac{ec10coa_{mito} - {}^{1}\!/_{K_{eq}^{ehyd-ec10}} \cdot lc10coa_{mito}}{ec10coa_{mito} + K_m^{ec10coa_{mito}}} \right)$$

$V_{max}^{ehyd-ec10}$ for numerical value see Supplementary Table 1

$K_{eq}^{ehyd-ec10} = 2$ [18]

$$K_m^{ec10coa_{mito}} = K_0^{ec10coa_{mito}} \cdot \left(1 + \frac{kc4coa_{mito}}{K_i^{kc4coa_{mito}}}\right)$$

$K_0^{ec10coa_{mito}} = 0.025$ [19]

$K_i^{kc4coa_{mito}} = 0.025$ [19]

Enoyl-coa hydratase (Crontonase) (ec12)

$$v_{ehyd-ec12} = V_{max}^{ehyd-ec12} \cdot \left(\frac{ec12coa_{mito} - {}^{1}\!/_{K_{eq}^{ehyd-ec12}} \cdot lc12coa_{mito}}{ec12coa_{mito} + K_m^{ec12coa_{mito}}}\right)$$

$V_{max}^{ehyd-ec12}$ for numerical value see Supplementary Table 1

$K_{eq}^{ehyd-ec12} = 2$ [18]

$$K_m^{ec12coa_{mito}} = K_0^{ec12coa_{mito}} \cdot \left(1 + \frac{kc4coa_{mito}}{K_i^{kc4coa_{mito}}}\right)$$

$K_0^{ec12coa_{mito}} = 0.025$ [19]

$K_i^{kc4coa_{mito}} = 0.025$ [19]

Enoyl-coa hydratase (Crontonase) (ec14)

$$v_{ehyd-ec14} = V_{max}^{ehyd-ec14} \cdot \left(\frac{ec14coa_{mito} - {}^{1}\!/_{K_{eq}^{ehyd-ec14}} \cdot lc14coa_{mito}}{ec14coa_{mito} + K_m^{ec14coa_{mito}}}\right)$$

$V_{max}^{ehyd-ec14}$ for numerical value see Supplementary Table 1

$K_{eq}^{ehyd-ec14} = 2$ [18]

$$K_m^{ec14coa_{mito}} = K_0^{ec14coa_{mito}} \cdot \left(1 + \frac{kc4coa_{mito}}{K_i^{kc4coa_{mito}}}\right)$$

$K_0^{ec14coa_{mito}} = 0.025$ [19]

$K_i^{kc4coa_{mito}} = 0.025$ [19]

Enoyl-coa hydratase (Crontonase) (ec16)

$$v_{ehyd-ec16} = V_{max}^{ehyd-ec16} \cdot \left(\frac{ec16coa_{mito} \, {}^{1}\!/_{K_{eq}^{ehyd-ec16}} \cdot lc16coa_{mito}}{ec16coa_{mito} + K_m^{ec16coa_{mito}}} \right)$$

$V_{max}^{ehyd-ec16}$ for numerical value see Supplementary Table 1

$K_{eq}^{ehyd-ec16} = 2$ [18]

$$K_m^{ec16coa_{mito}} = K_0^{ec16coa_{mito}} \cdot \left(1 + \frac{kc4coa_{mito}}{K_i^{kc4coa_{mito}}}\right)$$

$K_0^{ec16coa_{mito}} = 0.15$ [19]

$K_i^{kc4coa_{mito}} = 0.025$ [19]

3-hydroxyacyl-coa dehydrogenase (lc4)

$$v_{3hdh-lc4} = V_{max}^{3hdh-lc4} \cdot \left(\frac{lc4coa_{mito} \cdot nad_{mito} - {}^{1}\!/_{K_{eq}^{3hdh-lc4}} \cdot kc4coa_{mito} \cdot nadh_{mito}}{\left(1 + \frac{lc4coa_{mito}}{K_m^{lc4coa_{mito}}}\right) \cdot \left(1 + \frac{nad_{mito}}{K_m^{nad_{mito}}}\right) + \left(1 + \frac{kc4coa_{mito}}{K_m^{kc4coa_{mito}}}\right) \cdot \left(1 + \frac{nadh_{mito}}{K_m^{nadh_{mito}}}\right) - 1} \right)$$

$V_{max}^{3hdh-lc4}$ for numerical value see Supplementary Table 1

$K_{eq}^{3hdh-lc4} = \frac{1}{0.012}$ [20]

$K_m^{lc4coa_{mito}}$ =0.0699 [21]

$K_m^{nad_{mito}}$ =0.0585 [21]

$K_m^{kc4coa_{mito}} = 0.0169$ [21]

$K_m^{nadh_{mito}} = 0.0054$ [21]

3-hydroxyacyl-coa dehydrogenase (lc5coa)

$$v_{3hdh-lc5} = V_{max}^{3hdh-lc5} \cdot \left(\frac{lc5coa_{mito} \cdot nad_{mito} - {}^{1}\!/\!{}_{K_{eq}^{3hdh-lc5}} \cdot kc5coa_{mito} \cdot nadh_{mito}}{\left(1 + \frac{lc5coa_{mito}}{K_m^{lc5coa_{mito}}}\right) \cdot \left(1 + \frac{nad_{mito}}{K_m^{nad_{mito}}}\right) + \left(1 + \frac{kc5coa_{mito}}{K_m^{kc5coa_{mito}}}\right) \cdot \left(1 + \frac{nadh_{mito}}{K_m^{nadh_{mito}}}\right) - 1} \right)$$

$V_{max}^{3hdh-lc5}$ for numerical value see Supplementary Table 1

$K_{eq}^{3hdh-lc5} = \frac{1}{10^{-3}}$

$K_m^{lc5coa_{mito}}$ =0.04

$K_m^{nad_{mito}}$ =0.0585 [21]

$K_m^{kc5coa_{mito}}$ =0.01

$K_m^{nadh_{mito}} = 0.0054$ [21] 0.0052

3-hydroxyacyl-coa dehydrogenase (lc6)

$$v_{3hdh-lc6} = V_{max}^{3hdh-lc6} \cdot \left(\frac{lc6coa_{mito} \cdot nad_{mito} - {}^{1}\!/\!{}_{K_{eq}^{3hdh-lc6}} \cdot kc6coa_{mito} \cdot nadh_{mito}}{\left(1 + \frac{lc6coa_{mito}}{K_m^{lc6coa_{mito}}}\right) \cdot \left(1 + \frac{nad_{mito}}{K_m^{nad_{mito}}}\right) + \left(1 + \frac{kc6coa_{mito}}{K_m^{kc6coa_{mito}}}\right) \cdot \left(1 + \frac{nadh_{mito}}{K_m^{nadh_{mito}}}\right) - 1} \right)$$

$V_{max}^{3hdh-lc6}$ for numerical value see Supplementary Table 1

$K_{eq}^{3hdh-lc6} = \frac{1}{8 \cdot 10^{-4}}$ [22]

$K_m^{lc6coa_{mito}}$ =0.0286 [21]

$K_m^{nad_{mito}}$ =0.0585 [19]

$K_m^{kc6coa_{mito}}$ =0.0057 [23]

$K_m^{nadh_{mito}} = 0.0054$ [21]

3-hydroxyacyl-coa dehydrogenase (lc8)

$$v_{3hdh-lc8} = V_{max}^{3hdh-lc8} \cdot \left(\frac{lc8coa_{mito} \cdot nad_{mito} - {}^{1}\!/\!{}_{K_{eq}^{3hdh-lc8}} \cdot kc8coa_{mito} \cdot nadh_{mito}}{\left(1 + \frac{lc8coa_{mito}}{K_m^{lc8coa_{mito}}}\right) \cdot \left(1 + \frac{nad_{mito}}{K_m^{nad_{mito}}}\right) + \left(1 + \frac{kc8coa_{mito}}{K_m^{kc8coa_{mito}}}\right) \cdot \left(1 + \frac{nadh_{mito}}{K_m^{nadh_{mito}}}\right) - 1} \right)$$

$V_{max}^{3hdh-lc8}$ for numerical value see Supplementary Table 1

$K_{eq}^{3hdh-lc8} = \frac{1}{10^{-3}}$

$K_m^{lc8coa_{mito}}$ =0.0163 [21]

$K_m^{nad_{mito}}$ =0.0585 [19]

$K_m^{kc8coa_{mito}} = 0.0031$ [23]

$K_m^{nadh_{mito}}$ =0.0054 [21]

3-hydroxyacyl-coa dehydrogenase (lc10)

$$v_{3hdh-lc10} = V_{max}^{3hdh-lc10} \cdot \left(\frac{lc10coa_{mito} \cdot nad_{mito} - {}^{1}\!/\!{}_{K_{eq}^{3hdh-lc10}} \cdot kc10coa_{mito} \cdot nadh_{mito}}{\left(1 + \frac{lc10coa_{mito}}{K_m^{lc10coa_{mito}}}\right) \cdot \left(1 + \frac{nad_{mito}}{K_m^{nad_{mito}}}\right) + \left(1 + \frac{kc10coa_{mito}}{K_m^{kc10coa_{mito}}}\right) \cdot \left(1 + \frac{nadh_{mito}}{K_m^{nadh_{mito}}}\right) - 1} \right)$$

$V_{max}^{3hdh-lc10}$ for numerical value see Supplementary Table 1

$K_{eq}^{3hdh-lc10} = \frac{1}{10^{-3}}$

$K_m^{lc10coa_{mito}} = 0.0019$ [23]

$K_m^{nad_{mito}}$ =0.0585 [21]

$K_m^{kc10_{coa_{mito}}} = 0.0018$ [23]

$K_m^{nadh_{mito}}$ =0.0054 [21]

3-hydroxyacyl-coa dehydrogenase (lc12)

$$v_{3hdh-lc12} = V_{max}^{3hdh-lc12} \cdot \left(\frac{lc12coa_{mito} \cdot nad_{mito} - {}^{1}\!/\!{}_{K_{eq}^{3hdh-lc12}} \cdot kc12coa_{mito} \cdot nadh_{mito}}{\left(1 + \frac{lc12coa_{mito}}{K_m^{lc12coa_{mito}}}\right) \cdot \left(1 + \frac{nad_{mito}}{K_m^{nad_{mito}}}\right) + \left(1 + \frac{kc12coa_{mito}}{K_m^{kc12coa_{mito}}}\right) \cdot \left(1 + \frac{nadh_{mito}}{K_m^{nadh_{mito}}}\right) - 1} \right)$$

$V_{max}^{3hdh-lc12}$ for numerical value see Supplementary Table 1

$K_{eq}^{3hdh-lc12} = \frac{1}{10^{-3}}$

$K_m^{lc12coa_{mito}} = 0.0018$ [23]

$K_m^{nad_{mito}}$ =0.0585 [21]

$K_m^{kc12coa_{mito}} = 0.0018$ [23]

$K_m^{nadh_{mito}}$ =0.0054 [21]

3-hydroxyacyl-coa dehydrogenase (lc14)

$$v_{3hdh-lc14} = V_{max}^{3hdh-lc14} \cdot \left(\frac{lc14coa_{mito} \cdot nad_{mito} - {}^{1}\!/\!{}_{K_{eq}^{3hdh-lc14}} \cdot kc14coa_{mito} \cdot nadh_{mito}}{\left(1 + \frac{lc14coa_{mito}}{K_m^{lc14coa_{mito}}}\right) \cdot \left(1 + \frac{nad_{mito}}{K_m^{nad_{mito}}}\right) + \left(1 + \frac{kc14coa_{mito}}{K_m^{kc14coa_{mito}}}\right) \cdot \left(1 + \frac{nadh_{mito}}{K_m^{nadh_{mito}}}\right) - 1} \right)$$

$V_{max}^{3hdh-lc14}$ for numerical value see Supplementary Table 1

$K_{eq}^{3hdh-lc14} = \frac{1}{10^{-3}}$

$K_m^{lc14coa_{mito}}$ =0.0015 [23]

$K_m^{nad_{mito}}$ =0.0585 [21]

$K_m^{kc14coa_{mito}}$ =0.0013 [23]

$K_m^{nadh_{mito}}$ =0.0054 [21]

3-hydroxyacyl-coa dehydrogenase (lc16)

$$v_{3hdh-lc16} = V_{max}^{3hdh-lc16} \cdot \left(\frac{lc16coa_{mito} \cdot nad_{mito} - {}^{1}\!/\!{}_{K_{eq}^{3hdh-lc16}} \cdot kc16coa_{mito} \cdot nadh_{mito}}{\left(1 + \frac{lc16coa_{mito}}{K_m^{lc16coa_{mito}}}\right) \cdot \left(1 + \frac{nad_{mito}}{K_m^{nad_{mito}}}\right) + \left(1 + \frac{kc16coa_{mito}}{K_m^{kc16coa_{mito}}}\right) \cdot \left(1 + \frac{nadh_{mito}}{K_m^{nadh_{mito}}}\right) - 1} \right)$$

$V_{max}^{3hdh-lc16}$ for numerical value see Supplementary Table 1

$K_{eq}^{3hdh-lc16} = \frac{1}{10^{-3}}$

$K_m^{lc16_{coa_{mito}}}$ =0.0015 [23]

$K_m^{nad_{mito}}$ =0.0585 [21]

$K_m^{kc16_{coa_{mito}}}$ =0.0013 [23]

$K_m^{nadh_{mito}} = 0.0054$ [21]

3-ketoacyl-coa thiolase (kc4)

$$v_{3kt}^{kc4coa} = V_{max}^{3kt-kc4_{coa}} \cdot \left(\frac{coa_{mito} \cdot kc4coa_{mito} - {}^{1}\!/_{K_{eq}^{3kt}} \cdot acoa_{mito}^2}{\left(1 + \frac{coa_{mito}}{K_m^{coa_{mito}}}\right) \cdot \left(1 + \frac{kc4coa_{mito}}{K_m^{kc4coa_{mito}}}\right) + \left(1 + \frac{acoa_{mito}}{K_m^{acoa_{mito}}}\right) \quad - 1} \right)$$

$V_{max}^{3kt-kc4}$ for numerical value see Supplementary Table 1

$K_{eq}^{3kt} = 2500$ [24]

$K_m^{coa_{mito}} = \ 0.0022$ [25]

$$K_m^{kc4coa_{mito}} = K_0^{kc4coa_{mito}} \cdot \left(1 + \frac{acoa_{mito}}{K_i^{acao_{mito}}}\right)$$

$K_i^{acoa_{mito}}$ =0.24 [26]

$K_0^{kc4coa_{mito}}$ =0.0114 [26]

$$K_m^{acoa_{mito}} = K_0^{acoa_{mito}} \cdot \left(1 + \frac{kc4coa_{mito}}{K_i^{kc4coa_{mito}}}\right)$$

$K_0^{acoa_{mito}}$ =0.71 [26]

$K_i^{kc4coa_{mito}}$ =0.0022 [26]

3-ketoacyl-coa thiolase (kc5)

$$v_{3kt}^{kc5coa} = V_{max}^{3kt-c5coa} \cdot \left(\frac{kc5coa_{mito} \cdot coa_{mito} \ - {}^{1}\!/_{K_{eq}^{3kt}} \cdot acoa_{mito} \cdot propcoa_{mito}}{\left(coa_{mito} + K_m^{coa_{mito}}\right) \cdot \left(kc5coa_{mito} + K_m^{kc5coa_{mito}}\right)} \right)$$

$V_{max}^{3kt-kc5}$ for numerical value see Supplementary Table 1

$K_{eq}^{3kt} = 2500$ [24]

$K_m^{coa_{mito}} = 0.0189$ [25]

$K_m^{kc5coa_{mito}} = 0.01$

3-ketoacyl-coa thiolase (kc6)

$$v_{3kt}^{kc6coa} = V_{max}^{3kt-c6_{coa}} \cdot \left(\frac{kc6coa_{mito} \cdot coa_{mito} - 1/K_{eq}^{3kt} \cdot acoa_{mito} \cdot c4coa_{mito}}{\left(coa_{mito} + K_m^{coa_{mito}}\right) \cdot \left(kc6coa_{mito} + K_m^{kc6coa_{mito}}\right)} \right)$$

$V_{max}^{3kt-kc6}$ for numerical value see Supplementary Table 1

$K_{eq}^{3kt} = 2500$ [24]

$K_m^{coa_{mito}} = 0.0189$ [25]

$K_m^{kc6coa_{mito}} = 0.0067$ [25]

3-ketoacyl-coa thiolase (kc8)

$$v_{3kt}^{kc8coa} = V_{max}^{3kt-kc8} \cdot \left(\frac{kc8coa_{mito} \cdot coa_{mito} - 1/K_{eq}^{3kt} \cdot acoa_{mito} \cdot c6coa_{mito}}{\left(coa_{mito} + K_m^{coa_{mito}}\right) \cdot \left(kc8coa_{mito} + K_m^{kc8coa_{mito}}\right)} \right)$$

$V_{max}^{3kt-kc8}$ for numerical value see Supplementary Table 1

$K_{eq}^{3kt} = 2500$ [24]

$K_m^{coa_{mito}} = 0.0355$ [25]

$K_m^{kc8coa_{mito}} = 0.0032$ [25]

3-ketoacyl-coa thiolase (kc10)

$$v_{3kt}^{kc10coa} = V_{max}^{3kt-kc10} \cdot \left(\frac{kc10coa_{mito} \cdot coa_{mito} - 1/K_{eq}^{3kt} \cdot acoa_{mito} \cdot c8coa_{mito}}{\left(coa_{mito} + K_m^{coa_{mito}}\right) \cdot \left(kc10coa_{mito} + K_m^{kc10coa_{mito}}\right)} \right)$$

$V_{max}^{3kt-kc10}$ for numerical value see Supplementary Table 1

$K_{eq}^{3kt} = 2500$ [24]

$K_m^{coa_{mito}} = 0.0357$ [25]

$K_m^{kc10coa_{mito}}$ =0.0021 [25]

3-ketoacyl-coa thiolase (kc12)

$$v_{3kt}^{kc12coa} = V_{max}^{3kt-kc12} \cdot \left(\frac{kc12coa_{mito} \cdot coa_{mito} - {}^{1}\!/\!_{K_{eq}^{3kt}} \cdot acoa_{mito} \cdot c10coa_{mito}}{\left(coa_{mito} + K_m^{coa_{mito}}\right) \cdot \left(kc12coa_{mito} + K_m^{kc12coa_{mito}}\right)} \right)$$

$V_{max}^{3kt-kc12}$ for numerical value see Supplementary Table 1

$K_{eq}^{3kt} = 2500$ [24]

$K_m^{coa_{mito}}$ =0.0384 [25]

$K_m^{kc12coa_{mito}}$ =0.0013 [25]

3-ketoacyl-coa thiolase (kc14)

$$v_{3kt}^{kc14coa} = V_{max}^{3kt-kc14} \cdot \left(\frac{kc14coa_{mito} \cdot coa_{mito} - {}^{1}\!/\!_{K_{eq}^{3kt}} \cdot acoa_{mito} \cdot c12coa_{mito}}{\left(coa_{mito} + K_m^{coa_{mito}}\right) \cdot \left(kc14coa_{mito} + K_m^{kc14coa_{mito}}\right)} \right)$$

$V_{max}^{3kt-kc14}$ for numerical value see Supplementary Table 1

$K_{eq}^{3kt} = 2500$ [24]

$K_m^{coa_{mito}} = 0.032$

$K_m^{kc14coa_{mito}} = 0.0011$

3-ketoacyl-coa thiolase (kc16)

$$v_{3kt}^{kc16coa} = V_{max}^{3kt-kc16} \cdot \left(\frac{kc16coa_{mito} \cdot coa_{mito} - {}^{1}\!/\!_{K_{eq}^{3kt}} \cdot acoa_{mito} \cdot c14coa_{mito}}{\left(coa_{mito} + K_m^{coa_{mito}}\right) \cdot \left(kc16coa_{mito} + K_m^{kc16coa_{mito}}\right)} \right)$$

$V_{max}^{3kt-kc16}$ for numerical value see Supplementary Table 1

$K_{eq}^{3kt} = 2500$ [24]

$K_m^{coa_{mito}} = 0.0286$ [25]

$K_m^{kc16coa_{mito}} = 0.0011$ [25]

Propionyl-coa carboxylase

$$v_{pcc} = V_{max}^{ppc} \cdot \left(\frac{propcoa_{mito}}{propcoa_{mito} + K_m^{propcoa_{mito}}}\right) \cdot \left(\frac{atp_{mito}}{atp_{mito} + K_m^{atp_{mito}}}\right) \cdot \left(\frac{hco3_{mito}}{hco3_{mito} + K_m^{hco3_{mito}}}\right)$$

V_{max}^{ppc} for numerical value see Supplementary Table 1

$K_m^{propcoa_{mito}} = 0.01$ [27]

$K_m^{atp_{mito}} = 1$ [27]

$K_m^{hco3_{mito}} = 2$ [27]

Methylmalonyl-coa racemase

$$v_{mmrm} = V_{max}^{mmrm} \cdot \left(dmemcoa_{mito} - {}^{1}/_{K_{eq}^{mmrm}} \cdot lmemcoa_{mito}\right)$$

V_{max}^{mmrm} for numerical value see Supplementary Table 1

$K_{eq}^{mmrm} = 1$ [28]

Methylmalonyl-coa mutase

$$v_{mmm} = V_{max}^{mmm} \cdot \left(lmemcoa_{mito} - {}^{1}/_{K_{eq}^{mmm}} \cdot succoa_{mito}\right)$$

V_{max}^{mmm} for numerical value see Supplementary Table 1

$K_{eq}^{mmm} = 23$ [29]

ETF-FAD

$$v_{ETF-FAD} = V_{max}^{ETF-FAD} \cdot \left(etffadh2_{mito} \cdot etfq_{mito} - {}^{1}/_{K_{eq}^{ETF-FAD}} \cdot etfqh2_{mito} \cdot etffad_{mito}\right)$$

$V_{max}^{ETF-FAD}$ for numerical value see Supplementary Table 1

$$K_{eq}^{ETF-FAD} = \exp\left(\frac{\left(-n \cdot E_0^{etffad_{mito}/etffadh2_{mito}} - n \cdot E_0^{etfqh2_{mito}/etfq_{mito}}\right) \cdot F}{R \cdot T}\right)$$

$E_0^{etfqh2_{mito}/etfq_{mito}} = -25\ mV$ [30]

$E_0^{etffad_{mito}/etffadh2_{mito}} = -23\ mV$ [31]

$n = 2$

ETF-QO

$$v_{ETF-QO} = V_{max}^{ETF-QO} \cdot \left(etfqh2_{mito} \cdot q_{mm} - {}^{1}\!/\!_{K_{eq}^{ETF-QO}} \cdot etfq_{mito} \cdot qh_{2_{mm}} \right)$$

V_{max}^{ETF-QO} for numerical value see Supplementary Table 1

$$K_{eq}^{ETF-QO} = \exp\left(\frac{\left(n \cdot E_0^{q_{mm}/qh2_{mm}} + n \cdot E_0^{etfqh2_{mito}/etfq_{mito}}\right) \cdot F}{R \cdot T} \right)$$

$E_0^{q_{mm}/qh2_{mm}} = 87$ mV [32]

$E_0^{etfqh2_{mito}/etfq_{mito}} = -25\ mV$ [30]

$n = 2$

Citric acid cycle

Pyruvate dehydrogenase complex

$$v_{pdhc} = \gamma_{pdhc} \cdot v_{pdhc-np}$$

$$v_{pdhc-np} = V_{max}^{pdhc-np} \cdot \left(\frac{pyr_{mito}}{pyr_{mito} + K_{m-pyr}^{pdhc-np}} \right) \cdot \left(\frac{nad_{mito}}{nad_{mito} + K_{m-nad}^{pdhc-np}} \right) \cdot \left(\frac{coa_{mito}}{coa_{mito} + K_{m-coa}^{pdhc-np}} \right)$$

$V_{max}^{pdhc-np}$ for numerical value see Supplementary Table 1

$K_{m-pyr}^{pdhc-np} = 0.03$ [33]

$$K_{m-nad}^{pdhc-np} = K_0^{nad} \cdot \left(1 + \frac{nadh_{mito}}{K_i^{nadh_{mito}}} \right)$$

$K_0^{nad} = 0.064$ [34]

$K_i^{nadh_{mito}} = 0.3$ [34]

$$K_{m-coa}^{pdhc-np} = K_0^{coa} \cdot \left(1 + \frac{acoa_{mito}}{K_i^{acoa_{mito}}} \right)$$

$K_0^{coa} = 0.005$ [33]

$K_i^{acoa_{mito}}$ =0.003 [33]

$\gamma_{pdhc} = \gamma_{pdhc}^{acoa}$

$$\gamma_{pdhc}^{acoa} = 1 - \frac{\frac{acoa_{mito}}{coa_{mito}}}{\frac{acoa_{mito}}{coa_{mito}} + K_i^{\left(\frac{acoa_{mito}}{coa_{mito}}\right)}}$$ [35]

$K_i^{\left(\frac{acoa_{mito}}{coa_{mito}}\right)} = 0.053\ [dimensionless]$ [35]

Citrate synthase

$$v_{cs} = V_{max}^{cs} \cdot \left(\frac{oaa_{mito}}{oaa_{mito} + K_m^{oaa_{mito}}}\right) \cdot \left(\frac{acoa_{mito}}{acoa_{mito} + K_m^{acoa_{mito}}}\right)$$

$$V_{max}^{cs} = \frac{V_0^{cs}}{\left(1 + \frac{c16coa_{mito}}{K_i^{c16coa_{mito}}}\right)}\left(1 - \frac{cit_{mito}}{cit_{mito} + K_i^{cit_{mito}}}\right)$$

V_0^{cs} for numerical value see Supplementary Table 1

$K_i^{c16coa_{mito}} = 0.0042$ [36]

$K_m^{oaa_{mito}}$ =0.002 [37]

$K_m^{acoa_{mito}}$ =0.016 [37]

$K_i^{cit_{mito}} = 1.6$ [38]

Aconitase

$$v_{ac} = V_{max}^{ac} \cdot \left(\frac{cit_{mito} - {}^{1}/_{K_{eq}^{ac}} \cdot isocit_{mito}}{1 + \frac{cit_{mito}}{K_m^{cit_{mito}}} + \frac{isocit_{mito}}{K_m^{isocit_{mito}}}}\right)$$

V_{max}^{ac} for numerical value see Supplementary Table 1

$K_{eq}^{ac} = 0.1$ [39]

$K_m^{cit_{mito}}$ =0.48 [40]

$K_m^{isocit_{mito}}$ =0.12 [40]

NAD-dependent isocitrate dehydrogenase

$$v_{idh} = V_{max}^{idh} \cdot \left(\frac{isocit_{mito}}{isocit_{mito} + K_m^{isocit_{mito}}}\right) \cdot \left(\frac{nad_{mito}}{nad_{mito} + K_m^{nad_{mito}}}\right)$$

V_{max}^{idh} for numerical value see Supplementary Table 1

$$K_m^{isocit_{mito}} = K_0^{isocit_{mito}} \cdot \left(1 - n_{adp_{mito}} \frac{adp_{mito}}{adp_{mito} + K_a^{adp_{mito}}}\right)$$

$K_0^{isocit_{mito}}$ =0.32 [41]

$n_{adp_{mito}} = 0.67$ [41]

$K_a^{adp_{mito}}$ =0.1 [41]

$$K_m^{nad_{mito}} = K_0^{nad_{mito}} \cdot \left(1 + \frac{nadh_{mito}}{K_i^{nadh_{mito}}}\right)$$

$K_0^{nad_{mito}}$ =0.06 [41]

$K_i^{nadh_{mito}}$ =0.02 [41]

α-ketogluterate dehydrogenase

$$v_{kgdhc} = V_{mx}^{kgdhc} \cdot \left(\frac{akg_{mito}}{akg_{mito} + K_m^{akg_{mito}} \cdot \left(1 + \frac{nadh_{mito}}{K_{i2}^{nadh_{mito}}}\right)}\right) \cdot \left(\frac{nad_{mito}}{nad_{mito} + K_m^{nad} \cdot \left(1 + \frac{nadh_{mito}}{K_i^{nadh_{mito}}}\right)}\right) \cdot \left(\frac{coa_{mito}}{coa_{mito} + K_m^{coa_{mito}} \cdot \left(1 + \frac{succoa_{mito}}{K_i^{succoa_{mito}}}\right)}\right)$$

V_{mx}^{kgdhc} for numerical value see Supplementary Table 1

$K_{i2}^{nadh_{mito}}$ =0.0127 [42]

$K_m^{akg_{mito}}$ =0.6 [43]

$K_m^{nad_{mito}}$ =0.021 [42]

$K_i^{nadh_{mito}}$ =0.0045 [42]

$K_m^{coa_{mito}}$ = 0.0027 [42]

$K_i^{succoa_{mito}}$ =0.0069 [42]

Succinyl-Coa Synthetase [44]

$$v_{scs-atp} = V_{max}^{scs-atp} \cdot \left(\frac{succoa_{mito} \cdot adp_{mito} \cdot p_{mito} - 1 / K_{eq}^{scs-atp} \cdot suc_{mito} \cdot coa_{mito} \cdot atp_{mito}}{\left(1 + \frac{succoa_{mito}}{K_m^{succoa_{mito}}}\right) \cdot \left(1 + \frac{adp_{mito}}{K_m^{adp_{mito}}}\right) \cdot \left(1 + \frac{p_{mito}}{K_m^{p_{mito}}}\right) + \left(1 + \frac{suc_{mito}}{K_m^{suc_{mito}}}\right) \cdot \left(1 + \frac{coa_{mito}}{K_m^{coa_{mito}}}\right) \cdot \left(1 + \frac{atp_{mito}}{K_m^{atp_{mito}}}\right) - 1} \right)$$

$$V_{max}^{scs-atp} = V_0^{scs-atp} \cdot \left(\frac{p_{mito}^n}{p_{mito}^n + K_a^{p^n}} \right)$$

$V_0^{scs-atp}$ for numerical value see Supplementary Table 1

$K_a^{pn} = 2.3$ [45]

$n = 2.4$ [45]

$K_{eq}^{scs-atp} = 1/0.27$ [46]

$K_m^{succoa_{mito}} = 0.041$ [47]

$K_m^{adp_{mito}} = 0.25$ [47]

$K_m^{p_{mito}} = 0.72$ [47]

$K_m^{suc_{mito}} = 5.1$ [47]

$K_m^{coa_{mito}} = 0.032$ [47]

$K_m^{atp_{mito}} = 0.055$ [47]

$$v_{scs-gtp} = V_{max}^{scs-gtp} \cdot \left(\frac{succoa_{mito} \cdot gdp_{mito} \cdot p_{mito} - 1 / K_{eq}^{scs-atp} \cdot suc_{mito} \cdot coa_{mito} \cdot gtp_{mito}}{\left(1 + \frac{succoa_{mito}}{K_m^{succoa_{mito}}}\right) \cdot \left(1 + \frac{gdp_{mito}}{K_m^{gdp_{mito}}}\right) \cdot \left(1 + \frac{p_{mito}}{K_m^{p_{mito}}}\right) + \left(1 + \frac{suc_{mito}}{K_m^{suc_{mito}}}\right) \cdot \left(1 + \frac{coa_{mito}}{K_m^{coa_{mito}}}\right) \cdot \left(1 + \frac{gtp_{mito}}{K_m^{gtp_{mito}}}\right) - 1} \right)$$

$$V_{max}^{scs-gtp} = V_0^{scs-gtp} \cdot \left(\frac{p_{mito}^n}{p_{mito}^n + K_a^{p^n}} \right)$$

$V_0^{scs-gtp}$ for numerical value see Supplementary Table 1

$K_a^{p_{mito}} = 2.3$ [45]

$n = 2.4$ [45]

$K_{eq}^{scs-gtp} = 3.70$ [48]

$K_m^{succoa_{mito}} = 0.086$ [47]

$K_m^{gdp_{mito}}$ =0.007 [47]

$K_m^{p_{mito}}$ =2.26 [47]

$K_m^{suc_{mito}}$ =0.49 [47]

$K_m^{coa_{mito}}$ =0.036 [47]

$K_m^{gtp_{mito}}$ =0.036 [47]

Succinate dehydrogenase

$$v_{succdh} = V_{max}^{succdh} \cdot \left(\frac{suc_{mito} \cdot q_{mm} - {}^{1}\!/\!{}_{K_{eq}^{succdh}} \cdot fum_{mito} \cdot qh_{2_{mm}}}{\left(suc_{mito} + K_m^{suc_{mito}} \cdot \left(1 + \frac{mal_{mito}}{K_i^{mal_{mito}}} \right) \right) \cdot \left(q_{mm} + K_m^{q_{mm}} \right)} \right)$$

V_{max}^{succdh} for numerical value see Supplementary Table 1

$$K_{eq}^{succdh} = \exp\left(\frac{E_0^{fum_{mito}/suc_{mito}} - E_0^{q_{mm}/qh2_{mm}}}{R \cdot T} \cdot F \right)$$

$E_0^{fum_{mito}/suc_{mito}} - E_0^{q_{mm}/qh2_{mm}} = 25\ mV$

$K_m^{suc_{mito}} = 0.34$ [49]

$K_i^{mal_{mito}} = 2.2$ [50]

$K_m^{q_{mm}}$ =0.0005 [51]

Fumerase

$$v_{fum} = V_{max}^{fum} \cdot \left(\frac{fum_{mito} - {}^{1}\!/\!{}_{K_{eq}^{fum}} \cdot mal_{mito}}{1 + \frac{fum_{mito}}{K_m^{fum_{mito}}} + \frac{mal_{mito}}{K_m^{mal_{mito}}}} \right)$$

V_{max}^{fum} for numerical value see Supplementary Table 1

K_{eq}^{fum} =4.2 [52]

$K_m^{fum_{mito}}$ =0.333 [53]

$K_m^{mal_{mito}}$ =0.59 [53]

Malate dehydrogenase (mitochondrial)

$$v_{mdh_{mito}} = V_{max}^{mdh_{mito}} \cdot \left(\frac{mal_{mito} \cdot nad_{mito} - {}^{1}\!/\!{}_{K_{eq}^{mdh_{mito}}} \cdot oaa_{mito} \cdot nadh_{mito}}{\left(1 + \frac{mal_{mito}}{K_m^{mal_{mito}}}\right) \cdot \left(1 + \frac{nad_{mito}}{K_m^{nad_{mito}}}\right) + \left(1 + \frac{oaa_{mito}}{K_m^{oaa_{mito}}}\right) \cdot \left(1 + \frac{nadh_{mito}}{K_m^{nadh_{mito}}}\right) - 1} \right)$$

$V_{max}^{mdh_{mito}}$ for numerical value see Supplementary Table 1

$K_{eq}^{mdh_{mito}} = 1 \cdot 10^{-5} \cdot \left(\frac{h_{cyt}}{h_{mito}}\right)$ [54]

$K_m^{mal_{mito}}$ =0.33 [55]

$K_m^{nad_{mito}} = 0.06$ [56]

$K_m^{oaa_{mito}} = 0.017$ [56]

$K_m^{nadh_{mito}}$ =0.044 [56]

Transdehydrogenase [57]

$$v_{tdh} = V_{max}^{tdh} \cdot \left(nadh_{mito} \cdot nadp_{mito} - {}^{1}\!/\!{}_{K_{eq}^{tdh}} \cdot nad_{mito} \cdot nadph_{mito} \right)$$

$V_{max}^{tdh_{mito}}$ for numerical value see Supplementary Table 1

$$K_{eq}^{tdh_{mito}} = K_0^{tdh} \cdot \exp\left(-\frac{v_{mm} \cdot F}{R \cdot T}\right) \cdot \left(\frac{h_{cyt}}{h_{mito}}\right)$$

$K_0^{tdh} = 1.5$ [58]

Mitochondrial electrophysiology and ATP synthesis

chloride

$$I_{cl_{ed}} = P_{cl} \cdot A_m \cdot U \cdot F \cdot \left(\frac{cl_{cyt} - cl_{mito} \cdot exp(-U)}{1 - exp(-U)}\right)$$

$U = \frac{v_{mm} \cdot F}{R \cdot T}$

$P_{cl} = 4 \cdot 10^{-10}\ m/s$

sodium

$$I_{na}^{pump} = V_{max}^{Na-pump} \cdot \left(\frac{na_{cyt} \cdot h_{mito} - na_{mito} \cdot h_{cyt}}{1 + \frac{na_{cyt}}{K_m^{na}} + \frac{na_{mito}}{K_m^{na}}} \right)$$

$V_{max}^{Na-pump}$ for numerical value see Supplementary Table 1

$K_m^{na} = 26.5$ [59]

$$I_{na_{ed}} = P_{na} \cdot A_m \cdot U \cdot F \cdot \left(\frac{na_{cyt} - na_{mito} \cdot exp(U)}{exp(U) - 1} \right)$$

$U = \frac{v_{mm} \cdot F}{R \cdot T}$

$P_{na} = 8.2 \cdot 10^{-11} m/s$

$I_{na} = I_{na}^{pump} + I_{na_{ed}}$

potassium

$$I_K^{pump} = V_{max}^{K-pump} \cdot \left(k_{cyt} \cdot h_{mito} - k_{mito} \cdot h_{cyt} \right)$$

V_{max}^{K-pump} for numerical value see Supplementary Table 1

$$I_{k_{ed}} = P_k \cdot A_m \cdot U \cdot F \cdot \left(\frac{k_{cyt} - k_{mito} \cdot exp(U)}{exp(U) - 1} \right)$$

$U = \frac{v_{mm} \cdot F}{R \cdot T}$

$P_K = 2.132 \cdot 10^{-10} m/s$

$I_k = I_k^{pump} + I_{k_{ed}}$

F0F1 synthetase

$$v_{F0F1} = V_{max}^{F0F1} \cdot \left(\frac{adp_{mito} \cdot p_{mito} - {}^{1}\!/_{K_{eq}^{F0F1}} \cdot atp_{mito}}{\left(K_m^{adp_{mito}} + adp_{mito} \right) \cdot \left(K_m^{p_{mito}} + p_{mito} \right)} \right)$$

$$V_{max}^{F0F1} = V_{F0F1} \cdot \left(0.114 + 0.886 \, \frac{(|V_{mm}|)^n}{(|V_{mm}|)^n + \left(K_m^{V_{mm}} \right)^n} \right)$$ [60]

V_{F0F1} for numerical value see Supplementary Table 1

$n = 10$ [60]

$K_m^{V_{mm}} = 140$ mV [60]

$$K_{eq}^{F0F1} = exp\left(\left(\frac{-E_0^{ATP}}{R \cdot T} \right) - n_H \cdot \left(\frac{V_{mm} \cdot F}{R \cdot T} \right) \right) \cdot \left(\frac{H_{cyt}}{H_{mito}} \right)^{n_H} mM^{-1}$$

$n_H = 3$

$E_0^{ATP} = 30500$ J/mol

$K_m^{adp_{mito}}$ =0.025 [61]

$K_m^{p_{mito}} = 6.1$ [61]

ATP-ADP nucleotide exchanger [62]

$$v_{nex} = V_{max}^{nex} \cdot \left(\frac{1 - \frac{atp_{cyt} \cdot adp_{mito}}{adp_{cyt} \cdot atp_{mito}} \cdot exp\left(\frac{v_{mm} \cdot F}{R \cdot T}\right)}{1 + \frac{atp_{cyt}}{adp_{cyt}} \cdot exp\left(f \cdot \frac{v_{mm} \cdot F}{R \cdot T}\right) \cdot \left(1 + \frac{adp_{mito}}{atp_{mito}}\right)} \right)$$

V_{max}^{nex} for numerical value see Supplementary Table 1

f =0.2

Phosphate exchanger

$$v_{P-ex} = V_{max}^{P-ex} \cdot \left(\frac{p_{cyt} \cdot h_{cyt} - p_{mito} \cdot h_{mito}}{p_{cyt} + K_m^{p_{cyt}}} \right)$$

V_{max}^{P-ex} for numerical value see Supplementary Table 1

$K_m^{p_{cyt}} = 1.6$ [63]

Complex I

$$v_{cxI} = V_{max}^{cxI} \cdot \left(\frac{nadh_{mito} \cdot q_{mm} - {}^{1}\!/_{K_{eq}^{cxI}} \cdot nad_{mito} \cdot qh_{2_{mm}}}{\left(nadh_{mito} + K_m^{nadh_{mito}}\right) \cdot \left(q_{mm} + K_m^{q_{mm}}\right)} \right)$$

V_{max}^{cxI} for numerical value see Supplementary Table 1

$$K_{eq}^{cxI} = \exp\left(\frac{\left(n \cdot E_0^{nadh/nad} + n \cdot E_0^{Q/QH_2} + n_H \cdot V_{mm}\right) \cdot F}{R \cdot T} \right) \cdot \left(\frac{h_{mito}}{h_{cyt}}\right)^{n_H}$$

$n = 2$

$E_0^{nadh/nad} = 320$ mV [64]

$E_0^{Q/QH_2} = 87mV$ [32]

$n_H = 4$

$K_m^{nadh_{mito}} = 0.0017$ [65]

$K_m^{q_{mm}} = 0.013$ [65]

Complex II : see succinate dehydrogenase

Complex III

$$v_{cxIII} = V_{max}^{cxIII} \cdot \left(\frac{qh2_{mm} \cdot cytc_{ox_{mm}} - {}^{1}\!/\!_{K_{eq}^{cxIII}} \cdot q_{mm} \cdot cytc_{red_{mm}}}{\left(qh2_{mm} + K_m^{qh2_{mm}}\right) \cdot \left(cytc_{ox_{mm}} + K_m^{cytc_{ox}}\right)^2} \right)$$

V_{max}^{cxIII} for numerical value see Supplementary Table 1

$$K_{eq}^{cxIII} = \exp\left(\frac{\left(-n \cdot E_0^{Q/QH_2} + n \cdot E_0^{cytC_{ox}/cytC_{red}} + n \cdot v_{mm}\right) \cdot F}{R \cdot T} \right) \cdot \left(\frac{h_{mito}}{h_\emptyset}\right)^{n_{h_{mito}}} \cdot \left(\frac{h_\emptyset}{h_{cyt}}\right)^{n_{h_{cyt}}}$$

$n = 2$

$E_0^{cytC_{ox}/cytC_{red}} = 255\ mV$ [66]

$E_0^{Q/QH_2} = 87\ mV$ [32]

$n_{h_{mito}} = 2$

$n_{h_{cyt}} = 4$

$h_\emptyset = 10^{-4} mM \stackrel{\text{def}}{=} \text{pH } 7$

$K_m^{qh_{2mm}} = 0.013$ [67]

$K_m^{cytc_{ox}} = 0.014$ [67]

Complex IV

$$v_{cxIV} = V_{max}^{cxIV} \cdot \left(\frac{cytc_{red}}{cytc_{red} + K_m^{cytc_{red}}}\right) \cdot \left(\frac{O_2}{O_2 + K_m^{O_2}}\right)$$

V_{max}^{cxIV} for numerical value see Supplementary Table 1

$K_m^{cytc_{red}} = 0.007$ [68]

$K_m^{O_2} = 2\ mmHg$

Adenylate kinase

$$v_{ak_{cyt}} = V_{max}^{ak_{cyt}} \cdot \left(\frac{atp_{cyt} \cdot amp_{cyt} - {}^{1}\!/_{K_{eq}^{ak}} \cdot adp_{cyt} \cdot adp_{cyt}}{\left(1 + \frac{atp_{cyt}}{K_m^{atp_{cyt}}}\right) \cdot \left(1 + \frac{amp_{cyt}}{K_m^{amp_{cyt}}}\right) + \left(1 + \frac{adp_{cyt}}{K_m^{adp_{cyt}}}\right)^2 - 1} \right)$$

$V_{max}^{ak_{cyt}}$ for numerical value see Supplementary Table 1

$K_{eq}^{ak} = 1$ [69]

$K_m^{atp_{cyt}} = 0.125$ [70]

$K_m^{adp_{cyt}} = 0.184$ [70]

$K_m^{amp_{cyt}} = 0.125$ [70]

Pyrophosphatase

$$v_{ppase} = V_{max}^{ppase} \cdot \left(\frac{pp_{cyt}}{pp_{cyt} + K_m^{pp_{cyt}}} \right)$$

V_{max}^{ppase} for numerical value see Supplementary Table 1

$K_m^{pp_{cyt}} = 0.005$ [71]

ATP usage

$$v_{atp-usage} = V_{max}^{atp-usage} \cdot \left(\frac{atp_{cyt}}{atp_{cyt} + K_m^{atp_{cyt}}} \right)$$

$V_{max}^{atp-usage}$ for numerical value see Supplementary Table 1

$K_m^{atp_{cyt}} = 2$

O_2 diffusion

$$v_{O_{2\,diff}} = V_{max}^{O_2-diff} \cdot \left(o2_{ext} - o2_{cyt} \right)$$

$V_{max}^{O_2-diff}$ for numerical value see Supplementary Table 1

Proton fluxes

$$I_H^{pump} = 4 \cdot v_{cxI} + 2 \cdot v_{cxIII} + 4 \cdot v_{cxIV}$$

$$I_{H_{ed}} = P_H \cdot A_m \cdot U \cdot F \cdot \left(\frac{H_{cyt} - H_{mito} \cdot exp(U)}{exp(U) - 1}\right)$$

$$P_H = 2.46 \cdot 10^{-4} m/s$$

Mitochondrial membrane potential

$$v_{V_{mm}} = \frac{10^{-1}}{c_m \cdot A_m} \cdot \left(-I_{C_{ed}} + I_{K_{ed}} + I_{H_{ed}} + I_{Na_{ed}} + I_H^{pump} + v_{nex} + 3 \cdot v_{syn} + F \cdot 10 \cdot v_{pepT} \cdot Vol_{cyt}\right)$$

Glycolysis

Glut2 glucose transporter (Glut2)

$$v_{gluT2} = V_{max}^{gluT2} \cdot \frac{glc_{ext} - glc_{cyt}}{1 + \frac{glc_{ext}}{K_m^{glc_{ext}}} + \frac{glc_{cyt}}{K_m^{glc_{cyt}}}}$$

v_{gluT2} for numerical value see Supplementary Table 1

$K_m^{glc_{cyt}} = 17.3$ [72]

$K_m^{glc_{ext}} = 17.3$ [72]

$V_{max}^{gluT2} =$

Glucokinase

$$v_{Gk} = V_{max}^{Gk} \cdot \frac{atp_{cyt}}{atp_{cyt} + K_m^{atp_{cyt}}} \cdot \frac{(glc_{cyt})^n}{(glc_{cyt})^n + \left(K_m^{glc_{cyt}}\right)^n}$$

$$V_{max}^{Gk} = V_0^{gk} \cdot \frac{(glc_{cyt})^{n2}}{(glc_{cyt})^{n2} + \left(K_a^{glc_{cyt}}\right)^{n2}} \cdot \left(1 - f \cdot \frac{fru6p_{cyt}}{fru6p_{cyt} + K_i^{fru6p_{cyt}}}\right)$$

V_0^{gk} for numerical value see Supplementary Table 1

$n = 1.5$ [73]

$K_m^{glc_{cyt}} = K_0^{glc} \cdot \left(1 + \frac{c16coa_{cyt}}{K_i^{c16coa_{cyt}}}\right)$ [73]

$K_0^{glc} = 9$ [73]

$K_i^{c16coa_{cyt}} = 0.0075$ [74]

$K_m^{atp_{cyt}} = 0.55$ [75]

$K_i^{fru6p_{cyt}} = 0.005$ [76]

$f = 0.75$ [76]

$n2 = 3.7$ [77]

$K_a^{glc_{cyt}} = 15.9$ [77]

Hexokinase

$$v_{hk} = V_{max}^{hk} \cdot \left(\frac{glc_{cyt}}{glc_{cyt} + K_m^{glc_{cyt}}}\right) \cdot \left(\frac{atp_{cyt}}{atp_{cyt} + K_m^{atp_{cyt}}}\right)$$

V_{max}^{hk} for numerical value see Supplementary Table 1

$K_m^{glc_{cyt}} = 0.007$ [78]

$K_m^{atp_{cyt}} = 0.98$ [78]

Glucose-6-phosphate transport to ER

$$v_{glc6pT_{er}} = V_{max}^{glc6pT_{er}} \cdot \frac{(glc6p_{cyt} - glc6p_{er})}{1 + \frac{glc6p_{cyt}}{K_m^{glc6p_{cyt}}} + \frac{glc6p_{er}}{K_m^{glc6p_{er}}}}$$

$V_{max}^{glc6pT_{er}}$ for numerical value see Supplementary Table 1

$K_m^{glc6p_{er}} = 1.12$ [79]

$K_m^{glc6p_{cyt}} = 1.12$ [79]

D-Glucose-6-phosphate phosphatase

$$v_{glc6pp_{er}} = V_{max}^{glc6pp_{er}} \cdot \frac{glc6p_{er}}{glc6p_{er} + K_m^{glc6p_{er}}}$$

$K_m^{glc6p_{er}} = 1.84$ [80]

$V_{max}^{glc6pp_{er}}$ for numerical value see Supplementary Table 1

Glucose transport to ER

$$v_{glcT_{er}} = V_{max}^{glcT_{er}} \cdot \left(\frac{glc_{cyt} - glc_{er}}{1 + \frac{glc_{cyt}}{K_m^{glc_{cyt}}} + \frac{glc_{er}}{K_m^{glc_{er}}}} \right)$$

$K_m^{glc_{cyt}} = 1.37$ [81]

$K_m^{glc_{er}} = 1.22$ [81]

$V_{max}^{glcT_{er}}$ for numerical value see Supplementary Table 1

phosphate transport to ER

$$v_{pT_{er}} = V_{max}^{pT_{er}} \cdot (p_{cyt} - p_{er})$$

$V_{max}^{pT_{er}}$ for numerical value see Supplementary Table 1

D-Glucose-6-phosphate isomerase (Gpi)

$$v_{gpi} = V_{max}^{gpi} \cdot \frac{glc6p_{cyt} - \frac{fru6p_{cyt}}{K_{eq}^{gpi}}}{1 + \frac{glc6p_{cyt}}{k_m^{glc6p_{cyt}}} + \frac{fru6p_{cyt}}{k_m^{fru6p_{cyt}}}}$$

V_{max}^{gpi} for numerical value see Supplementary Table 1

$k_{eq}^{Gpi} =$ 0.3 [82]

$k_m^{glc6p_{cyt}} = 0.055$ [83]

$K_m^{fru6p_{cyt}} = 0.12$ [83]

Phosphofructokinase 2 (Pfk2):

$$v_{pfk2} = V_{max}^{pfk2} \cdot \left(\left(1 - \gamma^{Pfk2}\right) \cdot v_{pfk2}^{native} + \gamma^{pfk2} \cdot v_{pfk2}^{p} \right)$$

$$V_{max}^{pfk2} = V_0^{pfk2} \cdot \left(1 - \frac{fru26bp_{cyt}}{K_i^{fru26bp_{cyt}}}\right) \cdot \left(1 - n_0 \cdot \frac{pep_{cyt}}{pep_{cyt} + K_i^{pep_{cyt}}}\right)$$

V_0^{pfk2} for numerical value see Supplementary Table 1

$K_i^{pep_{cyt}} = 0.25$ [84]

$n_0 = 0.85$ [84]

$K_i^{fru26bp_{cyt}} = 0.1$

$$v_{pfk2}^{native} = \frac{fru6p_{cyt}^n}{fru6p_{cyt}^n + \left(K_m^{fru6p_{cyt}}\right)^n} \cdot \frac{atp_{cyt}}{atp_{cyt} + K_m^{atp_{cyt}}}$$

$K_m^{fru6p_{cyt}}$ =0.015 [85]

$n = 1.3$ [85]

$K_m^{atp_{cyt}} = 0.25$ [85]

$$v_{pfk2}^{p} = \frac{fru6p_{cyt}^n}{fru6p_{cyt}^n + \left(K_m^{fru6p_{cyt}}\right)^n} \cdot \frac{atp_{cyt}}{atp_{cyt} + K_m^{atp_{cyt}}}$$

$K_m^{fru6p_{cyt}} = 0.05$ [85]

$n = 2$ [85]

$K_m^{atp_{cyt}} = 0.5\ mM$ [85]

Fructose-2,6-bisphosphatase (FBP2)

$$v_{fbp2} = V_{max}^{fbp2} * \left(\left(1 - \gamma^{fbp2}\right) \cdot v_{fbp2}^{native} + \gamma^{fbp2} \cdot v_{fbp2}^{p}\right)$$

V_{max}^{fbp2} for numerical value see Supplementary Table 1

$$v_{fbp2}^{native} = \frac{fru26bp_{cyt}}{fru26bp_{cyt} + K_m^{fru26bp_{cyt}}} / \left(1 + \frac{fru6p_{cyt}}{K_i^{fru6p_{cyt}}}\right)$$

$K_m^{fru26bp_{cyt}} = 0.01$ [86]

$K_i^{fru6p_{cyt}} = 0.0035$ [85]

$$v_{fbp2}^{p} = f \cdot \frac{fru26bp_{cyt}}{fru26bp_{cyt} + K_m^{fru26bp_{cyt}}} / \left(1 + \frac{fru6p_{cyt}}{K_i^{fru6p_{cyt}}}\right)$$

$f = 0.33$ [86]

$K_m^{fru26p_{cyt}} = 0.0005$ [85]

$K_i^{fru6p_{cyt}} = 0.01$ [85]

Phosphofructokinase 1 (Pfk1):

$$v_{pfk1} = v_{max}^{pfk1} \cdot \frac{atp_{cyt}}{atp_{cyt} + K_m^{atp_{cyt}}} \cdot \left(1 - \frac{atp_{cyt}^{n_i}}{atp_{cyt}^{n_i} + \left(K_i^{atp_{cyt}}\right)^{n_i}}\right) \cdot \frac{\left(fru6p_{cyt}\right)^{n_{fru6p_{cyt}}}}{\left(fru6p_{cyt}\right)^{n_{fru6p_{cyt}}} + \left(K_m^{fru6p_{cyt}}\right)^{n_{fru6p_{cyt}}}}$$

v_{max}^{pfk1} for numerical value see Supplementary Table 1

$$K_m^{atp_{cyt}} = K_0^{atp_{cyt}} \cdot \left(1 - \mathrm{k_{fru26bp}} \frac{fru26bp_{cyt}}{fru26bp_{cyt} + K_a^{fru26bp_{cyt}}}\right)$$

$K_0^{atp_{cyt}}$ =0.2 [87 88]

$\mathrm{k_{fru26bp}} = 0.8$ [87 88]

$K_a^{fru26bp_{cyt}} = 0.0027$ [87 88]

$$K_i^{atp_{cyt}} = K_{i0}^{atp_{cyt}} \cdot \left(1 + f_{fru26bp} \frac{fru26bp_{cyt}}{fru26bp_{cyt} + K_{a2}^{fru26bp_{cyt}}}\right)$$

$K_{i0}^{atp_{cyt}} = 0.7$ [87 88]

$f_{fru26bp} = 9$ [87 88]

$K_{a2}^{fru26bp_{cyt}} = 0.54$ [87 88]

n_i =4 [87 88]

$$K_m^{fru6p_{cyt}} = K_0^{fru6p} \cdot \left(1 + \frac{atp_{cyt}}{K_i^{atp_{cyt}}}\right) \cdot \left(1 + \frac{cit_{cyt}}{K_i^{cit_{cyt}}}\right) \cdot \left(1 - f_{amp} \frac{amp_{cyt}^{n_{amp}}}{amp_{cyt}^{n_{amp}} + \left(K_a^{amp_{cyt}}\right)^{n_{amp}}}\right)$$
$$\cdot \left(1 - f_p \frac{p_{cyt}}{p_{cyt} + K_a^{p_{cyt}}}\right) \cdot \left(1 - f_{fru26bp} \frac{fru26bp_{cyt}^{n_{fru26bp}}}{fru26bp_{cyt}^{n_{fru26p}} + \left(K_a^{fru26bp}\right)^{n_{fru26p}}}\right)$$
$$\cdot \left(1 - f_{fru16p} \frac{fru16bp_{cyt}^{n_{fru16bp}}}{fru16bp_{cyt}^{n_{fru16bp}} + \left(K_a^{fru16bp}\right)^{n_{fru16bp}}}\right)$$

$K_0^{fru6p} = 1.14$ [89]

$K_i^{atp_{cyt}} = 0.6$ [89]

$K_i^{cit_{cyt}} = 3.27$ [89]

$f_{amp} = 0.77$ [89]

$K_a^{amp} = 0.1$ [89]

$n_{amp} = 1.84$ [89]

$f_p = 0.85$ [89]

$K_a^{p_{cyt}} = 0.69$ [89]

$f_{fru26bp} = 0.92$ [87]

$K_a^{fru16bp} = 0.0045$ [87]

$n_{fru16bp} = 1.2$ [87]

$f_{fru16bp} = 0.78$ [89]

$K_a^{fru16bp} = 0.03$ [89]

$n_{fru16bp} = 2.21$ [89]

$$n^{fru6p} = \left(n_0 + \frac{atp_{cyt}^{n_{atp}}}{atp_{cyt}^{n_{atp}} + \left(K_i^{atp_{cyt}}\right)^{n_{atp}}}\right) \cdot \left(1 - f_{amp}\frac{amp_{cyt}^{n_{amp}}}{amp_{cyt}^{n_{amp}} + \left(K_a^{amp_{cyt}}\right)^{n_{amp}}}\right)$$
$$\cdot \left(1 + f_{cit}\frac{cit_{cyt}^{n_{cit}}}{cit_{cyt}^{n_{cit}} + \left(K_i^{cit_{cyt}}\right)^{n_{cit}}}\right) \cdot \left(1 - f_p\frac{p_{cyt}^{n_p}}{p_{cyt}^{n_p} + \left(K_a^{p_{cyt}}\right)^{n_p}}\right) \cdot$$
$$\cdot \left(1 - f_{fru26bp}\frac{fru26bp_{cyt}^{n_{fru26bp}}}{fru26bp_{cyt}^{n_{fru26bp}} + \left(K_a^{fru26bp_{cyt}}\right)^{n_{fru26bp}}}\right)$$
$$\cdot \left(1 - f_{fru16bp}\frac{fru16bp_{cyt}^{n_{fru16bp}}}{fru16bp_{cyt}^{n_{fru16bp}} + \left(K_a^{fru16bp_{cyt}}\right)^{n_{fru16bp}}}\right)$$

$n_0 = 3.67$ [89]

$K_i^{atp_{cyt}} = 0.13$ [89]

$n_{atp} = 1.59$ [89]

$f_{amp} = 0.4$ [89]

$K_a^{amp_{cyt}} = 0.086$ [89]

$n_{amp} = 2.22$ [89]

$f_{cit} = 0.1$ [89]

$K_i^{cit_{cyt}} = 0.18$ [89]

$n_{cit} = 4$ [89]

$f_p = 0.28$ [89]

$K_a^{p_{cyt}} = 0.53$ [89]

$n_p = 4$ [89]

$f_{fru26bp} = 0.37$ [87]

$K_a^{fru26bp_{cyt}} = 0.0021$ [87]

$n_{fru26bp} = 4$ [87]

$f_{fru16bp} = 0.68$ [89]

$K_a^{fru16bp_{cyt}} = 0.022$ [89]

$n_{fru26bp} = 1.67$ [89]

Fructose-1,6-bisphosphatase (Fbp1)

$$v_{fbp1} = V_{max}^{fbp1} \cdot \left(\left(1 - \gamma^{fbp1}\right) \cdot v_{fbp1}^{native} + \gamma^{fbp1} \cdot v_{fbp1}^{p} \right)$$

V_{max}^{fbp1} for numerical value see Supplementary Table 1

$$v_{fbp1}^{native} = \frac{fru16bp_{cyt}}{fru16bp_{cyt} + K_m^{fru16bp_{cyt}}} / \left(1 + \frac{fru26bp_{cyt}^{n}}{\left(K_i^{fru26bp_{cyt}}\right)^n} \right)$$

$K_m^{fru16bp_{cyt}} = 0.0029$ [90]

$K_i^{fru26p_{cyt}} = 0.00113$ [90]

$n = 1.26$ [90]

$$v_{fbp1}^{p} = \frac{fru16bp_{cyt}}{fru16bp_{cyt} + K_m^{fru16bp_{cyt}}} / \left(1 + \frac{fru26bp_{cyt}^{n}}{\left(K_i^{fru26bp_{cyt}}\right)^n} \right)$$

$K_m^{fru16bp_{cyt}} = 0.0019$ [90]

$K_i^{fru26bp_{cyt}} = 0.00113$ [90]

$n = 1.26$ [90]

Aldolase (Ald)

$$v_{ald} = v_{max}^{ald} \cdot \frac{fru16bp_{cyt} - \frac{1}{K_{eq}^{ald}} grap_{cyt} \cdot dhap_{cyt}}{\left(1 + \frac{fru16bp_{cyt}}{K_m^{fru16bp_{cyt}}}\right) + \left(1 + \frac{grap_{cyt}}{K_m^{grap_{cyt}}}\right) + \left(1 + \frac{dhap_{cyt}}{K_m^{dhap_{cyt}}}\right) - 1}$$

v_{max}^{ald} for numerical value see Supplementary Table 1

K_{eq}^{ald} =0.099 [91]

$K_m^{fru16bp_{cyt}} = 0.004$ [92]

$K_m^{grap_{cyt}} = 0.48$ [93]

$K_m^{dhap_{cyt}} = 0.38$ [93]

Triosephosphate isomerase (Tpi)

$$v_{tpi} = v_{max}^{tpi} \cdot \frac{dhap_{cyt} - \frac{grap_{cyt}}{K_{eq}^{tpi}}}{1 + \frac{dhap_{cyt}}{K_m^{dhap_{cyt}}} + \frac{grap_{cyt}}{K_m^{grap_{cyt}}}}$$

v_{max}^{tpi} for numerical value see Supplementary Table 1

$K_{eq}^{tpi} = 0.04545$ [91]

$K_m^{dhap_{cyt}} = 0.59$ [94]

$K_m^{grap_{cyt}} = 0.415$ [94]

D-Glyceraldehyde-3-phosphate:NAD$^+$ oxidoreductase (Gapdh)

$$v_{gapdh} = v_{max}^{Gapdh} * \frac{nad_{cyt} \cdot grap_{cyt} \cdot p_{cyt} - \frac{1}{K_{eq}^{gapdh}} \cdot bpg13_{cyt} \cdot nadh_{cyt}}{\left(1 + \frac{nad_{cyt}}{K_m^{nad_{cyt}}}\right) \cdot \left(1 + \frac{grap_{cyt}}{K_m^{grap_{cyt}}}\right) \cdot \left(1 + \frac{p_{cyt}}{K_m^{p_{cyt}}}\right) + \left(1 + \frac{nadh_{cyt}}{K_m^{nadh_{cyt}}}\right) \cdot \left(1 + \frac{bpg13_{cyt}}{K_m^{bpg13_{cyt}}}\right) - 1}$$

v_{max}^{Gapdh} for numerical value see Supplementary Table 1

$K_{eq}^{gapdh} = 10^{-4} mM^{-1}$ [95]

$K_m^{nad_{cyt}} = 0.010$ [96]

$K_m^{grap_{cyt}} = 0.035$ [96]

$K_m^{p_{cyt}} = 3.8$ [97]

$K_m^{nadh_{cyt}} = 0.006$ [97]

$K_m^{bpg13_{cyt}} = 0.01$ [96]

Phosphyglyceratekinase (Pgk)

$$v_{pgk} = v_{max}^{pgk} \cdot \frac{adp_{cyt} \cdot bpg13_{cyt} - \frac{1}{K_{eq}^{pgk}} \cdot atp_{cyt} \cdot pg3_{cyt}}{\left(1 + \frac{adp_{cyt}}{K_m^{adp_{cyt}}}\right) \cdot \left(1 + \frac{bpg13_{cyt}}{K_m^{bpg13_{cyt}}}\right) + \left(1 + \frac{atp_{cyt}}{K_m^{atp_{cyt}}}\right) \cdot \left(1 + \frac{pg3_{cyt}}{K_m^{pg3_{cyt}}}\right) - 1}$$

v_{max}^{pgk} for numerical value see Supplementary Table 1

$K_{eq}^{pgk} = 1830$ [98]

$K_m^{adp_{cyt}} = 0.35$ [99]

$K_m^{bpg13_{cyt}} = 0.0022$ [99]

$K_m^{atp_{cyt}} = 0.24$ [100]

$K_m^{pg3_{cyt}} = 1.65$ [100]

2-Phospho-D-glycerate 2,3 phosphomutase (Pgm)

$$v_{pgm} = v_{max}^{pgm} \cdot \frac{pg3_{cyt} - \frac{1}{K_{eq}^{pgm}} pg2_{cyt}}{pg3_{cyt} + K_m^{pg3_{cyt}} \cdot \left(1 + \frac{pg2_{cyt}}{K_m^{pg2}}\right)}$$

v_{max}^{pgm} for numerical value see Supplementary Table 1

$K_{eq}^{pgm} = 0.1725$ [101]

$K_m^{pg3_{cyt}} = 0.52$ [102]

$K_m^{pg2} = 0.24$ [102]

2-Phospho-D-glycerate hydrolase (Eno)

$$v_{eno} = v_{max}^{eno} \cdot \frac{pg2_{cyt} - \frac{1}{K_{eq}^{eno}} \cdot pep_{cyt}}{1 + \frac{pg2_{cyt}}{K_m^{pg2_{cyt}}} + \frac{pep_{cyt}}{K_m^{pep_{cyt}}}}$$

v_{max}^{eno} for numerical value see Supplementary Table 1

$K_{eq}^{eno} = 1.7$ [103]

$K_m^{pg2_{cyt}} = 0.14$ [104]

$K_m^{pep_{cyt}} = 0.31$ [104]

Pyruvate kinase (Pk): $\boldsymbol{Pep + ADP \leftrightarrow Pyr + ATP}$

$$v_{pk} = v_{max}^{Pk} \cdot \left((1-\gamma^{Pk}) \cdot v_{Pk}^{native} + \gamma^{Pk} \cdot v_{Pk}^{phospho}\right)$$

$$v_{Pk}^{native} = \frac{pep_{cyt}}{pep_{cyt} + K_m^{pep_{cyt}} \cdot \left(1 + \frac{atp_{cyt}}{K_i^{atp_{cyt}}}\right) \cdot \left(1 + \frac{ala_{cyt}}{K_i^{ala_{cyt}}}\right) \cdot \left(1 - \frac{fru16bp_{cyt}}{fru16bp_{cyt} + K_a^{fru16bp_{cyt}}}\right)} \cdot \frac{adp_{cyt}}{adp_{cyt} + K_m^{adp_{cyt}}}$$

v_{max}^{Pk} for numerical value see Supplementary Table 1

$K_m^{pep_{cyt}} = 1.6$ [105]

n =1 [106]

$K_i^{atp_{cyt}}$ =1 [106]

$K_i^{ala_{cyt}}$ =1.08 [107]

$K_a^{fru16bp_{cyt}} = 0.0078$ [108]

$K_m^{adp_{cyt}} = 0.25$ [109]

$$v_{Pk}^{phospho} = \frac{pep_{cyt}^n}{pep_{cyt}^n + \left(K_m^{pep_{cyt}} \cdot \left(1 + \frac{atp_{cyt}}{K_i^{atp_{cyt}}}\right) \cdot \left(1 + \frac{ala_{cyt}}{K_i^{ala_{cyt}}}\right) \cdot \left(1 - \frac{fru16bp_{cyt}}{fru16bp_{cyt} + K_a^{fru16bp_{cyt}}}\right)\right)^n} \cdot \frac{adp_{cyt}}{adp_{cyt} + K_m^{adp_{cyt}}}$$

$K_m^{pep_{cyt}}$ =2.5 [105]

n =2.9 [106]

$K_a^{fru16bp_{cyt}}$ =0.0095 [108]

$K_i^{atp_{cyt}} = 0.32$ [107]

$K_i^{ala_{cyt}}$ =0.52 [107]

$K_m^{adp_{cyt}}$ =0.33 [109]

Phosphoenolpyruvate carboxykinase (Pepck)

$$v_{pepck} = v_{max}^{pepck} \cdot \frac{oaa_{cyt} \cdot gtp_{cyt} - \frac{1}{K_{eq}^{pepck}} \cdot pep_{cyt} \cdot gdp_{cyt} \cdot hco3_{cyt}}{\left(1 + \frac{oaa_{cyt}}{K_m^{oaa_{cyt}}}\right) \cdot \left(1 + \frac{gtp_{cyt}}{K_m^{gtp_{cyt}}}\right) + \left(1 + \frac{pep_{cyt}}{K_m^{pep_{cyt}}}\right) \cdot \left(1 + \frac{gdp_{cyt}}{K_m^{gdp_{cyt}}}\right) \cdot \left(1 + \frac{hco3_{cyt}}{K_m^{hco3_{cyt}}}\right) - 1}$$

v_{max}^{pepck} for numerical value see Supplementary Table 1

$K_{eq}^{pepck} = 500mM$ [110]

$K_m^{oaa_{cyt}} = 0.024$ [111]

$K_m^{gtp_{cyt}} = 0.021$ [112]

$K_m^{pep_{cyt}} = 0.4$ [113]

$K_m^{gdp_{cyt}} = 0.02$ [114]

$K_m^{hco3_{cyt}} = 1.194$ [115]

mitochondrial phosphoenolpyruvate carboxykinase (Pepck-mito)

$$v_{pepck_{mito}} = v_{max}^{pepck_{mito}} \cdot \frac{oaa_{mito} \cdot gtp_{mito} - \frac{1}{K_{eq}^{pepck_{mito}}} \cdot pep_{mito} \cdot gdp_{mito} \cdot hco3_{mito}}{\left(1 + \frac{oaa_{mito}}{K_m^{oaa_{mito}}}\right) \cdot \left(1 + \frac{gtp_{mito}}{K_m^{gtp_{mito}}}\right) + \left(1 + \frac{pep_{mito}}{K_m^{pep_{mito}}}\right) \cdot \left(1 + \frac{gdp_{mito}}{K_m^{gdp_{mito}}}\right) \cdot \left(1 + \frac{hco3_{mito}}{K_m^{hco3_{mito}}}\right) - 1}$$

$v_{max}^{pepck_{mito}}$ for numerical value see Supplementary Table 1

$K_{eq}^{pepck_{mito}} = 1700$ [110]

$K_m^{oaa_{mito}} = 0.0085$ [116]

$K_m^{gtp_{mito}} = 0.022$ [114]

$K_m^{pep_{mito}} = 0.4$ [113]

$K_m^{gdp_{mito}} = 0.02$ [114]

$K_m^{hco3_{mito}} = 1.06$ [117]

Pyruvate carboxylase (Pc):

$$v_{pc} = v_{max}^{Pc} \cdot \frac{atp_{mito} \cdot pyr_{mito} \cdot hco3_{mito} - \frac{1}{k_{eq}^{Pc}} \cdot oaa_{mito} \cdot adp_{mito} \cdot h_{mito} \cdot p_{mito}}{\left(atp_{mito} + k_m^{atp_{mito}}\right) \cdot \left(pyr_{mito} + k_m^{pyr_{mito}}\right) \cdot \left(hco3_{mito} + k_m^{hco3_{mito}}\right)}$$

v_{max}^{Pc} for numerical value see Supplementary Table 1

$k_m^{atp_{mito}}$ =0.14 [118]

$k_m^{pyr_{mito}}$ =0.33 [118]

$k_m^{hco3_{mito}}$ =4.2 [118]

$k_{eq}^{Pc} = \ 6.55\, mM$ [110]

Lactate dehydrogenase (Ldh):

$$v_{ldh} = v_{max}^{ldh} * \frac{pyr_{cyt} \cdot nadh_{cyt} - \frac{1}{K_{eq}^{ldh}} \cdot lac_{cyt} \cdot nad_{cyt}}{\left(1 + \frac{nadh_{cyt}}{K_m^{nadh_{cyt}}}\right) \cdot \left(1 + \frac{pyr_{cyt}}{K_m^{pyr_{cyt}}}\right) + \left(1 + \frac{lac_{cyt}}{K_m^{lac_{cyt}}}\right) \cdot \left(1 + \frac{nad_{cyt}}{K_m^{nad_{cyt}}}\right) - 1}$$

v_{max}^{ldh} for numerical value see Supplementary Table 1

K_{eq}^{ldh} =9000 [119]

$K_m^{nadh_{cyt}} = 0.015$ [120]

$K_m^{pyr_{cyt}} = 0.15$ [120]

$K_m^{lac_{cyt}} = 36$ [121]

$K_m^{nad_{cyt}} = 0.11$ [120]

Lactate transport (LacT):

$$v_{lacT} = v_{max}^{lacT} \cdot \frac{lac_{ext} - lac_{cyt}}{1 + \frac{lac_{cyt}}{K_m^{lac_{cyt}}} + \frac{lac_{ext}}{K_m^{lac_{ext}}}}$$

v_{max}^{lacT} for numerical value see Supplementary Table 1

$K_m^{lac_{cyt}} = 2.42$ [122]

$K_m^{lac_{ext}} = 2.42$ [122]

pyruvate transport (PyrT):

$$v_{lacT} = v_{max}^{pyrT} \cdot \frac{pyr_{ext} - pyr_{cyt}}{1 + \frac{pyr_{cyt}}{K_m^{pyr_{cyt}}} + \frac{pyr_{ext}}{K_m^{pyr_{ext}}}}$$

v_{max}^{pyrT} for numerical value see Supplementary Table 1

$K_m^{pyr_{ext}} = 0.63$ [122]

$K_m^{pyr_{cyt}} = 0.63$ [122]

Mitochondrial pyruvate transport:

$$v_{pyrT_{mito}} = v_{max}^{pyrT_{mito}} \cdot \frac{pyr_{cyt} \cdot h_{cyt} - pyr_{mito} \cdot h_{mito}}{1 + \frac{pyr_{cyt}}{K_m^{pyr_{cyt}}} + \frac{pyr_{mito}}{K_m^{pyr_{mito}}}}$$

$v_{max}^{PyrT_{mito}}$ for numerical value see Supplementary Table 1

$K_m^{pyr_{cyt}}$ =0.15 [123]

$K_m^{pyr_{mito}}$ =0.15 [123]

Mitochondrial malate-phosphate transport

$$v_{malT} = v_{max}^{malT} \cdot \left(\frac{mal_{mito} \cdot p_{cyt} - mal_{cyt} \cdot p_{mito}}{\left(1 + \frac{mal_{mito}}{K_m^{mal_{mito}}}\right) \cdot \left(1 + \frac{p_{cyt}}{K_m^{p_{cyt}}}\right) + \left(1 + \frac{mal_{cyt}}{K_m^{mal_{cyt}}}\right) \cdot \left(1 + \frac{p_{mito}}{K_m^{p_{mito}}}\right) - 1} \right)$$

v_{max}^{MalT} for numerical value see Supplementary Table 1

$K_m^{p_{cyt}}$ =1.41 [124]

$K_m^{mal_{mito}} = 0.49$ [124]

$K_m^{p_{mito}}$ =1.41 [124]

$K_m^{mal_{cyt}} = 0.49$ [124]

Malate-pyruvate antiport (MalPyrT)

$$v_{mal-pyrT} = v_{max}^{mal-pyrT} * \left(\frac{mal_{mito} \cdot pyr_{cyt} - mal_{cyt} \cdot pyr_{mito}}{\left(1 + \frac{mal_{mito}}{K_m^{mal_{mito}}}\right) \cdot \left(1 + \frac{pyr_{cyt}}{K_m^{pyr_{cyt}}}\right) + \left(1 + \frac{mal_{cyt}}{K_m^{mal_{cyt}}}\right) \cdot \left(1 + \frac{pyr_{mito}}{K_m^{pyr_{mito}}}\right) - 1} \right)$$

$v_{max}^{MalPyrT}$ for numerical value see Supplementary Table 1

$K_m^{pyr_{cyt}} = 0.84$ [125]

$K_m^{mal_{cyt}} = 0.7$ [126]

$K_m^{pyr_{mito}} = 0.84$ [125]

$K_m^{mal_{cyt}} = 0.7$ [126]

cytosolic malate dehydrogenase (Mdh)

$$v_{mdh} = v_{max}^{Mdh} \cdot \frac{mal_{cyt} \cdot nad_{cyt} - \frac{1}{K_{eq}^{mdh_{cyt}}} \cdot oaa_{cyt} \cdot nadh_{cyt}}{\left(1 + \frac{mal_{cyt}}{K_m^{mal_{cyt}}}\right) \cdot \left(1 + \frac{nad_{cyt}}{K_m^{nad_{cyt}}}\right) + \left(1 + \frac{oaa_{cyt}}{K_m^{oaa_{cyt}}}\right) \cdot \left(1 + \frac{nadh_{cyt}}{K_m^{nadh_{cyt}}}\right) - 1}$$

v_{max}^{Mdh} for numerical value see Supplementary Table 1

$K_{eq}^{mdh_{cyt}} = 10^{-5}$ [54]

$K_m^{mal_{cyt}} = 1.1$ [127]

$K_m^{nad_{cyt}} = 0.114$ [127]

$K_m^{oaa_{cyt}} = 0.088$ [127]

$K_m^{nadh_{cyt}} = 0.026$ [127]

NADP dependent malic enzyme (cytosol)

$$v_{me} = V_{max}^{me} \cdot \left(\frac{mal_{cyt} \cdot nadp_{cyt} - {}^{1}\!/_{K_{eq}^{me}} \cdot pyr_{cyt} \cdot nadph_{cyt} \cdot hco3_{cyt}}{\left(1 + \frac{mal_{cyt}}{K_m^{mal_{cyt}}}\right) \cdot \left(1 + \frac{nadp_{cyt}}{K_m^{nadp_{cyt}}}\right) + \left(1 + \frac{pyr_{cyt}}{K_m^{pyr_{cyt}}}\right) \cdot \left(1 + \frac{nadph_{cyt}}{K_m^{nadph_{cyt}}}\right) \cdot \left(1 + \frac{hco3_{cyt}}{K_m^{hco3_{cyt}}}\right)} \right)$$

V_{max}^{me} for numerical value see Supplementary Table 1

$K_{eq}^{me} = 34.4$ [128]

$K_m^{mal_{cyt}} = 0.12$ [129]

$K_m^{nadp_{cyt}} = 0.0092$ [129]

$K_m^{pyr_{cyt}} = 5.9$ [129]

$K_m^{nadph_{cyt}} = 0.0053$ [129]

$K_m^{hco3_{cyt}} = 27.9$ [129]

Phosphoenolpyruvate transporter

$$v_{pepT} = V_{max}^{pepT} \cdot \left(\frac{pep_{mito} - 1/K_{eq}^{pepT} \cdot pep_{cyt}}{1 + \frac{pep_{cyt}}{K_m^{pep_{cyt}} \cdot \left(1 + \frac{cit_{cyt}}{K_i^{cit_{cyt}}}\right)} + \frac{pep_{mito}}{K_m^{pep_{mito}} \cdot \left(1 + \frac{cit_{mito}}{K_i^{cit_{mito}}}\right)}} \right)$$

V_{max}^{pepT} for numerical value see Supplementary Table 1

$K_{eq}^{pepT} = exp\left(\frac{-V_{mm} \cdot F}{R \cdot T}\right)$

$K_m^{pep_{cyt}} = 0.1$ [126]

$K_i^{cit_{cyt}} = 0.11$ [126]

$K_m^{pep_{mito}} = 0.1$ [126]

$K_i^{cit_{mito}} = 0.11$ [126]

Nudiki (cytosolic)

$$v_{ndk_{cyt}} = V_{max}^{ndk_{cyt}} \cdot \left(\frac{atp_{cyt} \cdot gdp_{cyt} - 1/K_{eq}^{ndk} \cdot adp_{cyt} \cdot gtp_{cyt}}{\left(1 + \frac{atp_{cyt}}{K_m^{atp_{cyt}}}\right) \cdot \left(1 + \frac{gdp_{cyt}}{K_m^{gdp_{cyt}}}\right) + \left(1 + \frac{adp_{cyt}}{K_m^{adp_{cyt}}}\right) \cdot \left(1 + \frac{gtp_{cyt}}{K_m^{gtp_{cyt}}}\right) - 1} \right)$$

$V_{max}^{ndk_{cyt}}$ for numerical value see Supplementary Table 1

$K_{eq}^{ndk} = 1$ [48]

$K_m^{atp_{cyt}} = 1.33$ [130]

$K_m^{gdp_{cyt}} = 0.031$ [130]

$K_m^{adp_{cyt}} = 0.042$ [130]

$K_m^{gtp_{cyt}} = 0.15$ [131]

Nudiki (mito)

$$v_{ndk_{mito}} = V_{max}^{ndk_{mito}} \cdot \left(\frac{atp_{mito} \cdot gdp_{mito} - 1/K_{eq}^{ndk} \cdot adp_{mito} \cdot gtp_{mito}}{\left(1 + \frac{atp_{mito}}{K_m^{atp_{mito}}}\right) \cdot \left(1 + \frac{gdp_{mito}}{K_m^{gdp_{mito}}}\right) + \left(1 + \frac{adp_{mito}}{K_m^{adp_{mito}}}\right) \cdot \left(1 + \frac{gtp_{mito}}{K_m^{gtp_{mito}}}\right) - 1} \right)$$

$V_{max}^{ndk_{mito}}$ for numerical value see Supplementary Table 1

$K_{eq}^{ndk} = 1$ [48]

$K_m^{atp_{mito}} = 1.66$ [130]

$K_m^{gdp_{mito}} = 0.036$ [130]

$K_m^{adp_{mito}} = 0.073$ [130]

$K_m^{gtp_{mito}} = 0.15$ [131]

Glycogen metabolism

alpha-D-Glucose 1-phosphate 1,6-phosphomutase:

$$v_{gpm} = v_{max}^{gpm} \cdot \frac{glc1p_{cyt} - \frac{1}{K_{eq}^{gpm}} \cdot glc6p_{cyt}}{1 + \frac{glc1p_{cyt}}{K_m^{glc1p_{cyt}}} + \frac{glc6p_{cyt}}{K_m^{glc6p_{cyt}}}}$$

v_{max}^{gpm} for numerical value see Supplementary Table 1

$K_{eq}^{gpm} = 16.2$ [132]

$K_m^{glc1p_{cyt}} = 0.045$ [133]

$K_m^{glc6p_{cyt}} = 0.67$ [133]

UTP:Glucose-1-phosphate uridylyltransferase (UPGase):

$$v_{upgase} = v_{max}^{upgase} \cdot \frac{utp_{cyt} \cdot glc1p_{cyt} - \frac{1}{K_{eq}^{upgase}} \cdot udpglc_{cyt} \cdot pp_{cyt}}{\left(1 + \frac{utp_{cyt}}{K_m^{utp_{cyt}}}\right) \cdot \left(1 + \frac{glc1p_{cyt}}{K_m^{glc1p_{cyt}}}\right) + \left(1 + \frac{udpglc_{cyt}}{K_m^{udpglc_{cyt}}}\right) \cdot \left(1 + \frac{pp_{cyt}}{K_m^{pp_{cyt}}}\right) - 1}$$

v_{max}^{UPGase} for numerical value see Supplementary Table 1

$K_{eq}^{upgase} = 0.3122$ [134]

$K_m^{utp_{cyt}} = 0.2$ [134]

$K_m^{glc1p_{cyt}} = 0.055$ [134]

$K_m^{udpglc_{cyt}} = 0.06$ [134]

$K_m^{pp_{cyt}} = 0.084$ [134]

Glycogen synthase (GS):

$$v_{gs} = V_{max}^{gs} \cdot \left((1-\gamma^{gs}) \cdot v_{gs}^{native} + \gamma^{gs} \cdot v_{gs}^{p} \right)$$

$$V_{max}^{gs} = V_0^{gs} \frac{(store - glyglc)}{(store - glyglc) + 10mM}$$

V_0^{gs} for numerical value see Supplementary Table 1

$$store = 400\ mM$$

$$v_{gs}^{native} = \frac{udpglc_{cyt}}{udpglc_{cyt} + K_{m-native}^{udpglc_{cyt}}}$$

$$K_{m-native}^{udpglc_{cyt}} = K_{0-native}^{udpglc_{cyt}} \cdot \left(1 - \frac{glc6p_{cyt}}{glc6p_{cyt} + K_{a}^{glc6p_{cyt}}}\right) \cdot \left(1 - \frac{fru1p_{cyt}}{fru1p_{cyt} + K_{a-native}^{fru1p_{cyt}}}\right) + K_{b-native}^{udpglc_{cyt}}$$

$K_{0-native}^{udpglc_{cyt}} = 1.4$ [135]

$K_{a}^{glc6p_{cyt}} = 0.007$ [135]

$K_{b-native}^{udpglc_{cyt}} = 0.2$ [135]

$K_{a-native}^{fru1p_{cyt}} = 1$ [136]

$$v_{gs}^{p} = \frac{udpglc_{cyt}}{udpglc_{cyt} + K_{m-p}^{udpglc_{cyt}}}$$

$$K_{m-p}^{udpglc_{cyt}} = K_{0-p}^{udpglc_{cyt}} \cdot \left(1 - \frac{glc6p_{cyt}}{glc6p_{cyt} + K_{a-p}^{glc6p_{cyt}}}\right) \cdot \left(1 - \frac{fru1p_{cyt}}{fru1p_{cyt} + K_{a-p}^{fru1p_{cyt}}}\right) + K_{b-p}^{udpglc_{cyt}}$$

$K_{0-p}^{udpglc_{cyt}} = 32$ [135]

$K_{a-p}^{glc6p_{cyt}} = 0.09$ [135]

$K_{b-p}^{udpglc_{cyt}} = 0.3$ [135]

$K_{a-p}^{fru1p_{cyt}} = 1$ [136]

Glycogen-phosphorylase (GP):

$$v_{gp} = \left((1-\gamma^{gp}) \cdot v_{gp}^{native} + \gamma^{gp} \cdot v_{gp}^{p} \right)$$

$$v_{gp}^{native} = V_{max}^{gp-native} \cdot \frac{glyglc \cdot p_{cyt} - \frac{1}{K_{eq}^{gp}} \cdot glc1p_{cyt}}{\left(1 + \frac{glyglc}{K_{m-native}^{glycogen}}\right) \cdot \left(1 + \frac{p_{cyt}}{K_{m-native}^{p_{cyt}}}\right) + \left(1 + \frac{glc1p_{cyt}}{K_{m-native}^{glc1p_{cyt}}}\right) - 1}$$

$$V_{max-native}^{gp} = \frac{V_0^{gp}}{K_{m-native}^{p_{cyt}} \cdot K_{m-native}^{glyglc}} \cdot \left(\frac{amp_{cyt}}{amp_{cyt} + K_{a-native}^{amp_{cyt}}}\right) \cdot \left(\frac{glyglc}{glyglc + 0.1 \cdot store}\right)$$

V_0^{gp} for numerical value see Supplementary Table 1

$store = 400\ mM$

$K_{a-native}^{amp} = 0.036$ [137]

$K_{eq}^{gp} = 0.21(mM)^{-1}$ [138]

$K_{m-native}^{glyglc} = 2.5$ [139]

$K_{m-native}^{p_{cyt}} = 500$ [139]

$$K_{m-native}^{glc1p_{cyt}} = K_0^{glc1p} \cdot \left(1 - \frac{amp_{cyt}}{amp_{cyt} + K_{a-glc1p}^{amp_{cyt}}}\right)$$

$K_0^{glc1p} = 250$ [139]

$K_{a-glc1p}^{amp_{cyt}} = 0.5$ [139]

$$v_{gp}^{p} = V_{max-p}^{gp} \cdot \frac{glyglc \cdot p_{cyt} - \frac{1}{K_{eq}^{gp}} \cdot glc1p_{cyt}}{\left(1 + \frac{glyglc}{K_{m-p}^{glycogen}}\right) \cdot \left(1 + \frac{p_{cyt}}{K_{m-p}^{p_{cyt}}}\right) + \left(1 + \frac{glc1p_{cyt}}{K_{m-p}^{glc1p_{cyt}}}\right) - 1}$$

$$V_{max-p}^{gp} = \frac{V_0^{gp}}{K_{m-p}^{p} \cdot K_{m-p}^{glyglc}} \cdot \left(\frac{amp_{cyt}}{amp_{cyt} + K_{a-p}^{amp_{cyt}}}\right) \cdot \left(\frac{glyglc}{glyglc + 0.1 \cdot store}\right)$$

$K_{a-p}^{amp} = 0.017$ [137]

$K_{m-p}^{glyglc} = 1.8$ [139]

$$K_{m-p}^{p} = K_{0-p}^{p_{cyt}} \cdot \left(1 + \frac{fru1p_{cyt}}{K_i^{fru1p_{cyt}}}\right)$$

$K_{0-p}^{p_{cyt}} = 2.1$ [139]

$K_{m-p}^{glc1p_{cyt}} = 0.7$ [139]

$K_i^{fru1p_{cyt}} = 1.19$ [140]

Nudiki (cytosolic) (udp)

$$v_{ndk_{cyt}} = V_{max}^{ndk_{cyt}} \cdot \left(\frac{atp_{cyt} \cdot udp_{cyt} - {}^{1}\!/_{K_{eq}^{ndk}} \cdot adp_{cyt} \cdot utp_{cyt}}{\left(1 + \frac{atp_{cyt}}{K_m^{atp_{cyt}}}\right) \cdot \left(1 + \frac{udp_{cyt}}{K_m^{udp_{cyt}}}\right) + \left(1 + \frac{adp_{cyt}}{K_m^{adp_{cyt}}}\right) \cdot \left(1 + \frac{utp_{cyt}}{K_m^{utp_{cyt}}}\right) - 1} \right)$$

$V_{max}^{ndk_{cyt}}$ for numerical value see Supplementary Table 1

$K_{eq}^{ndk} = 1$ [48]

$K_m^{atp_{cyt}} = 1.33$ [130]

$K_m^{udp_{cyt}} = 0.19$ [130]

$K_m^{adp_{cyt}} = 0.042$ [130]

$K_m^{utp_{cyt}} = 16$ [131]

Malate-Aspartate shuttle

aspartate –amino transferase (mitochondrial)

$$v_{asat_{mito}} = V_{max}^{asat} \cdot \left(\frac{asp_{mito} \cdot akg_{mito} - {}^{1}\!/_{K_{eq}^{asat}} \cdot oaa_{mito} \cdot glu_{mito}}{\left(1 + \frac{asp_{mito}}{K_m^{asp_{mito}}}\right) \cdot \left(1 + \frac{akg_{mito}}{K_m^{akg_{mito}}}\right) + \left(1 + \frac{oaa_{mito}}{K_m^{oaa_{mito}}}\right) \cdot \left(1 + \frac{glu_{mito}}{K_m^{glu_{mito}}}\right) - 1} \right)$$

V_{max}^{asat} for numerical value see Supplementary Table 1

$K_{eq}^{asat} = 0.147$ [141]

$K_m^{asp_{mito}} = 0.75$ [142]

$K_m^{akg_{mito}} =1.75$ [142]

$K_m^{oaa_{mito}}=1.84$ [142]

$K_m^{glu_{mito}}=0.48$ [142]

aspartate –amino transferase (cytosolic)

$$v_{asat} = V_{max}^{asat} \cdot \left(\frac{asp_{cyt} \cdot akg_{cyt} - {}^{1}\!/_{K_{eq}^{asat}} \cdot oaa_{cyt} \cdot glu_{cyt}}{\left(1 + \frac{asp_{cyt}}{K_m^{asp_{cyt}}}\right) \cdot \left(1 + \frac{akg_{cyt}}{K_m^{akg_{cyt}}}\right) + \left(1 + \frac{oaa_{cyt}}{K_m^{oaa_{cyt}}}\right) \cdot \left(1 + \frac{glu_{cyt}}{K_m^{glu_{cyt}}}\right) - 1} \right)$$

V_{max}^{asat} for numerical value see Supplementary Table 1

$K_{eq}^{asat} = 0.147$ [141]

$K_m^{asp_{cyt}} = 0.35$ [142]

$K_m^{akg_{cyt}} = 1.25$ [142]

$K_m^{oaa_{cyt}} = 2.05$ [142]

$K_m^{glu_{cyt}} = 0.38$ [142]

aspartate –glutamate carrier

$$v_{agc} = V_{max}^{agc} \cdot \left(\frac{asp_{mito} \cdot glu_{cyt} - {}^{1}\!/_{K_{eq}^{agc}} \cdot asp_{cyt} \cdot glu_{mito}}{\left(1 + \frac{asp_{mito}}{K_m^{asp_{mito}}}\right) \cdot \left(1 + \frac{glu_{cyt}}{K_m^{glu_{cyt}}}\right) + \left(1 + \frac{asp_{cyt}}{K_m^{asp_{cyt}}}\right) \cdot \left(1 + \frac{glu_{mito}}{K_m^{glu_{mito}}}\right) - 1} \right)$$

V_{max}^{asat} for numerical value see Supplementary Table 1

$K_{eq}^{asat} = exp\left(\frac{-V_{mm} \cdot F}{R \cdot T}\right) \cdot \left(\frac{H_{cyt}}{H_{mito}}\right)$

$K_m^{asp_{mito}} = K_0^{asp_{mito}} \cdot \left(1 + \frac{glu_{mito}}{K_i^{glu_{mito}}}\right)$

$K_0^{asp_{mito}} = 0.05$ [143]

$K_i^{glu_{mito}} = 0.5$ [144]

$K_m^{asp_{cyt}} = K_0^{asp_{cyt}} \cdot \left(1 + \frac{glu_{cyt}}{K_i^{glu_{cyt}}}\right)$

$K_0^{asp_{cyt}} = 0.043$ [144]

$K_i^{glu_{cyt}} = 0.5$ [144]

$K_m^{glu_{mito}} = 3$ [145]

$K_m^{glu_{cyt}} = 5.8$ [146]

Malate – α-ketogluterate carrier

$$v_{mac} = V_{max}^{mac} \cdot \left(\frac{mal_{cyt} \cdot akg_{mito} - {}^{1}\!/_{K_{eq}^{mac}} \cdot mal_{mito} \cdot akg_{cyt}}{\left(1 + \frac{mal_{cyt}}{K_m^{mal_{cyt}}}\right) \cdot \left(1 + \frac{akg_{mito}}{K_m^{akg_{mito}}}\right) + \left(1 + \frac{mal_{mito}}{K_m^{mal_{mito}}}\right) \cdot \left(1 + \frac{akg_{cyt}}{K_m^{akg_{cyt}}}\right) - 1} \right)$$

V_{max}^{mac} for numerical value see Supplementary Table 1

$K_{eq}^{mac} = 1$

$K_m^{mal_{cyt}}$ =0.7 [147]

$K_m^{akg_{mito}}$ =0.17 [147]

$K_m^{mal_{mito}}$ =1.4 [147]

$$K_m^{akg_{cyt}} = K_0^{akg_{cyt}} \cdot \left(1 + \frac{mal_{cyt}}{K_i^{mal_{cyt}}}\right) \cdot \left(1 + \frac{cit_{cyt}}{K_i^{cit_{cyt}}}\right) \cdot \left(1 + \frac{glu_{cyt}}{K_i^{glu_{cyt}}}\right)$$

$K_0^{akg_{cyt}}$ =0.046 [148]

$K_i^{mal_{cyt}} = 0.12$ [148]

$K_i^{cit_{cyt}} = 3.6$ [148]

$K_i^{glu_{cyt}} = 2.5$ [148]

Glycerol-3-phosphate dehydrogenase (cytosolic)

$$v_{g3pdh} = V_{max}^{g3pdh_{cyt}} \cdot \left(\frac{dhap_{cyt} \cdot nadh_{cyt} - {1}/{K_{eq}^{g3pdh}} \cdot g3p_{cyt} \cdot nad_{cyt}}{\left(1 + \frac{dhap_{cyt}}{K_m^{dhap_{cyt}}}\right) \cdot \left(1 + \frac{nadh_{cyt}}{K_m^{nadh_{cyt}}}\right) + \left(1 + \frac{g3p_{cyt}}{K_m^{g3p_{cyt}}}\right) \cdot \left(1 + \frac{nad_{cyt}}{K_m^{nad_{cyt}}}\right) - 1} \right)$$

$V_{max}^{g3pdh_{cyt}}$ for numerical value see Supplementary Table 1

$K_{eq}^{g3pdh_{cyt}} = \frac{1}{3 \cdot 10^{-4}}$ [149]

$K_m^{dhap_{cyt}} = 0.16$ [150]

$K_m^{nadh_{cyt}}$ =0.008 [150]

$K_m^{g3p_{cyt}}$ =0.22 [150]

$K_m^{nad_{cyt}}$ =0.013 [150]

Glycerol-3-phosphate dehydrogenase (mitochondrial)

$$v_{g3pdh_{mito}} = V_{max}^{g3pdh_{mito}} \cdot \left(\frac{dhap_{cyt} \cdot qh2_{mm} - {1}/{K_{eq}^{g3pdh}} \cdot g3p_{cyt} \cdot q_{mm}}{\left(1 + \frac{dhap_{cyt}}{K_m^{dhap_{cyt}}}\right) + \left(1 + \frac{g3p_{cyt}}{K_m^{g3p_{cyt}}}\right) - 1} \right)$$

$V_{max}^{g3pdh_{mito}}$ for numerical value see Supplementary Table 1

$$K_{eq}^{g3pdh_{mito}} = K_{eq}^{g3pdh_{cyt}} \cdot exp\left(\frac{\left(n \cdot E_0^{nad/nadh} + n \cdot E_0^{QH_2/Q}\right) \cdot F}{R \cdot T}\right)$$

$K_m^{dhap_{cyt}}$ =0.043 [151]

$K_m^{g3p_{cyt}} = 6$ [152]

$E_0^{nad/nadh} = -320mV$ [64]

$E_0^{QH_2/Q} = -87mV$ [32]

$n = 2$

Pentose phosphate shunt

Glucose-6-phopshate dehydrogenase

$$v_{g6pdh} = V_{max}^{g6pdh} \cdot \left(\left(\frac{glc6p_{cyt}}{glc6p_{cyt} + K_m^{glc6p_{cyt}} \cdot \left(1 + \frac{c16coa_{cyt}}{K_i^{c16coa_{cyt}}}\right)}\right) \cdot \left(\frac{nadp_{cyt}}{nadp_{cyt} + K_m^{nadp_{cyt}} \cdot \left(1 + \frac{nadph_{cyt}}{K_i^{nadph_{cyt}}}\right)}\right)\right)$$

V_{max}^{g6pdh} for numerical value see Supplementary Table 1

$K_m^{glc6p_{cyt}}$ =0.013 [153]

$K_i^{c16coa_{cyt}} = 0.029$ [154]

$K_m^{nadp_{cyt}} = 0.013$ [153]

$K_i^{nadph_{cyt}}$ =0.01 [155]

6-Phosphogluconolactase

$$v_{pgls} = V_{max}^{pgls} \cdot \left(\frac{pgl6_{cyt}}{pgl6_{cyt} + K_m^{pgl6_{cyt}}}\right)$$

V_{max}^{pgls} for numerical value see Supplementary Table 1

$K_m^{pgl6_{cyt}} = 0.7$ [156]

6-Phosphogluconate dehydrogenase

$$v_{pgdh} = V_{max}^{pgdh} \cdot \left(\frac{nadp_{cyt} \cdot pg6_{cyt} - 1/K_{eq}^{pgdh} \cdot ru5p_{cyt} \cdot nadph_{cyt}}{\left(1 + \frac{nadp_{cyt}}{K_m^{nadp_{cyt}} \cdot \left(1 + \frac{nadph_{cyt}}{K_i^{nadph_{cyt}}}\right)}\right) \cdot \left(1 + \frac{pg6_{cyt}}{K_m^{pg6_{cyt}}}\right) + \left(1 + \frac{ru5p_{cyt}}{K_m^{ru5p_{cyt}}}\right) \cdot \left(1 + \frac{co2_{cyt}}{K_m^{co2_{cyt}}}\right) \cdot \left(1 + \frac{nadph_{cyt}}{K_m^{nadph_{cyt}}}\right) - 1} \right)$$

V_{max}^{pgdh} for numerical value see Supplementary Table 1

$K_{eq}^{pgdh} = 74$ [157]

$K_m^{nadp_{cyt}} = 0.028$ [153]

$K_i^{nadph} = 0.02$ [158]

$K_m^{pg6_{cyt}} = 0.071$ [158]

$K_m^{co2_{cyt}} = 5$ [159]

$K_m^{nadph_{cyt}} = 0.001$ [159]

$K_m^{ru5p_{cyt}} = 0.123$ [159]

Ribulose-phosphate-3-epimerase

$$v_{rpe} = V_{max}^{rpe} \cdot \left(\frac{ru5p_{cyt} - 1/K_{eq}^{rpe} \cdot x5p_{cyt}}{1 + \frac{ru5p_{cyt}}{K_m^{ru5p_{cyt}}} + \frac{x5p_{cyt}}{K_m^{x5p_{cyt}}}} \right)$$

V_{max}^{rpe} for numerical value see Supplementary Table 1

$K_{eq}^{rpe} = 1.5$ [160]

$K_m^{ru5p_{cyt}} = 0.2$ [161]

$K_m^{x5p_{cyt}} = 0.5$

Ribose-phosphate-isomerase

$$v_{rpi} = V_{max}^{rpi} \cdot \left(\frac{r5p_{cyt} - {}^{1}\!/_{K_{eq}^{rpi}} \cdot ru5p_{cyt}}{1 + \frac{r5p_{cyt}}{K_m^{r5p_{cyt}}} + \frac{ru5p_{cyt}}{K_m^{ru5p_{cyt}}}} \right)$$

V_{max}^{rpi} for numerical value see Supplementary Table 1

$K_{eq}^{rpi} = 0.32$ [162]

$K_m^{r5p_{cyt}}$ =9.1 [163]

$K_m^{ru5p_{cyt}} = 0.78$ [163]

Transladolase

$$v_{taldo} = V_{max}^{taldo} \cdot \left(\frac{s7p_{cyt} \cdot grap_{cyt} - {}^{1}\!/_{K_{eq}^{taldo}} \cdot e4p_{cyt} \cdot fru6p_{cyt}}{\left(1 + \frac{s7p_{cyt}}{K_m^{s7p_{cyt}}}\right) \cdot \left(1 + \frac{grap_{cyt}}{K_m^{grap_{cyt}}}\right) + \left(1 + \frac{e4p_{cyt}}{K_m^{e4p_{cyt}}}\right) \cdot \left(1 + \frac{fru6p_{cyt}}{K_m^{fru6p_{cyt}}}\right) - 1} \right)$$

V_{max}^{tado} for numerical value see Supplementary Table 1

K_{eq}^{taldo} =0.95 [164]

$K_m^{s7p_{cyt}}$ =0.17 [164]

$K_m^{grap_{cyt}}$ =0.038 [165]

$K_m^{e4p_{cyt}} = 0.13$ [166]

$K_m^{fru6p_{cyt}} = 0.3$ [166]

Transketolase 1

$$v_{tketo1} = V_{max}^{tketo1} \cdot \left(\frac{s7p_{cyt} \cdot grap_{cyt} - {}^{1}\!/_{K_{eq}^{tketo1}} \cdot r5p_{cyt} \cdot x5p_{cyt}}{\left(1 + \frac{s7p_{cyt}}{K_m^{s7p_{cyt}}}\right) \cdot \left(1 + \frac{grap_{cyt}}{K_m^{grap_{cyt}}}\right) + \left(1 + \frac{r5p_{cyt}}{K_m^{r5p_{cyt}}}\right) \cdot \left(1 + \frac{x5p_{cyt}}{K_m^{x5p_{cyt}}}\right) \cdot -1} \right)$$

V_{max}^{tketo1} for numerical value see Supplementary Table 1

$K_{eq}^{tketo1} = 0.845$ [167]

$K_m^{s7p_{cyt}} = 0.285$ [165]

$K_m^{grap_{cyt}} = 0.38$ [165]

$K_m^{r5p_{cyt}} = 0.066$ [168]

$K_m^{x5p_{cyt}} = 0.15$ [169]

Transketolase 2

$$v_{tketo2} = V_{max}^{tketo1} \cdot \left(\frac{fru6p_{cyt} \cdot grap_{cyt} - 1/K_{eq}^{tketo2} \cdot e4p_{cyt} \cdot x5p_{cyt}}{\left(1 + \frac{fru6p_{cyt}}{K_m^{fru6p_{cyt}}}\right) \cdot \left(1 + \frac{grap_{cyt}}{K_m^{grap_{cyt}}}\right) + \left(1 + \frac{e4p_{cyt}}{K_m^{e4p_{cyt}}}\right) \cdot \left(1 + \frac{x5p_{cyt}}{K_m^{x5p_{cyt}}}\right) \cdot -1} \right)$$

V_{max}^{tketo2} for numerical value see Supplementary Table 1

$K_{eq}^{tketo2} = 0.084$ [167]

$K_m^{fru6p_{cyt}} = 0.34$ [170]

$K_m^{grap_{cyt}} = 0.38$ [165]

$K_m^{e4p_{cyt}} = 0.044$ [171]

$K_m^{x5p_{cyt}} = 0.16$ [171]

Fatty acid synthesis

Citrate-malate exchanger

$$v_{cit-mal} = \frac{V_{max}^{cit-mal}}{K_m^{cit_{mito}} \cdot K_m^{mal_{cyt}}}$$

$$\cdot \left(\frac{cit_{mito} \cdot mal_{cyt} - 1/K_{eq}^{cit-mal} \cdot cit_{cyt} \cdot mal_{mito}}{\left(1 + \frac{cit_{mito}}{K_m^{cit_{mito}}}\right) \cdot \left(1 + \frac{mal_{cyt}}{K_m^{mal_{cyt}}}\right) + \left(1 + \frac{cit_{cyt}}{K_m^{cit_{cyt}}}\right) \cdot \left(1 + \frac{mal_{mito}}{K_m^{mal_{mito}}}\right) \cdot -1} \right)$$

$$V_{max}^{cit-mal} = V_0^{cit-mal} \cdot \left(1 - \frac{\left(c16coa_{cyt}\right)^n}{\left(c16coa_{cyt}\right)^n + \left(K_i^{c16coa_{cyt}}\right)^n} \right)$$

$V_0^{cit-mal}$ for numerical value see Supplementary Table 1

$K_i^{c16coa_{cyt}} = 0.033$ [172]

$n = 3$ [172]

$K_{eq}^{cit-mal} = 1$

$$K_m^{cit_{mito}} = K_0^{cit_{mito}} \cdot \left(1 + \frac{suc_{mito}}{K_i^{suc_{mito}}}\right) \cdot \left(1 + \frac{isocit_{mito}}{K_i^{isocit_{mito}}}\right) \cdot \left(1 + \frac{pep_{mito}}{K_i^{pep_{mito}}}\right)$$

$K_0^{cit_{mito}} = 0.14$ [173]

$K_i^{suc_{mito}} = 2.5$ [173]

$K_i^{isocit_{mito}} = 0.08$ [173]

$K_i^{pep_{mito}} = 0.18$ [173]

$K_m^{mal_{cyt}} = 0.76$ [173]

$$K_m^{cit_{cyt}} = K_0^{cit_{cyt}} \cdot \left(1 + \frac{pep_{cyt}}{K_i^{pep_{cyt}}}\right)$$

$K_0^{cit_{cyt}} = 0.039$ [174]

$K_i^{pep_{cyt}} = 0.18$ [173]

$K_m^{mal_{mito}} = 0.76$ [173]

ATP dependent citrate lyase

$$v_{cit-lys} = V_{max}^{cit-lys} \cdot \left(\frac{cit_{cyt} \cdot coa_{cyt} \cdot atp_{cyt} - {}^{1}\!/_{K_{eq}^{cit-lys}} \cdot acoa_{cyt} \cdot adp_{cyt} \cdot p_{cyt} \cdot oaa_{cyt}}{\left(1 + \frac{cit_{cyt}}{K_m^{cit_{cyt}}}\right) \cdot \left(1 + \frac{coa_{cyt}}{K_m^{coa_{cyt}}}\right) \cdot \left(1 + \frac{atp_{cyt}}{K_m^{atp_{cyt}}}\right)} \right)$$

$V_{max}^{cit-lys}$ for numerical value see Supplementary Table 1

$K_{eq}^{cit-lys} = 8.92 \cdot 10^{-3}\ mM$ [175]

$K_m^{cit_{cyt}} = 0.17$ [176]

$K_m^{coa_{cyt}} = 0.0065$ [177]

$$K_m^{atp_{cyt}} = K_0^{atp_{cyt}} \cdot \left(1 + \frac{adp_{cyt}}{K_i^{adp_{cyt}}}\right)$$

$K_0^{atp_{cyt}} = 0.172$ [178]

$K_i^{adp_{cyt}} = 0.171$ [178]

Acetyl-CoA carboxylase 1

$$v_{acc1} = \gamma \cdot v_{acc1-p} + (1-\gamma) \cdot v_{acc1-up}$$

$$v_{acc1-p} = V_{max}^{acc1-p} \cdot \left(\frac{atp_{cyt}}{atp_{cyt} + K_m^{atp_{cyt}}}\right) \cdot \left(\frac{acoa_{cyt}}{acoa_{cyt} + K_m^{acoa_{cyt}}}\right) \cdot \left(\frac{hco3_{cyt}}{hco3_{cyt} + K_m^{hco3_{cyt}}}\right)$$

$$V_{max}^{acc1-p} = V_{acc1-p} \cdot \left(\frac{cit_{cyt}}{cit_{cyt} + K_a^{cit_{cyt}}}\right) \cdot \left(1 - \frac{malcoa_{cyt}}{malcoa_{cyt} + K_i^{malcoa_{cyt}}}\right) \cdot \left(1 - \frac{c16coa_{cyt}}{c16coa_{cyt} + K_i^{c16coa_{cyt}}}\right)$$

V_{acc1-p} for numerical value see Supplementary Table 1

$K_i^{c16coa_{cyt}} = 0.009$ [179]

$K_a^{cit_{cyt}} = 2.1$ [180]

$K_i^{malcoa_{cyt}} = 0.009$ [181]

$K_m^{atp_{cyt}} = 0.36$ [179]

$K_m^{acoa_{cyt}} = 0.025$ [182]

$K_m^{hco3_{cyt}} = 2.5$ [182]

$$v_{acc1-up} = V_{max}^{acc1-up} \cdot \left(\frac{atp_{cyt}}{atp_{cyt} + K_m^{atp_{cyt}}}\right) \cdot \left(\frac{acoa_{cyt}}{acoa_{cyt} + K_m^{acoa_{cyt}}}\right) \cdot \left(\frac{hco3_{cyt}}{hco3_{cyt} + K_m^{hco3_{cyt}}}\right)$$

$$V_{max}^{acc1-up} = V_{acc1-up} \cdot \left(1 + n_{up} \frac{cit_{cyt}}{cit_{cyt} + K_a^{cit_{cyt}}}\right) \cdot \left(1 - \frac{malcoa_{cyt}}{malcoa_{cyt} + K_i^{malcoa_{cyt}}}\right)$$

$V_{acc1-up} = 2.5 \cdot V_{acc1-p}$ [180]

$n_{up} = 1.4$ [180]

$K_a^{cit_{cyt}} = 0.8$ [180]

$K_m^{atp_{cyt}} = 0.36$ [179]

$K_m^{acoa_{cyt}} = 0.025$ [182]

$K_i^{malcoa_{cyt}} = 0.009$ [181]

$K_m^{hco3_{cyt}} = 2.5$ [182]

Acetyl-CoA carboxylase 2

$$v_{acc2} = \gamma \cdot v_{acc2-p} + (1-\gamma) \cdot v_{acc2-up}$$

$$v_{acc2-p} = V_{max}^{acc2-p} \cdot \left(\frac{ATP_{cyt}}{ATP_{cyt} + K_m^{ATP_{cyt}}} \right) \cdot \left(\frac{acoa_{cyt}}{acoa_{cyt} + K_m^{acoa_{cyt}} \cdot \left(1 + \frac{malcoa2_{imm}}{K_i^{malcoa2_{imm}}} \right)} \right)$$

$$V_{max}^{acc2-p} = V_{acc2-p0} \cdot \left(\frac{cit_{cyt}}{cit_{cyt} + K_a^{cit_{cyt}}} \right)$$

$V_{acc2-p0}$ for numerical value see Supplementary Table 1

$K_m^{ATP_{cyt}} = 0.147$ [181]

$K_m^{acoa_{cyt}} = 0.037$ [181]

$K_a^{cit_{cyt}} = 2$ [180]

$K_i^{malcoa2_{cyt}} = 0.009$ [181]

$$v_{acc2-up} = V_{max}^{acc2-up} \cdot \left(\frac{atp_{cyt}}{atp_{cyt} + K_m^{atp_{cyt}}} \right) \cdot \left(\frac{acoa_{cyt}}{acoa_{cyt} + K_m^{acoa_{cyt}} \cdot \left(1 + \frac{macoa2_{imm}}{K_i^{malcoa2_{imm}}} \right)} \right)$$

$$V_{max}^{acc2-up} = V_{acc2-up} \cdot \left(1 + n_{up} \frac{cit_{cyt}}{cit_{cyt} + K_a^{cit_{cyt}}} \right)$$

$V_{acc2-up} = 2.5 \cdot V_{acc2-p0}$ [180]

$K_m^{ATP_{cyt}} = 0.147$ [181]

$K_m^{acoa_{cyt}} = 0.037$ [181]

$n_{up} = 1.4$ [180]

$K_a^{cit_{cyt}} = 0.8$ [180]

$K_i^{malcoa_{imm}} = 0.009$ [181]

Malonyl-CoA decarboxylase 2

$$v_{mdc2} = \gamma \cdot V_{max}^{mdc2} \cdot \left(\frac{malcoa2_{imm}}{malcoa2_{imm} + K_m^{malcoa2_{imm}}} \right)$$

V_{max}^{mdc2} for numerical value see Supplementary Table 1

$K_m^{malcoa2_{cyt}} = 0.054$ [183]

Fatty acid synthase c2->c4

$$v_{fas-c4} = V_{max}^{fas-c4} \cdot \left(\frac{acoa_{cyt}}{acoa_{cyt} + K_m^{acoa_{cyt}}} \right) \cdot \left(\frac{malcoa_{cyt}}{malcoa_{cyt} + K_m^{malcoa_{cyt}}} \right) \cdot \left(\frac{nadph_{cyt}^n}{nadph_{cyt}^n + \left(K_m^{nadph_{cyt}}\right)^n} \right)$$

V_{max}^{fas-c4} for numerical value see Supplementary Table 1

$K_m^{acoa_{cyt}} = 0.018$ [184] 0.0044

$K_m^{malcoa_{cyt}} = 0.026$ [184] 0.01

$K_m^{nadph_{cyt}} = 0.012$ [184]

$n = 2$

Fatty acid synthase c4->c6

$$v_{fas-c6} = V_{max}^{fas-c6} \cdot \left(\frac{c4coa_{cyt}}{c4coa_{cyt} + K_m^{c4coa_{cyt}}} \right) \cdot \left(\frac{malcoa_{cyt}}{malcoa_{cyt} + K_m^{malcoa_{cyt}}} \right) \cdot \left(\frac{nadph_{cyt}^n}{nadph_{cyt}^n + \left(K_m^{nadph_{cyt}}\right)^n} \right)$$

V_{max}^{fas-c6} for numerical value see Supplementary Table 1

$K_m^{c4coa_{cyt}} = 0.012$ [184]

$K_m^{malcoa_{cyt}} = 0.026$ [184]

$K_m^{nadph_{cyt}} = 0.012$ [184]

$n = 2$

Fatty acid synthase c6->c8

$$v_{fas-c8} = V_{max}^{fas-c8} \cdot \left(\frac{c6coa_{cyt}}{c6coa_{cyt} + K_m^{c6coa_{cyt}}} \right) \cdot \left(\frac{malcoa_{cyt}}{malcoa_{cyt} + K_m^{malcoa_{cyt}}} \right) \cdot \left(\frac{nadph_{cyt}^n}{nadph_{cyt}^n + \left(K_m^{nadph_{cyt}}\right)^n} \right)$$

V_{max}^{fas-c8} for numerical value see Supplementary Table 1

$K_m^{c6coa_{cyt}} = 0.068$ [184]

$K_m^{malcoa_{cyt}} = 0.026$ [184]

$K_m^{nadph_{cyt}} = 0.012$ [184]

$n = 2$

Fatty acid synthase c8->c10

$$v_{fas-c10} = V_{max}^{fas-c10} \cdot \left(\frac{c8coa_{cyt}}{c8coa_{cyt} + K_m^{c8coa_{cyt}}} \right) \cdot \left(\frac{malcoa_{cyt}}{malcoa_{cyt} + K_m^{malcoa_{cyt}}} \right) \cdot \left(\frac{nadph_{cyt}^n}{nadph_{cyt}^n + \left(K_m^{nadph_{cyt}}\right)^n} \right)$$

$V_{max}^{fas-c10}$ for numerical value see Supplementary Table 1

$K_m^{c8coa_{cyt}} = 0.125$ [184]

$K_m^{malcoa_{cyt}} = 0.026$ [184]

$K_m^{nadph_{cyt}} = 0.012$ [184]

$n = 2$

Fatty acid synthase c10->c12

$$v_{fas-c12} = V_{max}^{fas-c12} \cdot \left(\frac{c10coa_{cyt}}{c10coa_{cyt} + K_m^{c10coa_{cyt}}} \right) \cdot \left(\frac{malcoa_{cyt}}{malcoa_{cyt} + K_m^{malcoa_{cyt}}} \right) \cdot \left(\frac{nadph_{cyt}^n}{nadph_{cyt}^n + \left(K_m^{nadph_{cyt}}\right)^n} \right)$$

$V_{max}^{fas-c12}$ for numerical value see Supplementary Table 1

$K_m^{c10coa_{cyt}} = 0.05$

$K_m^{malcoa_{cyt}} = 0.026$ [184]

$K_m^{nadph_{cyt}} = 0.012$ [184]

$n = 2$

Fatty acid synthase c12->c14

$$v_{fas-c14} = V_{max}^{fas-c14} \cdot \left(\frac{c12coa_{cyt}}{c12coa_{cyt} + K_m^{c12coa_{cyt}}} \right) \cdot \left(\frac{malcoa_{cyt}}{malcoa_{cyt} + K_m^{malcoa_{cyt}}} \right) \cdot \left(\frac{nadph_{cyt}^n}{nadph_{cyt}^n + \left(K_m^{nadph_{cyt}} \right)^n} \right)$$

$V_{max}^{fas-c14}$ for numerical value see Supplementary Table 1

$K_m^{c12coa_{cyt}} = 0.05$

$K_m^{malcoa_{cyt}} = 0.026$ [184]

$K_m^{nadph_{cyt}} = 0.012$ [184]

$n = 2$

Fatty acid synthase c14->c16

$$v_{fas-c16} = V_{max}^{fas-c16} \cdot \left(\frac{c14coa_{cyt}}{c14coa_{cyt} + K_m^{c14coa_{cyt}}} \right) \cdot \left(\frac{malcoa_{cyt}}{malcoa_{cyt} + K_m^{malcoa_{cyt}}} \right) \cdot \left(\frac{nadph_{cyt}^n}{nadph_{cyt}^n + \left(K_m^{nadph_{cyt}} \right)^n} \right)$$

$V_{max}^{fas-c16}$ for numerical value see Supplementary Table 1

$K_m^{c14coa_{cyt}} = 0.05$

$K_m^{malcoa_{cyt}} = 0.026$ [184]

$K_m^{nadph_{cyt}} = 0.012$ [184]

$n = 2$

TAG synthesis

Glycerol-uptake

$$v_{glycT} = V_{max}^{glycT} \cdot \left(\frac{glyc_{ext} - glyc_{cyt}}{1 + \frac{glyc_{ext}}{K_m^{glyc_{ext}}} + \frac{glyc_{cyt}}{K_m^{glyc_{cyt}}}} \right)$$

V_{max}^{glycT} for numerical value see Supplementary Table 1

$K_m^{glyc_{ext}} = 0.4$ [185]

$K_m^{glyc_{cyt}} = 0.4$ [185]

Glycerol kinase

$$v_{glycK} = V_{max}^{glycK} \cdot \left(\frac{glyc_{cyt}}{glyc_{cyt} + K_m^{glyc_{cyt}} \cdot \left(1 + \frac{g3p_{cyt}}{K_i^{g3p_{cyt}}}\right)} \right) \cdot \left(\frac{atp_{cyt}}{atp_{cyt} + K_m^{atp_{cyt}}} \right)$$

V_{max}^{glycK} for numerical value see Supplementary Table 1

$K_m^{glyc_{cyt}} = 0.04$ [185]

$K_i^{g3p_{cyt}} = 0.58$ [186]

$K_m^{ATP_{cyt}} = 0.058$ [186]

Glycerophosphate Acyltransferase

$$v_{gpat} = V_{max}^{gpat} \cdot \left(\frac{g3p_{cyt}}{g3p_{cyt} + K_m^{g3p_{cyt}}} \right) \cdot \left(\frac{c16coa_{cyt}}{c16coa_{cyt} + K_m^{c16coa_{cyt}}} \right)$$

V_{max}^{gpat} for numerical value see Supplementary Table 1

$K_m^{g3p_{cyt}}$ =0.2 [187]

$K_m^{c16coa_{cyt}}$ =0.09 [188]

Acetyl glycerol-3-phosphate acyltransferase

$$v_{agpat} = V_{max}^{agpat} \cdot \left(\frac{lpa_{er}}{lpa_{er} + K_m^{lpa_{er}}} \right) \cdot \left(\frac{c16coa_{cyt}}{c16coa_{cyt} + K_m^{c16coa_{cyt}}} \right)$$

V_{max}^{agpat} for numerical value see Supplementary Table 1

$K_m^{lpa_{er}} = 0.0065$ [189]

$K_m^{c16coa_{cyt}}$ =0.004 [189]

Phosphatidic acid phosphatase

$$v_{pap} = V_{max}^{pap} \cdot \left(\frac{pa_{er}^n}{pa_{er}^n + \left(K_m^{pa_{er}}\right)^n} \right)$$

V_{max}^{pap} for numerical value see Supplementary Table 1

$K_m^{pa_{er}} = 0.35$ [190] (human)

$n = 2.2$ [190] (human)

Diacylglycerol acyltransferase

$$v_{dgat} = V_{max}^{dgat} \cdot \left(\frac{dag_{er}}{dag_{er} + K_m^{dag_{er}}} \right) \cdot \left(\frac{c16coa_{cyt}}{c16coa_{cyt} + K_m^{c16coa_{cyt}}} \right)$$

V_{max}^{dgat} for numerical value see Supplementary Table 1

$K_m^{dag_{cyt}} = 0.03$ [191]

$K_m^{c16coa_{cyt}} = 0.1$ [192]

Urea synthesis

ammonia-uptake

$$v_{nh_3-uptake} = V_{max}^{nh_3-uptake} \cdot \left(\frac{nh3_{ext} - keq_{nh3-uptake} \cdot nh3_{cyt}}{nh_{3_{ext}} + K_m^{nh3_{ext}}} \right)$$

$V_{max}^{nh_3-uptake}$ for numerical value see Supplementary Table 1

$$keq_{nh3-uptake} = e\text{xp}\left(\frac{Vm \cdot F}{R \cdot T} \right)$$

$K_m^{nh3_{ext}}$ = 2.3 [193]

$Vm = \ -30\ mV$

ammonia-diffusion

$$v_{nh_3-diff} = V_{max}^{nh_3-diff} \cdot \left(nh3_{cyt} - nh3_{mito} \right)$$

$V_{max}^{nh_3-diff}$ for numerical value see Supplementary Table 1

Mitochondrial glutamine transport

$$v_{glnT_{mito}} = V_{max}^{glnT_{mito}} \cdot \left(\frac{gln_{cyt} \cdot h_{cyt} - gln_{mito} \cdot h_{mito}}{1 + \frac{gln_{cyt}}{K_m^{gln_{cyt}}}} \right)$$

$V_{max}^{glnT_{mito}}$ for numerical value see Supplementary Table 1

$K_m^{gln_{cyt}}$ =3.3 [194]

Glutaminase: regulated by reversible binding to mitochondrial membrane [195]

$$v_{glnase} = \gamma \cdot v_{glnase-b} + (1-\gamma) \cdot v_{glnase-free}$$ [196]

$$v_{glnase-b} = V_{max}^{glnase-b} \cdot \left(\frac{gln_{mito}^n}{gln_{mito}^n + \left(K_m^{gln_{mito}}\right)^n} \right)$$

$V_{max}^{glnase-b}$ for numerical value see Supplementary Table 1

$K_m^{gln_{mito}} = 3.85$ [197]

$n = 2.47$ [197]

$$v_{glnase-free} = V_{max}^{glnase-free} \cdot \left(\frac{gln_{mito}}{gln_{mito} + K_m^{gln_{mito}}} \right)$$

$V_{max}^{glnase-free} = 0.5 \cdot V_{max}^{glnase-b}$

$K_m^{gln_{mito}} = 21$ [198]

Glutamatedehydrogenase (nad - dependent)

$$v_{gdh} = V_{max}^{gdh} \cdot \left(\frac{glu_{mito} \cdot nad_{mito} - {}^{1}\!/\!_{K_{eq}^{gdh}} \cdot akg_{mito} \cdot nadh_{mito} \cdot nh3_{mito}}{\left(1 + \frac{glu_{mito}}{K_m^{glu_{mito}}}\right) \cdot \left(1 + \frac{nad_{mito}}{K_m^{nad_{mito}}}\right) + \left(1 + \frac{akg_{mito}}{K_m^{akg_{mito}}}\right)\left(1 + \frac{nadh_{mito}}{K_m^{nadh_{mito}}}\right) \cdot \left(1 + \frac{nh3_{mito}}{K_m^{nh3_{mito}}}\right)} \right)$$

$$V_{max}^{gdh} = V_0^{gdh} \cdot \left(1 - \frac{c16coa_{mito}}{c16coa_{mito} + K_i^{c16coa_{mito}}}\right) \cdot \left(A_0 + (1 - A_0)\left(1 - \frac{mal_{mito}}{mal_{mito} + K_i^{mal_{mito}}}\right)\right) \cdot$$

V_0^{gdh} for numerical value see Supplementary Table 1

$K_i^{c16coa_{mito}} = 0.0001$ [199]

$A_0 = 0.7$ [199]

$K_i^{mal_{mito}} = 2$ [199]

$K_{eq}^{gdh} = 0.00387mM$ [119]

$$K_m^{glu_{mito}} = K_0^{glu_{mito}} \cdot \left(1 + \frac{akg_{mito}}{K_i^{akg_{mito}}}\right) \cdot \left(1 + \frac{nh3_{mito}}{K_i^{nh3_{mito}}}\right)$$

$K_0^{glu_{mito}}$ =4.61 [200]

$K_i^{akg_{mito}}$ =1.49 [201]

$K_i^{nh3_{mito}} = 3.1$ [201]

$K_m^{nad_{mito}} = K_0^{nad_{mito}} \cdot \left(1 + \frac{nadh_{mito}}{K_i^{nadh_{mito}}}\right)$

$K_0^{nad_{mito}}$ =0.364 [200]

$K_i^{nadh_{mito}}$ =0.0086 [201]

$K_m^{akg_{mito}}$ =0.18 [202]

$K_m^{nadh_{mito}}$ =0.03 [202]

$K_m^{nh3_{mito}} = 20$ [202]

Glutamatedehydrogenase (nadp – dependent)

$$v_{gdh-nadp} = V_{max}^{gdh-nadp} \cdot \left(\frac{glu_{mito} \cdot nadp_{mito} - {}^{1}\!/_{K_{eq}^{gdh}} \cdot akg_{mito} \cdot nadph_{mito} \cdot nh3_{mito}}{\left(1 + \frac{glu_{mito}}{K_m^{glu_{mito}}}\right) \cdot \left(1 + \frac{nadp_{mito}}{K_m^{nadp_{mito}}}\right) + \left(1 + \frac{akg_{mito}}{K_m^{akg_{mito}}}\right)\left(1 + \frac{nadph_{mito}}{K_m^{nadph_{mito}}}\right) \cdot \left(1 + \frac{nh3_{mito}}{K_m^{nh3_{mito}}}\right)} \right)$$

$V_{max}^{gdh-nadp}$ for numerical value see Supplementary Table 1

$$V_{max}^{gdh-nadp} = V_0^{gdh-nadp} \cdot \left(1 - \frac{c16coa_{mito}}{c16coa_{mito} + K_i^{c16coa_{mito}}}\right) \cdot \left(A_0 + (1 - A_0)\left(1 - \frac{mal_{mito}}{mal_{mito} + K_i^{mal_{mito}}}\right)\right) \cdot$$

$V_0^{gdh-nadp}$ for numerical value see Supplementary Table 1

$K_i^{c16coa_{mito}} = 0.0001$ [199]

$A_0 = 0.7$ [199]

$K_i^{mal_{mito}} = 2$ [199]

$K_{eq}^{gdh} = 0.00387mM$ [119]

$K_m^{glu_{mito}} = 0.6$ [202]

$K_m^{nadp_{mito}} = 0.23$ [202]

$K_m^{akg_{mito}} = 0.18$ [202]

$K_m^{nadph_{mito}}$ =0.02 [202]

$K_m^{nh_{3_{mito}}} = 20$ [202]

Arginine Transporter

$$v_{argT} = V_{max}^{argT} \cdot \frac{\left(arg_{cyt} - arg_{mito}\right)}{1 + \frac{arg_{cyt}}{K_m^{arg_{cyt}}} + \frac{arg_{mito}}{K_m^{arg_{mito}}}}$$

V_{max}^{argT} for numerical value see Supplementary Table 1

$K_m^{arg_{cyt}} = \ 0.08$ [203]

$K_m^{arg_{mito}} = 0.08$ [203]

Acetyl-glutamate synthesis

$$v_{aglu-syn} = V_{max}^{aglu-syn} \cdot \left(\frac{glu_{mito}}{glu_{mito} + K_m^{glu_{mito}}}\right) \cdot \left(\frac{acoa_{mito}}{acoa_{mito} + K_m^{acoa_{mito}}}\right)$$

$$V_{max}^{aglu-syn} = V_{aglu_{syn}} \cdot \left(1 - \frac{valcoa}{valcoa + K_i^{valcoa}}\right) \cdot \left(1 + K_0 \cdot \frac{arg_{mito}^n}{arg_{mito}^n + \left(K_a^{arg_{mito}}\right)^n}\right)$$

$V_{aglu_{syn}}$ for numerical value see Supplementary Table 1

$K_i^{valcoa} = 0.46$ [204]

$K_0 = 19.8$ [205]

$K_a^{arg_{mito}} = 0.22$ [205]

$n = 1.5$ [205]

$$K_m^{glu_{mito}} = K_0^{glu_{mito}} \cdot \left(1 + \frac{acglu_{mito}}{K_i^{acglu_{mito}}}\right)$$

$K_0^{glu_{mito}} = 1$ [205]

$K_i^{acglu_{mito}} = 0.25$ [205]

$$K_m^{acoa_{mito}} = K_0^{acoa_{mito}} \cdot \left(1 + \frac{acglu_{mito}}{K_i^{acglu_{mito}}}\right) \cdot \left(1 + \frac{valcoa_{mito}}{K_i^{valcoa_{mito}}}\right)$$

$K_0^{acoa_{mito}} = 0.7$ [206]

$K_i^{acglu_{mito}} = 0.2$ [206]

$K_i^{valcoa_{mito}} = 0.46$ [204]

Acetyl-glutamate transporter [207]

$$v_{acgluT} = V_{max}^{acgluT} \cdot \left(acglu_{mito} - acglu_{cyt} \right)$$

V_{max}^{acgluT} for numerical value see Supplementary Table 1

Acetyl-glutamate hydrolase

$$v_{acglu-hyd} = V_{max}^{acglu-hyd} \cdot \left(\frac{acglu_{cyt}}{acglu_{cyt} + K_m^{acglu_{cyt}}} \right)$$

$V_{max}^{acglu-hyd}$ for numerical value see Supplementary Table 1

$K_m^{acglu_{cyt}} = 10$ [208]

glutamate carrier

$$v_{gluT_{mito}} = V_{max}^{gluT_{mito}} \cdot \left(\frac{glu_{cyt} \cdot h_{cyt} - glu_{mito} \cdot h_{mito}}{1 + \frac{glu_{cyt}}{K_m^{glu_{cyt}}} + \frac{glu_{mito}}{K_m^{glu_{mito}}}} \right)$$

$V_{max}^{gluT_{mito}}$ for numerical value see Supplementary Table 1

$K_m^{glu_{cyt}} = 5$ [209]

$K_m^{glu_{mito}} = 0.25$ [210]

Acetyl-CoA synthetase

$$v_{acoa-syn} = V_{max}^{acoa-syn} \cdot \frac{atp_{cyt} \cdot acetate_{cyt} \cdot coa_{cyt} - 1/K_{eq}^{acoa-syn} \cdot pp_{cyt} \cdot acoa_{cyt} \cdot amp_{cyt}}{\left(atp_{cyt} + K_m^{atp_{cyt}}\right) \cdot \left(acetate_{cyt} + K_m^{acetate_{cyt}}\right) \cdot \left(coa_{cyt} + K_m^{coa_{cyt}}\right)}$$

$V_{max}^{acoa-syn}$ for numerical value see Supplementary Table 1

$K_{eq}^{acoa-syn} = 16$

$K_m^{atp_{cyt}}$ =0.57 [211]

$K_m^{acetate_{cyt}}$ =0.0525 [211]

$K_m^{coa_{cyt}}$ =0.0505 [211]

Carbamoyl-phosphate synthase

$$v_{cps} = V_{max}^{cps} \cdot \left(\frac{nh_{3_{mito}}}{nh_{3_{mito}} + K_m^{nh_{3_{mito}}}} \right) \cdot \left(\frac{atp_{mito}}{atp_{mito} + K_m^{atp_{mito}}} \right) \cdot \left(\frac{hco3_{mito}}{hco3_{mito} + K_m^{hco3_{mito}}} \right)$$

$$V_{max}^{cps} = V_{cps} \cdot \left(1 - \frac{c16coa_{mito}}{c16coa_{mito} + K_i^{c16coa_{mito}}} \right) \cdot \left(\frac{acglu_{mito}}{acglu_{mito} + K_a^{acglu_{mito}}} \right)$$

V_{cps} for numerical value see Supplementary Table 1

$K_i^{c16coa_{mito}} = 0.019$ [212]

$K_a^{acgl_{mito}} = 0.19$ [213]

$K_m^{nh_{3_{mito}}}$ =0.21[212]

$K_m^{atp_{mito}}$ =0.9 [214]

$K_m^{hco3_{mito}}$ =4.5 [214]

Ornithine transcarbamylase

$$v_{otc} = V_{max}^{otc} \cdot \left(\frac{cmp_{mito}}{cmp_{mito} + K_m^{cmp_{mito}} \cdot \left(1 + \frac{p_{mito}}{K_i^{p_{mito}}} \right)} \right) \cdot \left(\frac{orn_{mito}}{orn_{mito} + K_m^{orn_{mito}}} \right)$$

V_{max}^{otc} for numerical value see Supplementary Table 1

$K_m^{cmp_{mito}} = 0.026$ [215]

$K_i^{p_{mito}} = 0.25$ [215]

$K_m^{orn_{mito}} = 0.4$ [215]

Ornithine Citrulline exchanger

$$v_{ocT} = V_{max}^{ocT} \cdot \left(\frac{orn_{cyt} \cdot ctl_{mito} \cdot h_{mito} - orn_{mito} \cdot ctl_{cyt} \cdot h_{cyt}}{\left(1 + \frac{orn_{cyt}}{K_m^{orn_{cyt}}}\right) \cdot \left(1 + \frac{ctl_{mito}}{K_m^{ctl_{mito}}}\right) + \left(1 + \frac{orn_{mito}}{K_m^{orn_{mito}}}\right) \cdot \left(1 + \frac{ctl_{cyt}}{K_m^{ctl_{cyt}}}\right) - 1} \right)$$

V_{max}^{ocT} for numerical value see Supplementary Table 1

$K_m^{orn_{cyt}} = 0.16$ [216]

$K_m^{ctl_{mito}} = 3.6$ [216]

$K_m^{orn_{mito}} = 0.16$ [216]

$K_m^{ctl_{cyt}} = 3.6$ [216]

Argininosuccinate synthase

$$v_{ass} = V_{max}^{ass} \cdot \left(\frac{ctl_{cyt}}{ctl_{cyt} + K_m^{ctl_{cyt}} \cdot \left(1 + \frac{argsucc_{cyt}}{K_i^{argsucc_{cyt}^{ctl_{cyt}}}}\right)} \right) \cdot \left(\frac{asp_{cyt}}{asp_{cyt} + K_m^{asp_{cyt}} \cdot \left(1 + \frac{argsucc_{cyt}}{K_i^{argsucc_{cyt}^{asp_{cyt}}}}\right)} \right) \cdot \left(\frac{atp_{cyt}}{atp_{cyt} + K_m^{atp_{cyt}} \cdot \left(1 + \frac{adp_{cyt}}{K_i^{adp_{cyt}}}\right) \cdot \left(1 + \frac{argsucc_{cyt}}{K_i^{argsucc_{cyt}^{atp_{cyt}}}}\right)} \right)$$

V_{max}^{ass} for numerical value see Supplementary Table 1

$K_m^{ctl_{cyt}} = 0.2$ [217]

$K_i^{argsucc_{cyt}^{ctl_{cyt}}} = 0.3$ [218]

$K_m^{asp_{cyt}} = 0.15$ [217]

$K_i^{argsucc_{cyt}^{asp_{cyt}}} = 0.46$ [218]

$K_m^{atp_{cyt}} = 0.32$ [218]

$K_i^{adp_{cyt}} = 0.5$ [217]

$K_i^{argsucc_{cyt}^{atp_{cyt}}} = 1.1$ [218]

Argininosuccinate lyase

$$v_{asl} = V_{max}^{asl} \cdot \left(\frac{argsucc_{cyt} - {}^{1}\!/\!{}_{K_{eq}^{asl}} \cdot fum_{cyt} \cdot arg_{cyt}}{\left(1 + \frac{argsucc_{cyt}}{K_m^{argsucc_{cyt}}}\right) + \left(1 + \frac{fum_{cyt}}{K_m^{fum_{cyt}}}\right) \cdot \left(1 + \frac{arg_{cyt}}{K_m^{arg_{cyt}}}\right) - 1} \right)$$

V_{max}^{asl} for numerical value see Supplementary Table 1

$K_{eq}^{asl} = 3.7\ mM$ [219]

$K_m^{argsucc_{cyt}} = 0.2$ [220]

$K_m^{fum_{cyt}} = 5.3$ [220]

$K_m^{arg_{cyt}} = 3$ [220]

Argininase

$$v_{argase} = V_{max}^{argase} \cdot \left(\frac{arg_{cyt}}{arg_{cyt} + K_m^{arg_{cyt}} \cdot \left(1 + \frac{orn_{cyt}}{K_i^{orn_{cyt}}}\right)} \right)$$

V_{max}^{argase} for numerical value see Supplementary Table 1

$K_m^{arg_{cyt}} =1.7$ [221]

$K_i^{orn_{cyt}} = 5$ [222]

Urea transport

$$v_{ureaT} = V_{max}^{ureaT} \cdot \left(\frac{urea_{cyt.}}{urea_{cyt.} + K_m^{urea_{cyt}}} \right)$$

V_{max}^{ureaT} for numerical value see Supplementary Table 1

$K_m^{urea_{cyt}} = 200$ [223]

Fumerate-malate exchanger

$$v_{fmT} = V_{max}^{fmT} \cdot \left(\frac{fum_{mito} \cdot mal_{cyt} - fum_{cyt} \cdot mal_{mito}}{\left(1 + \frac{fum_{mito}}{K_m^{fum_{mito}}}\right) \cdot \left(1 + \frac{mal_{cyt}}{K_m^{mal_{cyt}}}\right) + \left(1 + \frac{fum_{cyt}}{K_m^{fum_{cyt}}}\right) \cdot \left(1 + \frac{mal_{mito}}{K_m^{mal_{mito}}}\right) - 1} \right)$$

V_{max}^{fmT} for numerical value see Supplementary Table 1

$K_m^{fum_{mito}} = 0.05$ [224]

$K_m^{mal_{cyt}} = 0.23$ [225]

$K_m^{fum_{cyt}} = 0.05$ [224]

$K_m^{mal_{mito}} = 0.23$ [225]

Alanine amino transferase

$$v_{aat} = V_{max}^{aat} \cdot \left(\frac{akg_{cyt} \cdot ala_{cyt} - 1/_{K_{eq}^{aat}} \cdot glu_{cyt} \cdot pyr_{cyt}}{\left(1 + \frac{akg_{cyt}}{K_m^{akg_{cyt}}}\right) \cdot \left(1 + \frac{ala_{cyt}}{K_m^{ala_{cyt}}}\right) + \left(1 + \frac{glu_{cyt}}{K_m^{glu_{cyt}}}\right) \cdot \left(1 + \frac{pyr_{cyt}}{K_m^{pyr_{cyt}}}\right) - 1} \right)$$

V_{max}^{ass} for numerical value see Supplementary Table 1

$K_{eq}^{asl} = 1.5$ [141]

$K_m^{akg_{cyt}} = 1.1$ [226]

$K_m^{ala_{cyt}} = 34$ [226]

$K_m^{glu_{cyt}} = 15$ [226]

$K_m^{pyr_{cyt}} = 0.9$ [226]

glutamate-sodium-transporter

$$v_{gluT} = V_{max}^{gluT} \cdot \left(\frac{glu_{ext} - glu_{cyt} \cdot \frac{na_{cyt}}{na_{ext}}}{1 + \frac{glu_{ext}}{K_m^{glu_{ext}}}} \right)$$

V_{max}^{gluT} for numerical value see Supplementary Table 1

$K_m^{glu_{ext}} = 0.25$ [227]

alanin-sodium-transporter

$$v_{alaT} = V_{max}^{alnT} \cdot \left(\frac{ala_{ext} - ala_{cyt} \cdot \frac{na_{cyt}}{na_{ext}}}{ala_{ext} + \ K_m^{ala_{ext}}} \right)$$

V_{max}^{alnT} for numerical value see Supplementary Table 1

$K_m^{aln_{ext}} = 4.5$ [228]

serine-sodium-transporter

$$v_{serT} = V_{max}^{serT} \cdot \left(\frac{ser_{ext} - ser_{cyt} \cdot \frac{na_{cyt}}{na_{ext}}}{ser_{ext} + \ K_m^{ser_{ext}}} \right)$$

V_{max}^{serT} for numerical value see Supplementary Table 1

$K_m^{ser_{ext}} = 3.7$ [228]

serinedehydrogenase

$$v_{sdh} = V_{max}^{sdh} \cdot \left(\frac{ser_{cyt}}{K_m^{ser_{cyt}} + ser_{cyt}} \right)$$

V_{max}^{sdh} for numerical value see Supplementary Table 1

$K_m^{ser_{cyt}} = 57$ [229]

glutamine-sodium-transporter

$$v_{glnT} = V_{max}^{glnT} \cdot \left(\frac{gln_{ext} \cdot na_{ext} - gln_{cyt} \cdot na_{cyt}}{gln_{ext} + k_m^{gln_{ext}}} \right)$$

V_{max}^{glnT} for numerical value see Supplementary Table 1

$K_m^{gln_{ext}} = 4.5$ [228]

Glutamine synthetase

$$v_{gln-syn} = V_{max}^{gln-syn} \cdot \left(\frac{glu_{cyt}}{glu_{cyt} + K_m^{glu_{cyt}}} \right) \cdot \left(\frac{nh3_{cyt}}{nh3_{cyt} + K_m^{nh3_{cyt}}} \right) \cdot \left(\frac{atp_{cyt}}{atp_{cyt} + K_m^{atp_{cyt}}} \right)$$

$V_{max}^{gln-syn}$ for numerical value see Supplementary Table 1

$K_m^{glu_{cyt}} = 5$ [230]

$K_m^{nh3_{cyt}} = 0.3$ [230]

$K_m^{atp_{cyt}} = 1.2$ [231]

VLDL-LD synthesis

LD synthesis (tag)

$$v_{LD-syn-tag} = V_{max}^{Ld-syn-tag} \cdot \left(\frac{tag_{er}}{tag_{er} + K_m^{tag_{er}}} \right)$$

$V_{max}^{LD-syn-tag}$ for numerical value see Supplementary Table 1

$K_m^{tag_{er}} = 0.1$

LD synthesis (cholesterol ester)

$$v_{LD-syn-ce} = V_{max}^{Ld-syn-ce} \cdot \left(\frac{ce_{er}}{ce_{er} + K_m^{ce_{er}}} \right)$$

$V_{max}^{LD-syn-ce}$ for numerical value see Supplementary Table 1

$K_m^{ce_{er}} = 0.1$

ApoB synthesis

$$v_{ApoB-syn} = V_{max}^{ApoB-syn}$$

$V_{max}^{ApoB-syn}$ for numerical value see Supplementary Table 1

ApoB degradation

$$v_{ApoB-deg} = V_{max}^{ApoB-syn} \cdot \ ApoB \cdot \left(k_1 + k_2 \frac{ins_{ext}}{ins_{ext} + K_m^{ins_{ext}}} \right)$$

$V_{max}^{ApoB-syn}$ for numerical value see Supplementary Table 1

$k_1 = 0.3$ [232]

$k_2 = 0.7$ [232]

$K_m^{ins_{ext}} = 5 \cdot 10^3 pM$ [232]

Microsomal transfer protein (tag)

$$v_{MTP}^{tag} = V_{max}^{MTP} \cdot apoB \cdot \left(\frac{tag_{er}}{tag_{er} + K_m^{tag_{er}}}\right)$$

$V_{max-tag}^{MTP}$ for numerical value see Supplementary Table 1

$K_m^{tag_{er}} = 0.1$

Microsomal transfer protein (cholesterol ester)

$$v_{MTP}^{ce} = V_{max-ce}^{MTP} \cdot apoB \cdot \left(\frac{ce_{er}}{ce_{er} + K_m^{ce_{er}}}\right)$$

$V_{max-ce}^{MTP} = 0.77 \cdot V_{max}^{MTP}$ [233]

$K_m^{ce_{er}} = 0.1$

VLDL release (tag)

$$v_{VLDL-ex-tag} = V_{max}^{VLDL-ex-tag} \cdot \left(\frac{tag_{vldl}}{tag_{vldl} + K_m^{tag_{vldl}}}\right)$$

$V_{max}^{VLDL-ex-tag}$ for numerical value see Supplementary Table 1

$K_m^{tag_{vldl}} = 10$

VLDL release (cholesterol ester)

$$v_{VLDL-ex-ce} = V_{max}^{VLDL-ex-ce} \cdot \left(\frac{ce_{vldl}}{ce_{vldl} + K_m^{ce_{vldl}}}\right)$$

$V_{max}^{VLDL-ex-ce}$ for numerical value see Supplementary Table 1

$K_m^{ce_{vldl}} = 10$

ATGL [234]

$$v_{ATGL}^{tag} = V_{max-tag}^{ATGL} \cdot Sur_{ld} \cdot \gamma \cdot \left(\frac{tag_{ld}}{tag_{ld} + K_m^{tag_{ld}}}\right)$$

$V_{max-tag}^{ATGL}$ for numerical value see Supplementary Table 1

$K_m^{tag_{ld}} = 10$

$$Sur_{ld} = \left(tag_{ld} + \frac{2}{3} \cdot dag_{ld} + \frac{1}{3} \cdot mag_{ld} + \frac{2}{3} ce_{ld}\right)^{\frac{2}{3}}$$

Hormone sensitive lipase (HSL) (dag) [235]

$$v_{HSL}^{tag} = V_{max-tag}^{HSL} \cdot Sur_{ld} \cdot \gamma \cdot \left(\frac{dag_{ld}}{dag_{ld} + K_m^{dag_{ld}}}\right)$$

$V_{max-tag}^{HSL}$ for numerical value see Supplementary Table 1

$K_m^{dag_{ld}} = 10$

$$Sur_{ld} = \left(tag_{ld} + \frac{2}{3} \cdot dag_{ld} + \frac{1}{3} \cdot mag_{ld} + \frac{2}{3} ce_{ld}\right)^{\frac{2}{3}}$$

Monoaclyglycerol lipase

$$v_{magl} = V_{max}^{magl} \cdot Sur_{ld} \cdot \left(\frac{mag_{ld}}{mag_{ld} + K_m^{mag_{ld}}}\right)$$

V_{max}^{magl} for numerical value see Supplementary Table 1

$K_m^{mag_{ld}} = 0.51$ [236]

$$Sur_{ld} = \left(tag_{ld} + \frac{2}{3} \cdot dag_{ld} + \frac{1}{3} \cdot mag_{ld} + \frac{2}{3} ce_{ld}\right)^{\frac{2}{3}}$$

Cholesterol ester esterase

$$v_{cee} = V_{max}^{cee} \cdot Sur_{ld} \cdot \gamma \cdot \left(\frac{ce_{ld}}{ce_{ld} + K_m^{ce_{ld}}}\right)$$

V_{max}^{cee} for numerical value see Supplementary Table 1

$K_m^{ce_{ld}} = 5$

$$Sur_{ld} = \left(tag_{ld} + \frac{2}{3} \cdot dag_{ld} + \frac{1}{3} \cdot mag_{ld} + \frac{2}{3} ce_{ld}\right)^{\frac{2}{3}}$$

Ketone body synthesis

HMG-CoA synthase

$$v_{hmg-syn} = V_{max}^{hmg-syn} \cdot \left(\frac{acoa_{mito}}{acoa_{mito} + K_m^{acoa_{mito}} \cdot \left(1 + \frac{kc4coa_{mito}}{K_i^{kc4coa_{mito}}}\right)} \right) \cdot \left(\frac{kc4coa_{mito}}{kc4coa_{mito} + K_m^{kc4coa_{mito}}} \right)$$

$V_{max}^{hmg-syn}$ for numerical value see Supplementary Table 1

$K_m^{acoa_{mito}} = 0.029$ [237]

$K_i^{kc4coa_{mito}} = 0.01$ [238]

$K_m^{kc4coa_{mito}} = 0.00035$ [238]

HMG-CoA lyase

$$v_{hmg-lys} = V_{max}^{hmg-lys} \cdot \left(\frac{hmgcoa_{mito}}{hmgcoa_{mito} + K_m^{hmgcoa_{mito}}} \right)$$

$V_{max}^{hmg-syn}$ for numerical value see Supplementary Table 1

$K_m^{hmgcoa_{mito}} = 0.048$ [239]

B-Hydroxy butyrate dehydrogenase

$$v_{\beta hdh} = V_{max}^{\beta hdh} \cdot \left(\frac{acac_{mito} \cdot nadh_{mito} - {1}/{K_{eq}^{\beta hdh}} \cdot bhbut_{mito} \cdot nad_{mito}}{\left(1 + \frac{acac_{mito}}{K_{m_{mito}}^{acac}}\right) \cdot \left(1 + \frac{nadh_{mito}}{K_m^{nadh_{mito}}}\right) + \left(1 + \frac{bhbut_{mito}}{K_m^{bhbut_{mito}}}\right) \cdot \left(1 + \frac{nad_{mito}}{K_m^{nad_{mito}}}\right) - 1} \right)$$

$V_{max}^{\beta hdh}$ for numerical value see Supplementary Table 1

$K_{eq}^{\beta hdh} = 20.3 \cdot \frac{h_{mito}}{h_{cyt}}$ [119]

$K_m^{acac_{mito}} = 0.204$ [240]

$$K_m^{nadh_{mito}} = K_0^{nadh_{mito}} \cdot \left(1 + \frac{nad_{mito}}{K_i^{nad_{mito}}}\right)$$

$K_0^{nadh_{mito}} = 0.017$ [240]

$K_i^{nad_{mito}} = 0.121$ [240]

$K_m^{hbut_{mito}} = 1.604$ [240]

$$K_m^{nad_{mito}} = K_0^{nad_{mito}} \cdot \left(1 + \frac{nadh_{mito}}{K_i^{nadh_{mito}}}\right)$$

$K_0^{nad_{mito}} = 0.067$ [240]

$K_i^{nadh_{mito}} = 0.066$ [240]

Acetoacetate transport (mitochondrial)

$$v_{acacT} = V_{max}^{acacT} \cdot \left(\frac{acac_{mito}}{acac_{mito} + K_m^{acac_{mito}}}\right)$$

V_{max}^{acacT} for numerical value see Supplementary Table 1

$K_m^{acac_{mito}} = 0.56$ [241]

B-Hydroxy butyrate transport (mitochondrial)

$$v_{\beta hbT} = V_{max}^{\beta hbT} \cdot \left(\frac{bhbut_{mito}}{bhbut_{mito} + K_m^{bhbut_{mito}}}\right)$$

$V_{max}^{\beta hbT}$for numerical value see Supplementary Table 1

$K_m^{bhbut_{mito}} = 5.6$ [241]

Acetoacetate export (MCT1/MCT2)

$$v_{acac-ex} = V_{max}^{acac-ex} \cdot \left(\frac{acac_{ext} - acac_{cyt}}{1 + \frac{acac_{cyt}}{K_m^{acac_{cyt}}} + \frac{acac_{ext}}{K_m^{acac_{ext}}}}\right)$$

$V_{max}^{acac-ex}$ for numerical value see Supplementary Table 1

$K_m^{acac_{cyt}} = 1.2$ [242]

$K_m^{acac_{ext}} = 1.2$ [242]

B-Hydroxy butyrate export (MCT1/MCT2)

$$v_{\beta hb-ex} = V_{max}^{\beta hb-ex} \cdot \left(\frac{bhbut_{ext} - bhbut_{cyt}}{1 + \frac{bhbut_{cyt}}{K_m^{bhbut_{cyt}}} + \frac{bhbut_{ext}}{K_m^{bhbut_{ext}}}}\right)$$

$V_{max}^{\beta hb-ex}$ for numerical value see Supplementary Table 1

$K_m^{bhbut_{cyt}} = 0.8$ [242]

$K_m^{bhbut_{ext}} = 0.8$ [242]

Cholesterol synthesis

Acetoacetyl-CoA synthetase

$$v_{acaccoa-syn} = V_{max}^{acaccoa-syn} \cdot \left(\frac{atp_{cyt}}{atp_{cyt} + K_m^{atp_{cyt}}} \right) \cdot \left(\frac{acac_{cyt}}{acac_{cyt} + K_m^{acac_{cyt}}} \right) \cdot \left(\frac{coa_{cyt}}{coa_{cyt} + K_m^{coa_{cyt}}} \right)$$

$V_{max}^{acaccoa-syn}$ for numerical value see Supplementary Table 1

$K_m^{ATP_{cyt}}$ =0.06 [243]

$K_m^{acac_{cyt}}$ =0.008 [243]

$K_m^{coa_{cyt}}$ =0.01 [243]

Thiolase (cytosolic, peroxisomal)

$$v_{3kt-cyt}^{kc4coa} = V_{max-cyt}^{3kt-kc4_{coa}} \cdot \left(\frac{coa_{cyt} \cdot kc4coa_{cyt} - 1/K_{eq}^{3kt-cyt} \cdot acoa_{cyt}^2}{\left(1 + \frac{coa_{cyt}}{K_m^{coa_{cyt}}}\right) \cdot \left(1 + \frac{kc4coa_{cyt}}{K_m^{kc4coa_{cyt}}}\right) + \left(1 + \frac{acoa_{cyt}}{K_m^{acoa_{cyt}}}\right)^2 - 1} \right)$$

$V_{max-cyt}^{3kt-kc4}$ for numerical value see Supplementary Table 1

$K_{eq}^{3kt-cyt} = 7800$ [244]

$K_m^{coa_{cyt}}$ =0.015 [244]

$$K_m^{kc4coa_{cyt}} = K_0^{kc4coa_{cyt}} \cdot \left(1 + \frac{coa_{cyt}}{K_i^{coa_{cyt}}}\right)$$

$K_i^{coa_{cyt}} = 0.073$ [244]

$K_0^{kc4coa_{cyt}} = 0.033$ [244]

$K_m^{acoa_{mito}} = 0.115$ [244]

Cytosolic HMG-CoA synthase

$$v_{hmg-syn-cyt} = V_{max}^{hmg-syn-cyt} \cdot \left(\frac{acoa_{cyt}}{acoa_{cyt} + K_m^{acoa_{cyt}} \cdot \left(1 + \frac{kc4coa_{cyt}}{K_i^{kc4coa_{cyt}}}\right)} \right) \cdot \left(\frac{kc4coa_{cyt}}{kc4coa_{cyt} + K_m^{kc4coa_{cyt}}} \right) \cdot \frac{1}{1 + \frac{hmgcoa_{cyt}}{K_i^{hmgcoa_{cyt}}}}$$

$V_{max}^{hmg-syn-cyt}$ for numerical value see Supplementary Table 1

$K_m^{acoa_{cyt}} = 0.076$ [245]

$K_i^{kc4coa_{cyt}} = 0.03$ [246]

$K_m^{kc4coa_{cyt}} = 0.0025 \cdot 10^{-5}$ [246]

$K_i^{hmgcoa_{cyt}} = 0.0011$ [245]

HMG-CoA lyase

$$v_{hmgl} = V_{max}^{hmgl} \cdot \left(\frac{hmgcoa_{cyt}}{hmgcoa_{cyt} + K_m^{hmgcoa_{cyt}}} \right)$$

V_{max}^{hmgl} for numerical value see Supplementary Table 1

$K_m^{hmgcoa_{cyt}} = 0.028$ [247]

HMG-CoA reductase

$$v_{hmgr} = V_{max}^{hmgr} \cdot \left(\frac{hmgcoa_{cyt}}{hmgcoa_{cyt} + K_m^{hmgcoa_{cyt}}} \right) \cdot \left(\frac{nadph_{cyt}}{nadph_{cyt} + K_m^{nadph_{cyt}}} \right)^2$$

$V_{max}^{hmgr} = V_0^{hmgr} \cdot (1 - \gamma_{hmgr})$

V_0^{hmgr} for numerical value see Supplementary Table 1

$\gamma_{hmgr} = \gamma(K_{0.5}^{ins}, K_{0.5}^{glucagon}, n^{glucagon})$

$K_{0.5}^{ins} = 200pM$

$K_{0.5}^{glucagon} = 300pM$

$n^{glucagon} = 3.6$

$K_m^{hmgcoa_{cyt}} = 0.004$ [248]

$K_m^{nadph_{cyt}} = 0.087$ [249]

Mevalonate kinase

$$v_{mevk} = V_{max}^{mevk} \cdot \left(\frac{mev_{cyt}}{mev_{cyt} + K_m^{mev_{cyt}}} \right) \cdot \left(\frac{atp_{cyt}}{atp_{cyt} + K_m^{atp_{cyt}}} \right)$$

V_{max}^{mevk} for numerical value see Supplementary Table 1

$K_m^{mev_{cyt}} = 0.271$ [250]

$$K_m^{atp_{cyt}} = K_0^{atp_{cyt}} \cdot \left(1 + \frac{fpp_{cyt}}{K_i^{fpp_{cyt}}} \right) \cdot \left(1 + \frac{gpp_{cyt}}{K_i^{gpp_{cyt}}} \right)$$

$K_0^{atp_{cyt}} = 1.75$ [250]

$K_i^{fpp_{cyt}} = 0.0025$ [250]

$K_i^{gpp_{cyt}} = 0.005$ [251]

Phopshomevalonate kinase

$$v_{pmevk} = V_{max}^{mevpk} \cdot \left(\frac{mev5p_{cyt}}{mev5p_{cyt} + K_m^{mev5p_{cyt}}} \right) \cdot \left(\frac{atp_{cyt}}{atp_{cyt} + K_m^{atp_{cyt}}} \right)$$

V_{max}^{mevpk} for numerical value see Supplementary Table 1

$K_m^{mev5p_{cyt}} = 0.075$ [252]

$K_m^{atp_{cyt}} = 0.46$ [252]

Mevalonate-5-pyrophopshatedecarboxylase

$$v_{mdp} = V_{max}^{mdp} \cdot \left(\frac{mev5pp_{cyt}}{mev5pp_{cyt} + K_m^{mev5pp_{cyt}}} \right) \cdot \left(\frac{atp_{cyt}}{atp_{cyt} + K_m^{atp_{cyt}}} \right)$$

V_{max}^{mdp} for numerical value see Supplementary Table 1

$K_m^{mev5pp_{cyt}} = 0.02$ [253]

$$K_m^{atp_{cyt}} = K_0^{atp_{cyt}} \cdot \left(1 + \frac{gpp_{cyt}}{K_i^{gpp_{cyt}}} \right)$$

$K_0^{atp_{cyt}} = 0.53$ [254]

$K_i^{gpp_{cyt}} = 0.065$ [254]

Isopentenylphyrophosphate isomerase

$$v_{ippi} = V_{max}^{ippi} \cdot \left(\frac{ipp_{cyt} - \frac{1}{K_{eq}^{ippi}} \cdot dmpp_{cyt}}{\left(1 + \frac{ipp_{cyt}}{K_m^{ipp_{cyt}}} + \frac{dmpp_{cyt}}{K_m^{dmpp_{cyt}}}\right)} \right)$$

V_{max}^{ippi} for numerical value see Supplementary Table 1

$K_{eq}^{ippi} = 9$ [255]

$K_m^{ipp_{cyt}} = 0.0082$ [256]

$K_m^{dmpp_{cyt}} = 0.017$ [257]

Geranyl pyrophosphate synthase

$$v_{gpps} = V_{max}^{gpps} \cdot \left(\frac{ipp_{cyt}}{ipp_{cyt} + K_m^{ipp_{cyt}}} \right) \cdot \left(\frac{dmpp_{cyt}}{dmpp_{cyt} + K_m^{dmpp_{cyt}}} \right)$$

V_{max}^{gpps} for numerical value see Supplementary Table 1

$K_m^{ipp_{cyt}} = 0.00125$ [258]

$K_m^{dmpp_{cyt}} = 0.0022$ [258]

Farnesyl pyrophosphate synthase

$$v_{fpps} = V_{max}^{fpps} \cdot \left(\frac{ipp_{cyt}}{ipp_{cyt} + K_m^{ipp_{cyt}}} \right) \cdot \left(\frac{gpp_{cyt}}{gpp_{cyt} + K_m^{gpp_{cyt}}} \right)$$

V_{max}^{gpps} for numerical value see Supplementary Table 1

$K_m^{ipp_{cyt}} = 0.004$ [259]

$K_m^{gpp_{cyt}} = 0.002$ [259]

Squalene synthase

$$v_{sqs-nadph} = V_{max}^{sqs-nadph} \cdot \left(\frac{fpp_{cyt}}{fpp_{cyt} + K_m^{fpp_{cyt}}} \right)^2 \cdot \left(\frac{nadph_{cyt}}{nadph_{cyt} + K_m^{nadph_{cyt}}} \right)$$

$V_{max}^{sqs-nadph}$ for numerical value see Supplementary Table 1

$K_m^{fpp_{cyt}} = 0.001$ [260]

$K_m^{nadph_{cyt}} = 0.04$ [260]

$$v_{sqs-nadh} = V_{max}^{sqs-nadh} \cdot \left(\frac{fpp_{cyt}}{fpp_{cyt} + K_m^{fpp_{cyt}}} \right)^2 \cdot \left(\frac{nadh_{cyt}}{nadh_{cyt} + K_m^{nadh_{cyt}}} \right)$$

$V_{max}^{sqs-nadh}$ for numerical value see Supplementary Table 1

$K_m^{fpp_{cyt}} = 0.001$ [260]

$K_m^{nadh_{cyt}} = 0.8$ [260]

Squalene epoxidase

$$v_{sqe} = V_{max}^{sqe} \cdot \left(\frac{squ_{cyt}}{squ_{cyt} + K_m^{squ_{cyt}}} \right) \cdot \left(\frac{nadph_{cyt}}{nadph_{cyt} + K_m^{nadph_{cyt}}} \right) \cdot \left(\frac{o2_{cyt}}{o2_{cyt} + K_m^{o2_{cyt}}} \right)$$

V_{max}^{sqe} for numerical value see Supplementary Table 1

$K_m^{squ_{cyt}} = 0.0038$ [261] [262]

$K_m^{nadph_{cyt}} = 0.11$ [262]

$K_m^{o2_{cyt}} = 0.0043$ [263]

Squalene epoxide cyclase

$$v_{osc} = V_{max}^{osc} \cdot \left(\frac{sqe_{cyt}}{sqe_{cyt} + K_m^{sqe_{cyt}}} \right)$$

V_{max}^{osc} for numerical value see Supplementary Table 1

$K_m^{sqe_{cyt}} = 0.055$ [264]

Sterol 14-Demethylase P450 (CYP51)

$$v_{s14dm-lan} = V_{max}^{s14dm-lan} \cdot \left(\frac{lan_{er}}{lan_{er} + K_m^{lan_{er}}} \right) \cdot \left(\frac{nadph_{cyt}}{nadph_{cyt} + K_m^{nadph_{cyt}}} \right) \cdot \left(\frac{o2_{cyt}}{o2_{cyt} + K_m^{o2_{cyt}}} \right)$$

$V_{max}^{s14dm-lan}$ for numerical value see Supplementary Table 1

$K_m^{lan_{er}} = 0.0105$ [265]

$K_m^{nadph_{cyt}} = 0.006$ [266]

$K_m^{o2_{cyt}} = 0.0068$ [267]

$$v_{s14dm-dihlan} = V_{max}^{s14dm-dihlan} \cdot \left(\frac{dihlan_{er}}{dihlan_{er} + K_m^{dihlan_{er}}} \right) \cdot \left(\frac{nadph_{cyt}}{nadph_{cyt} + K_m^{nadph_{cyt}}} \right) \cdot \left(\frac{o2_{cyt}}{o2_{cyt} + K_m^{o2_{cyt}}} \right)$$

$V_{max}^{s14dm-dihlan}$ for numerical value see Supplementary Table 1

$K_m^{dihlan_{er}} = 0.02$ [265]

$K_m^{nadph_{cyt}} = 0.006$ [266]

$K_m^{o2_{cyt}} = 0.0068$ [267]

Sterol Δ14 reductase

$$v_{s14r-ffmas} = V_{max}^{s14r-ffmas} \cdot \left(\frac{ffmas_{er}}{ffmas_{er} + K_m^{ffmas_{er}}} \right) \cdot \left(\frac{nadph_{cyt}}{nadph_{cyt} + K_m^{nadph_{cyt}}} \right)$$

$V_{max}^{s14r-ffmas}$ for numerical value see Supplementary Table 1

$K_m^{ffmas_{er}} = 0.03$

$K_m^{nadph_{cyt}} = 0.6$ [268]

$$v_{s14r-dihffmas} = V_{max}^{s14r-dihffmas} \cdot \left(\frac{dihffmas_{er}}{dihffmas_{er} + K_m^{dihffmas_{er}}} \right) \cdot \left(\frac{nadph_{cyt}}{nadph_{cyt} + K_m^{nadph_{cyt}}} \right)$$

$V_{max}^{s14r-dihffmas}$ for numerical value see Supplementary Table 1

$K_m^{dihffmas_{er}} = 0.00885$ [269]

$K_m^{nadph_{cyt}} = 0.6$ [268]

Methyl sterol oxidase (Methylsterol monooxygenase)

$$v_{mso-tmas} = V_{max}^{mso-tmas} \cdot \left(\frac{tmas_{er}}{tmas_{er} + K_m^{tmas_{er}}} \right) \cdot \left(\frac{nadph_{cyt}}{nadph_{cyt} + K_m^{nadph_{cyt}}} \right) \cdot \left(\frac{o2_{cyt}}{o2_{cyt} + K_m^{o2_{cyt}}} \right)$$

$V_{max}^{mso-tmas}$ for numerical value see Supplementary Table 1

$K_m^{tmas_{er}} = 0.1$ [270] ($\mathrm{K_m^{dimethylsterol}}$)

$K_m^{nadph_{cyt}} = 0.006$ [266]

$K_m^{o2_{cyt}} = 0.0068$ [267]

$$v_{mso-dmtmas} = V_{max}^{mso-dmtmas} \cdot \left(\frac{dmtmas_{er}}{dmtmas_{er} + K_m^{dmtmas_{er}}} \right) \cdot \left(\frac{nadph_{cyt}}{nadph_{cyt} + K_m^{nadph_{cyt}}} \right) \cdot \left(\frac{o2_{cyt}}{o2_{cyt} + K_m^{o2_{cyt}}} \right)$$

$V_{max}^{mso-dmtmas}$ for numerical value see Supplementary Table 1

$K_m^{dmtmas_{er}} = 0.1$ [270] $(K_m^{dimethylsterol})$

$K_m^{nadph_{cyt}} = 0.006$ [266]

$K_m^{o2_{cyt}} = 0.0068$ [267]

$$v_{mso-dihtmas} = V_{max}^{mso-dihtmas} \cdot \left(\frac{dihtmas_{er}}{dihtmas_{er} + K_m^{dihtmas_{er}}} \right) \cdot \left(\frac{nadph_{cyt}}{nadph_{cyt} + K_m^{nadph_{cyt}}} \right) \cdot \left(\frac{o2_{cyt}}{o2_{cyt} + K_m^{o2_{cyt}}} \right)$$

$V_{max}^{mso-dihtmas}$ for numerical value see Supplementary Table 1

$K_m^{dihtmas_{er}} = 0.1$ [270] $(K_m^{dimethylsterol})$

$K_m^{nadph_{cyt}} = 0.006$ [266]

$K_m^{o2_{cyt}} = 0.0068$ [267]

$$v_{mso-dmdihtmas} = V_{max}^{mso-dmdihtmas} \cdot \left(\frac{dmdihtmas_{er}}{dmdihtmas_{er} + K_m^{dihtmas_{er}}} \right) \cdot \left(\frac{nadph_{cyt}}{nadph_{cyt} + K_m^{nadph_{cyt}}} \right) \cdot \left(\frac{o2_{cyt}}{o2_{cyt} + K_m^{o2_{cyt}}} \right)$$

$V_{max}^{mso-dmdihtmas}$ for numerical value see Supplementary Table 1

$K_m^{dmdihtmas_{er}} = 0.1$ [270] $(K_m^{dimethylsterol})$

$K_m^{nadph_{cyt}} = 0.006$ [266]

$K_m^{o2_{cyt}} = 0.0068$ [267]

4α-carboxylic acid sterol decarboxylase

$$v_{casdc} = V_{max}^{casdc-dmtmasca} \cdot \left(\frac{dmtmasca_{er}}{dmtmasca_{er} + K_m^{dmtmasca_{er}}} \right) \cdot \left(\frac{nad_{cyt}}{nad_{cyt} + K_m^{nad_{cyt}}} \right)$$

$V_{max}^{ccasdc-dmtmasca}$ for numerical value see Supplementary Table 1

$K_m^{dmtmasca_{er}} = 0.007$ [271]

$K_m^{nad_{cyt}} = 0.3$ [272]

$$v_{casdc} = V_{max}^{ccasdc-ddimtmasca} \cdot \left(\frac{ddimtmasca_{er}}{ddimtmasca_{er} + K_m^{ddimtmasca_{er}}} \right) \cdot \left(\frac{nad_{cyt}}{nad_{cyt} + K_m^{nad_{cyt}}} \right)$$

$V_{max}^{ccasdc-ddimtmasca}$ for numerical value see Supplementary Table 1

$K_m^{ddimtmasca_{er}} = 0.007$ [271]

$K_m^{nad_{cyt}} = 0.3$ [272]

$$v_{casdc} = V_{max}^{ccasdc-dmdihtmasca} \cdot \left(\frac{dmdihtmasca_{er}}{dmdihtmasca_{er} + K_m^{dmdihtmasca_{er}}} \right) \cdot \left(\frac{nad_{cyt}}{nad_{cyt} + K_m^{nad_{cyt}}} \right)$$

$V_{max}^{ccasdc-dmdihtmasca}$ for numerical value see Supplementary Table 1

$K_m^{dmdihtmasca_{er}} = 0.007$ [271]

$K_m^{nad_{cyt}} = 0.3$ [272]

$$v_{casdc} = V_{max}^{ccasdc-ddimdihtmasca} \cdot \left(\frac{ddimdihtmasca_{er}}{ddimdihtmasca_{er} + K_m^{ddimdihtmasca_{er}}} \right) \cdot \left(\frac{nad_{cyt}}{nad_{cyt} + K_m^{nad_{cyt}}} \right)$$

$V_{max}^{ccasdc-ddimdihtmasca}$ for numerical value see Supplementary Table 1

$K_m^{ddimdihtmasca_{er}} = 0.007$ [271]

$K_m^{nad_{cyt}} = 0.3$ [272]

3-keto steroid reductase

$$v_{3ksr} = V_{max}^{3ksr-dmktmas} \cdot \left(\frac{dmktmas_{er}}{dmktmas_{er} + K_m^{dmktmas_{er}}} \right) \cdot \left(\frac{nadph_{cyt}}{nadph_{cyt} + K_m^{nadph_{cyt}}} \right)$$

$V_{max}^{3ksr-dmktmas}$ for numerical value see Supplementary Table 1

$K_m^{dmktmas_{er}} = 0.078$ (4,4-gem-dimethyl-3-ketosteroid) [273]

$K_m^{nadph_{cyt}} = 0.00012$ [274]

$$v_{3ksr} = V_{max}^{3ksr-ddimktmas} \cdot \left(\frac{ddimktmas_{er}}{ddimktmas_{er} + K_m^{ddimktmas_{er}}}\right) \cdot \left(\frac{nadph_{cyt}}{nadph_{cyt} + K_m^{nadph_{cyt}}}\right)$$

$V_{max}^{3ksr-ddimktmas}$ for numerical value see Supplementary Table 1

$K_m^{ddimktmas_{er}} = 0.078$ (4,4-gem-dimethyl-3-ketosteroid) [273]

$K_m^{nadph_{cyt}} = 0.00012$ [274]

$$v_{3ksr} = V_{max}^{3ksr-dmkdihtmas} \cdot \left(\frac{dmkdihtmas_{er}}{dmkdihtmas_{er} + K_m^{dmkdihtmas_{er}}}\right) \cdot \left(\frac{nadph_{cyt}}{nadph_{cyt} + K_m^{nadph_{cyt}}}\right)$$

$V_{max}^{3ksr-dmkdihtmas}$ for numerical value see Supplementary Table 1

$K_m^{dmkdihtmas_{er}} = 0.078$ (4,4-gem-dimethyl-3-ketosteroid) [273]

$K_m^{nadph_{cyt}} = 0.00012$ [274]

$$v_{3ksr} = V_{max}^{3ksr-ddimkdihtmas} \cdot \left(\frac{ddimkdihtmas_{er}}{ddimkdihtmas_{er} + K_m^{ddimkdihtmas_{er}}}\right) \cdot \left(\frac{nadph_{cyt}}{nadph_{cyt} + K_m^{nadph_{cyt}}}\right)$$

$V_{max}^{3ksr-ddimkdihtmas}$ for numerical value see Supplementary Table 1

$K_m^{ddimkdihtmas_{er}} = 0.078$ (4,4-gem-dimethyl-3-ketosteroid) [273]

$K_m^{nadph_{cyt}} = 0.00012$ [274]

Steroid7,8 isomerase (cholesterol Δ isomerase)

$$v_{s78i-zym} = V_{max}^{s78i-zym} \cdot \left(\frac{zym_{er} - 1/K_{eq}^{s78i} \cdot dhlath_{er}}{zym_{er} + K_m^{zym_{er}}}\right)$$

$V_{max}^{s78i-zym}$ for numerical value see Supplementary Table 1

$K_{eq}^{s78i} = 19$ [275]

$K_m^{zym_{er}} = 0.052$ [276]

$$v_{s78i-zymostenol} = V_{max}^{s78i-zymostenol} \cdot \left(\frac{zymostenol_{er} - 1/K_{eq}^{s78i} \cdot lath_{er}}{zymostenol_{er} + K_m^{zymostenol_{er}}} \right)$$

$V_{max}^{s78i-zymostenol}$ for numerical value see Supplementary Table 1

$K_m^{zymosternol_{er}} = 0.05$

Lathosterol oxidase (assumed to be member of cytochrome P450 superfamily) [277]

$$v_{lathox-lath} = V_{max}^{lathox-lath} \cdot \left(\frac{lath_{er}}{lath_{er} + K_m^{lath_{er}}} \right) \cdot \left(\frac{nadph_{cyt}}{nadph_{cyt} + K_m^{nadph_{cyt}}} \right) \cdot \left(\frac{o2_{cyt}}{o2_{cyt} + K_m^{o2_{cyt}}} \right)$$

$V_{max}^{lathox-lath}$ for numerical value see Supplementary Table 1

$K_m^{lath_{er}} = 0.0357$ [277]

$K_m^{nadph_{cyt}} = 0.006$ [266]

$K_m^{o2_{cyt}} = 0.0068$ [267]

$$v_{lathox-dhlath} = V_{max}^{lathox-dhlath} \cdot \left(\frac{dhlath_{er}}{dhlath_{er} + K_m^{dhlath_{er}}} \right) \cdot \left(\frac{nadph_{cyt}}{nadph_{cyt} + K_m^{nadph_{cyt}}} \right) \cdot \left(\frac{o2_{cyt}}{o2_{cyt} + K_m^{o2_{cyt}}} \right)$$

$V_{max}^{lathox-dhlath}$ for numerical value see Supplementary Table 1

$K_m^{dhlath_{er}} = 0.0357$ [277]

$K_m^{nadph_{cyt}} = 0.006$ [266]

$K_m^{o2_{cyt}} = 0.0068$ [267]

Sterol Δ7 reductase (cytochrome P450 oxidoreductase)

$$v_{s7r-dhchol} = V_{max}^{s7r-dhchol} \cdot \left(\frac{dhchol_{er}}{dhchol_{er} + K_m^{dhchol_{er}}} \right) \cdot \left(\frac{nadph_{cyt}}{nadph_{cyt} + K_m^{nadph_{cyt}}} \right)$$

$V_{max}^{s7r-dhchol}$ for numerical value see Supplementary Table 1

$K_m^{dhchol_{er}} = 0.03$ [278]

$K_m^{nadph_{cyt}} = 0.006$ [266]

$$v_{s7r-dhdesm} = V_{max}^{s7r-dhdesm} \cdot \left(\frac{desmo_{er}}{desmo_{er} + K_m^{desmo_{er}}} \right) \cdot \left(\frac{nadph_{cyt}}{nadph_{cyt} + K_m^{nadph_{cyt}}} \right)$$

$V_{max}^{s7r-dhdesmo}$ for numerical value see Supplementary Table 1

$K_m^{desmo_{er}} = 0.03$ [278]

$K_m^{nadph_{cyt}} = 0.006$ [266]

Sterol Δ24 reductase

$$v_{s24r-lan} = V_{max}^{s24r-lan} \cdot \left(\frac{lan_{er}}{lan_{er} + K_m^{lan_{er}}} \right) \cdot \left(\frac{nadph_{cyt}}{nadph_{cyt} + K_m^{nadph_{cyt}}} \right)$$

$V_{max}^{s24r-lan}$ for numerical value see Supplementary Table 1

$K_m^{lan_{er}} = 0.109$ [279]

$K_m^{nadph_{cyt}} = 0.01$

$$v_{s24r-ffmas} = V_{max}^{s24r-ffmas} \cdot \left(\frac{ffmas_{er}}{ffmas_{er} + K_m^{ffmas_{er}}} \right) \cdot \left(\frac{nadph_{cyt}}{nadph_{cyt} + K_m^{nadph_{cyt}}} \right)$$

$V_{max}^{s24r-ffmas}$ for numerical value see Supplementary Table 1

$K_m^{ffmas_{er}} = 0.1$

$K_m^{nadph_{cyt}} = 0.01$

$$v_{s24r-tmas} = V_{max}^{s24r-tmas} \cdot \left(\frac{tmas_{er}}{tmas_{er} + K_m^{tmas_{er}}} \right) \cdot \left(\frac{nadph_{cyt}}{nadph_{cyt} + K_m^{nadph_{cyt}}} \right)$$

$V_{max}^{s24r-tmas}$ for numerical value see Supplementary Table 1

$K_m^{tmas_{er}} = 0.1$

$K_m^{nadph_{cyt}} = 0.01$

$$v_{s24r-zym} = V_{max}^{s24r-zym} \cdot \left(\frac{zym_{er}}{zym_{er} + K_m^{zym_{er}}} \right) \cdot \left(\frac{nadph_{cyt}}{nadph_{cyt} + K_m^{nadph_{cyt}}} \right)$$

$V_{max}^{s24r-zym}$ for numerical value see Supplementary Table 1

$K_m^{zym_{er}} = 0.176$ [279]

$K_m^{nadph_{cyt}} = 0.01$

$$v_{s24r-dhlath} = V_{max}^{s24r-dhlath} \cdot \left(\frac{dhlath_{er}}{dhlath_{er} + K_m^{dhlath_{er}}}\right) \cdot \left(\frac{nadph_{cyt}}{nadph_{cyt} + K_m^{nadph_{cyt}}}\right)$$

$V_{max}^{s24r-dhlath}$ for numerical value see Supplementary Table 1

$K_m^{dhlath_{cyt}} = 0.1$

$K_m^{nadph_{cyt}} = 0.01$

$$v_{s24r-dhdesmo} = V_{max}^{s24r-dhdesmo} \cdot \left(\frac{dhdesmo_{er}}{dhdesmo_{er} + K_m^{dhdesmo_{er}}}\right) \cdot \left(\frac{nadph_{cyt}}{nadph_{cyt} + K_m^{nadph_{cyt}}}\right)$$

$V_{max}^{s24r-dhdesmo}$ for numerical value see Supplementary Table 1

$K_m^{dhdesmo_{cyt}} = 0.037$ [279]

$K_m^{nadph_{cyt}} = 0.01$

$$v_{s24r-desmo} = V_{max}^{s24r-desmo} \cdot \left(\frac{desmo_{er}}{desmo_{er} + K_m^{desmo_{er}}}\right) \cdot \left(\frac{nadph_{cyt}}{nadph_{cyt} + K_m^{nadph_{cyt}}}\right)$$

$V_{max}^{s24r-desmo}$ for numerical value see Supplementary Table 1

$K_m^{desmo_{er}} = 0.163$ [279]

$K_m^{nadph_{cyt}} = 0.01$

Acyl-CoA cholesterol Acyltransferase

$$v_{acat} = V_{max}^{acat} \cdot \left(\frac{c16coa_{cyt}}{c16coa_{cyt} + K_m^{c16coa_{cyt}}}\right) \cdot \left(\frac{chol_{er}}{chol_{er} + K_m^{chol_{er}}}\right)$$

V_{max}^{acat} for numerical value see Supplementary Table 1

$K_m^{c16coa_{cyt}} = 0.038$ [280]

$K_m^{chol_{er}} = 0.101$ [281]

Alcohol metabolism

Ethanol uptake

$$v_{ethT} = V_{max}^{ethT} \cdot \left(ethanol_{ext} - ethanol_{cyt} \right)$$

V_{max}^{ethT} for numerical value see Supplementary Table 1

Alcohol dehydrogenase

$$v_{adh} = V_{max}^{adh} \cdot \left(1 + \frac{gra_{cyt}}{gra_{cyt} + K_a^{gra_{cyt}}}\right) \cdot \left(\frac{ethanol_{cyt}}{ethanol_{cyt} + K_m^{ethanol_{cyt}}}\right) \cdot \left(\frac{nad_{cyt}}{nad_{cyt} + K_m^{nad_{cyt}} \cdot \left(1 + \frac{nadh_{cyt}}{K_i^{nadh_{cyt}}}\right)}\right)$$

V_{max}^{adh} for numerical value see Supplementary Table 1

$K_a^{gra_{cyt}} = 0.001$

$$K_m^{ethanol_{cyt}} = \left(K_0^{ethanol_{cyt}} + \frac{ethanol_{cyt}^2}{K_i^{ethanol_{cyt}}}\right)$$

$K_0^{ethanol_{cyt}} = 0.489$ [282]

$K_i^{ethanol_{cyt}} = 170$ [282]

$K_m^{nad_{cyt}} = 0.033$ [282]

$K_i^{nadh_{cyt}} = 0.0006$ [282]

Aldehyde dehydrogenase I

$$v_{alddhI} = V_{max}^{alddhI} \cdot \left(\frac{aald_{cyt}}{aald_{cyt} + K_m^{aald_{cyt}}}\right) \cdot \left(\frac{nad_{cyt}}{nad_{cyt} + K_m^{nad_{cyt}}}\right)$$

V_{max}^{alddhI} for numerical value see Supplementary Table 1

$K_m^{aald_{cyt}} = 1$ [283]

$K_m^{nad_{cyt}} = 0.02$ [283]

Aldehyde dehydrogenase II

$$v_{alddhII} = V_{max}^{alddhII} \cdot \left(\frac{aald_{cyt}}{aald_{cyt} + K_m^{aald_{cyt}}} \right) \cdot \left(\frac{nad_{cyt}}{nad_{cyt} + K_m^{nad_{cyt}}} \right)$$

$V_{max}^{alddhII} = \frac{0.7}{1.1} \cdot V_{max}^{alddhI}$ [283]

$K_m^{aald_{cyt}} = 0.7$ [283]

$K_m^{nad_{cyt}} = 0.04$ [283]

Acetaldehyde transport

$$v_{aaldT} = V_{max}^{aaldT} \cdot (aald_{cyt} - aald_{mito})$$

V_{max}^{aaldT} for numerical value see Supplementary Table 1

Mitochondrial Aldehyde dehydrogenase

$$v_{alddh_{mito}} = V_{max}^{alddh_{mito}} \cdot \left(\frac{aald_{mito}}{aald_{mito} + K_m^{aald_{mito}}} \right) \cdot \left(\frac{nad_{mito}}{nad_{mito} + K_m^{nad_{mito}}} \right)$$

$V_{max}^{alddh_{mito}}$ for numerical value see Supplementary Table 1

$K_m^{aald_{mito}} = 0.0004$ [284]

$K_m^{nad_{mito}} = 0.02$ [284]

Acetate transport

$$v_{aceT} = V_{max}^{aceT} \cdot (\frac{acetate_{ext} - acetate_{cyt}}{1 + \frac{acetate_{ext}}{K_m^{acetate_{ext}}} + \frac{acetate_{cyt}}{K_m^{acetate_{cyt}}}})$$

V_{max}^{aceT} for numerical value see Supplementary Table 1

$K_m^{acetate_{ext}} = 5.4$ [285]

$K_m^{acetate_{cyt}} = 5.4$ [285]

Acetate transport mitochondrial

$$v_{aceT_{mito}} = V_{max}^{aceT_{mito}} \cdot (\frac{acetate_{cyt} - acetate_{mito}}{1 + \frac{acetate_{cyt}}{K_m^{acetate_{cyt}}} + \frac{acetate_{mito}}{K_m^{acetate_{mito}}}})$$

$V_{max}^{aceT_{mito}}$ for numerical value see Supplementary Table 1

$K_m^{acetate_{ext}} = 5.4$ [285]

$K_m^{acetate_{cyt}} = 5.4$ [285]

Galactose metabolism

Galactose uptake

$$v_{galT} = V_{max}^{galT} \cdot \left(\frac{gal_{ext} - gal_{cyt}}{1 + \frac{gal_{ext}}{K_m^{gal_{ext}}} + \frac{gal_{cyt}}{K_m^{gal_{cyt}}}} \right)$$

V_{max}^{galT} for numerical value see Supplementary Table 1

$K_m^{gal_{ext}} = 8.5$ [286]

$K_m^{gal_{cyt}} = 8.5$ [286]

Aldose reductase

$$v_{aldor} = V_{max}^{aldor} \cdot \left(\frac{gal_{cyt}}{gal_{cyt} + K_m^{gal_{cyt}}} \right) \cdot \left(\frac{nadph_{cyt}}{nadph_{cyt} + K_m^{nadph_{cyt}}} \right)$$

V_{max}^{aldor} for numerical value see Supplementary Table 1

$K_m^{gal_{cyt}} = 55$ [287]

$K_m^{nadph_{cyt}} = 0.0026$ [288]

Galactitol transport

$$v_{galolT} = V_{max}^{galolT} \cdot \left(galactitol_{cyt} \right)$$

V_{max}^{galolT} for numerical value see Supplementary Table 1

Galactosedehydrogenase

$$v_{galdh} = V_{max}^{galdh} \cdot \left(\frac{gal_{cyt}}{gal_{cyt} + K_m^{gal_{cyt}}} \right) \cdot \left(\frac{nad_{cyt}}{nad_{cyt} + K_m^{nad_{cyt}}} \right)$$

V_{max}^{galdh} for numerical value see Supplementary Table 1

$K_m^{gal_{cyt}} = 26$ [289]

$$K_m^{nad_{cyt}} = K_0^{nad_{cyt}} \cdot \left(1 + \frac{nadh_{cyt}}{K_i^{nadh_{cyt}}}\right)$$

$K_0^{nad_{cyt}} = 0.17$ [289]

$K_i^{nadh_{cyt}} = 0.034$ [289]

Galactonate transport

$$v_{galaT} = V_{max}^{galaT} \cdot (\, galactonate_{cyt})$$

V_{max}^{galaT} for numerical value see Supplementary Table 1

Gulonate dehydrogenase [290]

$$v_{guldh} = V_{max}^{guldh} \cdot \left(\frac{galactonate_{cyt}}{galactonate_{cyt} + K_m^{galactonate_{cyt}}}\right) \cdot \left(\frac{nad_{cyt}}{nad_{cyt} + K_m^{nad_{cyt}}}\right)$$

V_{max}^{guldh} for numerical value see Supplementary Table 1

$K_m^{galactonate_{cyt}} = 0.2$ [291]

$K_m^{nad_{cyt}} = 0.01$ [291]

Decarboxylation B-keto galactonic acid [290]

$$v_{galdc} = V_{max}^{galdc} \cdot kga_{cyt}$$

V_{max}^{galdc} for numerical value see Supplementary Table 1

Xylulose kinase

$$v_{xylk} = V_{max}^{xylk} \cdot \left(\frac{xyl_{cyt}}{xyl_{cyt} + K_m^{xyl_{cyt}}}\right) \cdot \left(\frac{atp_{cyt}}{atp_{cyt} + K_m^{atp_{cyt}} \cdot \left(1 + \frac{adp_{cyt}}{K_i^{adp_{cyt}}}\right) \cdot \left(1 + \frac{amp_{cyt}}{K_i^{amp_{cyt}}}\right)}\right)$$

V_{max}^{xylk} for numerical value see Supplementary Table 1

$K_m^{xyl_{cyt}} = 4$ [292]

$K_m^{atp_{cyt}} = 0.08$ [293]

$K_i^{adp_{cyt}} = 0.34$ [293]

$K_i^{amp_{cyt}} = 0.35$ [293]

Galactokinase

$$v_{galk} = V_{max}^{galk} \cdot \left(\frac{gal_{cyt}}{gal_{cyt} + K_m^{gal_{cyt}}} \right) \cdot \left(\frac{atp_{cyt}}{atp_{cyt} + K_m^{atp_{cyt}}} \right) \cdot \left(1 - \frac{gal1p_{cyt}}{gal1p_{cyt} + K_i^{gal1p_{cyt}}} \right)$$

V_{max}^{galk} for numerical value see Supplementary Table 1

$K_m^{gal_{cyt}} = 0.15$ [294]

$K_m^{atp_{cyt}} = 0.095$ [295]

$K_i^{gal1p_{cyt}} = 2$ [294]

Galactose-1-phosphate uridyltransferase

$$v_{galt} = V_{max}^{galt} \cdot \left(\frac{gal1p_{cyt} \cdot udpglc_{cyt} - {}^{1}\!/_{K_{eq}^{galt}} \cdot udpgal_{cyt} \cdot glc1p_{cyt}}{\left(1 + \frac{gal1p_{cyt}}{K_m^{gal1p_{cyt}}}\right) \cdot \left(1 + \frac{udpglc_{cyt}}{K_m^{udpglc_{cyt}}}\right) + \left(1 + \frac{udpgal_{cyt}}{K_m^{udpgal_{cyt}}}\right) \cdot \left(1 + \frac{glc1p_{cyt}}{K_m^{glc1p_{cyt}}}\right) - 1} \right)$$

$$V_{max}^{galt} = V_0^{galt} \cdot \left(1 + \frac{gal1p_{cyt}}{K_a^{gal1p_{cyt}}}\right) \cdot \left(1 + \frac{udpglc_{cyt}}{K_a^{udpglc_{cyt}}}\right) \cdot \left(\frac{K_0^{udpglc_{cyt}}}{K_m^{udpglc_{cyt}}}\right)$$

V_0^{galt} for numerical value see Supplementary Table 1

$K_a^{gal1p_{cyt}} = 0.5$ [296]

$K_a^{udpglc_{cyt}} = 0.25$ [296]

$K_{eq}^{galt} = 1.67$ [297]

$K_m^{gal1p_{cyt}} = 0.139$ [296]

$$K_m^{udpglc_{cyt}} = K_0^{udpglc_{cyt}} \cdot \left(1 + \frac{udp_{cyt}}{K_i^{udp_{cyt}}}\right) \cdot \left(1 + \frac{utp_{cyt}}{K_i^{utp_{cyt}}}\right)$$

$K_0^{udpglc_{cyt}} = 0.156$ [296]

$K_i^{udp_{cyt}} = 0.35$ [298]

$K_i^{utp_{cyt}} = 0.13$ [298]

$K_m^{udpgal_{cyt}} = 0.167$ [296]

$K_m^{glc1p_{cyt}} = 0.16$ [299]

Uridine diphosphate (UDP)-galactose-4-epimerase

$$v_{gale} = V_{max}^{gale} \cdot \left(\frac{udpgal_{cyt} - {}^{1}\!/_{K_{eq}^{gale}} \cdot udpglc_{cyt}}{\left(1 + \frac{udpglc_{cyt}}{K_m^{udpglc_{cyt}}} + \frac{udpgal_{cyt}}{K_m^{udpgal_{cyt}}}\right)} \right)$$

$$V_{max}^{gale} = V_0^{gale} \cdot \left(1 - \frac{nadh_{cyt}}{nadh_{cyt} + K_i^{nadh_{cyt}}}\right)$$

V_0^{gale} for numerical value see Supplementary Table 1

$K_i^{nadh_{cyt}} = 0.002$ [300]

$K_{eq}^{gale} = 3.1$ [300]

$K_m^{udpglc_{cyt}} = 0.09$ [301]

$K_m^{udpgal_{cyt}} = 0.05$ [301]

Galactose-1-phosphatase

$$v_{gal1pp} = V_{max}^{gal1pp} \cdot \left(\frac{gal1p_{cyt}}{gal1p_{cyt} + K_m^{gal1p_{cyt}}} \right)$$

V_{max}^{gal1pp} for numerical value see Supplementary Table 1

$K_m^{gal1p_{cyt}} = 2.5$ [302]

Fructose metabolism

Fructose uptake

$$v_{fruT} = V_{max}^{fruT} \cdot \frac{\left(fru_{ext} - fru_{cyt}\right)}{\left(1 + \frac{fru_{ext}}{K_m^{fru_{ext}}} + \frac{fru_{cyt}}{K_m^{fru_{cyt}}}\right)}$$

V_{max}^{fruT} for numerical value see Supplementary Table 1

$K_m^{fru_{ext}} = 66.7$ [303]

$K_m^{fru_{cyt}} = 66.7$ [303]

Fructokinase

$$v_{fruk} = V_{max}^{fruk} \cdot \left(\frac{fru_{cyt}}{fru_{cyt} + K_m^{fru_{cyt}}} \right) \cdot \left(\frac{atp_{cyt}}{atp_{cyt} + K_m^{atp_{cyt}}} \right) \cdot \left(1 - \frac{adp_{cyt}^n}{adp_{cyt}^n + \left(K_i^{adp_{cyt}} \right)^n} \right)$$

V_{max}^{fruk} for numerical value see Supplementary Table 1

$K_m^{fru_{cyt}} = 0.4$ [304]

$K_m^{atp_{cyt}} = 1.5$ [304]

$n = 1.5$ [305]

$K_i^{adp_{cyt}} = 3.06$ [305]

Aldolase B

$$v_{aldB} = V_{max}^{aldB} \cdot \frac{\left(fru1p_{cyt} - {}^{1}\!/_{K_{eq}^{aldB}} \cdot gra_{cyt} \cdot dhap_{cyt} \right)}{\left(1 + \frac{fru1p_{cyt}}{K_m^{fru1p_{cyt}}} \right) + \left(1 + \frac{gra_{cyt}}{K_m^{gra_{cyt}}} \right) \cdot \left(1 + \frac{dhap_{cyt}}{K_m^{dhap_{cyt}}} \right) - 1}$$

V_{max}^{aldB} for numerical value see Supplementary Table 1

K_{eq}^{aldB} =0.0028 [306]

$K_m^{fru1p_{cyt}} = 4.5$ [307]

$K_m^{gra_{cyt}} = 0.48$ [93]

$K_m^{dhap_{cyt}} = 0.016$ [308]

Triokinase

$$v_{triok} = V_{max}^{triok} \cdot \left(\frac{gra_{cyt}}{gra_{cyt} + K_m^{gra_{cyt}}} \right) \cdot \left(\frac{atp_{cyt}}{atp_{cyt} + K_m^{atp_{cyt}}} \right)$$

V_{max}^{triok} for numerical value see Supplementary Table 1

$K_m^{gra_{cyt}} = 0.03$ [309]

$K_m^{atp_{cyt}} = 0.2$ [309]

Aldehyde reductase

$$v_{aldr} = V_{max}^{aldr} \cdot \left(\frac{gra_{cyt}}{gra_{cyt} + K_m^{gra_{cyt}}} \right) \cdot \left(\frac{nadph_{cyt}}{nadph_{cyt} + K_m^{nadph_{cyt}}} \right)$$

V_{max}^{aldr} for numerical value see Supplementary Table 1

$K_m^{gra_{cyt}} = 1.5$ [310]

$K_m^{nadph_{cyt}} = 0.004$ [310]

Aldehydedehydrogenase

$$v_{alddh-gra} = V_{max}^{alddh-gra} \cdot \left(\frac{gra_{cyt}}{gra_{cyt} + K_m^{gra_{cyt}}} \right) \cdot \left(\frac{nad_{cyt}}{nad_{cyt} + K_m^{nad_{cyt}}} \right)$$

$V_{max}^{alddh-gra}$ for numerical value see Supplementary Table 1

$$K_m^{gra_{cyt}} = K_{m0}^{gra_{cyt}} \cdot \left(1 + \frac{aald_{cyt}}{K_i^{aald_{cyt}}} \right)$$

$K_{m0}^{gra_{cyt}} = 0.3$ [311]

$K_i^{aald_{cyt}} = 1$ [283]

$K_m^{nad_{cyt}} = 0.02$ [283]

Sorbitoldehydrogenase

$$v_{sordh} = V_{max}^{sordh} \cdot \left(\frac{fru_{cyt}}{fru_{cyt} + K_m^{fru_{cyt}}} \right) \cdot \left(\frac{nadh_{cyt}}{nadh_{cyt} + K_m^{nadh_{cyt}}} \right)$$

V_{max}^{sordh} for numerical value see Supplementary Table 1

$K_m^{fru_{cyt}} = 0.136$ [312]

$K_m^{nadh_{cyt}} = 0.067$ [312]

Glycerate kinase

$$v_{gck} = V_{max}^{gck} \cdot \left(\frac{glycerate_{cyt}}{glycerate_{cyt} + K_m^{glycerate_{cyt}}} \right) \cdot \left(\frac{atp_{cyt}}{atp_{cyt} + K_m^{atp_{cyt}}} \right)$$

V_{max}^{gck} for numerical value see Supplementary Table 1

$K_m^{glycerate_{cyt}} = 0.032$ [313]

$K_m^{atp_{cyt}} = 0.091$ [313]

Glyceraldehyde transport

$$v_{graT} = V_{max}^{graT} \cdot \left(\frac{gra_{ext}}{gra_{ext} + K_m^{gra_{ext}}} \right)$$

V_{max}^{graT} for numerical value see Supplementary Table 1

$K_m^{gra_{ext}} = 1$

Valproate metabolism

Valproate uptake

$$v_{val-diff} = V_{max}^{val-diff} \cdot (val_{ext} - val_{cyt})$$

$V_{max}^{val-diff}$ for numerical value see Supplementary Table 1

Valproylcoa synthetase

$$v_{vcs} = V_{max}^{vcs} \cdot \frac{val_{cyt}}{val_{cyt} + K_m^{val_{cyt}}} \cdot \frac{atp_{cyt}}{atp_{cyt} + K_m^{atp_{cyt}}} \cdot \frac{coa_{cyt}}{coa_{cyt} + K_m^{coa_{cyt}}}$$

V_{max}^{vcs} for numerical value see Supplementary Table 1

$K_m^{val_{cyt}} = 1.65$ [314]

$K_m^{atp_{cyt}} = 0.6$ [314]

$K_m^{coa_{cyt}} = 0.0025$ [314]

Carnitinpalmitoyltransferase I

$$v_{CPT1-val} = V_{max}^{CPT1-val} \cdot \frac{valcoa_{cyt} \cdot car_{cyt}}{\left(valcoa_{cyt} + K_m^{valcoa_{cyt}}\right) \cdot \left(car_{cyt} + K_m^{car_{cyt}}\right)}$$

$V_{max}^{CPT1-val}$ for numerical value see Supplementary Table 1

$$K_m^{valcoa_{cyt}} = K_0^{valcoa_{cyt}} \cdot \left(1 + \frac{malcoa2_{imm}}{K_i^{malcoa2_{imm}}}\right) \cdot \left(1 + \frac{c16coa_{cyt}}{K_i^{c16coa_{cyt}}}\right)$$

$K_i^{c16coa_{cyt}}$ =0.03 [5]

$K_i^{malcoa2_{imm}} = 0.0025$ [6]

$K_m^{car_{cyt}}$ =0.032 [7]

$K_0^{valcoa_{cyt}} = 0.057$ [315]

Carnitin-Acylcarnitin translocase

$$v_{CACT-val} = V_{max}^{CACT-val} \cdot \left(\frac{car_{mito} \cdot valcar_{cyt} - 1/K_{eq}^{CACT} \cdot car_{cyt} \cdot valcar_{mito}}{\left(1 + \frac{car_{mito}}{K_m^{car_{mito}}}\right)\left(1 + \frac{valcar_{cyt}}{K_m^{valcar_{cyt}}}\right) + \left(1 + \frac{car_{cyt}}{K_m^{car_{cyt}}}\right)\left(1 + \frac{valcar_{mito}}{K_m^{valcar_{mito}}}\right) - 1} \right)$$

$V_{max}^{CACT-val}$ for numerical value see Supplementary Table 1

$K_{eq}^{CACT} = 1$

$K_m^{car_{mito}} = 5.8$ [10]

$K_m^{valcar_{cyt}}$ =0.01

$K_m^{car_{cyt}} = 0.51$ [216]

$K_m^{valcar_{mito}} = 0.01$

Carnitinpalmitoyltransferase 2

$$v_{CPT2-val} = V_{max}^{CPT2-val} \cdot \left(\frac{valcar_{mito} \cdot coa_{mito} - 1/K_{eq}^{CPT2} \cdot valcoa_{mito} \cdot car_{mito}}{\left(1 + \frac{valcar_{mito}}{K_m^{valcar_{mito}}}\right)\left(1 + \frac{coa_{mito}}{K_m^{coa_{mito}}}\right) + \left(1 + \frac{valcoa_{mito}}{K_m^{valcoa_{mito}}}\right)\left(1 + \frac{car_{mito}}{K_m^{car_{mito}}}\right) - 1} \right)$$

$V_{max}^{CPT2-val}$ for numerical value see Supplementary Table 1

$K_{eq}^{CPT2} = 2$ [13]

$K_m^{valcar_{mito}} = 0.2$

$K_m^{coa_{mito}} = 0.0055$ [14]

$K_m^{valcoa_{mito}} = 0.2$

$K_m^{car_{mito}}$ =0.121 [15]

branched chain acyl-coa dehydrogenase (valcoa)

$$v_{valcoa-bcdh} = V_{max}^{valcoa-bcdh} \cdot \left(\frac{valcoa_{mito}}{valcoa_{mito} + K_m^{valcoa_{mito}}}\right) \cdot \left(\frac{etffad_{mito}}{etffad_{mito} + K_m^{etffad_{mito}}}\right)$$

$V_{max}^{valcoa-bcdh}$ for numerical value see Supplementary Table 1

$K_m^{valcoa_{mito}} = 1$

$K_m^{etffad_{mito}} = 0.0045$ [16]

Enoyl-coa hydratase (Crontonase) (e-valcoa)

$$v_{ehyd-evalcoa} = V_{max}^{ehyd-evalcoa} \cdot \left(\frac{evalcoa_{mito} - {}^{1}\!/\!_{K_{eq}^{ehyd-evalcoa}} \cdot lvalcoa_{mito}}{evalcoa_{mito} + K_m^{evalcoa_{mito}}}\right)$$

$V_{max}^{ehyd-evalcoa}$ for numerical value see Supplementary Table 1

$K_{eq}^{ehyd-evalcoa} = 2$ [18]

$K_m^{evalcoa_{mtio}} = K_0^{evalcoa_{mito}} \cdot \left(1 + \frac{kc4coa_{mito}}{K_i^{kc4coa_{mito}}}\right)$

$K_0^{evalcoa_{mito}} = 0.045$

$K_i^{kc4coa_{mito}} = 0.025$ [19]

3-hydroxyacyl-coa dehydrogenase (lvalcoa)

$$v_{3hdh-lvalcoa} = V_{max}^{3hdh-lvalcoa} \cdot \left(\frac{lvalcoa_{mito} \cdot nad_{mito} - {}^{1}\!/\!_{K_{eq}^{3hdh-lvalcoa_{mito}}} \cdot kvalcoa_{mito} \cdot nadh_{mito}}{\left(1 + \frac{lvalcoa_{mito}}{K_m^{lvalcoa_{mito}}}\right) \cdot \left(1 + \frac{nad_{mito}}{K_m^{nad_{mito}}}\right) + \left(1 + \frac{kvalcoa_{mito}}{K_m^{kvalcoa_{mito}}}\right) \cdot \left(1 + \frac{nadh_{mito}}{K_m^{nadh_{mito}}}\right) - 1}\right)$$

$V_{max}^{3hdh-lvalcoa}$ for numerical value see Supplementary Table 1

$K_{eq}^{3hdh-lvalcoa} = 10^4$

$K_m^{lvalcoa_{mito}}$ =0.04

$K_m^{nad_{mito}}$ =0.0585 [21]

$K_m^{kvalcoa_{mito}} = 0.01$

$K_m^{nadh_{mito}}$ =0.0054 [21]

3-ketoacyl-coa thiolase (kvalcoa)

$$v_{3kt}^{kvalcoa} = V_{max}^{3kt-kvalcoa} \cdot \left(\frac{kvalcoa_{mito} \cdot coa_{mito} - {}^{1}\!/\!{}_{K_{eq}^{3kt}} \cdot c3coa_{mito} \cdot c5coa_{mito}}{\left(coa_{mito} + K_m^{coa_{mito}}\right) \cdot \left(kvalcoa_{mito} + K_m^{kvalcoa_{mito}}\right)} \right)$$

$V_{max}^{3kt-kc8}$ for numerical value see Supplementary Table 1

$K_{eq}^{3kt} = 2500$ [24]

$K_m^{coa_{mito}} = 0.0189$ [25]

$K_m^{kvalcoa_{mito}} = 0.01$

Stoichiometric matrix:

$$\frac{d}{dt}aald_{cyt} = +v_{adh} - v_{alddhI} - v_{alddhII} - v_{aaldT}$$

$$\frac{d}{dt}aald_{mito} = -v_{alddh_{mito}} + \frac{Vol_{cyt}}{Vol_{mito}} \cdot v_{aaldT}$$

$$\frac{d}{dt}acac_{cyt} = +\frac{Vol_{mito}}{Vol_{cyt}} \cdot v_{acacT} + v_{acac-ex} - v_{acaccoa-syn} + v_{hmgl}$$

$$\frac{d}{dt}acac_{ext} = 0$$

$$\frac{d}{dt}acac_{mito} = +v_{hmg-lys} - v_{\beta hdh} - v_{acacT}$$

$$\frac{d}{dt}acetate_{cyt} = -v_{acoa-syn} + v_{acglu-hyd} + v_{alddhI} + v_{alddhII} + v_{aceT} - v_{aceT_{mito}}$$

$$\frac{d}{dt}acetate_{ext} = 0$$

$$\frac{d}{dt}acetate_{mito} = +\frac{Vol_{mito}}{Vol_{cyt}} \cdot v_{aceT_{mito}} + v_{alddh_{mito}}$$

$$\frac{d}{dt}acglu_{cyt} = +\frac{Vol_{mito}}{Vol_{cyt}} \cdot v_{acgluT} - v_{acglu-hyd}$$

$$\frac{d}{dt}acglu_{mito} = +v_{aglu-syn} - v_{acgluT}$$

$$\frac{d}{dt}acoa_{cyt} = +v_{cit-lys} - v_{acc1} - v_{acc2} + v_{mdc2} - v_{fas-c4} + v_{acoa-syn} + 2 \cdot v_{3kt-cyt}^{kc4coa} - v_{hmg-syn-cyt} + v_{hmgl}$$

$$\frac{d}{dt}acoa_{mito} = +2 \cdot v_{3kt}^{kc4coa} + v_{3kt}^{kc5coa} + v_{3kt}^{kc6coa} + v_{3kt}^{kc8coa} + v_{3kt}^{kc10coa} + v_{3kt}^{kc12coa} + v_{3kt}^{kc14coa} + v_{3kt}^{kc16coa} + v_{pdhc} - v_{cs} - v_{aglu-syn} + v_{hmg-lys} - v_{hmg-syn}$$

$$\frac{d}{dt}adp_{cyt} = -\frac{v_{nex}}{10 \cdot F \cdot Vol_{cyt}} + v_{ndk_{cyt}} + 2 \cdot v_{ak_{cyt}} + v_{atp-usage} + v_{Gk} + v_{ndk_{cyt}}^{udp} + v_{pfk2} - v_{pfk1} - v_{pgk} - v_{pk} + v_{cit-lys} + v_{acc1} + v_{acc2} + v_{glycK} + v_{hkIII} + v_{gln-syn} + v_{xylk} + v_{galk} + v_{fruk} + v_{triok} + v_{gck} + v_{mevk} + v_{pmevk} + v_{mdp}$$

$$\frac{d}{dt}adp_{mito} = -v_{scs-atp} - \frac{v_{F0F1}}{10 \cdot F \cdot Vol_{mito}} + \frac{v_{nex}}{10 \cdot F \cdot Vol_{mito}} + v_{ndk_{mito}} + v_{pc} + v_{pcc} + 2 \cdot v_{cps}$$

$$\frac{d}{dt}akg_{cyt} = -v_{asat} + v_{mac} - v_{aat}$$

$$\frac{d}{dt}akg_{mito} = +v_{idh} - v_{kgdhc} - v_{asat_{mito}} - \frac{Vol_{cyt}}{Vol_{mito}} \cdot v_{mac} + v_{gdh} + v_{gdh-nadp}$$

$$\frac{d}{dt}ala_{cyt} = -v_{aat} + v_{alaT}$$

$$\frac{d}{dt} amp_{cyt} = +v_{ACSL5} + v_{ACSL4} + v_{ACSL1} + v_{acoa-syn} + v_{ass} + v_{vcs} + v_{acaccoa-syn} - v_{ak_{cyt}}$$

$$\frac{d}{dt} apoB = +v_{apoB-syn} - v_{apoB-deg} - \frac{Vol_{er}}{Vol_{cyt}} \cdot v_{MTP}^{tag}$$

$$\frac{d}{dt} arg_{cyt} = +v_{asl} - v_{argase} - v_{argT}$$

$$\frac{d}{dt} arg_{mito} = +\frac{Vol_{cyt}}{Vol_{mito}} \cdot v_{argT}$$

$$\frac{d}{dt} argsucc_{cyt} = +v_{ass} - v_{asl}$$

$$\frac{d}{dt} asp_{cyt} = -v_{asat} + v_{agc} - v_{ass}$$

$$\frac{d}{dt} asp_{mito} = -v_{asat_{mito}} - \frac{Vol_{cyt}}{Vol_{er}} \cdot v_{agc}$$

$$\begin{aligned}\frac{d}{dt} atp_{cyt} = &-v_{ACSL5} - v_{ACSL4} - v_{ACSL1} + \frac{v_{nex}}{10 \cdot F \cdot Vol_{cyt}} - v_{ndk_{cyt}} - v_{atp-usage} - v_{Gk} - v_{pfk2} - v_{pfk1} \\ &+ v_{pgk} + v_{pk} - v_{ndk_{cyt}}^{udp} - v_{cit-lys} - v_{acc1} - v_{acc2} - v_{glycK} - v_{acoa-syn} + v_{ass} - v_{hkIII} \\ &- v_{gln-syn} - v_{xylk} - v_{galk} - v_{fruk} - v_{triok} - v_{gck} - v_{vcs} - v_{acaccoa-syn} - v_{mevk} \\ &- v_{pmevk} - v_{mdp} - v_{ak_{cyt}}\end{aligned}$$

$$\frac{d}{dt} atp_{mito} = +v_{scs-atp} + \frac{v_{F0F1}}{10 \cdot F \cdot Vol_{mito}} - \frac{v_{nex}}{10 \cdot F \cdot Vol_{mito}} - v_{ndk_{mito}} - v_{pc} - v_{pcc} - 2 \cdot v_{cps}$$

$$\frac{d}{dt} bhbut_{cyt} = +\frac{Vol_{mito}}{Vol_{cyt}} \cdot v_{\beta hbT} + v_{\beta hb-ex}$$

$$\frac{d}{dt} bhbut_{ext} = 0$$

$$\frac{d}{dt} bhbut_{mito} = +v_{\beta hdh} - v_{\beta hbT}$$

$$\frac{d}{dt} bpg13_{cyt} = +v_{gapdh} - v_{pgk}$$

$$\frac{d}{dt} c10coa_{cyt} = +v_{fas-c10} - v_{fas-c12}$$

$$\frac{d}{dt} c10coa_{mito} = -v_{c10coa-mcdh} - v_{c10coa-lcdh} + v_{3kt}^{kc12coa}$$

$$\frac{d}{dt} c12coa_{cyt} = +v_{fas-c12} - v_{fas-c14}$$

$$\frac{d}{dt} c12coa_{mito} = -v_{c12coa-mcdh} - v_{c12coa-lcdh} + v_{3kt}^{kc14coa}$$

$$\frac{d}{dt} c14coa_{cyt} = +v_{fas-c14} - v_{fas-c16}$$

$$\frac{d}{dt}c14coa_{mito} = -v_{c14coa-lcdh} + v_{3kt}^{kc16coa}$$

$$\frac{d}{dt}c16car_{cyt} = +v_{CPT1} - v_{CACT}$$

$$\frac{d}{dt}c16car_{mito} = +\frac{Vol_{cyt}}{Vol_{mito}} \cdot v_{CACT} - v_{CPT2}$$

$$\frac{d}{dt}c16coa_{cyt} = +v_{ACSL5} + v_{ACSL4} + v_{ACSL1} - v_{CPT1} + v_{fas-c16} - v_{gpat} - v_{agpat} - v_{dgat}$$

$$\frac{d}{dt}c16coa_{mito} = +v_{CPT2} - v_{c16coa-lcdh}$$

$$\frac{d}{dt}c16_{cyt} = +v_{c16_{cyt}-uptake}^{carrier} + v_{c16_{cyt}-uptake}^{diffusion} - v_{ACSL5} - v_{ACSL4} - v_{ACSL1} + \frac{Vol_{ld}}{Vol_{cyt}} \cdot v_{HSL}^{dag} + \frac{Vol_{ld}}{Vol_{cyt}} \cdot v_{ATGL}^{tag} + \frac{Vol_{ld}}{Vol_{cyt}} \cdot v_{magl} + \frac{Vol_{ld}}{Vol_{cyt}} \cdot v_{cee} - v_{acat}$$

$$\frac{d}{dt}c16_{ext} = 0$$

$$\frac{d}{dt}c4coa_{cyt} = +v_{fas-c4} - v_{fas-c6}$$

$$\frac{d}{dt}c4coa_{mito} = -v_{c4coa-scdh} + v_{3kt}^{kc6coa}$$

$$\frac{d}{dt}c5_{coa_{mito}} = -v_{c5coa-scdh} + v_{3kt}^{kvalcoa}$$

$$\frac{d}{dt}c6coa_{cyt} = +v_{fas-c6} - v_{fas-c8}$$

$$\frac{d}{dt}c6coa_{mito} = -v_{c6coa-mcdh} + v_{3kt}^{kc8coa}$$

$$\frac{d}{dt}c8coa_{cyt} = +v_{fas-c8} - v_{fas-c10}$$

$$\frac{d}{dt}c8coa_{mito} = -v_{c8coa-mcdh} + v_{3kt}^{kc10coa}$$

$$\frac{d}{dt}car_{cyt} = -v_{CPT1} + v_{CACT} - v_{CPT1-val} + v_{CACT-val}$$

$$\frac{d}{dt}car_{mito} = -\frac{Vol_{cyt}}{Vol_{mito}} \cdot v_{CACT} + v_{CPT2} - \frac{Vol_{cyt}}{Vol_{mito}} \cdot v_{CACT-val} + v_{CPT2-val}$$

$$\frac{d}{dt}ce_{er} = -v_{LD-syn-ce} - v_{MTP}^{ce} + \frac{Vol_{cyt}}{Vol_{er}} \cdot v_{acat}$$

$$\frac{d}{dt}ce_{ld} = +\frac{Vol_{er}}{Vol_{ld}} \cdot v_{LD-syn-ce} - v_{cee}$$

$$\frac{d}{dt}ce_{vldl} = +\frac{Vol_{er}}{Vol_{vldl}} v_{MTP}^{ce} - v_{VLDL-ex-ce}$$

$$\frac{d}{dt}chol_{er} = +\frac{Vol_{ld}}{Vol_{er}} \cdot v_{cee} + v_{s7r-dhchol} + v_{s24r-desmo} - \frac{Vol_{cyt}}{Vol_{er}} \cdot v_{acat}$$

$$\frac{d}{dt}cit_{cyt} = +\frac{Vol_{mito}}{Vol_{cyt}} \cdot v_{cit-mal} - v_{cit-lys}$$

$$\frac{d}{dt}cit_{mito} = +v_{cs} - \mathrm{v_{ac}} - v_{cit-mal}$$

$$\frac{d}{dt}cl_{cyt} = 0$$

$$\frac{d}{dt}cl_{mito} = +\frac{I_{cl_{ed}}}{10 \cdot F \cdot Vol_{mito}}$$

$$\frac{d}{dt}cmp_{mito} = +v_{cps} - v_{otc}$$

$$\begin{aligned}\frac{d}{dt}coa_{cyt} = & -v_{ACSL5} - v_{ACSL4} - v_{ACSL1} + v_{CPT1} - v_{cit-lys} + v_{fas-c4} + v_{fas-c6} + v_{fas-c8} + v_{fas-c10} \\ & + v_{fas-c12} + v_{fas-c14} + v_{fas-c16} + v_{gpat} + v_{agpat} + v_{dgat} - v_{acoa-syn} - v_{vcs} + v_{CPT1-val} \\ & - v_{acaccoa-syn} - v_{3kt-cyt}^{kc4coa} + v_{hmg-syn-cyt} + v_{hmgr} + v_{acat}\end{aligned}$$

$$\begin{aligned}\frac{d}{dt}coa_{mito} = & -v_{CPT2} - v_{3kt}^{kc4coa} - v_{3kt}^{kc5coa} - v_{3kt}^{kc6coa} - v_{3kt}^{kc8coa} - v_{3kt}^{kc10coa} - v_{3kt}^{kc12coa} - v_{3kt}^{kc14coa} \\ & - v_{3kt}^{kc16coa} - v_{pdhc} + v_{cs} - v_{kgdhc} + v_{scs-atp} + v_{scs-gtp} + v_{aglu-syn} + v_{hmg-syn} \\ & - v_{CPT2-val} - v_{3kt}^{kvalcoa}\end{aligned}$$

$$\frac{d}{dt}co2_{cyt} = 0$$

$$\frac{d}{dt}co2_{mito} = 0$$

$$\frac{d}{dt}ctl_{cyt} = +\frac{Vol_{mito}}{Vol_{cyt}} \cdot v_{ocT} - v_{ass}$$

$$\frac{d}{dt}ctl_{mito} = +v_{otc} - v_{ocT}$$

$$\frac{d}{dt}cytc_{ox_{mm}} = -\frac{v_{cxIII}}{10 \cdot F \cdot Vol_{membrane}} + \frac{v_{cxIV}}{10 \cdot F \cdot Vol_{membrane}}$$

$$\frac{d}{dt}cytc_{red_{mm}} = +\frac{v_{cxIII}}{10 \cdot F \cdot Vol_{membrane}} - \frac{v_{cxIV}}{10 \cdot F \cdot Vol_{membrane}}$$

$$\frac{d}{dt}dag_{er} = +\frac{Vol_{cyt}}{Vol_{er}} \cdot v_{pap} - \frac{Vol_{cyt}}{Vol_{er}} \cdot v_{dgat}$$

$$\frac{d}{dt}dag_{ld} = +v_{ATGL}^{tag} - v_{HSL}^{dag}$$

$$\frac{d}{dt}ddimdihtmasca_{er} = +v_{mso-dmdihtmas} - v_{casdc-ddimdihtmasca}$$

$$\frac{d}{dt}ddimktmas_{er} = +v_{casdc-ddimtmasca} - v_{3ksr-ddimktmas}$$

$$\frac{d}{dt}ddimkdihtmas_{er} = +v_{casdc-ddimdihtmasca} - v_{3ksr-ddimkdihtmas}$$

$$\frac{d}{dt}ddimtmasca_{er} = +v_{mso-dmtmas} - v_{casdc-ddimtmasca}$$

$$\frac{d}{dt}desmo_{er} = +v_{s7r-dhdesm} - v_{s24r-desmo}$$

$$\frac{d}{dt}dhap_{cyt} = +v_{ald} - v_{tpi} - v_{g3pdh} - v_{g3pdh_{mito}} + v_{aldB}$$

$$\frac{d}{dt}dhchol_{er} = +v_{lathox-lath} - v_{s7r-dhchol} + v_{s24r-dhdesmo}$$

$$\frac{d}{dt}dhdesmo_{er} = +v_{lathox-dhlath} - v_{s7r-dhdesm} - v_{s24r-dhdesmo}$$

$$\frac{d}{dt}dhlath_{er} = +v_{s78i-zym} - v_{lathox-dhlath} - v_{s24r-dhlath}$$

$$\frac{d}{dt}dihffmas_{er} = +v_{s14dm-dihlan} - v_{s14r-dihffmas} + v_{s24r-ffmas}$$

$$\frac{d}{dt}dihlan_{er} = -v_{s14dm-dihlan} + v_{s24r-lan}$$

$$\frac{d}{dt}dihtmas_{er} = +v_{s14r-dihffmas} - v_{mso-dihtmas} + v_{s24r-tmas}$$

$$\frac{d}{dt}dmdihtmas_{er} = -v_{mso-dmdihtmas} + v_{3ksr-dmkdihtmas}$$

$$\frac{d}{dt}dmdihtmasca_{er} = +v_{mso-dihtmas} - v_{casdc-dmdihtmasca}$$

$$\frac{d}{dt}dmemcoa_{mito} = +v_{pcc} - v_{mmrm}$$

$$\frac{d}{dt}dmkdihtmas_{er} = +v_{casdc-dmdihtmasca} - v_{3ksr-dmkdihtmas}$$

$$\frac{d}{dt}dmktmas_{er} = +v_{casdc-dmtmasca} - v_{3ksr-dmktmas}$$

$$\frac{d}{dt}dmpp_{cyt} = +v_{ippi} - v_{gpps}$$

$$\frac{d}{dt}dmtmas_{er} = -v_{mso-dmtmas} + v_{3ksr-dmktmas}$$

$$\frac{d}{dt}dmtmasca_{er} = +v_{mso-tmas} - v_{casdc-dmtmasca}$$

$$\frac{d}{dt}ec10coa_{mito} = +v_{c10coa-mcdh} + v_{c10coa-lcdh} - v_{ehyd-ec10}$$

$$\frac{d}{dt}ec12coa_{mito} = +v_{c12coa-mcdh} + v_{c12coa-lcdh} - v_{ehyd-ec12}$$

$$\frac{d}{dt}ec14coa_{mito} = +v_{c14coa-lcdh} - v_{ehyd-ec14}$$

$$\frac{d}{dt}ec16coa_{mito} = +v_{c16coa-lcdh} - v_{ehyd-ec16}$$

$$\frac{d}{dt}ec4coa_{mito} = +v_{c4coa-scdh} - v_{ehyd-ec4}$$

$$\frac{d}{dt}ec5_{coa_{mito}} = +v_{c5coa-scdh} - v_{ehyd-ec5}$$

$$\frac{d}{dt}ec6coa_{mito} = +v_{c6coa-mcdh} - v_{ehyd-ec6}$$

$$\frac{d}{dt}ec8coa_{mito} = +v_{c8coa-mcdh} - v_{ehyd-ec8}$$

$$\frac{d}{dt}e4p_{cyt} = +v_{taldo} + v_{tketo2}$$

$$\frac{d}{dt}etffad_{mito} = -v_{c4coa-scdh} - v_{c5coa-scdh} - v_{c6coa-mcdh} - v_{c8coa-mcdh} - v_{c10coa-mcdh} - v_{c12coa-mcdh} - v_{c10coa-lcdh} - v_{c12coa-lcdh} - v_{c14coa-lcdh} - v_{c16coa-lcdh} + v_{ETF-FAD} - v_{valcoa-bcdh}$$

$$\frac{d}{dt}etffadh2_{mito} = +v_{c4coa-scdh} + v_{c5coa-scdh} + v_{c6coa-mcdh} + v_{c8coa-mcdh} + v_{c10coa-mcdh} + v_{c12coa-mcdh} + v_{c10coa-lcdh} + v_{c12coa-lcdh} + v_{c14coa-lcdh} + v_{c16coa-lcdh} - v_{ETF-FAD} + v_{valcoa-bcdh}$$

$$\frac{d}{dt}etfq_{mito} = -v_{ETF-FAD} + v_{ETF-QO}$$

$$\frac{d}{dt}etfqh2_{mito} = +v_{ETF-FAD} - v_{ETF-QO}$$

$$\frac{d}{dt}ethanol_{cyt} = +v_{ethT} - v_{adh}$$

$$\frac{d}{dt}ethanol_{ext} = 0$$

$$\frac{d}{dt}evalcoa_{mito} = +v_{valcoa-bcdh} - v_{ehyd-evalcoa}$$

$$\frac{d}{dt}ffmas_{er} = +v_{s14dm-lan} - v_{s14r-ffmas} - v_{s24r-ffmas}$$

$$\frac{d}{dt}fpp_{cyt} = +v_{fpps} - 2 \cdot v_{sqs-nadph} - 2 \cdot v_{sqs-nadh}$$

$$\frac{d}{dt}fru_{cyt} = +v_{fruT} - v_{fruk} - v_{sordh}$$

$$\frac{d}{dt}fru_{ext} = 0$$

$$\frac{d}{dt}fru1p_{cyt} = +v_{fruk} - v_{aldB}$$

$$\frac{d}{dt}fru16bp_{cyt} = +v_{pfk1} - v_{fbp1} - v_{ald}$$

$$\frac{d}{dt}fru26bp_{cyt} = +v_{pfk2} - v_{fbp2}$$

$$\frac{d}{dt}fru6p_{cyt} = +v_{gpi} - v_{pfk2} + v_{fbp2} - v_{pfk1} + v_{fbp1} + v_{taldo} - v_{tketo2}$$

$$\frac{d}{dt}fum_{cyt} = +v_{asl} - v_{fmT}$$

$$\frac{d}{dt}fum_{mito} = +v_{succdh} - v_{fum} + \frac{Vol_{cyt}}{Vol_{mito}} \cdot v_{fmT}$$

$$\frac{d}{dt}g3p_{cyt} = +v_{g3pdh} + v_{g3pdh_{mito}} + v_{glycK} - v_{gpat}$$

$$\frac{d}{dt}gal_{cyt} = +v_{galT} - v_{galk} - v_{aldor} - v_{galdh} + v_{gal1pp}$$

$$\frac{d}{dt}gal_{ext} = 0$$

$$\frac{d}{dt}gal1p_{cyt} = -v_{galt} + v_{galk} - v_{gal1pp}$$

$$\frac{d}{dt}galactitol_{cyt} = -v_{galolT} + v_{aldor}$$

$$\frac{d}{dt}galactitol_{ext} = 0$$

$$\frac{d}{dt}galactonate_{cyt} = +v_{galdh} - v_{guldh}$$

$$\frac{d}{dt}gdp_{cyt} = -v_{ndk_{cyt}} + v_{pepck}$$

$$\frac{d}{dt}gdp_{mito} = -v_{scs-gtp} - v_{ndk_{mito}} + v_{pepck_{mito}}$$

$$\frac{d}{dt}glc_{cyt} = +v_{gluT2} - v_{Gk} - v_{GlcT_{ER}} - v_{hkIII}$$

$$\frac{d}{dt}glc_{er} = +v_{Glc6PP_{ER}} + \frac{Vol_{cyt}}{Vol_{er}} \cdot v_{GlcT_{ER}}$$

$$\frac{d}{dt}glc_{ext} = 0$$

$$\frac{d}{dt}glc1p_{cyt} = -v_{gpm} - v_{upgase} + v_{gp} + v_{galt}$$

$$\frac{d}{dt}glc6p_{cyt} = -v_{Glc6PT_{ER}} + v_{Gk} - v_{gpi} + v_{gpm} - v_{g6pdh} + v_{hkIII}$$

$$\frac{d}{dt}glc6p_{er} = +\frac{Vol_{cyt}}{Vol_{er}} \cdot v_{Glc6PT_{ER}} - v_{Glc6PP_{ER}}$$

$$\frac{d}{dt}gln_{cyt} = +v_{glnT} - v_{glnT_{mito}} + v_{gln-syn}$$

$$\frac{d}{dt}gln_{ext} = 0$$

$$\frac{d}{dt}gln_{mito} = +\frac{Vol_{cyt}}{Vol_{mito}} \cdot v_{glnT} - v_{glnase}$$

$$\frac{d}{dt}glu_{cyt} = +v_{asat} - v_{agc} - v_{gluT_{mito}} + v_{aat} + v_{gluT} + v_{acglu-hyd} - v_{gln-syn}$$

$$\frac{d}{dt}glu_{ext} = 0$$

$$\frac{d}{dt}glu_{mito} = +v_{asat_{mito}} + \frac{Vol_{cyt}}{Vol_{mito}} \cdot v_{agc} + v_{glnase} - v_{gdh} - v_{gdh-nadp} + \frac{Vol_{cyt}}{Vol_{mito}} \cdot v_{gluT} - v_{aglu-syn}$$

$$\frac{d}{dt}glyc_{cyt} = +v_{glycT} - v_{glycK} + \frac{Vol_{ld}}{Vol_{cyt}} \cdot v_{magl} + v_{aldr}$$

$$\frac{d}{dt}glyc_{ext} = 0$$

$$\frac{d}{dt}glycerate = +v_{alddh-gra} - v_{gck}$$

$$\frac{d}{dt}glyglc = +v_{gs} - v_{gp}$$

$$\frac{d}{dt}gpp_{cyt} = +v_{gpps} - v_{fpps}$$

$$\frac{d}{dt}gra_{cyt} = +v_{aldB} - v_{triok} - v_{aldr} - v_{alddh-gra} - v_{graT}$$

$$\frac{d}{dt}gra_{ext} = 0$$

$$\frac{d}{dt}grap_{cyt} = +v_{ald} + v_{tpi} - v_{gapdh} - v_{taldo} - v_{tketo1} - v_{tketo2} + v_{triok}$$

$$\frac{d}{dt}gtp_{cyt} = +v_{ndk_{cyt}} - v_{pepck}$$

$$\frac{d}{dt}gtp_{mito} = +v_{scs-gtp} + v_{ndk_{mito}} - v_{pepck_{mito}}$$

$$\frac{d}{dt}h_{cyt} = +\frac{I_H^{pump} - I_{H_{ed}} - v_{P-ex} + I_k^{pump} + I_{na}^{pump} - 3 \cdot v_{F0F1}}{10 \cdot F \cdot Vol_{cyt}}$$

$$\frac{d}{dt}h_{mito} = +\frac{-I_H^{pump} + I_{H_{ed}} + v_{P-ex} - I_k^{pump} - I_{na}^{pump} + 3 \cdot v_{F0F1}}{10 \cdot F \cdot Vol_{mito}}$$

$$\frac{d}{dt}hco3_{cyt} = 0$$

$$\frac{d}{dt}hco3_{mito} = 0$$

$$\frac{d}{dt}hmgcoa_{cyt} = +v_{hmg-syn-cyt} - v_{hmgl} - v_{hmgr}$$

$$\frac{d}{dt}hmgcoa_{mito} = +v_{hmg-syn} - v_{hmg-lys}$$

$$\frac{d}{dt}ipp_{cyt} = +v_{mdp} - v_{ippi} - v_{gpps} - v_{fpps}$$

$$\frac{d}{dt}isocit_{mito} = +\mathrm{v}_{\mathrm{ac}} - v_{idh}$$

$$\frac{d}{dt}kc10coa_{mito} = +v_{3hdh-lc10} - v_{3kt}^{kc10coa}$$

$$\frac{d}{dt}kc12coa_{mito} = +v_{3hdh-lc12} - v_{3kt}^{kc12coa}$$

$$\frac{d}{dt}kc14coa_{mito} = +v_{3hdh-lc14} - v_{3kt}^{kc14coa}$$

$$\frac{d}{dt}kc16coa_{mito} = +v_{3hdh-lc16} - v_{3kt}^{kc16coa}$$

$$\frac{d}{dt}kc4coa_{cyt} = +v_{acaccoa-syn} - v_{3kt-cyt}^{kc4coa} - v_{hmg-syn-cyt}$$

$$\frac{d}{dt}kc4coa_{mito} = +v_{3hdh-lc4} - v_{3kt}^{kc4coa} - v_{hmg-syn}$$

$$\frac{d}{dt}kc5_{coa_{mito}} = +v_{3hdh-lc5} - v_{3kt}^{kc5coa}$$

$$\frac{d}{dt}kc6\ coa_{mito} = +v_{3hdh-lc6} - v_{3kt}^{kc6coa}$$

$$\frac{d}{dt}kc8coa_{mito} = +v_{3hdh-lc8} - v_{3kt}^{kc8coa}$$

$$\frac{d}{dt}k_{cyt} = 0$$

$$\frac{d}{dt}k_{mito} = +\frac{I_K^{pump} + I_{k_{ed}}}{10 \cdot F \cdot Vol_{mito}}$$

$$\frac{d}{dt}kga_{cyt} = +v_{guldh} - v_{galdc}$$

$$\frac{d}{dt}kvalcoa_{mito} = +v_{3hdh-lvalcoa} - v_{3kt}^{kvalcoa}$$

$$\frac{d}{dt}lan_{er} = +\frac{Vol_{cyt}}{Vol_{er}} \cdot v_{osc} - v_{s14dm-lan} - v_{s24r-lan}$$

$$\frac{d}{dt}lath_{er} = +v_{s78i-zymostenol} - v_{lathox-lath} + v_{s24r-dhlath}$$

$$\frac{d}{dt}lc10coa_{mito} = +v_{ehyd-ec10} - v_{3hdh-lc10}$$

$$\frac{d}{dt}lc12coa_{mito} = +v_{ehyd-ec12} - v_{3hdh-lc12}$$

$$\frac{d}{dt}lc14coa_{mito} = +v_{ehyd-ec14} - v_{3hdh-lc14}$$

$$\frac{d}{dt}lc16coa_{mito} = +v_{ehyd-ec16} - v_{3hdh-lc16}$$

$$\frac{d}{dt}lc4coa_{mito} = +v_{ehyd-ec4} - v_{3hdh-lc4}$$

$$\frac{d}{dt}lc5_{coa_{mito}} = +v_{ehyd-ec5} - v_{3hdh-lc5}$$

$$\frac{d}{dt}lc6coa_{mito} = +v_{ehyd-ec6} - v_{3hdh-lc6}$$

$$\frac{d}{dt}lc8coa_{mito} = +v_{ehyd-ec8} - v_{3hdh-lc8}$$

$$\frac{d}{dt}lac_{cyt} = +v_{ldh} + v_{lacT}$$

$$\frac{d}{dt}lac_{ext} = 0$$

$$\frac{d}{dt}lmemcoa_{mito} = +v_{mmrm} - v_{mmm}$$

$$\frac{d}{dt}lpa_{er} = +\frac{Vol_{cyt}}{Vol_{er}} \cdot v_{gpat} - \frac{Vol_{cyt}}{Vol_{er}} \cdot v_{agpat}$$

$$\frac{d}{dt}lvalcoa_{mito} = +v_{ehyd-evalcoa} - v_{3hdh-lvalcoa}$$

$$\frac{d}{dt}mag_{ld} = -v_{magl} + v_{HSL}^{dag}$$

$$\frac{d}{dt}mal_{cyt} = +v_{malT} + v_{mal-pyrT} - v_{mdh} - v_{mac} - \frac{Vol_{mito}}{Vol_{cyt}} \cdot v_{cit-mal} - v_{fmT} - v_{me}$$

$$\frac{d}{dt}mal_{mito} = +v_{fum} - v_{mdh_{mito}} - \frac{Vol_{cyt}}{Vol_{mito}} \cdot v_{malT} - \frac{Vol_{cyt}}{Vol_{mito}} \cdot v_{mal-pyrT} + \frac{Vol_{cyt}}{Vol_{mito}} \cdot v_{mac} + v_{cit-mal} - \frac{Vol_{cyt}}{Vol_{mito}} \cdot v_{fmT}$$

$$\frac{d}{dt}malcoa_{cyt} = +v_{acc1} - v_{fas-c4} - v_{fas-c6} - v_{fas-c8} - v_{fas-c10} - v_{fas-c12} - v_{fas-c14} - v_{fas-c16}$$

$$\frac{d}{dt}malcoa2_{imm} = +\frac{Vol_{cyt}}{Vol_{imm}} \cdot v_{acc2} - \frac{Vol_{cyt}}{Vol_{imm}} \cdot v_{mdc2}$$

$$\frac{d}{dt}mev_{cyt} = +v_{hmgr} - v_{mevk}$$

$$\frac{d}{dt}mev5p_{cyt} = +v_{mevk} - v_{pmevk}$$

$$\frac{d}{dt}mev5pp_{cyt} = +v_{pmevk} - v_{mdp}$$

$$\frac{d}{dt}na_{cyt} = 0$$

$$\frac{d}{dt}na_{mito} = +\frac{I_{na}^{pump} + I_{na_{ed}}}{10 \cdot F \cdot Vol_{mito}}$$

$$\frac{d}{dt}nad_{cyt} = -v_{gapdh} + v_{ldh} - v_{mdh} + v_{g3pdh} - v_{galdh} - v_{guldh} - v_{alddhI} - v_{alddhII} - v_{adh} + v_{sordh} - v_{alddh-gra} + v_{sqs-nadh} - \frac{Vol_{er}}{Vol_{cyt}} \cdot v_{casdc-dmtmasca} - \frac{Vol_{er}}{Vol_{cyt}} \cdot v_{casdc-ddimtmasca} - \frac{Vol_{er}}{Vol_{cyt}} \cdot v_{casdc-dmdihtmasca} - \frac{Vol_{er}}{Vol_{cyt}} \cdot v_{casdc-ddimdihtmasca}$$

$$\frac{d}{dt}nad_{mito} = -v_{3hdh-lc4} - v_{3hdh-lc5} - v_{3hdh-lc6} - v_{3hdh-lc8} - v_{3hdh-lc10} - v_{3hdh-lc12} - v_{3hdh-lc14} - v_{3hdh-lc16} - v_{pdhc} - v_{idh} - v_{kgdhc} - v_{mdh_{mito}} + v_{tdh} + \frac{v_{cxI}}{10 \cdot F \cdot Vol_{mito}} - v_{gdh} + v_{\beta hdh} - v_{alddh_{mito}} - v_{3hdh-lvalcoa}$$

$$\frac{d}{dt}nadh_{cyt} = +v_{gapdh} - v_{ldh} + v_{mdh} - v_{g3pdh} + v_{guldh} + v_{galdh} + v_{alddhI} + v_{alddhII} + v_{adh} - v_{sordh} + v_{alddh-gra} - v_{sqs-nadh} + \frac{Vol_{er}}{Vol_{cyt}} \cdot v_{casdc-dmtmasca} + \frac{Vol_{er}}{Vol_{cyt}} \cdot v_{casdc-ddimtmasca} + \frac{Vol_{er}}{Vol_{cyt}} \cdot v_{casdc-dmdihtmasca} + \frac{Vol_{er}}{Vol_{cyt}} \cdot v_{casdc-ddimdihtmasca}$$

$$\frac{d}{dt}nadh_{mito} = +v_{3hdh-lc4} + v_{3hdh-lc5} + v_{3hdh-lc6} + v_{3hdh-lc8} + v_{3hdh-lc10} + v_{3hdh-lc12} + v_{3hdh-lc14} + v_{3hdh-lc16} + v_{pdhc} + v_{idh} + v_{kgdhc} + v_{mdh_{mito}} - v_{tdh} - \frac{v_{cxI}}{10 \cdot F \cdot Vol_{mito}} + v_{gdh} - v_{\beta hdh} + v_{alddh_{mito}} + v_{3hdh-lvalcoa}$$

$$\frac{d}{dt}nadp_{cyt} = +2 \cdot v_{fas-c4} + 2 \cdot v_{fas-c6} + 2 \cdot v_{fas-c8} + 2 \cdot v_{fas-c10} + 2 \cdot v_{fas-c12} + 2 \cdot v_{fas-c14} + 2 \cdot v_{fas-c16} - v_{g6pdh} - v_{pgdh} - v_{me} + v_{aldor} + v_{aldr} + 2 \cdot v_{hmgr} + v_{sqs-nadph} + v_{sqe} + 3 \cdot \frac{Vol_{er}}{Vol_{cyt}} \cdot v_{s14dm-lan} + 3 \cdot \frac{Vol_{er}}{Vol_{cyt}} \cdot v_{s14dm-dihlan} + \frac{Vol_{er}}{Vol_{cyt}} \cdot v_{s14r-ffmas} + \frac{Vol_{er}}{Vol_{cyt}} \cdot v_{s14r-dihffmas} + 3 \cdot \frac{Vol_{er}}{Vol_{cyt}} \cdot v_{mso-tmas} + 3 \cdot \frac{Vol_{er}}{Vol_{cyt}} \cdot v_{mso-dmtmas} + 3 \cdot \frac{Vol_{er}}{Vol_{cyt}} \cdot v_{mso-dihtmas} + 3 \cdot \frac{Vol_{er}}{Vol_{cyt}} \cdot v_{mso-dmdihtmas} + \frac{Vol_{er}}{Vol_{cyt}} \cdot v_{3ksr-dmktmas} + \frac{Vol_{er}}{Vol_{cyt}} \cdot v_{3ksr-ddimktmas} + \frac{Vol_{er}}{Vol_{cyt}} \cdot v_{3ksr-ddimkdihtmas} + \frac{Vol_{er}}{Vol_{cyt}} \cdot v_{3ksr-dmkdihtmas} + \frac{Vol_{er}}{Vol_{cyt}} \cdot v_{lathox-lath} + \frac{Vol_{er}}{Vol_{cyt}} \cdot v_{lathox-dhlath} + \frac{Vol_{er}}{Vol_{cyt}} \cdot v_{s7r-dhchol} + \frac{Vol_{er}}{Vol_{cyt}} \cdot v_{s7r-dhdesm} + \frac{Vol_{er}}{Vol_{cyt}} \cdot v_{s24r-lan} + \frac{Vol_{er}}{Vol_{cyt}} \cdot v_{s24r-ffmas} + \frac{Vol_{er}}{Vol_{cyt}} \cdot v_{s24r-tmas} + \frac{Vol_{er}}{Vol_{cyt}} \cdot v_{s24r-zym} + \frac{Vol_{er}}{Vol_{cyt}} \cdot v_{s24r-dhlath} + \frac{Vol_{er}}{Vol_{cyt}} \cdot v_{s24r-dhdesmo} + \frac{Vol_{er}}{Vol_{cyt}} \cdot v_{s24r-desmo}$$

$$\frac{d}{dt}nadp_{mito} = -v_{tdh} - v_{gdh-nadp}$$

$$\frac{d}{dt}nadph_{cyt} = -2 \cdot v_{fas-c4} - 2 \cdot v_{fas-c6} - 2 \cdot v_{fas-c8} - 2 \cdot v_{fas-c10} - 2 \cdot v_{fas-c12} - 2 \cdot v_{fas-c14} - 2 \cdot v_{fas-c16} + v_{g6pdh} + v_{pgdh} + -v_{me} - v_{aldor} - v_{aldr} - 2 \cdot v_{hmgr} + v_{sqs-nadph} - v_{sqe} - 3 \cdot \frac{Vol_{er}}{Vol_{cyt}} \cdot v_{s14dm-lan} - 3 \cdot \frac{Vol_{er}}{Vol_{cyt}} \cdot v_{s14dm-dihlan} - \frac{Vol_{er}}{Vol_{cyt}} \cdot v_{s14r-ffmas} - \frac{Vol_{er}}{Vol_{cyt}} \cdot v_{s14r-dihffmas} - 3 \cdot \frac{Vol_{er}}{Vol_{cyt}} \cdot v_{mso-tmas} - 3 \cdot \frac{Vol_{er}}{Vol_{cyt}} \cdot v_{mso-dmtmas} - 3 \cdot \frac{Vol_{er}}{Vol_{cyt}} \cdot v_{mso-dihtmas} - 3 \cdot \frac{Vol_{er}}{Vol_{cyt}} \cdot v_{mso-dmdihtmas} - \frac{Vol_{er}}{Vol_{cyt}} \cdot v_{3ksr-dmktmas} - \frac{Vol_{er}}{Vol_{cyt}} \cdot v_{3ksr-ddimktmas} - \frac{Vol_{er}}{Vol_{cyt}} \cdot v_{3ksr-ddimkdihtmas} - \frac{Vol_{er}}{Vol_{cyt}} \cdot v_{3ksr-dmkdihtmas} - \frac{Vol_{er}}{Vol_{cyt}} \cdot v_{lathox-lath} - \frac{Vol_{er}}{Vol_{cyt}} \cdot v_{lathox-dhlath} - \frac{Vol_{er}}{Vol_{cyt}} \cdot v_{s7r-dhchol} - \frac{Vol_{er}}{Vol_{cyt}} \cdot v_{s7r-dhdesm} - \frac{Vol_{er}}{Vol_{cyt}} \cdot v_{s24r-lan} - \frac{Vol_{er}}{Vol_{cyt}} \cdot v_{s24r-ffmas} - \frac{Vol_{er}}{Vol_{cyt}} \cdot v_{s24r-tmas} - \frac{Vol_{er}}{Vol_{cyt}} \cdot v_{s24r-zym} - \frac{Vol_{er}}{Vol_{cyt}} \cdot v_{s24r-dhlath} - \frac{Vol_{er}}{Vol_{cyt}} \cdot v_{s24r-dhdesmo} - \frac{Vol_{er}}{Vol_{cyt}} \cdot v_{s24r-desmo}$$

$$\frac{d}{dt}nadph_{mito} = +v_{tdh} + v_{gdh-nadp}$$

$$\frac{d}{dt}nh3_{cyt} = +v_{nh_3-uptake} - v_{nh_3-diff} + v_{sdh} - v_{gln-syn}$$

$$\frac{d}{dt}nh3_{ext} = 0$$

$$\frac{d}{dt}nh3_{mito} = +\frac{Vol_{cyt}}{Vol_{mito}} \cdot v_{nh_3-diff} + v_{glnase} + v_{gdh} + v_{gdh-nadp} - v_{cps}$$

$$\frac{d}{dt}o2_{cyt} = -\frac{1}{4} \cdot \frac{v_{cxIV}}{10 \cdot F \cdot Vol_{cyt}} + v_{O_{2diff}} - v_{sqe} - 3 \cdot \frac{Vol_{er}}{Vol_{cyt}} \cdot v_{s14dm-lan} - 3 \cdot \frac{Vol_{er}}{Vol_{cyt}} \cdot v_{s14dm-dihlan} - 3 \cdot \frac{Vol_{er}}{Vol_{cyt}} \cdot v_{mso-tmas} - 3 \cdot \frac{Vol_{er}}{Vol_{cyt}} \cdot v_{mso-dmtmas} - 3 \cdot \frac{Vol_{er}}{Vol_{cyt}} \cdot v_{mso-dihtmas} - 3 \cdot \frac{Vol_{er}}{Vol_{cyt}} \cdot v_{mso-dmdihtmas} - \frac{Vol_{er}}{Vol_{cyt}} \cdot v_{lathox-lath} - \frac{Vol_{er}}{Vol_{cyt}} \cdot v_{lathox-dhlath}$$

$$\frac{d}{dt}o2_{ext} = 0$$

$$\frac{d}{dt}oaa_{cyt} = -v_{pepck} + v_{mdh} + v_{asat} + v_{cit-lys}$$

$$\frac{d}{dt}oaa_{mito} = +v_{mdh_{mito}} - v_{pepck_{mito}} + v_{pc} + v_{asat_{mito}} - v_{cs}$$

$$\frac{d}{dt}orn_{cyt} = -\frac{Vol_{mito}}{Vol_{cyt}} \cdot v_{ocT} + v_{argase}$$

$$\frac{d}{dt}orn_{mito} = -v_{otc} + v_{ocT}$$

$$\frac{d}{dt}p_{cyt} = -\frac{v_{P-ex}}{10 \cdot F \cdot Vol_{cyt}} + 2 \cdot v_{ppase} + v_{atp-usage} + v_{fbp2} + v_{fbp1} - v_{gapdh} - v_{malT} - v_{gp} + v_{cit-lys} + v_{acc1} + v_{pap} - v_{pT_{er}} + v_{gln-syn} + v_{gal1pp} + v_{mdp}$$

$$\frac{d}{dt}p_{er} = +v_{Glc6PP_{ER}} + \frac{Vol_{cell}}{Vol_{ER}} \cdot v_{pT_{er}}$$

$$\frac{d}{dt}p_{mito} = -v_{scs-atp} - v_{scs-gtp} - \frac{v_{F0F1}}{10 \cdot F \cdot Vol_{mito}} + \frac{v_{P-ex}}{10 \cdot F \cdot Vol_{mito}} + v_{pc} + \frac{Vol_{cell}}{Vol_{mito}} \cdot v_{malT} + v_{pcc} + v_{cps} + v_{otc}$$

$$\frac{d}{dt}pa_{er} = +\frac{Vol_{cyt}}{Vol_{er}} \cdot v_{agpat} - \frac{Vol_{cyt}}{Vol_{er}} \cdot v_{pap}$$

$$\frac{d}{dt}pep_{cyt} = +v_{pepT} + v_{eno} - v_{pk} + v_{pepck}$$

$$\frac{d}{dt}pep_{mito} = -\frac{Vol_{cyt}}{Vol_{mito}} \cdot v_{pepT} + v_{pepck_{mito}}$$

$$\frac{d}{dt}pg2_{cyt} = +v_{pgm} - v_{eno} + v_{gck}$$

$$\frac{d}{dt}pg3_{cyt} = +v_{pgk} - v_{pgm}$$

$$\frac{d}{dt}pg6_{cyt} = +v_{pgls} - v_{pgdh}$$

$$\frac{d}{dt}pgl6_{cyt} = +v_{g6pdh} - v_{pgls}$$

$$\frac{d}{dt}pp_{cyt} = +v_{ACSL5} + v_{ACSL4} + v_{ACSL1} - v_{ppase} + v_{upgase} + v_{acoa-syn} + v_{ass} + v_{vcs} + v_{acaccoa-syn} + v_{gpps} + v_{fpps} + 2 \cdot v_{sqs-nadph} + 2 \cdot v_{sqs-nadh}$$

$$\frac{d}{dt}propcoa_{mito} = +v_{3kt}^{kc5coa} - v_{pcc} + v_{3kt}^{kvalcoa}$$

$$\frac{d}{dt}pyr_{cyt} = +v_{pk} - v_{ldh} - v_{mal-pyrT} + v_{pyrT} - v_{pyrT_{mito}} + v_{me} + v_{aat} + v_{sdh}$$

$$\frac{d}{dt}pyr_{ext} = 0$$

$$\frac{d}{dt}pyr_{mito} = -v_{pdhc} - v_{pc} + \frac{Vol_{cell}}{Vol_{mito}} \cdot v_{pyrT} + \frac{Vol_{cell}}{Vol_{mito}} \cdot v_{mal-pyrT}$$

$$\frac{d}{dt}q_{mm} = -\frac{Vol_{mito}}{Vol_{membrane}} * v_{ETF-QO} - \frac{Vol_{mito}}{Vol_{membrane}} * v_{succdh} - \frac{v_{cxI}}{10 \cdot F \cdot Vol_{membrane}} + \frac{v_{cxIII}}{10 \cdot F \cdot Vol_{membrane}} + \frac{Vol_{cyt}}{Vol_{membrane}} v_{g3pdh_{mito}}$$

$$\frac{d}{dt}qh2_{mm} = +\frac{Vol_{mito}}{Vol_{membrane}} * v_{ETF-QO} + \frac{Vol_{mito}}{Vol_{membrane}} * v_{succdh} + \frac{v_{cxI}}{10 \cdot F \cdot Vol_{membrane}} - \frac{v_{cxIII}}{10 \cdot F \cdot Vol_{membrane}} - \frac{Vol_{cyt}}{Vol_{membrane}} v_{g3pdh_{mito}}$$

$$\frac{d}{dt}r5p_{cyt} = -v_{rpi} + v_{tketo1}$$

$$\frac{d}{dt}ru5p_{cyt} = +v_{pgdh} - v_{rpe} + v_{rpi}$$

$$\frac{d}{dt}s7p_{cyt} = -v_{taldo} - v_{tketo1}$$

$$\frac{d}{dt}ser_{cyt} = -v_{sdh} + v_{serT}$$

$$\frac{d}{dt}sqe_{cyt} = +v_{sqe} - v_{osc}$$

$$\frac{d}{dt}squ_{cyt} = +v_{sqs-nadph} + v_{sqs-nadh} - v_{sqe}$$

$$\frac{d}{dt}suc_{mito} = +v_{scs-atp} + v_{scs-gtp} - v_{succdh}$$

$$\frac{d}{dt}succoa_{mito} = +v_{kgdhc} - v_{scs-atp} - v_{scs-gtp} + v_{mmm}$$

$$\frac{d}{dt}tag_{er} = +\frac{Vol_{cyt}}{Vol_{er}} \cdot v_{dgat} - v_{MTP}^{tag} - v_{LD-syn-tag}$$

$$\frac{d}{dt}tag_{ld} = +\frac{Vol_{er}}{Vol_{ld}} \cdot v_{LD-syn-tag} - v_{ATGL}^{tag}$$

$$\frac{d}{dt}tag_{vldl} = -v_{VLDL-ex-tag} + \frac{Vol_{er}}{Vol_{vldl}} \cdot v_{MTP}^{tag}$$

$$\frac{d}{dt}tmas_{er} = +v_{s14r-ffmas} - v_{mso-tmas} - v_{s24r-tmas}$$

$$\frac{d}{dt}udp_{cyt} = -v_{ndk_{cyt}}^{udp} + v_{gs}$$

$$\frac{d}{dt}udpglc_{cyt} = +v_{upgase} - v_{gs} + v_{gale} - v_{galt}$$

$$\frac{d}{dt}udpgal_{cyt} = -v_{gale} + v_{galt}$$

$$\frac{d}{dt}urea_{cyt} = +v_{argase} - v_{ureaT}$$

$$\frac{d}{dt}urea_{ext} = 0$$

$$\frac{d}{dt}utp_{cyt} = +v_{ndk_{cyt}}^{udp} - v_{upgase}$$

$$\frac{d}{dt}val_{cyt} = +v_{val-diff} - v_{vcs}$$

$$\frac{d}{dt}val_{ext} = 0$$

$$\frac{d}{dt}valcar_{cyt} = +v_{CPT1-val} - v_{CACT-val}$$

$$\frac{d}{dt}valcar_{mito} = +\frac{Vol_{cyt}}{Vol_{mito}} \cdot v_{CACT-val} - v_{CPT2-val}$$

$$\frac{d}{dt}valcoa_{cyt} = +v_{vcs} - v_{CPT1-val}$$

$$\frac{d}{dt}valcoa_{mito} = +v_{CPT2-val} - v_{valcoa-bcdh}$$

$$\frac{d}{dt}v_{mm} = \frac{10^{-1}}{c_m \cdot A_m} \cdot \left(-I_{C_{ed}} + I_{K_{ed}} + I_{H_{ed}} + I_{Na_{ed}} + I_H^{pump} + v_{ex} + 3 \cdot v_{syn}\right) + \frac{\mathrm{F} \cdot \mathrm{Vol_{cyt}}}{c_m \cdot A_m} \cdot v_{pepT}$$

$$\frac{d}{dt}x5p_{cyt} = +v_{rpe} + v_{tketo1} + v_{tketo2} + v_{xylk}$$

$$\frac{d}{dt} xyl_{cyt} = +v_{galdc} - v_{xylk}$$

$$\frac{d}{dt} zym_{er} = +v_{3ksr-ddimktmas} - v_{s78i-zym} - v_{s24r-zym}$$

$$\frac{d}{dt} zymostenol_{er} = +v_{3ksr-ddimkdihtmas} - v_{s78i-zymostenol} + v_{s24r-zym}$$

Supplementary Note 2 – Construction of Glucose-Hormone Relationships and Diurnal Profiles of Plasma Metabolites and Hormones

Exchangeable metabolites: We used the model to investigate the response of the liver to diurnal variations of the plasma metabolite and hormone levels. The model describes the exchange of 17 metabolites with the plasma. Experimental plasma metabolite profiles monitored over 24 hours were used as model input. As no complete set of diurnal plasma profiles is available, we took plasma profiles from different publications (Supplementary Table 4).

Insulin and glucagon: The plasma concentrations of the two hormones insulin and glucagon determine the phosphorylation state of the inter-convertible enzymes. Both hormones are secreted by the pancreas into the portal vein and the secretion rate is mainly controlled by the glucose concentration of the blood. Therefore we used the empirical glucose hormone transfer function (GHT), which describes the relationship between the plasma level of glucose and the plasma levels of insulin and glucagon previously established in [316].

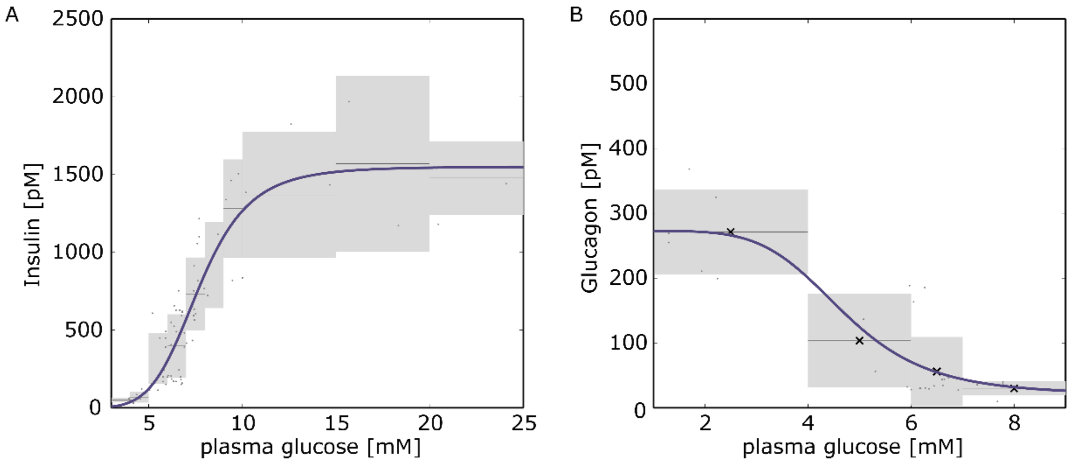

Supplementary Figure 6: The GHT functions describe the dependence of plasma insulin (A) and plasma glucagon (B) on plasma glucose levels. Experimentally determined plasma concentrations of glucose and hormone (grey dots) from various sources (insulin: [317-319], glucagon: [320-324]) were pooled (black lines – mean values, light grey boxes – standard deviations). Periportal hormone concentrations are twice as high as the measured blood plasma concentrations [320]. A Hill-type function was used to fit the data by least-square minimization yielding the GHT function.

$$\text{Ins} = 2 * \left(1.55\ \text{nM} * \frac{(\text{Glc}_{\text{ext}})^{5.7}}{(\text{Glc}_{\text{ext}})^{5.7} + (7.7\ \text{mM})^{5.7}}\right)$$

$$\text{Glucagon} = 2 * \left(0.253\ \text{nM} * \left(1 - \frac{(\text{Glc}_{\text{ext}})^{5.65}}{(\text{Glc}_{\text{ext}})^{5.65} + (4.7\ \text{mM})^{5.65}}\right) + 0.02\ \text{nM}\right)$$

The concentration of the hormones determines the phosphorylation state of the interconvertible enzymes [316]

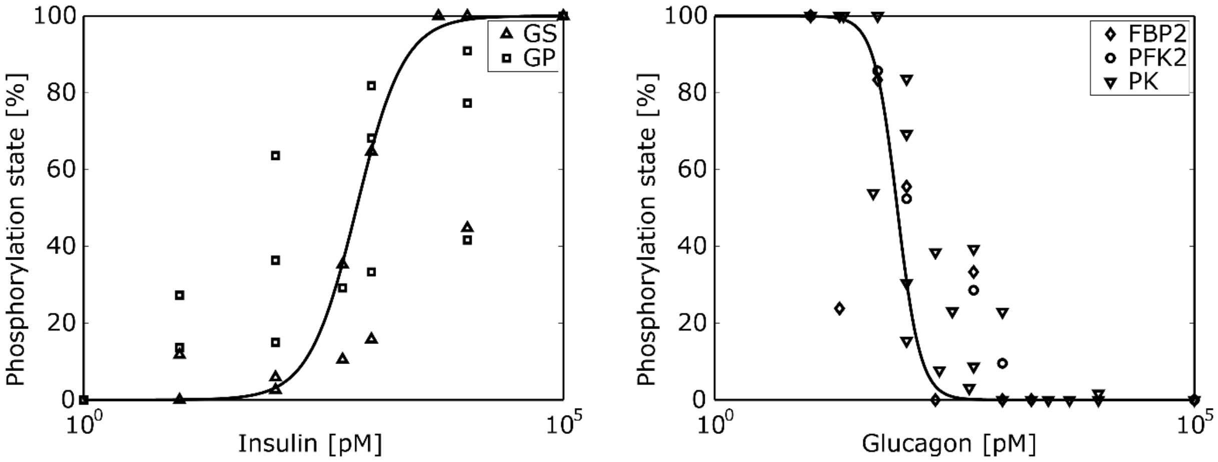

Supplementary Figure 7: Hormone phosphorylation function γ. Bold lines depict the function γ used to relate the level of insulin and glucagon to the phosphorylated form of enzymes regulated by eversible phosphorylation. Experimental data are from various sources [107, 325-331].

$$\gamma = \max\left(min\left(\frac{1}{2} \cdot \left(1 - \frac{Ins^{1.75}}{Ins^{1.75} + (0.70\, nM)^{1.75}} \cdot \left(1 - \frac{c16_{ext}}{c16_{ext} + 0.4mM}\right) + \frac{Glucagon^3}{Glucagon^3 + (0.08\, nM)^3}\right), 0.95\right), 0.05\right)$$

The phosphorylation state of interconvertible enzymes has been limited to be between 5 and 95%.

<u>Free (non-esterified) fatty acids (FFA):</u> The plasma concentration of free fatty acids is largely determined by the rate of triglyceride lipolysis in the adipose tissue which is mainly controlled by insulin and glucagon through the activity of the hormone sensitive lipases (HSL). Based on measured relations between the plasma levels of plasma and FFA we constructed an empirical glucose-FFA transfer function (GFT) (see Supplementary Figure 8).

.

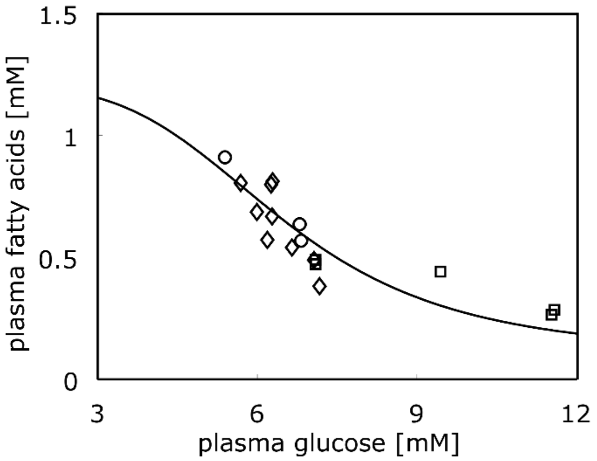

Supplementary Figure 8: *Glucose (glc) free fatty acids (ffa) transfer (GFT) functions for the dependence of plasma ffa levels from plasma glucose level.* Experimentally determined plasma concentration values of glucose and free fatty acids from various sources: [332-334]**.** Hill-type transfer functions were fitted to the data by least-square minimization yielding the GFT.

$$tfa_{plasma} = 1.2mM - 1.1mM \frac{Glc_{ext}^{4}}{Glc_{ext}^{4} + (6.5mM)^{4}}$$

Fatty acid albumin binding

Plasma fatty acids are largely bound to plasma albumin and only free fatty acids are taken up by the liver. We calculated the free fatty acid concentration assuming equilibrium between free fatty acids and albumin bound fatty acids.

The model albumin has 5 different binding sites for fatty acids that can each be occupied independent of the occupation states of the other sites. Albumin has therefore 32 different occupation states. We denote the occupation state as a binary vector (0 – not occupied; 1 – occupied) of length 5.

The fatty acid release rates from any of the 5 different binding sites are given by the following rate equations:

$$v_{alb_{1xxxx}} = V_{max}^{albumin} \cdot \left(alb_{1xxxx} - {}^{1}\!/_{K_{eq}^{alb1}} \cdot c16_{ext} \cdot alb_{0xxxx} \right)$$

$$v_{alb_{x1xxx}} = V_{max}^{albumin} \cdot \left(alb_{x1xxx} - {}^{1}\!/_{K_{eq}^{alb2}} \cdot c16_{ext} \cdot alb_{x0xxx} \right)$$

$$v_{alb_{xx1xx}} = V_{max}^{albumin} \cdot \left(alb_{xx1xx} - {}^{1}\!/_{K_{eq}^{alb3}} \cdot c16_{ext} \cdot alb_{xx0xx} \right)$$

$$v_{alb_{xxx1x}} = V_{max}^{albumin} \cdot \left(alb_{xxx1x} - {}^{1}\!/_{K_{eq}^{alb4}} \cdot c16_{ext} \cdot alb_{xxx0x} \right)$$

$$v_{alb_{xxxx1}} = V_{max}^{albumin} \cdot \left(alb_{xxxx1} - {}^{1}\!/_{K_{eq}^{alb5}} \cdot c16_{ext} \cdot alb_{xxxx0} \right)$$

$K_{eq}^{alb1} = 6.2 \cdot 10^{4}$ [335]

$K_{eq}^{alb2} = 2.3 \cdot 10^{4}$ [335]

$K_{eq}^{alb3} = 1.2 \cdot 10^{4}$ [335]

$K_{eq}^{alb4} = 3.1 \cdot 10^{3}$ [335]

$K_{eq}^{alb5} = 1.5 \cdot 10^{3}$ [335]

The binary variables x = [0,1] denote the occupation state of the respective binding site (1-5). If not further specified x means both occupied and non-occupied. The total release rate is obtained by summing up the elementary release rates for the individual sites:

$$v_{c16_{ext}} = \sum_{x_2,x_3,x_4,x_5} v_{alb_{1,x_2,x_3,x_4,x_5}} + \cdots + v_{alb_{x_1,x_2,x_3,x_4,1}}$$

Varying the external fatty acids between 0 and 1.2 mM and calculating the equilibrium free fatty acid concentration results in the relationship shown in Supplementary Figure 9. We used a fourth order polynomial fit function to calculate the free fatty acids (ffa) from the total fatty acids (tfa) in the plasma (r=1.0).

$$\frac{ffa_{plasma}}{[nM]} = 27.86\left(\frac{tfa_{plasma}}{[mM]}\right)^4 - 18.29\left(\frac{tfa_{plasma}}{[mM]}\right)^3 + 30.88\left(\frac{tfa_{plasma}}{[mM]}\right)^2 + 17.83\left(\frac{tfa_{plasma}}{[mM]}\right)$$

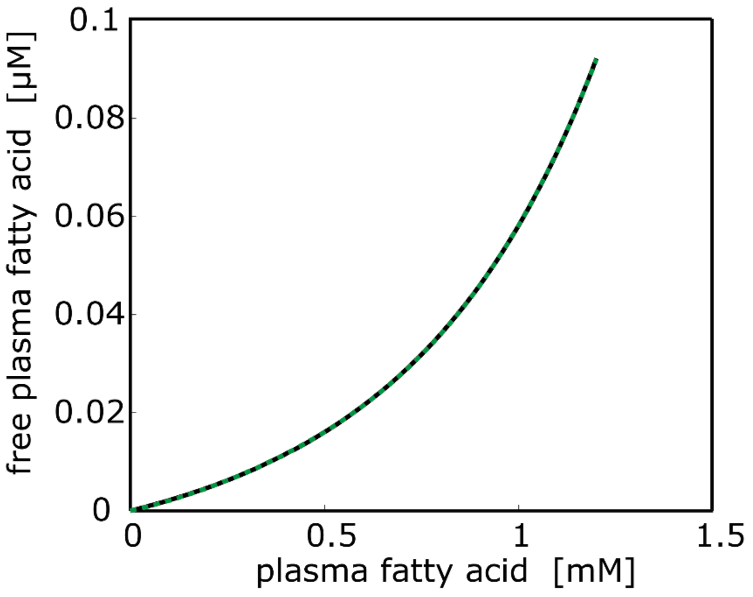

Supplementary Figure 9: A plasma albumin concentration of 0.5 mM was used according to [336]

Diurnal plasma metabolite concentrations

**Metabolite**	**Mean value**	**Maximum value**	**Minimum value**	**Ref.**
glucose [mM]	7.7	9.4	6.4	[333]
glycerol [mM]	0.066	0.092	0.047	[337]
Pyruvate [mM]	0.183	0.232	0.135	[338]
lactate [mM]	1.73	2.19	1.27	[339]
acetoacetate [mM]	0.11	0.13	0.087	[332]
β-hydroxyburerate [mM]	0.072	0.090	0.051	[332]
fatty acids [mM]	0.50	0.67	0.30	GFT
oxygen [Torr]	149	163	138	[340]
ammonia [mM]	0.20	0.26	0.17	[341]
glutamine [mM]	0.88	0.938	0.76	[342]
glutamate [mM]	0.104	0.135	0.085	[343]
alanine [mM]	0.575	0.64	0.495	[343]
serine [mM]	0.288	0.323	0.241	[343]
Insulin [pM]	1476	2358	791	GHT
Glucagon [pM]	80.1	116.4	49.7	GHT

Supplementary Table 4: Maximal, minimal and mean values for diurnal plasma metabolites

	metabolic function	varied		metabolic function	varied
	lipid metabolism			***energy metabolism***	
1	fatty acid uptake	fatty acids	12	adenine nucleotides	oxygen
2	production of ketone bodies	fatty acids	13	oxygen consumption	oxygen
3	VLDL secretion	fatty acids	14	reduction state of respiratory chain	oxygen
4	VLDL secretion	insulin		***carbohydrate metabolism***	
5	triglyceride synthesis	fatty acids	15	gluconeogenesis	lactate
6	triglyceride synthesis	glucose	16	gluconeogenesis	oxygen
7	glycerol uptake	glycerol	17	glucose uptake and glucose production	glucose
8	cholesterol synthesis	glucose	18	glycogen turnover	fasting - feeding
	ammonia detoxification		19	fructose uptake	fructose
9	ammonia uptake & urea synthesis	ammonia	20	galactose uptake	galactose
10	urea synthesis	oxygen		***ethanol detoxification***	
11	serine uptake	serine	21	ethanol uptake	etanol

Overview of simulated experiments used for model calibration

Except experiment #18 (glycogen turnover) all experiments and hence the related model simulations were conducted under quasi-stationary conditions. The perturbation experiment was initialized with a perfused organ or isolated liver cells that have already established a stationary metabolic state after perfusion/incubation with a defined medium. The response to the perturbation (change in the external concentration of a hormone or an exchangeable metabolite) was monitored over a time span that was much longer than the time span (several minutes) required for the establishment of a new stationary state.

Lipid metabolism

#1 Fatty acid uptake

Simulation Stationary uptake rate uptake of free fatty acids (FFAs) from the blood plasma /medium in dependence of free fatty acid supply

Physiological relevance The liver has a central role in converting FFAs taken up from the plasma into triglycerides (esterification of FFAs with glycerol), degrading FFAs to acetyl-CoA used as substrate of the citric acid cycle, the synthesis of ketone bodies and the synthesis of cholesterol.

Experiments Single-pass perfused liver [344]; primary culture with minimum essential medium [345]; perfused liver [346]

External Conditions/Initial Values

Glucose [mM]	Galactose [mM]	Fructose [mM]	Lactate [mM]	Pyruvate [mM]	Glycerol [mM]	Acetate [mM]	Ethanol [mM]	fatty acids [mM]
0.03	0	0	1	0.1	0.0676	0.5	0	0.01-1

Acetoacetate [mM]	β-hydroxybuterate [mM]	Oxygen [mmHg]	Ammonia [mM]	Glutamat [mM]	Glutamin [mM]	Serine [mM]	Alanine [mM]	Insulin [pM]	Glucagon [pM]
0.11	0.07	66	0.025	0.03	0.9	0.3	0.6	1	1

Comparison of model simulation with experimental data

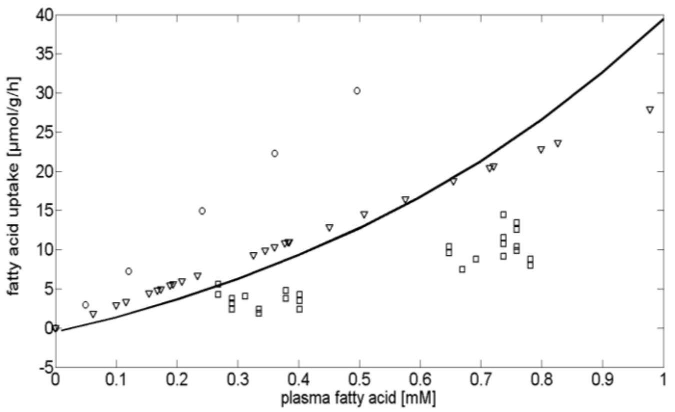

Supplementary Figure 10: Uptake rate of palmitate as function of the external palmitate concentration. Experimental data from [344] (o), [346] (□),[345] (Δ)

#2 Ketone body production

Simulation Stationary production rate of ketone bodies in dependence of external free fatty acids

Physiological relevance Hepatic provision of ketone bodies for systemic energy metabolism, in particular for muscle and brain in starvation

Experiments Perfused liver [346-349]

External Conditions/Initial Values

Glucose [mM]	Galactose [mM]	Fructose [mM]	Lactate [mM]	Pyruvate [mM]	Glycerol [mM]	Acetate [mM]	Ethanol [mM]	fatty acids [mM]
0.03	0	0	1	0.1	0.0676	0.5	0	0.01-1

Acetoacetate [mM]	β-hydroxybuterate [mM]	Oxygen [mmHg]	Ammonia [mM]	Glutamat [mM]	Glutamin [mM]	Serine [mM]	Alanine [mM]	Insulin [pM]	Glucagon [pM]
0.11	0.07	66	0.025	0.03	0.9	0.3	0.6	1	1

Comparison of model simulation with experimental data

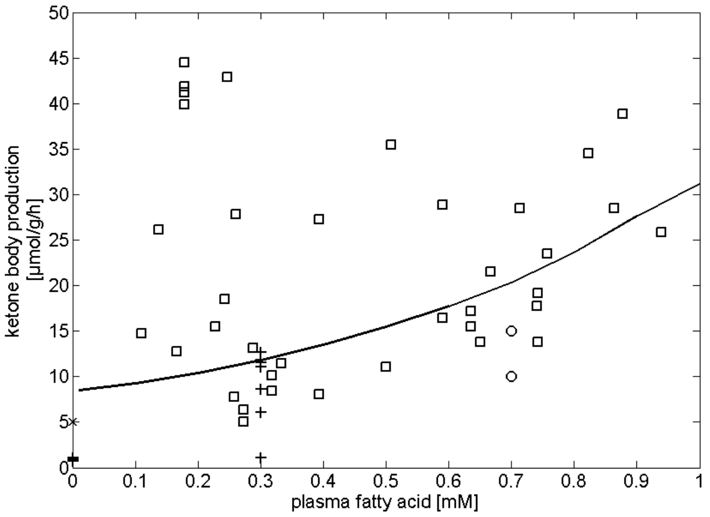

Supplementary Figure 11: Production rate of ketone bodies as function of the external palmitate concentration. Experimental data from [349] (o), [346] (□), [347] (+), [348] (x)

#3 Release of TAG stored in VLDL lipoproteins

Simulation Stationary release rate of VLDL-TAG in dependence of palmitate uptake

Physiological relevance Export of TAG and cholesterol packed in VLDL lipoproteins to extra-hepatic organs

Experiments Perfused liver [350, 351]

External Conditions/Initial Values

Glucose [mM]	Galactose [mM]	Fructose [mM]	Lactate [mM]	Pyruvate [mM]	Glycerol [mM]	Acetate [mM]	Ethanol [mM]	fatty acids [mM]
0.03	0	0	1	0.1	0.0676	0.5	0	0.01-1

Acetoacetate [mM]	β-hydroxybuterate [mM]	Oxygen [mmHg]	Ammonia [mM]	Glutamat [mM]	Glutamin [mM]	Serine [mM]	Alanine [mM]	Insulin [pM]	Glucagon [pM]
0.11	0.07	66	0.025	0.03	0.9	0.3	0.6	1	1

Comparison of model simulation with experimental data

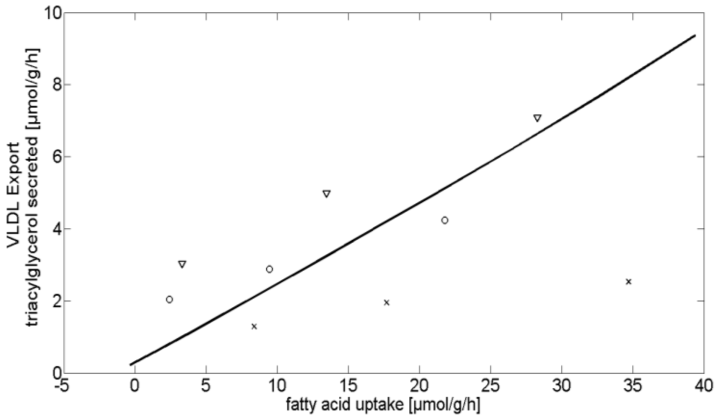

Supplementary Figure 12: Secretion rate of VLDL-TAG as function of the palmitate uptake rate [351] male (o), female (∇),[350] (x)

#4 Secretion rate of VLDL-ApoB100

Simulation Stationary secretion rate of VLDL-ApoB100 in dependence of external insulin concentration

Physiological relevance Export of TAG and cholesterol packed in VLDL lipoproteins to extra-hepatic organs

Experiments Cultures of isolated hepatocytes [232]

External Conditions/Initial Values

Glucose [mM]	Galactose [mM]	Fructose [mM]	Lactate [mM]	Pyruvate [mM]	Glycerol [mM]	Acetate [mM]	Ethanol [mM]	fatty acids [mM]
5	0	0	1	0.1	0.0676	0.5	0	0.5

Acetoacetate [mM]	β-hydroxybuterate [mM]	Oxygen [mmHg]	Ammonia [mM]	Glutamat [mM]	Glutamin [mM]	Serine [mM]	Alanine [mM]	Insulin [nM]	Glucagon [pM]
0.11	0.07	66	0.025	0.03	0.9	0.3	0.6	0-181	0

Comparison of model simulation with experimental data

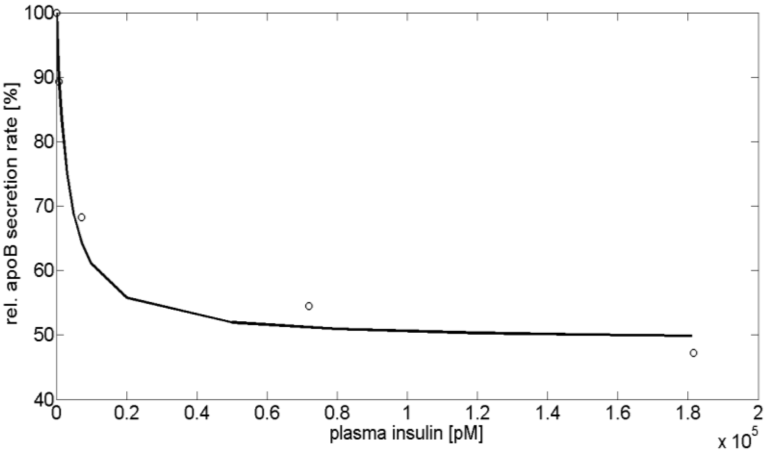

Supplementary Figure 13: Secretion rate of VLDL-ApoB100 as function of the external insulin concentration

#5 Cellular TAG accumulation

Simulation Cellular TAG content as function of the uptake rate of FFAs (palmitate)

Physiological relevance TAG stored in lipid droplets (LDs) prevents lipotoxicity of an excess of non-esterified fatty acids and may deliver FFAs by lipolysis if needed for the production of ATP, synthesis of VLDL-TAG, ketone bodies, cholesterol.

Experiments Perfused liver [351]

External Conditions/Initial Values

Glucose [mM]	Galactose [mM]	Fructose [mM]	Lactate [mM]	Pyruvate [mM]	Glycerol [mM]	Acetate [mM]	Ethanol [mM]	Free fatty acids [mM]
0.1	0	0	1	0.15	0.2	0.5	0	0-1.2

Acetoacetate [mM]	β-hydroxybuterate [mM]	Oxygen [mmHg]	Ammonia [mM]	Glutamat [mM]	Glutamin [mM]	Serine [mM]	Alanine [mM]	Insulin [pM]	Glucagon [pM]
0.1	0.06	66	0.025	0.03	0.75	0.1	0.3	1	1

Comparison of model simulation with experimental data

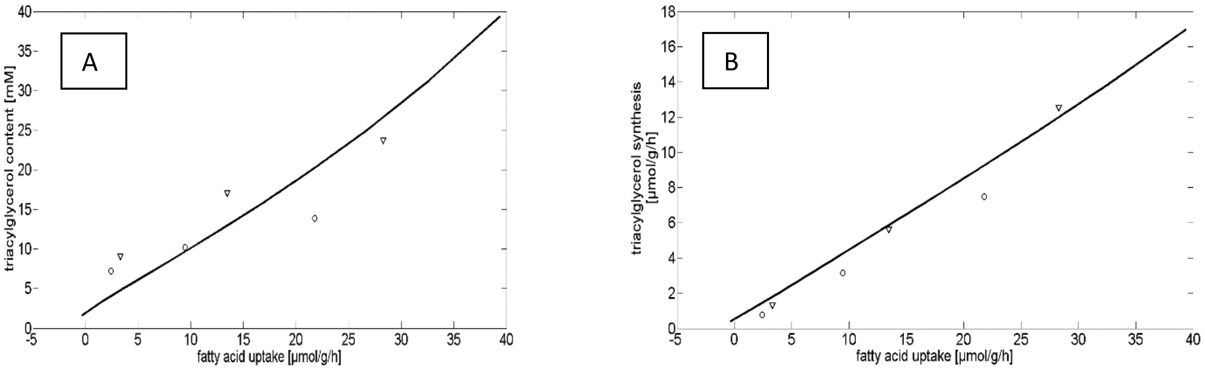

Supplementary Figure 14: Cellular TAG content (A) and TAG synthesis rate (B) as function of the uptake rate of FFAs (palmitate). [351] female (∇),male (o)

#6 De novo TAG synthesis

Simulation Rate of TAG synthesis as function of the external glucose

Physiological relevance During hyperglycemia the liver takes up excess glucose and converts it into lipids to avoid hyperglycemia and to save the energy in form of lipids

Experiments: Perfused liver experiments [352, 353] yield rates of 2-10 µmol/g/h

External Conditions/Initial Values

Glucose [mM]	Galactose [mM]	Fructose [mM]	Lactate [mM]	Pyruvate [mM]	Glycerol [mM]	Acetate [mM]	Ethanol [mM]	Free fatty acids [mM]
3-12	0	0	1.7257	0.15	0.0676	0	0	TF

Acetoacetate [mM]	β-hydroxybuterate [mM]	Oxygen [mmHg]	Ammonia [mM]	Glutamat [mM]	Glutamin [mM]	Serine [mM]	Alanine [mM]	Insulin [nM]	Glucagon [pM]
0.1105	0.07	66	0.02	0.1	1	0.3	0.6	TF	TF

Comparison of model simulation with experimental data

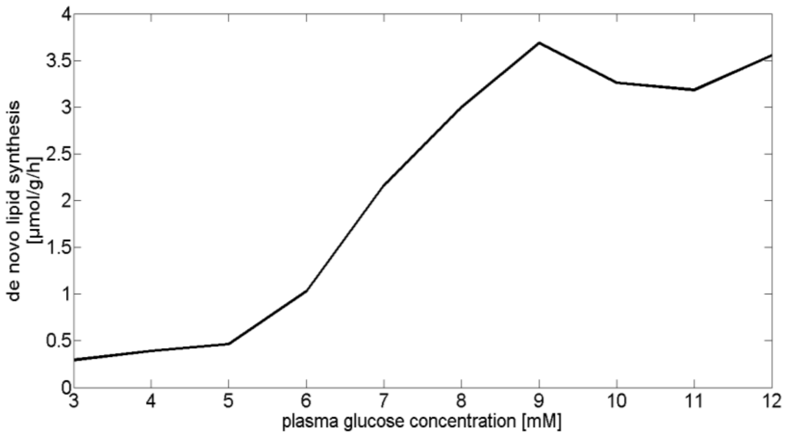

Supplementary Figure 15: Rate of TAG de novo synthesis as function of external glucose concentration

#7 Glycerol uptake

Simulation of stationary rate of glycerol uptake as function of external glycerol

Physiological relevance The liver takes up glycerol as substrate for gluconeogenesis and for the synthesis of triacylglycerides

Experiments: Perfused liver [186, 354]

External Conditions/Initial Values

Glucose [mM]	Galactose [mM]	Fructose [mM]	Lactate [mM]	Pyruvate [mM]	Glycerol [mM]	Acetate [mM]	Ethanol [mM]	Free fatty acids [mM]
0	0	0	0.1	0.1	0-10	0	0	0

Acetoacetate [mM]	β-hydroxybuterate [mM]	Oxygen [mmHg]	Ammonia [mM]	Glutamat [mM]	Glutamin [mM]	Serine [mM]	Alanine [mM]	Insulin [pM]	Glucagon [pM]
0	0	66	0.025	0	0	0	0	1	1

Comparison of model simulation with experimental data

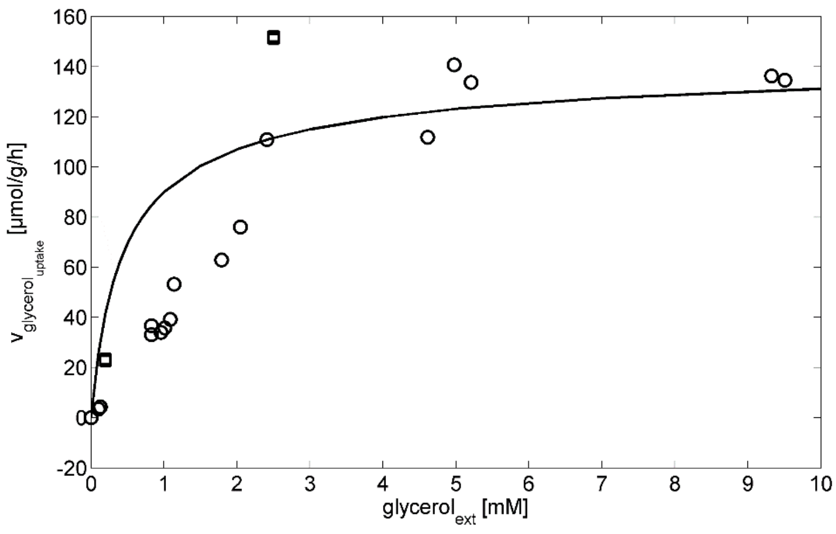

Supplementary Figure 16: Uptake rate of glycerol at various external concentrations of glycerol. Data from [354] (o) [186] (□)

#8 Cholesterol synthesis

Simulation Rate of cholesterol synthesis at varying external concentrations of glucose

Physiological relevance The liver is the main producer of cholesterol. Acetyl-CoA formed from glucose via the PDH reaction is partially converted into TAG and cholesterol.

Experiments Perfused liver

External Conditions/Initial Values

Glucose [mM]	Galactose [mM]	Fructose [mM]	Lactate [mM]	Pyruvate [mM]	Glycerol [mM]	Acetate [mM]	Ethanol [mM]	Free fatty acids [mM]
3-12	0	0	1.7257	0.15	0.0676	0	0	TF

Acetoacetate [mM]	β-hydroxybuterate [mM]	Oxygen [mmHg]	Ammonia [mM]	Glutamat [mM]	Glutamin [mM]	Serine [mM]	Alanine [mM]	Insulin [nM]	Glucagon [pM]
0.1105	0.07	66	0.02	0.1	1	0.3	0.6	TF	TF

Comparison of model simulation with experimental data

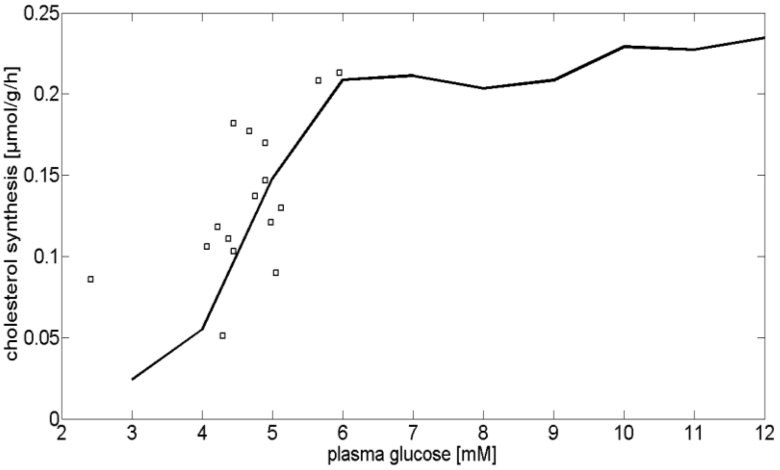

Supplementary Figure 17: Rate of cholesterol synthesis as function of the external glucose concentration. Data from [355]

Nitrogen & ethanol metabolism

#9 Ammonia uptake and urea synthesis

Simulation Stationary rate of urea synthesis as function of external ammonia

Physiological relevance: Conversion of ammonia (NH_4) into urea is a central detoxifying function of the liver. Ammonia taken up from the plasma stems from the amino acid metabolism of the enterocytes and the nitrogen metabolism of bacteria resident in the gut.

Experiments: Perfused liver [356]

External Conditions/Initial Values

Glucose [mM]	Galactose [mM]	Fructose [mM]	Lactate [mM]	Pyruvate [mM]	Glycerol [mM]	Acetate [mM]	Ethanol [mM]	Free fatty acids [mM]
5	0	0	1	0.1	0	0.5	0	0

Acetoacetate [mM]	β-hydroxybuterate [mM]	Oxygen [mmHg]	Ammonia [mM]	Glutamat [mM]	Glutamin [mM]	Serine [mM]	Alanine [mM]	Insulin [pM]	Glucagon [pM]
0.1	0.06	66	0.01-6	0	0	0	0	1	1

Comparison of model simulation with experimental data

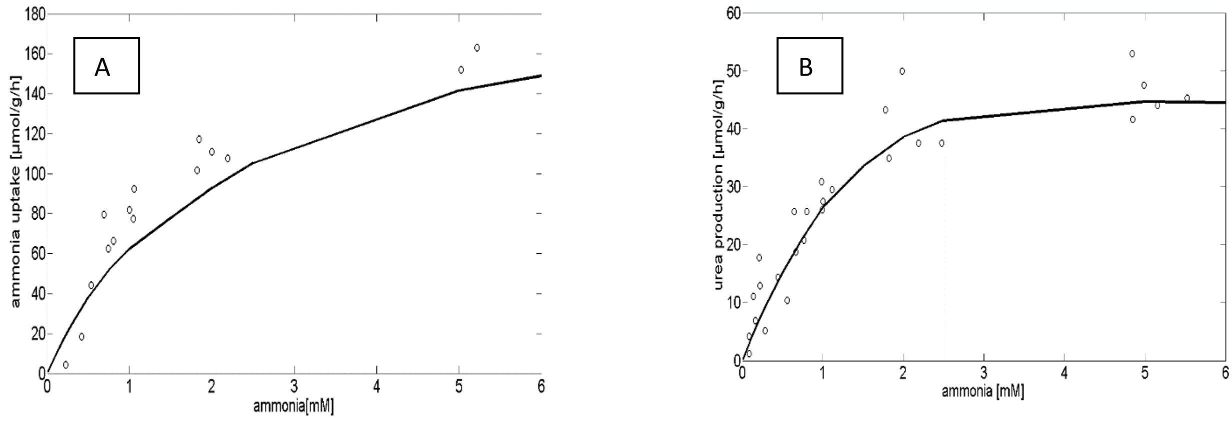

Supplementary Figure 18: Uptake rate of ammonia (A) and urea production rate (B) as function of the external ammonia concentration

#10 Urea synthesis

Simulation: Rate of urea synthesis and ammonia uptake as function of oxygen supply

Physiological relevance The formation of 1 mol of urea requires 4 mol of ATP thus rendering urea synthesis to one of the major ATP consuming and oxygen-depending pathways.

Experiments: Isolated rat hepatocytes [357]

External Conditions/Initial Values

Glucose [mM]	Galactose [mM]	Fructose [mM]	Lactate [mM]	Pyruvate [mM]	Glycerol [mM]	Acetate [mM]	Ethanol [mM]	Free fatty acids [mM]
37	0	4	1	0.2	0.0676	0	0-10	1.2

Acetoacetate [mM]	β-hydroxybuterate [mM]	Oxygen [mmHg]	Ammonia [mM]	Glutamat [mM]	Glutamin [mM]	Serine [mM]	Alanine [mM]	Insulin [pM]	Glucagon [pM]
0.11	0.07	66	0.025	0.03	0.9	0.3	0.6	1	1

Comparison of model simulation with experimental data

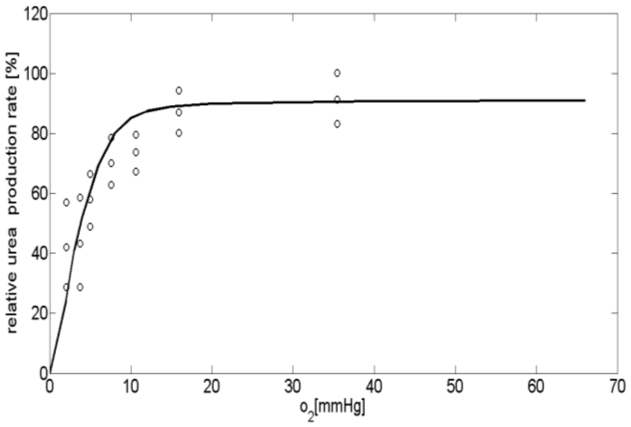

Supplementary Figure 19: Rate of urea synthesis as function of oxygen pressure

#11 Serine uptake

Simulation Stationary rate of serine uptake in dependence of plasma serine

Physiological relevance Serine is after alanine the most important amino acid supplier of carbons for gluconeogenesis and contributes with its amino group significantly to the formation of urea.

Experiments Isolated hepatocytes [228]

External Conditions/Initial Values

Glucose [mM]	Galactose [mM]	Fructose [mM]	Lactate [mM]	Pyruvate [mM]	Glycerol [mM]	Acetate [mM]	Ethanol [mM]	fatty acids [mM]
0	0	0	0	0	0.0676	0	0	0.5

Acetoacetate [mM]	β-hydroxybuterate [mM]	Oxygen [mmHg]	Ammonia [mM]	Glutamat [mM]	Glutamin [mM]	Serine [mM]	Alanine [mM]	Insulin [pM]	Glucagon [pM]
0.11	0.07	66	0.025	0	0	0.1-25	0	1	1

Comparison of model simulation with experimental data

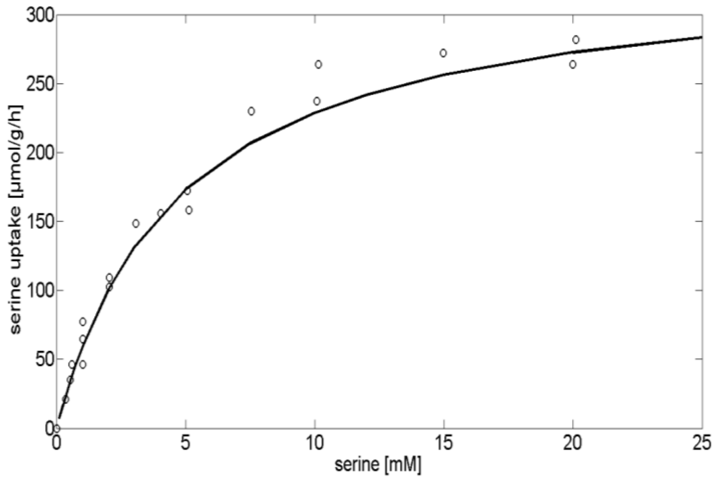

Supplementary Figure 20: Rate of serine uptake as function of external serine concentration.

Energy metabolism

#12 Adenine nucleotide content

Simulation Stationary concentration of ATP, ADP and ADP as function of external oxygen

Physiological relevance Phosphorylation state of adenine nucleotides depends on oxygen availability

Experiments Isolated rat hepatocytes hepatocytes [358, 359]

External Conditions/Initial Values

Glucose [mM]	Galactose [mM]	Fructose [mM]	Lactate [mM]	Pyruvate [mM]	Glycerol [mM]	Acetate [mM]	Ethanol [mM]	Free fatty acids [mM]
37	0	4	1	0.2	0.0676	0	0-10	1.2

Acetoacetate [mM]	β-hydroxybuterate [mM]	Oxygen [mmHg]	Ammonia [mM]	Glutamat [mM]	Glutamin [mM]	Serine [mM]	Alanine [mM]	Insulin [pM]	Glucagon [pM]
0.11	0.07	66	0.025	0.03	0.9	0.3	0.6	1	1

Comparison of model simulation with experimental data

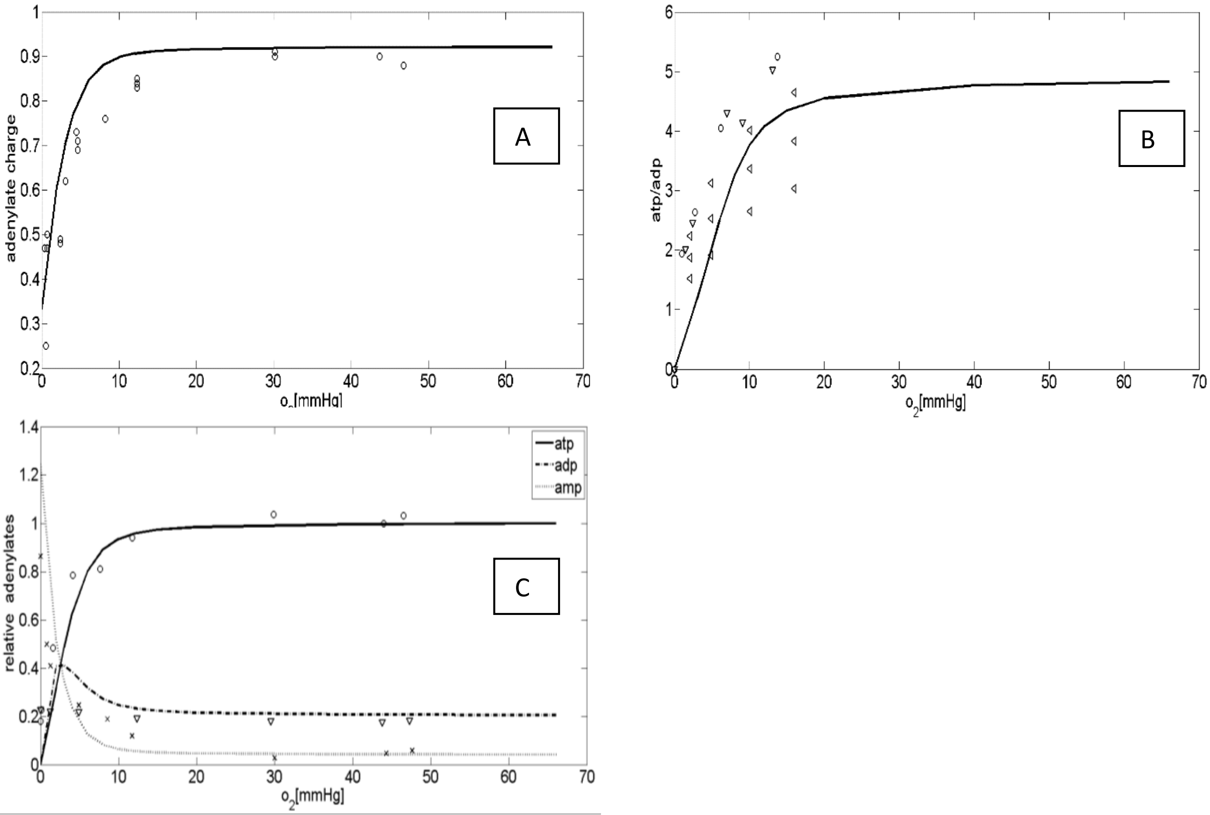

Supplementary Figure 21: Adenylate (energy) charge [ATP+0.5 ADP]/[AMP+ADP+ATP] (A), ATP/ADP ratio (B) and relative levels of ATP, ADP and AMP (C) as function of oxygen pressure. Data are from [359] and in (B) additionally from [358] (o),(∇).

#13 Oxygen uptake

Simulation Stationary rate of oxygen uptake as function of external oxygen. The liver accounts for about 20% of whole body oxygen expenditure

Physiological relevance Liver hypoxia endangers a multitude of ATP-consuming anabolic pathways such as gluconeogenesis or urea synthesis

Experiments Isolated rat hepatocytes [357-359]

External Conditions/Initial Values

Glucose [mM]	Galactose [mM]	Fructose [mM]	Lactate [mM]	Pyruvate [mM]	Glycerol [mM]	Acetate [mM]	Ethanol [mM]	Free fatty acids [mM]
37	0	4	1	0.2	0.0676	0	0-10	1.2

Acetoacetate [mM]	β-hydroxybuterate [mM]	Oxygen [mmHg]	Ammonia [mM]	Glutamat [mM]	Glutamin [mM]	Serine [mM]	Alanine [mM]	Insulin [pM]	Glucagon [pM]
0.11	0.07	66	0.025	0.03	0.9	0.3	0.6	1	1

Comparison of model simulation with experimental data

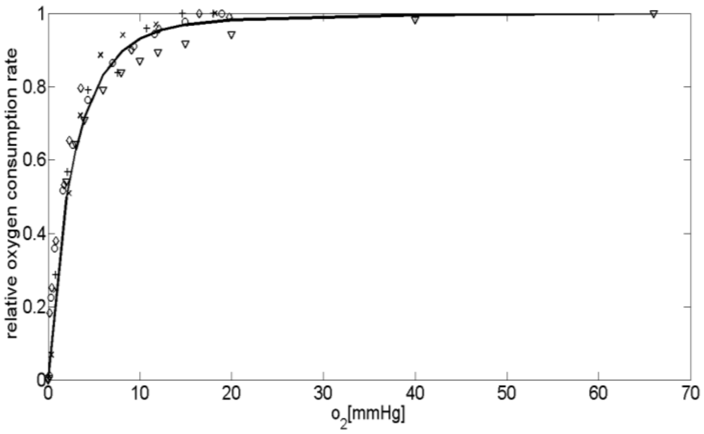

Supplementary Figure 22: Oxygen consumption rate as function of oxygen pressure. Data from [358] (∇), [359] (o), (◊) and (+) and from [357] (x).

#14 Reduction state of respiratory chain

Simulation Reduction state of cytochrome c as function of external oxygen

Physiological relevance Reduction state of the electron carriers in the respiratory chain determines proton motive force but also the production rate of reactive oxygen species (superoxide).

Experiments: Isolated rat hepatocytes [357]

External Conditions/Initial Values

Glucose [mM]	Galactose [mM]	Fructose [mM]	Lactate [mM]	Pyruvate [mM]	Glycerol [mM]	Acetate [mM]	Ethanol [mM]	Free fatty acids [mM]
37	0	4	1	0.2	0.0676	0	0-10	1.2

Acetoacetate [mM]	β-hydroxybuterate [mM]	Oxygen [mmHg]	Ammonia [mM]	Glutamat [mM]	Glutamin [mM]	Serine [mM]	Alanine [mM]	Insulin [pM]	Glucagon [pM]
0.11	0.07	66	0.025	0.03	0.9	0.3	0.6	1	1

Comparison of model simulation with experimental data

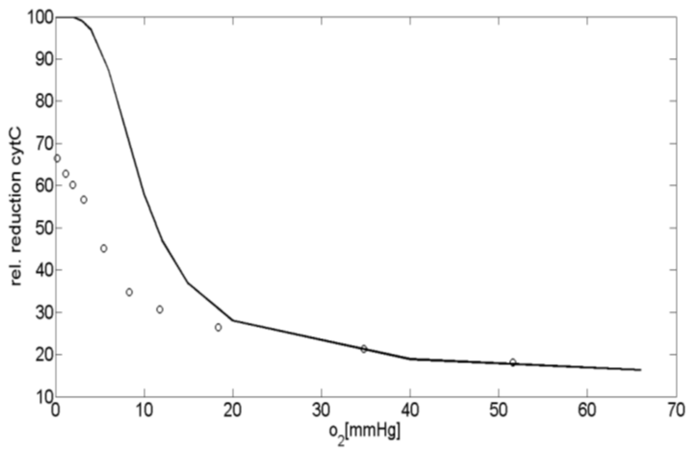

Supplementary Figure 23: Relative reduction state of cyt c as function of oxygen pressure. The discrepancy between model predictions and experimental data at low oxygen pressure suggests the presence of other routes (than transfer to cyt aa3 of complex 4) along which cyt c may release electrons.

Carbohydrate metabolism

#15 Gluconeogenesis

Simulation Stationary rate of gluconeogenesis as function of external lactate

Physiological relevance In the post-absorptive phase and during starvation the liver produces glucose to buffer plasma glucose. Lactate excessively formed during intensive muscle work is a preferred gluconeogenetic substrate.

Experiments Perfused liver [360-362]

External Conditions/Initial Values

Glucose [mM]	Galactose [mM]	Fructose [mM]	Lactate [mM]	Pyruvate [mM]	Glycerol [mM]	Acetate [mM]	Ethanol [mM]	Free fatty acids [mM]
0	0	0	0.1-20	1/7 lactate	0	0	0	0

Acetoacetate [mM]	β-hydroxybuterate [mM]	Oxygen [mmHg]	Ammonia [mM]	Glutamat [mM]	Glutamin [mM]	Serine [mM]	Alanine [mM]	Insulin [pM]	Glucagon [pM]
0.11	0.07	66	0.025	0.03	0.9	0.3	0.6	1	0

Comparison of model simulation with experimental data

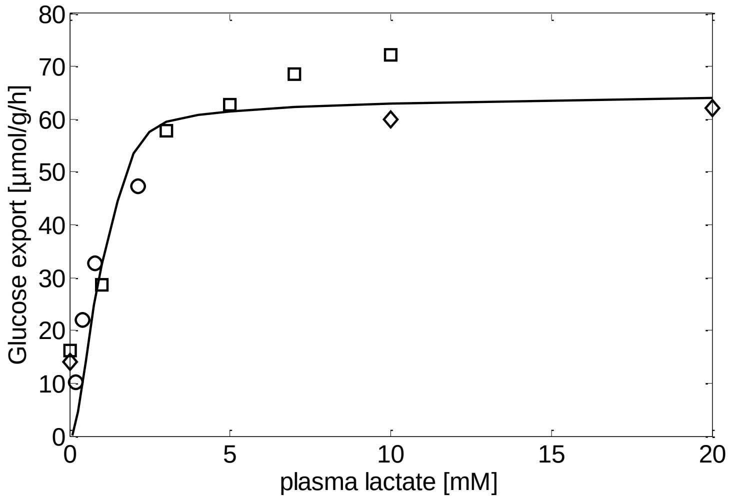

Supplementary Figure 24: Hepatic glucose production (HGP) as function of external lactate concentration. Data from [360] (◊),[362](o) and [361] (□).

#16 Gluconeogenesis

Simulation Stationary rate of gluconeogenesis as function of external oxygen

Physiological relevance Synthesis of 1 mol of glucose from lactate and alanine consumes 4 mol ATP thus rendering gluconeogenesis from these precursors sensitive to hypoxia and related ATP depletion

Experiments Isolated rat hepatocytes [357]

External Conditions/Initial Values

Glucose [mM]	Galactose [mM]	Fructose [mM]	Lactate [mM]	Pyruvate [mM]	Glycerol [mM]	Acetate [mM]	Ethanol [mM]	Free fatty acids [mM]
37	0	4	1	0.2	0.0676	0	0-10	1.2

Acetoacetate [mM]	β-hydroxybuterate [mM]	Oxygen [mmHg]	Ammonia [mM]	Glutamat [mM]	Glutamin [mM]	Serine [mM]	Alanine [mM]	Insulin [pM]	Glucagon [pM]
0.11	0.07	66	0.025	0.03	0.9	0.3	0.6	1	1

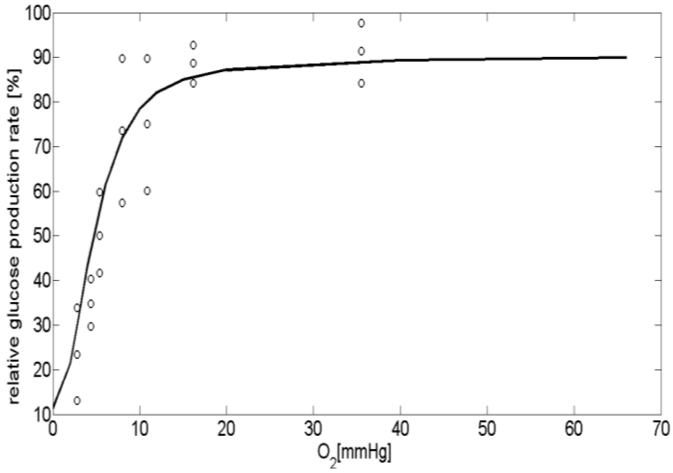

Supplementary Figure 25: Relative hepatic glucose production (HGP) as function of oxygen pressure

#17 Hepatic glucose uptake (HGU) and glucose production (HGP)

Simulation Stationary rate of glucose exchange between liver and plasma as function of external glucose

Physiological relevance The liver ensures homeostasis of plasma glucose by producing glucose during hypoglycemia (gluconeogenesis) and clearing excess glucose from the plasma (conversion to glycogen and lipids) during hyperglycemia

Experiments *In vivo* measurements of blood glucose and liver glucose exchange flux [320, 363-365]

External Conditions/Initial Values

Glucose [mM]	Galactose [mM]	Fructose [mM]	Lactate [mM]	Pyruvate [mM]	Glycerol [mM]	Acetate [mM]	Ethanol [mM]	Free fatty acids [mM]
3-12	0	0	1.7257	0.15	0.0676	0	0	TF

Acetoacetate [mM]	β-hydroxybuterate [mM]	Oxygen [mmHg]	Ammonia [mM]	Glutamat [mM]	Glutamin [mM]	Serine [mM]	Alanine [mM]	Insulin [nM]	Glucagon [pM]
0.1105	0.07	66	0.02	0.1	1	0.3	0.6	TF	TF

Comparison of model simulation with experimental data

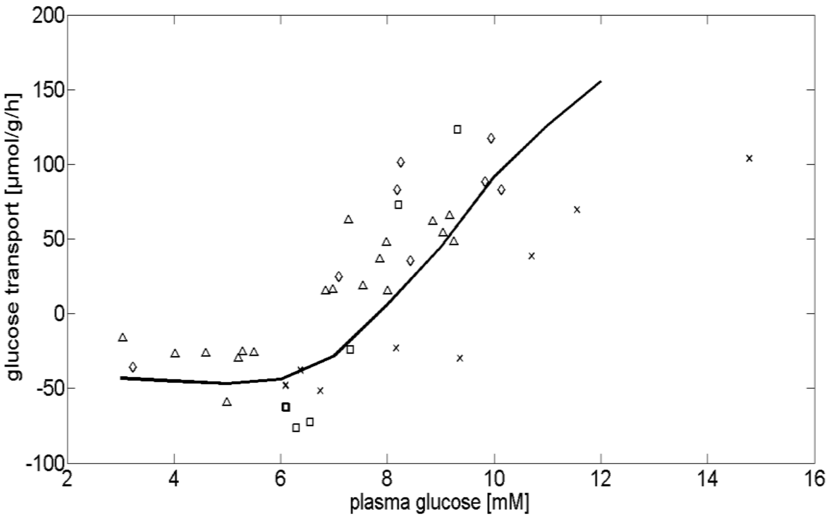

Supplementary Figure 26: Rate of glucose production (HGP = negative values) and glucose uptake (HGU = positive values) as function of plasma glucose. Experimental data from [364] (△), [363](◊), [320](□) and [365] (x)

#18 Diurnal dynamics of hepatic glycogen store

Simulation Temporal changes of glycogen during feeding and subsequent

Physiological relevance Liver glycogen can be rapidly mobilized during fasting and refilled during refeeding. The liver ensures homeostasis of plasma glucose by producing glucose during hypoglycemia (gluconeogenesis) and clearing excess glucose from the plasma (conversion to glycogen and lipids) during hyperglycemia

Experiments Fasted rats (24h food deprivation) were fed for 16h and fasted again for 32h [366]

External Conditions/Initial Values

Glucose [mM]	Galactose [mM]	Fructose [mM]	Lactate [mM]	Pyruvate [mM]	Glycerol [mM]	Acetate [mM]	Ethanol [mM]	Free fatty acids [mM]
8/4	0	0	2	0	0.0676	0.5	0	0.2

Acetoacetate [mM]	β-hydroxybuterate [mM]	Oxygen [mmHg]	Ammonia [mM]	Glutamat [mM]	Glutamin [mM]	Serine [mM]	Alanine [mM]	Insulin [pM]	Glucagon [pM]
0.11	0.07	66	0.025	0.03	0.9	0.3	0.6	TF	TF

Comparison of model simulation with experimental data

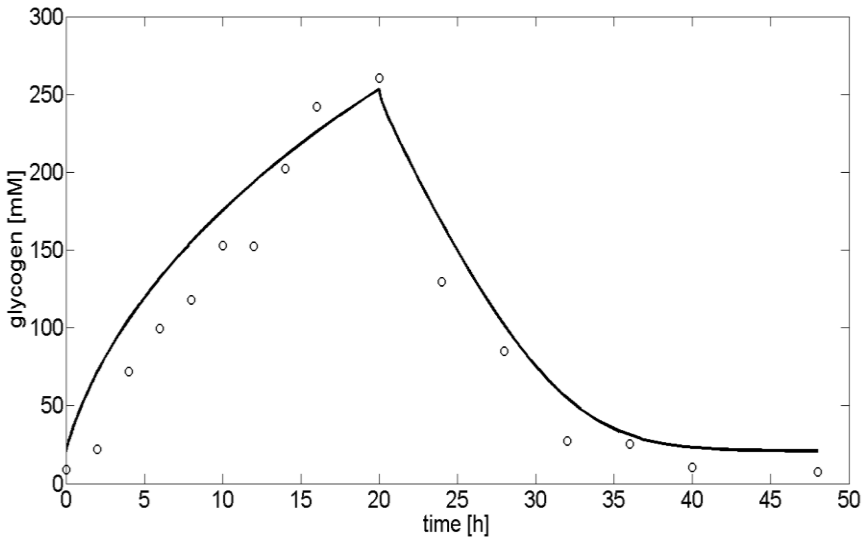

Supplementary Figure 27: Variation of hepatic glycogen during feeding (0-16h) and subsequent fasting.

19 Fructose uptake

Simulation: Stationary rate of fructose uptake as function of external fructose

Physiological relevance: Fructose taken up with fruits and fruit drinks is almost exclusively metabolized by the liver. The metabolism of fructose interferes with the glucose metabolism.

Experiments: Perfused liver [367]

External Conditions/Initial Values

Glucose [mM]	Galactose [mM]	Fructose [mM]	Lactate [mM]	Pyruvate [mM]	Glycerol [mM]	Acetate [mM]	Ethanol [mM]	fatty acids [mM]
0	0	0-160	1	0.1	0.676	0	0	0.2

Acetoacetate [mM]	β-hydroxybuterate [mM]	Oxygen [mmHg]	Ammonia [mM]	Glutamat [mM]	Glutamin [mM]	Serine [mM]	Alanine [mM]	Insulin [pM]	Glucagon [pM]
0.11	0.07	66	0.25	0.03	0.9	0.3	0.6	1	1

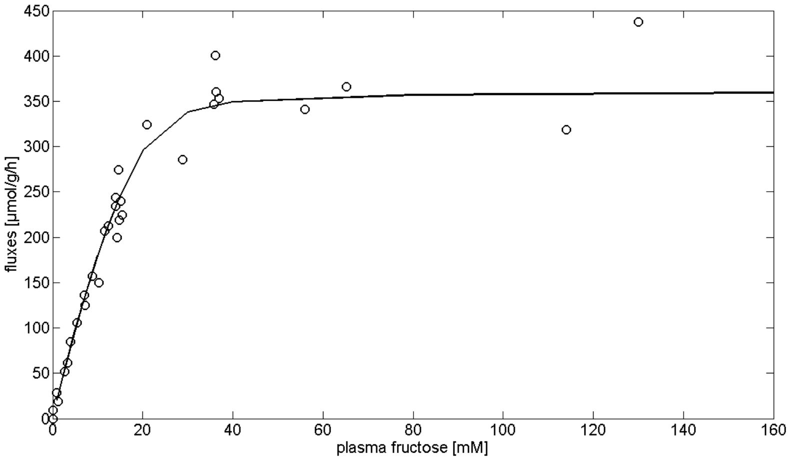

Supplementary Figure 28: Rate of fructose uptake as function of plasma fructose.

20 Galactose uptake

Simulation Stationary rate of galactose uptake as function of external galactose

Physiological relevance Galactose taken up with milk and dairy products is almost exclusively metabolized by the liver. Inherited disorders of hepatic galactose metabolism (in particular 'classic' type 1 galactosemia) may give rise to severe illness.

Experiments: Perfused liver [368]

External Conditions/Initial Values

Glucose [mM]	Galactose [mM]	Fructose [mM]	Lactate [mM]	Pyruvate [mM]	Glycerol [mM]	Acetate [mM]	Ethanol [mM]	fatty acids [mM]
0	0-4	0	1	0.1	0.0676	0	0	0.2

Acetoacetate [mM]	β-hydroxybuterate [mM]	Oxygen [mmHg]	Ammonia [mM]	Glutamat [mM]	Glutamin [mM]	Serine [mM]	Alanine [mM]	Insulin [pM]	Glucagon [pM]
0.11	0.07	66	0.025	0.03	0.9	0.3	0.6	1	1

Comparison of model simulation with experimental data

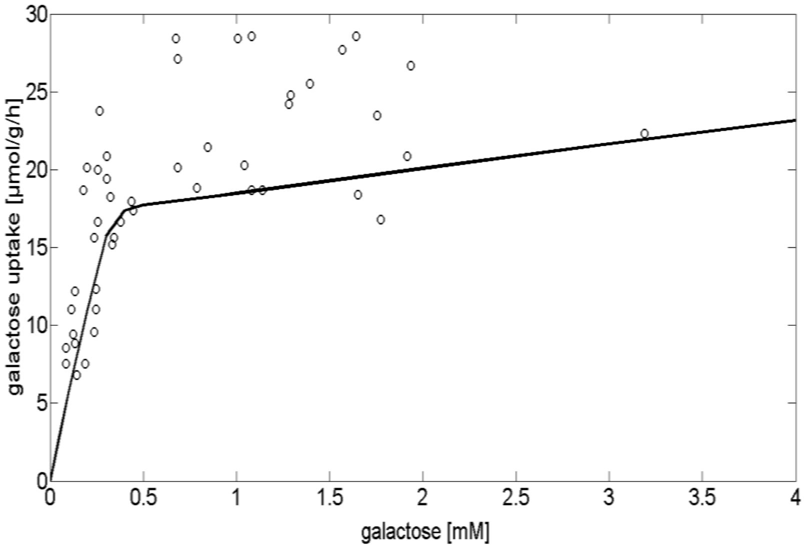

Supplementary Figure 29: Rate of galactose uptake as function of plasma galactose.

21 Ethanol uptake

Simulation Stationary rate of ethanol uptake in dependence of plasma ethanol

Physiological relevance Metabolization of ethanol to acetyl-CoA is one of the most important detoxifying functions of the liver

Experiments Isolated hepatocytes [369-372]

External Conditions/Initial Values

Glucose [mM]	Galactose [mM]	Fructose [mM]	Lactate [mM]	Pyruvate [mM]	Glycerol [mM]	Acetate [mM]	Ethanol [mM]	Free fatty acids [mM]
3	0	4	1	0.2	0.0676	0	0-10	1.2

Acetoacetate [mM]	β-hydroxybuterate [mM]	Oxygen [mmHg]	Ammonia [mM]	Glutamat [mM]	Glutamin [mM]	Serine [mM]	Alanine [mM]	Insulin [pM]	Glucagon [pM]
0.11	0.07	66	0.025	0.03	0.9	0.3	0.6	1	1

Comparison of model simulation with experimental data

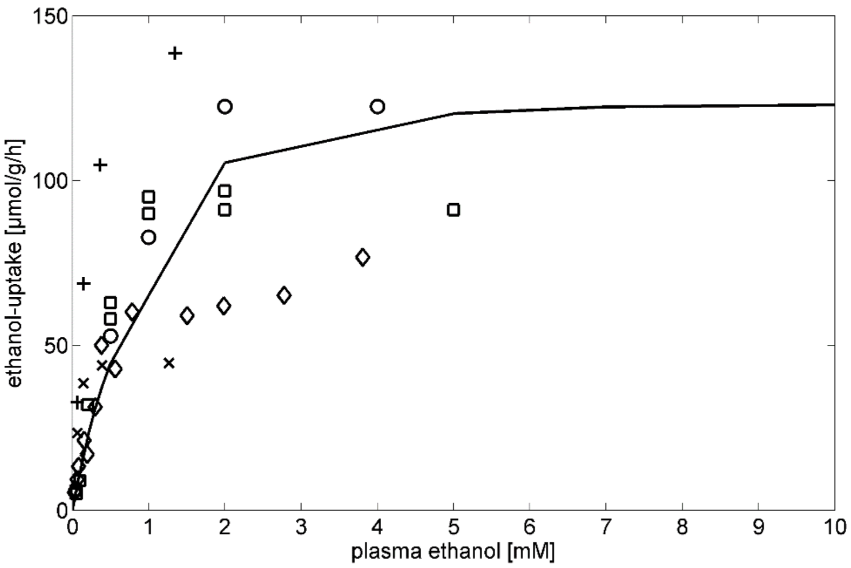

Supplementary Figure 30: Rate of ethanol uptake as function of external ethanol. Data from [369] (o), [370] (□), [371] (x) and (+), and [372] (◊).

Supplementary Note 4 Rate equations and kinetic parameters for galactose metabolism and galactosemia

Kinetic properties of enzymes of the Leloir pathway:

Galactokinase (galk)

$$v_{galk} = V_{max}^{galk} \cdot \left(\frac{gal_{cyt}}{gal_{cyt} + K_m^{gal_{cyt}}} \right) \cdot \left(\frac{atp_{cyt}}{atp_{cyt} + K_m^{atp_{cyt}}} \right) \cdot \left(1 - \frac{gal1p_{cyt}}{gal1p_{cyt} + K_i^{gal1p_{cyt}}} \right)$$

V_{max}^{galk} for numerical value see Supplementary Table 1

$K_m^{gal_{cyt}} = 0.15$ [294]

$K_m^{atp_{cyt}} = 0.095$ [295]

$K_i^{gal1p_{cyt}} = 2$ [294]

Galactose-1-phosphate uridyltransferase (galt)

$$v_{galt} = V_{max}^{galt} \cdot \frac{gal1p_{cyt} \cdot udpglc_{cyt} - {}^{1}\!/_{K_{eq}^{galt}} \cdot udpgal_{cyt} \cdot glc1p_{cyt}}{\left(\left(1 + \frac{gal1p_{cyt}}{K_m^{gal1p_{cyt}}}\right) \cdot \left(1 + \frac{udpglc_{cyt}}{K_m^{udpglc_{cyt}}}\right) + \left(1 + \frac{udpgal_{cyt}}{K_m^{udpgal_{cyt}}}\right) \cdot \left(1 + \frac{glc1p_{cyt}}{K_m^{glc1p_{cyt}}}\right) - 1 \right)}$$

$$V_{max}^{galt} = V_0^{galt} \cdot \left(1 + \frac{gal1p_{cyt}}{K_a^{gal1p_{cyt}}}\right) \cdot \left(1 + \frac{udpglc_{cyt}}{K_a^{udpglc_{cyt}}}\right)$$

V_0^{galt} for numerical value see Supplementary Table 1

$K_a^{gal1p_{cyt}} = 0.5$ [296]

$K_a^{udpglc_{cyt}} = 0.25$ [296]

$K_{eq}^{galt} = 1.67$ [297]

$K_m^{gal1p_{cyt}} = 0.139$ [296]

$$K_m^{udpglc_{cyt}} = K_0^{udpglc_{cyt}} \cdot \left(1 + \frac{udp_{cyt}}{K_i^{udp_{cyt}}}\right) \cdot \left(1 + \frac{utp_{cyt}}{K_i^{utp_{cyt}}}\right)$$

$K_0^{udpglc_{cyt}} = 0.156$ [296]

$K_i^{udp_{cyt}} = 0.35$ [298]

$K_i^{utp_{cyt}} = 0.13$ [298]

$K_m^{udpgal_{cyt}} = 0.167$ [296]

$K_m^{glc1p_{cyt}} = 0.016$ [299]

Uridine diphosphate (UDP)-galactose-4-epimerase (gale)

$$v_{gale} = V_{max}^{gale} \cdot \frac{udpgal_{cyt} - {}^{1}\!/_{K_{eq}^{gale}} \cdot udpglc_{cyt}}{\left(\left(1 + \frac{udpglc_{cyt}}{K_m^{udpglc_{cyt}}} + \frac{udpgal_{cyt}}{K_m^{udpgal_{cyt}}} \right) \right)}$$

$$V_{max}^{gale} = V_0^{gale} \cdot \left(1 - \frac{nadh_{cyt}}{nadh_{cyt} + K_i^{nadh_{cyt}}} \right)$$

V_0^{gale} for numerical value see Supplementary Table 1

$K_i^{nadh_{cyt}} = 0.002$ [301]

$K_{eq}^{gale} = 3.1$ [300]

$K_m^{udpglc_{cyt}} = 0.09$ [301]

$K_m^{udpgal_{cyt}} = 0.05$ [301]

Patient-specific enzymes deficiencies in type I, type II and type III galactosemia

Type I (**galt**)	$V_{max}^{galt} = 0.1 \cdot V_{max}^{galt}$ [373]
Type II (**galk**)	$V_{max}^{galt-typeII} = 0.14/0.91 \cdot V_{max}^{galt}$ [295] $K_m^{atp_{cyt}-typi\ II} = 0.755$ [295]
Type III **gale**	$V_{max}^{gale} = \frac{15}{36} \cdot V_{max}^{gale}$ [374]

Patient-specific parameter changes of the deficient enzymes studied in the simulations shown in Fig. 8, main text.

Galactose infusion kinetics

$$v_{gal-infus} = v_{max}^{gal-infus} \cdot \frac{t}{t + K_t} \cdot \exp\left(-\frac{t}{t_d} \right)$$

$v_{max}^{gal-infus} = 36\ \mu mol/g/h$

$K_t = 0.2\ h$

$t_d = 2\ h$

Human sample collection

Primary hepatocellular adenoma or HCC tissues and adjacent noncancerous tissues were collected from 3 patients undergoing curative resection (R0) at the Chirurgische Klinik, Campus Charité Mitte | Campus Virchow-Klinikum. Ethical approval for tissue sampling and analysis was obtained from the ethics committee at Charité – Universitätsmedizin Berlin (EA1/140/15). The three patients gave informed consent to the scientific use of their resected tissue specimen for molecular analyses. Macroscopically, the analysed HCC tissues were graded as T1 (HCC1) or T3a (HCC2) according to the TNM classification [375]. No distant metastases or spreading to lymph nodes were found. The histopathological grading (according to Hamilton and Aaltonen 2000) revealed in all HCC cases moderately differentiated G2 stage tumours. The analysed adenomatous tissue originated from a highly differentiated hepatocellular neoplasia with minimal nuclear atypia and without features of malignancy.

Proteomics

About 10 mg of every frozen tissue was homogenized under denaturing conditions in a buffer containing 3 M guanidinium chloride, 5 mM tris(2-carboxyethyl)phosphine, 20 mM chloroacetamide and 50 mM Tris pH 8.5 by FastPrep (settings: 2 x 60 s; 4 m/s) with a steel ball. Lysates were sonicated and boiled at 95°C for 15 min. Lysates were diluted 1:6 in 10% acetonitrile, 25 mM Tris, pH 8.5. 100 µg protein was digested sequentially with 2 µg LysC (Roche) at 25°C for 2h and 2 µg trypsin at 37°C overnight. Solid phase extraction (SPE) disc cartridges (C18-SD, Waters, Milford, MA) were used for desalting according to the manufacturer's instructions. Desalted peptides were further separated using five fractions of strong cation exchange chromatography (SCX, 3M Purification, Meriden, CT). Eluates were lyophilized and dissolved in 5% acetonitrile, 2% formic acid, vortexed and sonicate 5 minutes prior injection to LC-MS.

LC-MS Settings for Proteomics

LC−MS/MS was carried out by nanoflow reverse-phase liquid chromatography (Dionex Ultimate 3000, Thermo Scientific, USA) coupled online to a Q-Exactive HF Orbitrap mass spectrometer (Thermo Scientific). LC separation was performed using a PicoFrit analytical column (75 µm ID × 55 cm long, 15 µm Tip ID (New Objectives, Woburn, MA, USA)) in-house packed with 3-µm C18 resin (Reprosil-AQ Pur, Dr. Maisch, Germany). Peptides were eluted using a nonlinear gradient from 3.8 to 98% solvent B over 120 min at a flow rate of 266 nL/min (solvent A: 0.1% formic acid in water; solvent B: 80% acetonitrile and 0.1% formic acid) under a controlled temperature of 50°C. 3.5 kilovolts were applied for nanoelectrospray generation. A cycle of one full FT scan mass spectrum (300−1750 m/z, resolution of 60 000 at m/z 200, AGC target $1e^6$) was followed by 12 data-dependent MS/MS

scans (200-2000 m/z, resolution of 30 000, AGC target $5e^5$, isolation window 2 m/z) with normalized collision energy of 25 eV. Target ions already selected for MS/MS were dynamically excluded for 15 s. In addition, only peptide charge states between two to eight were allowed.

Raw MS data were processed with MaxQuant software (v1.5.3.30) [376] with the Andromeda search engine [377] and searched against the human proteome database UniProtKB with 70,228 entries released in 02/2016. A false discovery rate (FDR) of 0.01 for proteins and peptides, a minimum peptide length of 7 amino acids, a mass tolerance of 4.5 ppm for precursor and 20 ppm for fragment ions were required. A maximum of two missed cleavages was allowed for the tryptic digest. Cysteine carbamidomethylation was set as fixed modification, while N-terminal acetylation and methionine oxidation, were set as variable modifications. The label-free software MaxLFQ [378], which is integrated into MaxQuant, was used for relative quantification.

Fold changes of enzyme protein abundances of the tumor tissue with respect to normal liver tissue are given in Supplementary Data 3. If no protein abundance of an enzyme could be determined from the proteomics analysis its activity was not altered in the simulations (i.e. fold change = 1).

1. Stremmel, W., Strohmeyer, G. & Berk, P.D. Hepatocellular Uptake of Oleate Is Energy-Dependent, Sodium Linked, and Inhibited by an Antibody to a Hepatocyte Plasma-Membrane Fatty-Acid Binding-Protein. *Proceedings of the National Academy of Sciences of the United States of America* **83**, 3584-3588 (1986).
2. Kampf, J.P. & Kleinfeld, A.M. Fatty acid transport in adipocytes monitored by imaging intracellular free fatty acid levels. *Journal of Biological Chemistry* **279**, 35775-35780 (2004).
3. Kim, J.H., Lewin, T.M. & Coleman, R.A. Expression and characterization of recombinant rat Acyl-CoA synthetases 1, 4, and 5. Selective inhibition by triacsin C and thiazolidinediones. *The Journal of biological chemistry* **276**, 24667-24673 (2001).
4. Saggerson, E.D., Carpenter, C.A. & Tselentis, B.S. Effects of thyroidectomy and starvation on the activity and properties of hepatic carnitine palmitoyltransferase. *The Biochemical journal* **208**, 667-672 (1982).
5. Saggerson, E.D. & Carpenter, C.A. Effects of fasting and malonyl CoA on the kinetics of carnitine palmitoyltransferase and carnitine octanoyltransferase in intact rat liver mitochondria. *FEBS Lett* **132**, 166-168 (1981).
6. McGarry, J.D. & Brown, N.F. The mitochondrial carnitine palmitoyltransferase system. From concept to molecular analysis. *European journal of biochemistry* **244**, 1-14 (1997).
7. McGarry, J.D., Mills, S.E., Long, C.S. & Foster, D.W. Observations on the affinity for carnitine, and malonyl-CoA sensitivity, of carnitine palmitoyltransferase I in animal and human tissues. Demonstration of the presence of malonyl-CoA in non-hepatic tissues of the rat. *The Biochemical journal* **214**, 21-28 (1983).
8. Aires, C.C. et al. Inhibition of hepatic carnitine palmitoyl-transferase I (CPT IA) by valproyl-CoA as a possible mechanism of valproate-induced steatosis. *Biochem Pharmacol* **79**, 792-799 (2010).
9. Pieklik, J.R. & Guynn, R.W. Equilibrium constants of the reactions of choline acetyltransferase, carnitine acetyltransferase, and acetylcholinesterase under physiological conditions. *The Journal of biological chemistry* **250**, 4445-4450 (1975).
10. Indiveri, C., Tonazzi, A. & Palmieri, F. The reconstituted carnitine carrier from rat liver mitochondria: evidence for a transport mechanism different from that of the other mitochondrial translocators. *Biochim Biophys Acta* **1189**, 65-73 (1994).
11. Murthy, M.S. & Pande, S.V. Mechanism of carnitine acylcarnitine translocase-catalyzed import of acylcarnitines into mitochondria. *The Journal of biological chemistry* **259**, 9082-9089 (1984).
12. Indiveri, C., Tonazzi, A., Prezioso, G. & Palmieri, F. Kinetic characterization of the reconstituted carnitine carrier from rat liver mitochondria. *Biochim Biophys Acta* **1065**, 231-238 (1991).
13. Norum, K.R. Palmityl-Coa - Carnitine Palmityltransferase - Purification from Calf-Liver Mitochondria + Some Properties of Enzyme. *Biochimica Et Biophysica Acta* **89**, 95-& (1964).
14. Kopec, B. & Fritz, I.B. Properties of a Purified Carnitine Palmitoyltransferase, and Evidence for Existence of Other Carnitine Acyltransferases. *Canadian Journal of Biochemistry* **49**, 941-& (1971).
15. Brown, N.F., Anderson, R.C., Caplan, S.L., Foster, D.W. & McGarry, J.D. Catalytically important domains of rat carnitine palmitoyltransferase II as determined by site-directed mutagenesis and chemical modification. Evidence for a critical histidine residue. *The Journal of biological chemistry* **269**, 19157-19162 (1994).
16. Ikeda, Y., Okamura-Ikeda, K. & Tanaka, K. Purification and characterization of short-chain, medium-chain, and long-chain acyl-CoA dehydrogenases from rat liver mitochondria. Isolation of the holo- and apoenzymes and conversion of the apoenzyme to the holoenzyme. *The Journal of biological chemistry* **260**, 1311-1325 (1985).
17. Osumi, T., Hashimoto, T. & Ui, N. Purification and properties of acyl-CoA oxidase from rat liver. *J Biochem* **87**, 1735-1746 (1980).
18. Stern, J.R. & Del Campillo, A. Enzymes of fatty acid metabolism. II. Properties of crystalline crotonase. *The Journal of biological chemistry* **218**, 985-1002 (1956).
19. Furuta, S., Miyazawa, S., Osumi, T., Hashimoto, T. & Ui, N. Properties of mitochondria and peroxisomal enoyl-CoA hydratases from rat liver. *J Biochem* **88**, 1059-1070 (1980).

20. Lynen, F. & Wieland, O. Beta-Ketoreductase. *Methods in Enzymology* **1**, 566-573 (1955).
21. Osumi, T. & Hashimoto, T. Purification and properties of mitochondrial and peroxisomal 3-hydroxyacyl-CoA dehydrogenase from rat liver. *Arch Biochem Biophys* **203**, 372-383 (1980).
22. Wakil, S.J., Green, D.E., Mii, S. & Mahler, H.R. Studies on the Fatty Acid Oxidizing System of Animal Tissues .6. Beta-Hydroxyacyl Coenzyme a Dehydrogenase. *Journal of Biological Chemistry* **207**, 631-638 (1954).
23. Kobayashi, A., Jiang, L.L. & Hashimoto, T. Two mitochondrial 3-hydroxyacyl-CoA dehydrogenases in bovine liver. *J Biochem* **119**, 775-782 (1996).
24. Goldman, D.S. Studies on the Fatty Acid Oxidizing System of Animal Tissues .7. The Beta-Ketoacyl Coenzyme a Cleavage Enzyme. *Journal of Biological Chemistry* **208**, 345-357 (1954).
25. Miyazawa, S., Furuta, S., Osumi, T., Hashimoto, T. & Ui, N. Properties of peroxisomal 3-ketoacyl-coA thiolase from rat liver. *J Biochem* **90**, 511-519 (1981).
26. Yamashita, H., Itsuki, A., Kimoto, M., Hiemori, M. & Tsuji, H. Acetate generation in rat liver mitochondria; acetyl-CoA hydrolase activity is demonstrated by 3-ketoacyl-CoA thiolase. *Biochim Biophys Acta* **1761**, 17-23 (2006).
27. Kalousek, F., Darigo, M.D. & Rosenberg, L.E. Isolation and characterization of propionyl-CoA carboxylase from normal human liver. Evidence for a protomeric tetramer of nonidentical subunits. *The Journal of biological chemistry* **255**, 60-65 (1980).
28. Allen, S.H., Kellermeyer, R., Stjernholm, R., Jacobson, B. & Wood, H.G. The isolation, purification, and properties of methylmalonyl racemase. *The Journal of biological chemistry* **238**, 1637-1642 (1963).
29. Kellermeyer, R.W., Allen, S.H., Stjernholm, R. & Wood, H.G. Methylmalonyl Isomerase.Iv. Purification and Properties of the Enzyme from Propionibacteria. *The Journal of biological chemistry* **239**, 2562-2569 (1964).
30. Usselman, R.J. et al. Impact of mutations on the midpoint potential of the [4Fe-4S](+1,+2) cluster and on catalytic activity in electron transfer flavoprotein-ubiquinone oxidoreductase (ETF-QO). *Biochemistry* **47**, 92-100 (2008).
31. Husain, M., Stankovich, M.T. & Fox, B.G. Measurement of the oxidation-reduction potentials for one-electron and two-electron reduction of electron-transfer flavoprotein from pig liver. *The Biochemical journal* **219**, 1043-1047 (1984).
32. Brown, G.C. & Brand, M.D. Proton-Electron Stoichiometry of Mitochondrial Complex-I Estimated from the Equilibrium Thermodynamic Force Ratio. *Biochemical Journal* **252**, 473-479 (1988).
33. Siess, E.A., Nimmannit, S. & Wieland, O.H. Kinetic and regulatory properties of pyruvate dehydrogenase from Ehrlich ascites tumor cells. *Cancer Res* **36**, 55-59 (1976).
34. Roche, T.E. & Cate, R.L. Purification of porcine liver pyruvate dehydrogenase complex and characterization of its catalytic and regulatory properties. *Arch Biochem Biophys* **183**, 664-677 (1977).
35. Batenburg, J.J. & Olson, M.S. Regulation of pyruvate dehydrogenase by fatty acid in isolated rat liver mitochondria. *The Journal of biological chemistry* **251**, 1364-1370 (1976).
36. Wieland, O. & Weiss, L. Inhibition of Citrate-Synthase by Palmityl-Coenzyme A. *Biochem Biophys Res Commun* **13**, 26-31 (1963).
37. Shepherd, D. & Garland, P.B. The kinetic properties of citrate synthase from rat liver mitochondria. *The Biochemical journal* **114**, 597-610 (1969).
38. Smith, C.M. & Williams.Jr Inhibition of Citrate Synthase by Succinyl-Coa and Other Metabolites. *Febs Letters* **18**, 35-& (1971).
39. Blair, J.M. Magnesium and the aconitase equilibrium: determination of apparent stability constants of mangnesium substrate complexes from equilibrium data. *European journal of biochemistry* **8**, 287-291 (1969).
40. Guarriero-Bobyleva, V., Volpi-Becchi, M.A. & Masini, A. Parallel partial purification of cytoplasmic and mitochondrial aconitate hydratases from rat liver. *European journal of biochemistry* **34**, 455-458 (1973).
41. Plaut, G.W. & Aogaichi, T. Purification and properties of diphosphopyridine nuleotide-linked isocitrate dehydrogenase of mammalian liver. *The Journal of biological chemistry* **243**, 5572-5583 (1968).
42. Smith, C.M., Bryla, J. & Williamson, J.R. Regulation of mitochondrial alpha-ketoglutarate metabolism by product inhibition at alpha-ketoglutarate dehydrogenase. *The Journal of biological chemistry* **249**, 1497-1505 (1974).
43. Strumilo, S., Czygier, M., Kondracikowska, J., Dobrzyn, P. & Czerniecki, J. Kinetic and spectral investigation of allosteric interaction of coenzymes with 2-oxo acid dehydrogenase complexes. *Journal of Molecular Structure* **614**, 221-226 (2002).

44. Lambeth, D.O., Tews, K.N., Adkins, S., Frohlich, D. & Milavetz, B.I. Expression of two succinyl-CoA synthetases with different nucleotide specificities in mammalian tissues. *Journal of Biological Chemistry* **279**, 36621-36624 (2004).
45. Phillips, D., Aponte, A.M., French, S.A., Chess, D.J. & Balaban, R.S. Succinyl-CoA Synthetase Is a Phosphate Target for the Activation of Mitochondrial Metabolism. *Biochemistry* **48**, 7140-7149 (2009).
46. Kaufman, S. & Alivisatos, S.G.A. Purification and Properties of the Phosphorylating Enzyme from Spinach. *Journal of Biological Chemistry* **216**, 141-152 (1955).
47. Johnson, J.D., Muhonen, W.W. & Lambeth, D.O. Characterization of the ATP- and GTP-specific Succinyl-CoA synthetases in pigeon - The enzymes incorporate the same alpha-subunit. *Journal of Biological Chemistry* **273**, 27573-27579 (1998).
48. Lynn, R. & Guynn, R.W. Equilibrium-Constants under Physiological Conditions for Reactions of Succinyl Coenzyme-a Synthetase and Hydrolysis of Succinyl Coenzyme-a to Coenzyme-a and Succinate. *Journal of Biological Chemistry* **253**, 2546-2553 (1978).
49. Dutra, J.C. et al. Inhibition of Succinate-Dehydrogenase and Beta-Hydroxybutyrate Dehydrogenase-Activities by Methylmalonate in Brain and Liver of Developing Rats. *Journal of Inherited Metabolic Disease* **16**, 147-153 (1993).
50. Vinogradov, A.D., Kotlyar, A.B., Burov, V.I. & Belikova, Y.O. Regulation of Succinated Dehydrogenase and Tautomerization of Oxaloacetate. *Advances in Enzyme Regulation* **28**, 271-280 (1989).
51. Tushurashvili, P.R., Gavrikova, E.V., Ledenev, A.N. & Vinogradov, A.D. Studies on the Succinate Dehydrogenating System - Isolation and Properties of the Mitochondrial Succinate-Ubiquinone Reductase. *Biochimica Et Biophysica Acta* **809**, 145-159 (1985).
52. Bock, R.M. & Alberty, R.A. Studies of the Enzyme Fumarase .1. Kinetics and Equilibrium. *Journal of the American Chemical Society* **75**, 1921-1925 (1953).
53. Kobayashi, K., Yamanishi, T. & Tuboi, S. Physicochemical, Catalytic, and Immunochemical Properties of Fumarases Crystallized Separately from Mitochondrial and Cytosolic Fractions of Rat-Liver. *Journal of Biochemistry* **89**, 1923-1931 (1981).
54. Raval, D.N. & Wolfe, R.G. Malic Dehydrogenase .4. Ph Dependence of Kinetic Parameters. *Biochemistry* **1**, 1118-& (1962).
55. Thorne, C.J.R. Properties of Mitochondrial Malate Dehydrogenases. *Biochimica Et Biophysica Acta* **59**, 624-& (1962).
56. Gelpi, J.L., Dordal, A., Montserrat, J., Mazo, A. & Cortes, A. Kinetic-Studies of the Regulation of Mitochondrial Malate-Dehydrogenase by Citrate. *Biochemical Journal* **283**, 289-297 (1992).
57. Moyle, J. & Mitchell, P. Proton Translocating Nicotinamide-Adenine Dinucleotide (Phosphate) Transhydrogenase of Rat-Liver Mitochondria. *Biochemical Journal* **132**, 571-585 (1973).
58. Kaplan, N.O., Colowick, S.P. & Neufeld, E.F. Pyridine Nucleotide Transhydrogenase .3. Animal Tissue Transhydrogenases. *Journal of Biological Chemistry* **205**, 1-15 (1953).
59. Rosen, B.P. & Futai, M. Sodium-Proton Antiporter of Rat-Liver Mitochondria. *Febs Letters* **117**, 39-43 (1980).
60. Seren, S. et al. Current-voltage relationships for proton flow through the F0 sector of the ATP-synthase, carbonylcyanide-p-trifluoromethoxyphenylhydrazone or leak pathways in submitochondrial particles. *European journal of biochemistry* **152**, 373-379 (1985).
61. Gao, Y.Q., Yang, W. & Karplus, M. A structure-based model for the synthesis and hydrolysis of ATP by F-1-ATPase. *Cell* **123**, 195-205 (2005).
62. Bohnensack, R., Kuster, U. & Letko, G. Rate-Controlling Steps of Oxidative-Phosphorylation in Rat-Liver Mitochondria - a Synoptic Approach of Model and Experiment. *Biochimica Et Biophysica Acta* **680**, 271-280 (1982).
63. Coty, W.A. & Pedersen, P.L. Phosphate Transport in Rat-Liver Mitochondria - Kinetics and Energy-Requirements. *Journal of Biological Chemistry* **249**, 2593-2598 (1974).
64. Burton, K. & Wilson, T.H. The Free-Energy Changes for the Reduction of Diphosphopyridine Nucleotide and the Dehydrogenation of L-Malate and L-Glycerol 1-Phosphate. *Biochemical Journal* **54**, 86-94 (1953).
65. Nakashima, Y. et al. Steady-state kinetics of NADH:coenzyme Q oxidoreductase isolated from bovine heart mitochondria. *J Bioenerg Biomembr* **34**, 11-19 (2002).
66. Hinkle, P. & Mitchell, P. Effect of membrane potential on equilibrium poise between cytochrome a and cytochrome c in rat liver mitochondria. *J Bioenerg* **1**, 45-60 (1970).
67. Fato, R. et al. Steady-state kinetics of ubiquinol-cytochrome c reductase in bovine heart submitochondrial particles: diffusional effects. *The Biochemical journal* **290 (Pt 1)**, 225-236 (1993).

68. Napiwotzki, J. & Kadenbach, B. Extramitochondrial ATP/ADP-ratios regulate cytochrome c oxidase activity via binding to the cytosolic domain of subunit IV. *Biological Chemistry* **379**, 335-339 (1998).
69. Derr, R.F. & Zieve, L. Adenylate Energy Charge - Relation to Guanylate Energy Charge and Adenylate Kinase Equilibrium Constant. *Biochemical and Biophysical Research Communications* **49**, 1385-& (1972).
70. Criss, W.E., Sapico, V. & Litwack, G. Rat Liver Adenosine Triphosphate - Adenosine Monophosphate Phosphotransferase Activity .1. Purification and Physical and Kinetic Characterization of Adenylate Kinase-Iii. *Journal of Biological Chemistry* **245**, 6346-& (1970).
71. Yoshida, C., Shah, H. & Weinhouse, S. Purification and properties of inorganic pyrophosphatase of rat liver and hepatoma 3924A. *Cancer Res* **42**, 3526-3531 (1982).
72. Ciaraldi, T.P., Horuk, R. & Matthaei, S. Biochemical and Functional-Characterization of the Rat-Liver Glucose-Transport System - Comparisons with the Adipocyte Glucose-Transport System. *Biochemical Journal* **240**, 115-123 (1986).
73. Bontemps, F., Hue, L. & Hers, H.G. Phosphorylation of glucose in isolated rat hepatocytes. Sigmoidal kinetics explained by the activity of glucokinase alone. *The Biochemical journal* **174**, 603-611 (1978).
74. Dawson, C.M. & Hales, C.N. Inhibition of Rat Liver Glucokinase by Palmitoyl-Coa. *Biochimica Et Biophysica Acta* **176**, 657-& (1969).
75. Storer, A.C. & Cornishbowden, A. Kinetics of Rat-Liver Glucokinase - Cooperative Interactions with Glucose at Physiologically Significant Concentrations. *Biochemical Journal* **159**, 7-14 (1976).
76. Van Schaftingen, E. A protein from rat liver confers to glucokinase the property of being antagonistically regulated by fructose 6-phosphate and fructose 1-phosphate. *European journal of biochemistry* **179**, 179-184 (1989).
77. Agius, L. & Peak, M. Intracellular binding of glucokinase in hepatocytes and translocation by glucose, fructose and insulin. *The Biochemical journal* **296 (Pt 3)**, 785-796 (1993).
78. Grossbard, L. & Schimke, R.T. Multiple hexokinases of rat tissues. Purification and comparison of soluble forms. *The Journal of biological chemistry* **241**, 3546-3560 (1966).
79. Igarashi, Y., Kato, S. & Tada, K. Kinetic properties of the glucose-6-phosphate transport system in rat hepatic microsomal membranes. *J Inherit Metab Dis* **8**, 153-154 (1985).
80. Arion, W.J. & Nordlie, R.C. Liver Microsomal Glucose 6-Phosphatase, Inorganic Pyrophosphatase, and Pyrophosphate-Glucose Phosphotransferase. Ii. Kinetic Studies. *The Journal of biological chemistry* **239**, 2752-2757 (1964).
81. St-Denis, J.F., Berteloot, A., Vidal, H., Annabi, B. & van de Werve, G. Glucose transport and glucose 6-phosphate hydrolysis in intact rat liver microsomes. *The Journal of biological chemistry* **270**, 21092-21097 (1995).
82. Tewari, Y.B., Steckler, D.K. & Goldberg, R.N. Thermodynamics of isomerization reactions involving sugar phosphates. *The Journal of biological chemistry* **263**, 3664-3669 (1988).
83. Zalitis, J. & Oliver, I.T. Inhibition of Glucose Phosphate Isomerase by Metabolic Intermediates of Fructose. *Biochemical Journal* **102**, 753-& (1967).
84. Van Schaftingen, E., Davies, D.R. & Hers, H.G. Inactivation of phosphofructokinase 2 by cyclic AMP - dependent protein kinase. *Biochem Biophys Res Commun* **103**, 362-368 (1981).
85. Sakakibara, R., Kitajima, S. & Uyeda, K. Differences in kinetic properties of phospho and dephospho forms of fructose-6-phosphate, 2-kinase and fructose 2,6-bisphosphatase. *The Journal of biological chemistry* **259**, 41-46 (1984).
86. Vanschaftingen, E., Davies, D.R. & Hers, H.G. Fructose-2,6-Bisphosphatase from Rat-Liver. *European journal of biochemistry* **124**, 143-149 (1982).
87. Uyeda, K., Furuya, E. & Luby, L.J. The Effect of Natural and Synthetic D-Fructose 2,6-Bisphosphate on the Regulatory Kinetic-Properties of Liver and Muscle Phosphofructokinases. *Journal of Biological Chemistry* **256**, 8394-8399 (1981).
88. Van Schaftingen, E., Jett, M.F., Hue, L. & Hers, H.G. Control of liver 6-phosphofructokinase by fructose 2,6-bisphosphate and other effectors. *Proc Natl Acad Sci U S A* **78**, 3483-3486 (1981).
89. Reinhart, G.D. & Lardy, H.A. Rat liver phosphofructokinase: kinetic activity under near-physiological conditions. *Biochemistry* **19**, 1477-1484 (1980).
90. Meek, D.W. & Nimmo, H.G. Effects of phosphorylation on the kinetic properties of rat liver fructose-1,6-bisphosphatase. *The Biochemical journal* **222**, 125-130 (1984).
91. Veech, R.L., Raijman, L., Dalziel, K. & Krebs, H.A. Disequilibrium in the triose phosphate isomerase system in rat liver. *The Biochemical journal* **115**, 837-842 (1969).
92. Ikehara, Y., Endo, H. & Okada, Y. The identity of the aldolases isolated from rat muscle and primary hepatoma. *Arch Biochem Biophys* **136**, 491-497 (1970).

93. Malay, A.D., Procious, S.L. & Tolan, D.R. The temperature dependence of activity and structure for the most prevalent mutant aldolase B associated with hereditary fructose intolerance. *Archives of Biochemistry and Biophysics* **408**, 295-304 (2002).
94. Lee, E.W., Barriso, J.A., Pepe, M. & Snyder, R. Purification and Properties of Liver Triose Phosphate Isomerase. *Biochimica Et Biophysica Acta* **242**, 261-& (1971).
95. Cori, C.F., Velick, S.F. & Cori, G.T. The combination of diphosphopyridine nucleotide with glyceraldehyde phosphate dehydrogenase. *Biochim Biophys Acta* **4**, 160-169 (1950).
96. Ryzlak, M.T. & Pietruszko, R. Heterogeneity of Glyceraldehyde-3-Phosphate Dehydrogenase from Human-Brain. *Biochimica Et Biophysica Acta* **954**, 309-324 (1988).
97. Smith, C.M. & Velick, S.F. The glyceraldehyde 3-phosphate dehydrogenases of liver and muscle. Cooperative interactions and conditions for functional reversibility. *The Journal of biological chemistry* **247**, 273-284 (1972).
98. Cornell, N.W., Leadbetter, M. & Veech, R.L. Effects of free magnesium concentration and ionic strength on equilibrium constants for the glyceraldehyde phosphate dehydrogenase and phosphoglycerate kinase reactions. *The Journal of biological chemistry* **254**, 6522-6527 (1979).
99. Krietsch, W.K. & Bucher, T. 3-phosphoglycerate kinase from rabbit sceletal muscle and yeast. *European journal of biochemistry* **17**, 568-580 (1970).
100. Fritz, P.J. & White, E.L. 3-Phosphoglycerate kinase from rat tissues. Further characterization and developmental studies. *Biochemistry* **13**, 444-449 (1974).
101. Rodwell, V.W., Towne, J.C. & Grisolia, S. The kinetic properties of yeast and muscle phosphoglyceric acid mutase. *The Journal of biological chemistry* **228**, 875-890 (1957).
102. Fundele, R. & Krietsch, W.K. Purification and properties of the phosphoglycerate mutase isozymes from the mouse. *Comp Biochem Physiol B* **81**, 965-968 (1985).
103. Schuster, R. & Holzhutter, H.G. Use of mathematical models for predicting the metabolic effect of large-scale enzyme activity alterations. Application to enzyme deficiencies of red blood cells. *European journal of biochemistry* **229**, 403-418 (1995).
104. Rider, C.C. & Taylor, C.B. Enolase isoenzymes in rat tissues. Electrophoretic, chromatographic, immunological and kinetic properties. *Biochim Biophys Acta* **365**, 285-300 (1974).
105. Blair, J.B., Cimbala, M.A., Foster, J.L. & Morgan, R.A. Hepatic Pyruvate-Kinase - Regulation by Glucagon, Cyclic Adenosine 3' - 5'-Monophosphate, and Insulin in Perfused Rat-Liver. *Journal of Biological Chemistry* **251**, 3756-3762 (1976).
106. Middleton, M.C. & Walker, D.G. Comparison of the properties of two forms of pyruvate kinase in rat liver and determination of their separate activities during development. *The Biochemical journal* **127**, 721-731 (1972).
107. Feliu, J.E., Hue, L. & Hers, H.G. Hormonal control of pyruvate kinase activity and of gluconeogenesis in isolated hepatocytes. *Proc Natl Acad Sci U S A* **73**, 2762-2766 (1976).
108. van Berkel, T.J., Kruijt, J.K. & Koster, J.F. Hormone-induced changes in pyruvate kinase. Effects of glucagon and starvation. *European journal of biochemistry* **81**, 423-432 (1977).
109. Walker, P.R., Potter, V.R., Becker, J.E. & Bonney, R.J. Pyruvate-Kinase, Hexokinase, and Aldolase Isoenzymes in Rat-Liver Cells in Culture. *In Vitro-Journal of the Tissue Culture Association* **8**, 107-& (1972).
110. Wood, H.G., Davis, J.J. & Lochmuller, H. The equilibria of reactions catalyzed by carboxytransphosphorylase, carboxykinase, and pyruvate carboxylase and the synthesis of phosphoendolpyruvate. *The Journal of biological chemistry* **241**, 5692-5704 (1966).
111. Titheradge, M.A., Picking, R.A. & Haynes, R.C. Physiological Concentrations of 2-Oxoglutarate Regulate the Activity of Phosphoenolpyruvate Carboxykinase in Liver. *Biochemical Journal* **285**, 767-771 (1992).
112. Colombo, G., Carlson, G.M. & Lardy, H.A. Phosphoenolpyruvate Carboxykinase (Guanosine Triphosphate) from Rat-Liver Cytosol - Separation of Homogeneous Forms of Enzyme with High and Low Activity by Chromatography on Agarose-Hexane-Guanosine Triphosphate. *Biochemistry* **17**, 5321-5329 (1978).
113. Ballard, F.J. & Hanson, R.W. Phosphoenolpyruvate carboxykinase and pyruvate carboxylase in developing rat liver. *The Biochemical journal* **104**, 866-871 (1967).
114. Jo, J.S., Ishihara, N. & Kikuchi, G. Occurrence and Properties of 4 Forms of Phosphoenolpyruvate Carboxykinase in Chicken Liver. *Archives of Biochemistry and Biophysics* **160**, 246-254 (1974).
115. Johnson, T.A. & Holyoak, T. Increasing the Conformational Entropy of the Omega-Loop Lid Domain in Phosphoenolpyruvate Carboxykinase Impairs Catalysis and Decreases Catalytic Fidelity. *Biochemistry* **49**, 5176-5187 (2010).

116. Ballard, F.J. Kinetic studies with cytosol and mitochondrial phosphoenolpyruvate carboxykinases. *The Biochemical journal* **120**, 809-814 (1970).
117. Holyoak, T. & Nowak, T. pH dependence of the reaction catalyzed by avian mitochondrial phosphoenolpyruvate carboxykinase. *Biochemistry* **43**, 7054-7065 (2004).
118. Wimhurst, J.M. & Manchester, K.L. Some aspects of the kinetics of rat liver pyruvate carboxylase. *The Biochemical journal* **120**, 79-93 (1970).
119. Williamson, D.H., Lund, P. & Krebs, H.A. The redox state of free nicotinamide-adenine dinucleotide in the cytoplasm and mitochondria of rat liver. *The Biochemical journal* **103**, 514-527 (1967).
120. Prabhakaram, M. & Singh, S.N. Effect of age on the crystalline rat liver lactate dehydrogenase. *Arch Gerontol Geriatr* **5**, 57-64 (1986).
121. Anderson, S.R., Florini, J.R. & Vestling, C.S. Rat Liver Lactate Dehydrogenase. 3. Kinetics and Specificity. *The Journal of biological chemistry* **239**, 2991-2997 (1964).
122. Edlund, G.L. & Halestrap, A.P. The kinetics of transport of lactate and pyruvate into rat hepatocytes. Evidence for the presence of a specific carrier similar to that in erythrocytes. *The Biochemical journal* **249**, 117-126 (1988).
123. Halestrap, A.P. The mitochondrial pyruvate carrier. Kinetics and specificity for substrates and inhibitors. *The Biochemical journal* **148**, 85-96 (1975).
124. Indiveri, C., Capobianco, L., Kramer, R. & Palmieri, F. Kinetics of the Reconstituted Dicarboxylate Carrier from Rat-Liver Mitochondria. *Biochimica Et Biophysica Acta* **977**, 187-193 (1989).
125. Titheradge, M.A. & Coore, H.G. Mitochondrial Pyruvate Carrier, Its Exchange Properties and Its Regulation by Glucagon. *Febs Letters* **63**, 45-50 (1976).
126. Palmieri, F., Quagliar.E, Stipani, I. & Klingenb.M Kinetic Study of Tricarboxylate Carrier in Rat-Liver Mitochondria. *European journal of biochemistry* **26**, 587-& (1972).
127. Crow, K.E., Braggins, T.J., Batt, R.D. & Hardman, M.J. Rat liver cytosolic malate dehydrogenase: purification, kinetic properties, role in control of free cytosolic NADH concentration. Analysis of control of ethanol metabolism using computer simulation. *The Journal of biological chemistry* **257**, 14217-14225 (1982).
128. Veech, R.L., Eggleston, L.V. & Krebs, H.A. The redox state of free nicotinamide-adenine dinucleotide phosphate in the cytoplasm of rat liver. *The Biochemical journal* **115**, 609-619 (1969).
129. Zelewski, M. & Swierczynski, J. Malic enzyme in human liver. Intracellular distribution, purification and properties of cytosolic isozyme. *European journal of biochemistry* **201**, 339-345 (1991).
130. Kimura, N. & Shimada, N. Membrane-Associated Nucleoside Diphosphate Kinase from Rat-Liver - Purification, Characterization, and Comparison with Cytosolic Enzyme. *Journal of Biological Chemistry* **263**, 4647-4653 (1988).
131. Fukuchi, T. et al. Recombinant rat nucleoside diphosphate kinase isoforms (alpha and beta): purification, properties and application to immunological detection of native isoforms in rat tissues. *Biochim Biophys Acta* **1205**, 113-122 (1994).
132. Colowick, S.P. & Sutherland, E.W. Polysaccharide synthesis from glucose by means purified enzymes. *Journal of Biological Chemistry* **144**, 423-437 (1942).
133. Kashiwaya, Y. et al. Control of Glucose-Utilization in Working Perfused Rat-Heart. *Journal of Biological Chemistry* **269**, 25502-25514 (1994).
134. Turnquis.Rl, Gillett, T.A. & Hansen, R.G. Uridine-Diphosphate Glucose Pyrophosphorylase - Crystallization and Properties of Enzyme from Rabbit Liver and Species Comparisons. *Journal of Biological Chemistry* **249**, 7695-7700 (1974).
135. Westphal, S.A. & Nuttall, F.Q. Comparative characterization of human and rat liver glycogen synthase. *Arch Biochem Biophys* **292**, 479-486 (1992).
136. Gergely, P., Toth, B., Farkas, I. & Bot, G. Effect of Fructose 1-Phosphate on the Activation of Liver-Glycogen Synthase. *Biochemical Journal* **232**, 133-137 (1985).
137. Stalmans, W. & Gevers, G. The catalytic activity of phosphorylase b in the liver. With a note on the assay in the glycogenolytic direction. *The Biochemical journal* **200**, 327-336 (1981).
138. Maddaiah, V.T. & Madsen, N.B. Kinetics of purified liver phosphorylase. *The Journal of biological chemistry* **241**, 3873-3881 (1966).
139. Tan, A.W. & Nuttall, F.Q. Characteristics of the dephosphorylated form of phosphorylase purified from rat liver and measurement of its activity in crude liver preparations. *Biochim Biophys Acta* **410**, 45-60 (1975).
140. Thurston, J.H., Jones, E.M. & Hauhart, R.E. Decrease and Inhibition of Liver-Glycogen Phosphorylase after Fructose - Experimental Model for Study of Hereditary Fructose Intolerance. *Diabetes* **23**, 597-604 (1974).

141. Krebs, H.A. Equilibria in transamination systems. *The Biochemical journal* **54**, 82-86 (1953).
142. Rakhmanova, T.I. & Popova, T.N. Regulation of 2-oxoglutarate metabolism in rat liver by NADP-isocitrate dehydrogenase and aspartate aminotransferase. *Biochemistry-Moscow* **71**, 211-217 (2006).
143. Palmieri, L. et al. Citrin and aralar1 are Ca2+-stimulated aspartate/glutamate transporters in mitochondria. *Embo Journal* **20**, 5060-5069 (2001).
144. Lanoue, K.F., Duszynski, J., Watts, J.A. & Mckee, E. Kinetic-Properties of Aspartate Transport in Rat-Heart Mitochondrial Inner Membranes. *Archives of Biochemistry and Biophysics* **195**, 578-590 (1979).
145. Dierks, T. & Kramer, R. Asymmetric Orientation of the Reconstituted Aspartate Glutamate Carrier from Mitochondria. *Biochimica Et Biophysica Acta* **937**, 112-126 (1988).
146. Murphy, E., Coll, K.E., Viale, R.O., Tischler, M.E. & Williamson, J.R. Kinetics and Regulation of the Glutamate-Aspartate Translocator in Rat-Liver Mitochondria. *Journal of Biological Chemistry* **254**, 8369-8376 (1979).
147. Indiveri, C., Dierks, T., Kramer, R. & Palmieri, F. Reaction-Mechanism of the Reconstituted Oxoglutarate Carrier from Bovine Heart-Mitochondria. *European journal of biochemistry* **198**, 339-347 (1991).
148. Palmieri, F., Quagliar.E & Klingenb.M Kinetics and Specificity of Oxoglutarate Carrier in Rat-Liver Mitochondria. *European journal of biochemistry* **29**, 408-& (1972).
149. Euler, H., Adler, E. & Günther, G. in Hoppe-Seyler´s Zeitschrift für physiologische Chemie, Vol. 249 1 (1937).
150. Ostro, M.J. & Fondy, T.P. Isolation and Characterization of Multiple Molecular-Forms of Cytosolic Nad-Linked Glycerol-3-Phosphate Dehydrogenase from Normal and Neoplastic Rabbit Tissues. *Journal of Biological Chemistry* **252**, 5575-5583 (1977).
151. White, H.B. & Kaplan, N.O. Purification and Properties of 2 Types of Diphosphopyridine Nucleotide-Linked Glycerol 3-Phosphate Dehydrogenases from Chicken Breast Muscle and Chicken Liver. *Journal of Biological Chemistry* **244**, 6031-& (1969).
152. Garrib, A. & Mcmurray, W.C. Purification and Characterization of Glycerol-3-Phosphate Dehydrogenase (Flavin-Linked) from Rat-Liver Mitochondria. *Journal of Biological Chemistry* **261**, 8042-8048 (1986).
153. Glock, G.E. & Mclean, P. Further Studies on the Properties and Assay of Glucose 6-Phosphate Dehydrogenase and 6-Phosphogluconate Dehydrogenase of Rat Liver. *Biochemical Journal* **55**, 400-408 (1953).
154. Taketa, K. & Pogell, B.M. Effect of Palmityl Coenzyme a on Glucose 6-Phosphate Dehydrogenase and Other Enzymes. *Journal of Biological Chemistry* **241**, 720-& (1966).
155. Corpas, F.J., Garciasalguero, L., Peragon, J. & Lupianez, J.A. Kinetic-Properties of Hexose-Monophosphate Dehydrogenases .1. Isolation and Partial-Purification of Glucose-6-Phosphate-Dehydrogenase from Rat-Liver and Kidney Cortex. *Life Sciences* **56**, 179-189 (1994).
156. Bauer, H.P., Srihari, T., Jochims, J.C. & Hofer, H.W. 6-Phosphogluconolactonase - Purification, Properties and Activities in Various Tissues. *European journal of biochemistry* **133**, 163-168 (1983).
157. Villet, R.H. & Dalziel, K. The nature of the carbon dioxide substrate and equilibrium constant of the 6-phosphogluconate dehydrogenase reaction. *The Biochemical journal* **115**, 633-638 (1969).
158. Procsal, D. & Holten, D. Purification and Properties of Rat-Liver 6-Phosphogluconate Dehydrogenase - Activity at Normal in-Vivo Concentration of Coenzyme. *Biochemistry* **11**, 1310-& (1972).
159. Weisz, K.S., Schofield, P.J. & Edwards, M.R. Human-Brain 6-Phosphogluconate Dehydrogenase - Purification and Kinetic-Properties. *Journal of Neurochemistry* **44**, 510-517 (1985).
160. Horecker, B.L. & Hurwitz, J. The purification of phosphoketopentoepimerase from Lactobacillus pentosus and the preparation of xylulose 5-phosphate. *The Journal of biological chemistry* **223**, 993-1008 (1956).
161. Akana, J. et al. D-ribulose 5-phosphate 3-epimerase: Functional and structural relationships to members of the ribulose-phosphate binding (beta/alpha)(8)-barrel superfamily. *Biochemistry* **45**, 2493-2503 (2006).
162. Tabachnick, M., Srere, P.A., Cooper, J. & Racker, E. The oxidative pentose phosphate cycle. III. The interconversion of ribose 5-phosphate, ribulose 5-phosphate and xylulose 5-phosphate. *Arch Biochem Biophys* **74**, 315-325 (1958).
163. Kiely, M.E., Stuart, A.L. & Wood, T. Partial-Purification and Kinetic Properties of Ribose-5-Phosphate Ketol-Isomerase and Ribulose-5-Phosphate 3-Epimerase from Various Sources. *Biochimica Et Biophysica Acta* **293**, 534-541 (1973).
164. Venkataraman, R. & Racker, E. Mechanism of Action of Transaldolase .1. Crystallization and Properties of Yeast Enzyme. *Journal of Biological Chemistry* **236**, 1876-& (1961).

165. Sprenger, G.A., Schorken, U., Sprenger, G. & Sahm, H. Transaldolase-B of Escherichia-Coli K-12 - Cloning of Its Gene, Talb, and Characterization of the Enzyme from Recombinant Strains. *Journal of Bacteriology* **177**, 5930-5936 (1995).
166. Heinrich, P.C., Morris, H.P. & Weber, G. Behavior of Transaldolase (Ec-2.2.1.2) and Transketolase (Ec-2.2.1.1) Activities in Normal, Neoplastic, Differentiating, and Regenerating Liver. *Cancer Research* **36**, 3189-3197 (1976).
167. Datta, A.G. & Racker, E. Mechanism of action of transketolase. I. Properties of the crystalline yeast enzyme. *The Journal of biological chemistry* **236**, 617-623 (1961).
168. Paoletti, F. Purification and Properties of Transketolase from Fresh Rat-Liver. *Archives of Biochemistry and Biophysics* **222**, 489-496 (1983).
169. Takeuchi, T., Nishino, K. & Itokawa, Y. Purification and Characterization of, and Preparation of an Antibody to, Transketolase from Human Red-Blood-Cells. *Biochimica Et Biophysica Acta* **872**, 24-32 (1986).
170. Meshalkina, L.E., Solovjeva, O.N. & Kochetov, G.A. Interaction of Transketolase from Human Tissues with Substrates. *Biochemistry-Moscow* **76**, 1061-1064 (2011).
171. Novello, F. & Mclean, P. Pentose Phosphate Pathway of Glucose Metabolism - Measurement of Non-Oxidative Reactions of Cycle. *Biochemical Journal* **107**, 775-& (1968).
172. Halperin, M.L., Fritz, I.B. & Robinson, B.H. Effects of Palmitoyl Coa on Citrate and Malate Transport by Rat-Liver Mitochondria. *Proceedings of the National Academy of Sciences of the United States of America* **69**, 1003-& (1972).
173. Bisaccia, F., Depalma, A., Prezioso, G. & Palmieri, F. Kinetic Characterization of the Reconstituted Tricarboxylate Carrier from Rat-Liver Mitochondria. *Biochimica Et Biophysica Acta* **1019**, 250-256 (1990).
174. Bisaccia, F., Depalma, A., Dierks, T., Kramer, R. & Palmieri, F. Reaction-Mechanism of the Reconstituted Tricarboxylate Carrier from Rat-Liver Mitochondria. *Biochimica Et Biophysica Acta* **1142**, 139-145 (1993).
175. Guynn, R.W. & Veech, R.L. The equilibrium constants of the adenosine triphosphate hydrolysis and the adenosine triphosphate-citrate lyase reactions. *The Journal of biological chemistry* **248**, 6966-6972 (1973).
176. Singh, M., Richards, E.G., Mukherjee, A. & Srere, P.A. Structure of Atp Citrate Lyase from Rat-Liver - Physicochemical Studies and Proteolytic Modification. *Journal of Biological Chemistry* **251**, 5242-5250 (1976).
177. Houston, B. & Nimmo, H.G. Effects of Phosphorylation on the Kinetic-Properties of Rat-Liver Atp-Citrate Lyase. *Biochimica Et Biophysica Acta* **844**, 233-239 (1985).
178. Inoue, H., Suzuki, F., Fukunish.K, Adachi, K. & Takeda, Y. Studies on Atp Citrate Lyase of Rat Liver .I. Purification and Some Properties. *Journal of Biochemistry* **60**, 543-& (1966).
179. Carlson, C.A. & Kim, K.H. Differential Effects of Metabolites on Active and Inactive Forms of Hepatic Acetyl Coa Carboxylase. *Archives of Biochemistry and Biophysics* **164**, 490-501 (1974).
180. Jamil, H. & Madsen, N.B. Phosphorylation State of Acetyl-Coenzyme-a Carboxylase .1. Linear Inverse Relationship to Activity Ratios at Different Citrate Concentrations. *Journal of Biological Chemistry* **262**, 630-637 (1987).
181. Cheng, D. et al. Expression, purification, and characterization of human and rat acetyl coenzyme A carboxylase (ACC) isozymes. *Protein Expression and Purification* **51**, 11-21 (2007).
182. Hashimot.T & Numa, S. Kinetic Studies on Reaction Mechanism and Citrate Activation of Liver Acetyl Coenzyme-a Carboxylase. *European journal of biochemistry* **18**, 319-& (1971).
183. Kim, Y.S. & Kolattukudy, P.E. Purification and Properties of Malonyl-Coa Decarboxylase from Rat-Liver Mitochondria and Its Immunological Comparison with Enzymes from Rat-Brain, Heart, and Mammary-Gland. *Archives of Biochemistry and Biophysics* **190**, 234-246 (1978).
184. Aprahamian, S.A., Arslanian, M.J. & Wakil, S.J. Comparative Studies on the Kinetic-Parameters and Product Analyses of Chicken and Rat-Liver and Yeast Fatty-Acid Synthetase. *Comparative Biochemistry and Physiology B-Biochemistry & Molecular Biology* **71**, 577-582 (1982).
185. Westergaard, N., Madsen, P. & Lundgren, K. Characterization of glycerol uptake and glycerol kinase activity in rat hepatocytes cultured under different hormonal conditions. *Biochim Biophys Acta* **1402**, 261-268 (1998).
186. Robinson, J. & Newsholme, E.A. Some properties of hepatic glycerol kinase and their relation to the control of glycerol utilization. *The Biochemical journal* **112**, 455-464 (1969).

187. Yamashita, S. & Numa, S. Partial purification and properties of glycerophosphate acyltransferase from rat liver. Formation of 1-acylglycerol 3-phosphate from sn-glycerol 3-phosphate and palmityl coenzyme A. *European journal of biochemistry* **31**, 565-573 (1972).
188. Yada, R., Ide, H. & Nakazawa, Y. In vitro effects of chlorpromazine on glycerol-3-phosphate acyl transferase and 1-acylglycerol-3-phosphate acyltransferase in rat liver microsomes. *Biochem Pharmacol* **35**, 4083-4087 (1986).
189. Yamashita, A. et al. Topology of acyltransferase motifs and substrate specificity and accessibility in 1-acyl-sn-glycero-3-phosphate acyltransferase 1. *Biochim Biophys Acta* **1771**, 1202-1215 (2007).
190. Han, G.S. & Carman, G.M. Characterization of the human LPIN1-encoded phosphatidate phosphatase isoforms. *The Journal of biological chemistry* **285**, 14628-14638 (2010).
191. Coleman, R. & Bell, R.M. Triacylglycerol synthesis in isolated fat cells. Studies on the microsomal diacylglycerol acyltransferase activity using ethanol-dispersed diacylglycerols. *The Journal of biological chemistry* **251**, 4537-4543 (1976).
192. Hosaka, K., Schiele, U. & Numa, S. Diacylglycerol acyltransferase from rat liver microsomes. Separation and acyl-donor specificity. *European journal of biochemistry* **76**, 113-118 (1977).
193. Nakhoul, N.L. et al. Characteristics of renal Rhbg as an NH(4)(+) transporter. *American Journal of Physiology-Renal Physiology* **288**, F170-F181 (2005).
194. Soboll, S., Lenzen, C., Rettich, D., Grundel, S. & Ziegler, B. Characterization of Glutamine Uptake in Rat-Liver Mitochondria. *European journal of biochemistry* **197**, 113-117 (1991).
195. McGivan, J., Vadher, M., Lacey, J. & Bradford, N. Rat liver glutaminase. Regulation by reversible interaction with the mitochondrial membrane. *European journal of biochemistry* **148**, 323-327 (1985).
196. Lacey, J.H., Bradford, N.M., Joseph, S.K. & McGivan, J.D. Increased activity of phosphate-dependent glutaminase in liver mitochondria as a result of glucagon treatment of rats. *The Biochemical journal* **194**, 29-33 (1981).
197. Hortelano, P., Garcia-Salguero, L., Alleyne, G.A. & Lupianez, J.A. Variations in the kinetic response of several different phosphate-dependent glutaminase isozymes during acute metabolic acidosis. *Mol Cell Biochem* **108**, 113-123 (1991).
198. McGivan, J.D., Lacey, J.H. & Joseph, S.K. Localization and some properties of phosphate-dependent glutaminase in disrupted liver mitochondria. *The Biochemical journal* **192**, 537-542 (1980).
199. Fahien, L.A. & Kmiotek, E. Regulation of glutamate dehydrogenase by palmitoyl-coenzyme A. *Arch Biochem Biophys* **212**, 247-253 (1981).
200. Lee, W.K., Shin, S., Cho, S.S. & Park, J.S. Purification and characterization of glutamate dehydrogenase as another isoprotein binding to the membrane of rough endoplasmic reticulum. *J Cell Biochem* **76**, 244-253 (1999).
201. Engel, P.C. & Chen, S.S. A product-inhibition study of bovine liver glutamate dehydrogenase. *The Biochemical journal* **151**, 305-318 (1975).
202. Chee, P.Y., Dahl, J.L. & Fahien, L.A. Purification and Properties of Rat-Brain Glutamate-Dehydrogenase. *Journal of Neurochemistry* **33**, 53-& (1979).
203. Porter, R.K. Mammalian mitochondrial inner membrane cationic and neutral amino acid carriers. *Biochimica Et Biophysica Acta-Bioenergetics* **1459**, 356-362 (2000).
204. Aires, C.C. et al. New insights on the mechanisms of valproate-induced hyperammonemia: inhibition of hepatic N-acetylglutamate synthase activity by valproyl-CoA. *J Hepatol* **55**, 426-434 (2011).
205. Coude, F.X., Grimber, G., Parvy, P. & Kamoun, P. N-Acetyl glutamate synthetase in human liver: regulation of activity by L-arginine and N-acetylglutamate. *Biochem Biophys Res Commun* **102**, 1016-1020 (1981).
206. Shigesada, K. & Tatibana, M. N-Acetylglutamate synthetase from rat-liver mitochondria. Partial purification and catalytic properties. *European journal of biochemistry* **84**, 285-291 (1978).
207. Meijer, A.J., Van Woerkom, G.M., Wanders, R.J. & Lof, C. Transport of N-acetylglutamate in rat-liver mitochondria. *European journal of biochemistry* **124**, 325-330 (1982).
208. Reglero, A., Rivas, J., Mendelson, J., Wallace, R. & Grisolia, S. Deacylation and transacetylation of acetyl glutamate and acetyl ornithine in rat liver. *FEBS Lett* **81**, 13-17 (1977).
209. Meyer, J. & Vignais, P.M. Kinetic study of glutamate transport in rat liver mitochondria. *Biochim Biophys Acta* **325**, 375-384 (1973).
210. Palmieri, F., Indiveri, C., Bisaccia, F. & Iacobazzi, V. Mitochondrial metabolite carrier proteins: Purification, reconstitution, and transport studies. *Mitochondrial Biogenesis and Genetics, Pt A* **260**, 349-369 (1995).
211. Imesch, E. & Rous, S. Partial purification of rat liver cytoplasmic acetyl-CoA synthetase; characterization of some properties. *Int J Biochem* **16**, 875-881 (1984).

212. Corvi, M.M., Soltys, C.L. & Berthiaume, L.G. Regulation of mitochondrial carbamoyl-phosphate synthetase 1 activity by active site fatty acylation. *The Journal of biological chemistry* **276**, 45704-45712 (2001).
213. McGivan, J.D., Bradford, N.M. & Mendes-Mourao, J. The regulation of carbamoyl phosphate synthase activity in rat liver mitochondria. *The Biochemical journal* **154**, 415-421 (1976).
214. Kerson, L.A. & Appel, S.H. Kinetic studies on rat liver carbamyl phosphate synthetase. *The Journal of biological chemistry* **243**, 4279-4285 (1968).
215. Lusty, C.J., Jilka, R.L. & Nietsch, E.H. Ornithine transcarbamylase of rat liver. Kinetic, physical, and chemical properties. *The Journal of biological chemistry* **254**, 10030-10036 (1979).
216. Indiveri, C., Palmieri, L. & Palmieri, F. Kinetic characterization of the reconstituted ornithine carrier from rat liver mitochondria. *Biochim Biophys Acta* **1188**, 293-301 (1994).
217. Rochovansky, O., Kodowaki, H. & Ratner, S. Biosynthesis of urea. Molecular and regulatory properties of crystalline argininosuccinate synthetase. *The Journal of biological chemistry* **252**, 5287-5294 (1977).
218. Rochovansky, O. & Ratner, S. Biosynthesis of urea. XII. Further studies on argininosuccinate synthetase: substrate affinity and mechanism of action. *The Journal of biological chemistry* **242**, 3839-3849 (1967).
219. Raushel, F.M. & Nygaard, R. Kinetic mechanism of bovine liver argininosuccinate lyase. *Arch Biochem Biophys* **221**, 143-147 (1983).
220. O'Brien, W.E. & Barr, R.H. Argininosuccinate lyase: purification and characterization from human liver. *Biochemistry* **20**, 2056-2060 (1981).
221. Garganta, C.L. & Bond, J.S. Assay and kinetics of arginase. *Anal Biochem* **154**, 388-394 (1986).
222. Glass, R.D. & Knox, W.E. Arginase isozymes of rat mammary gland, liver, and other tissues. *The Journal of biological chemistry* **248**, 5785-5789 (1973).
223. Hediger, M.A. et al. Structure, regulation and physiological roles of urea transporters. *Kidney Int* **49**, 1615-1623 (1996).
224. Atlante, A., Passarella, S., Giannattasio, S. & Quagliariello, E. Fumarate permeation in rat liver mitochondria: fumarate/malate and fumarate/phosphate translocators. *Biochem Biophys Res Commun* **132**, 8-18 (1985).
225. Palmieri, F., Prezioso, G. & Quagliar.E Kinetic Study of Dicarboxylate Carrier in Rat Liver Mitochondria. *European journal of biochemistry* **22**, 66-& (1971).
226. Hopper, S. & Segal, H.L. Kinetic studies of rat liver glutamicalanine transaminase. *The Journal of biological chemistry* **237**, 3189-3195 (1962).
227. Low, S.Y., Taylor, P.M., Hundal, H.S., Pogson, C.I. & Rennie, M.J. Transport of L-glutamine and L-glutamate across sinusoidal membranes of rat liver. Effects of starvation, diabetes and corticosteroid treatment. *The Biochemical journal* **284 (Pt 2)**, 333-340 (1992).
228. Joseph, S.K., Bradford, N.M. & McGivan, J.D. Characteristics of the transport of alanine, serine and glutamine across the plasma membrane of isolated rat liver cells. *The Biochemical journal* **176**, 827-836 (1978).
229. Nakagawa, H. & Kimura, H. Properties of Crystalline Serine Dehydratase of Rat Liver. *Journal of Biochemistry* **66**, 669-& (1969).
230. Deuel, T.F., Louie, M. & Lerner, A. Glutamine-Synthetase from Rat-Liver Purification, Properties, and Preparation of Specific Antisera. *Journal of Biological Chemistry* **253**, 6111-6118 (1978).
231. Tiemeier, D.C. & Milman, G. Chinese-Hamster Liver Glutamine Synthetase - Purification, Physical and Biochemical Properties. *Journal of Biological Chemistry* **247**, 2272-& (1972).
232. Durrington, P.N., Newton, R.S., Weinstein, D.B. & Steinberg, D. Effects of insulin and glucose on very low density lipoprotein triglyceride secretion by cultured rat hepatocytes. *J Clin Invest* **70**, 63-73 (1982).
233. Jamil, H. et al. Microsomal Triglyceride Transfer Protein - Specificity of Lipid-Binding and Transport. *Journal of Biological Chemistry* **270**, 6549-6554 (1995).
234. Ong, K.T., Mashek, M.T., Bu, S.Y., Greenberg, A.S. & Mashek, D.G. Adipose Triglyceride Lipase Is a Major Hepatic Lipase That Regulates Triacylglycerol Turnover and Fatty Acid Signaling and Partitioning. *Hepatology* **53**, 116-126 (2011).
235. Osterlund, T. et al. Domain-structure analysis of recombinant rat hormone-sensitive lipase. *Biochemical Journal* **319**, 411-420 (1996).
236. Ikeda, Y., Okamura, K. & Fujii, S. Purification and Characterization of Rat-Liver Microsomal Monoacylglycerol Lipase in Comparison to Other Esterases. *Biochimica Et Biophysica Acta* **488**, 128-139 (1977).

237. Rokosz, L.L. et al. Human cytoplasmic 3-hydroxy-3-methylglutaryl coenzyme A synthase: expression, purification, and characterization of recombinant wild-type and Cys129 mutant enzymes. *Arch Biochem Biophys* **312**, 1-13 (1994).
238. Menahan, L.A., Hron, W.T., Hinkelman, D.G. & Miziorko, H.M. Interrelationships between 3-hydroxy-3-methylglutaryl-CoA synthase, acetoacetyl-CoA and ketogenesis. *European journal of biochemistry* **119**, 287-294 (1981).
239. Tuinstra, R.L., Wang, C.Z., Mitchell, G.A. & Miziorko, H.M. Evaluation of 3-hydroxy-3-methylglutaryl-coenzyme A lyase arginine-41 as a catalytic residue: use of acetyldithio-coenzyme A to monitor product enolization. *Biochemistry* **43**, 5287-5295 (2004).
240. Tucker, G.A. & Dawson, A.P. The kinetics of rat liver and heart mitochondrial beta-hydroxybutyrate dehydrogenase. *The Biochemical journal* **179**, 579-581 (1979).
241. Halestrap, A.P. Pyruvate and ketone-body transport across the mitochondrial membrane. Exchange properties, pH-dependence and mechanism of the carrier. *The Biochemical journal* **172**, 377-387 (1978).
242. Broer, S. et al. Characterization of the high-affinity monocarboxylate transporter MCT2 in Xenopus laevis oocytes. *The Biochemical journal* **341 (Pt 3)**, 529-535 (1999).
243. Ito, M., Fukui, T., Kamokari, M., Saito, T. & Tomita, K. Purification and characterization of acetoacetyl-CoA synthetase from rat liver. *Biochim Biophys Acta* **794**, 183-193 (1984).
244. Middleton, B. The kinetic mechanism and properties of the cytoplasmic acetoacetyl-coenzyme A thiolase from rat liver. *The Biochemical journal* **139**, 109-121 (1974).
245. Skaff, D.A. & Miziorko, H.M. A visible wavelength spectrophotometric assay suitable for high-throughput screening of 3-hydroxy-3-methylglutaryl-CoA synthase. *Analytical Biochemistry* **396**, 96-102 (2010).
246. Clinkenbeard, K.D., Sugiyama, T. & Lane, M.D. Cytosolic 3-hydroxy-3-methylglutaryl-CoA synthase from chicken liver. *Methods Enzymol* **35**, 160-167 (1975).
247. Montgomery, C., Pei, Z.T., Watkins, P.A. & Miziorko, H.M. Identification and Characterization of an Extramitochondrial Human 3-Hydroxy-3-methylglutaryl-CoA Lyase. *Journal of Biological Chemistry* **287**, 33227-33236 (2012).
248. Young, N.L., Saudek, C.D., Crawford, S.A. & Zuckerbrod, S.L. Recovery and Activation of Hydroxymethylglutaryl Co-Enzyme a Reductase from Rat Small-Intestine. *Journal of Lipid Research* **23**, 257-265 (1982).
249. Kawachi, T. & Rudney, H. Solubilization and purification of beta-hydroxy-beta-methylglutaryl coenzyme A reductase from rat liver. *Biochemistry* **9**, 1700-1705 (1970).
250. Tanaka, R.D., Schafer, B.L., Lee, L.Y., Freudenberger, J.S. & Mosley, S.T. Purification and regulation of mevalonate kinase from rat liver. *The Journal of biological chemistry* **265**, 2391-2398 (1990).
251. Qiu, Y. & Li, D. Bifunctional inhibitors of mevalonate kinase and mevalonate 5-diphosphate decarboxylase. *Org Lett* **8**, 1013-1016 (2006).
252. Bazaes, S. et al. Pig liver phosphomevalone kinase. 1. Purification and properties. *Biochemistry* **19**, 2300-2304 (1980).
253. Toth, M.J., Huwyler, L. & Park, J. Purification of rat liver mevalonate pyrophosphate decarboxylase. *Prep Biochem Biotechnol* **26**, 47-51 (1996).
254. Qiu, Y. & Li, D. Inhibition of mevalonate 5-diphosphate decarboxylase by fluorinated substrate analogs. *Biochim Biophys Acta* **1760**, 1080-1087 (2006).
255. Holloway, P.J. & Popjak, G. Isopentyl pyrophosphate isomerase from pig liver. *The Biochemical journal* **104**, 25P (1967).
256. Shah, D.H., Cleland, W.W. & Porter, J.W. The Partial Purification, Properties, and Mechanism of Action of Pig Liver Isopentenyl Pyrophosphate Isomerase. *The Journal of biological chemistry* **240**, 1946-1956 (1965).
257. Ramos-Valdivia, A.C., van der Heijden, R., Verpoorte, R. & Camara, B. Purification and characterization of two isoforms of isopentenyl-diphosphate isomerase from elicitor-treated Cinchona robusta cells. *European journal of biochemistry* **249**, 161-170 (1997).
258. Dorsey, J.K., Dorsey, J.A. & Porter, J.W. The purification and properties of pig liver geranyl pyrophosphate synthetase. *The Journal of biological chemistry* **241**, 5353-5360 (1966).
259. Benedict, C.R., Kett, J. & Porter, J.W. Properties of farnesyl pyrophosphate synthetase of pig liver. *Arch Biochem Biophys* **110**, 611-621 (1965).
260. Shechter, I. et al. Solubilization, purification, and characterization of a truncated form of rat hepatic squalene synthetase. *The Journal of biological chemistry* **267**, 8628-8635 (1992).

261. Abe, I. et al. Green tea polyphenols: novel and potent inhibitors of squalene epoxidase. *Biochem Biophys Res Commun* **268**, 767-771 (2000).
262. Nagumo, A., Kamei, T., Sakakibara, J. & Ono, T. Purification and characterization of recombinant squalene epoxidase. *J Lipid Res* **36**, 1489-1497 (1995).
263. Jahnke, L. & Klein, H.P. Oxygen requirements for formation and activity of the squalene epoxidase in Saccharomyces cerevisiae. *J Bacteriol* **155**, 488-492 (1983).
264. Kusano, M., Abe, I., Sankawa, U. & Ebizuka, Y. Purification and some properties of squalene-2,3-epoxide: lanosterol cyclase from rat liver. *Chem Pharm Bull (Tokyo)* **39**, 239-241 (1991).
265. Nitahara, Y., Aoyama, Y., Horiuchi, T., Noshiro, M. & Yoshida, Y. Purification and characterization of rat sterol 14-demethylase P450 (CYP51) expressed in Escherichia coli. *Journal of Biochemistry* **126**, 927-933 (1999).
266. Hamdane, D. et al. Structure and Function of an NADPH-Cytochrome P450 Oxidoreductase in an Open Conformation Capable of Reducing Cytochrome P450. *Journal of Biological Chemistry* **284**, 11374-11384 (2009).
267. Gaylor, J.L. & Mason, H.S. Investigation of Component Reactions of Oxidative Sterol Demethylation - Evidence against Participation of Cytochrome P-450. *Journal of Biological Chemistry* **243**, 4966-& (1968).
268. Paik, Y.K., Trzaskos, J.M., Shafiee, A. & Gaylor, J.L. Microsomal enzymes of cholesterol biosynthesis from lanosterol. Characterization, solubilization, and partial purification of NADPH-dependent delta 8,14-steroid 14-reductase. *The Journal of biological chemistry* **259**, 13413-13423 (1984).
269. Gibbons, G.F., Mitropoulos, K.A. & Pullinger, C.R. Lanosterol 14-Alpha-Demethylase - Metabolism of Some Potential Intermediates by Cell-Free Systems from Rat-Liver. *Biochemical and Biophysical Research Communications* **69**, 781-789 (1976).
270. Brady, D.R., Mattingly, T.W. & Gaylor, J.L. Simplified Assay of 4-Methyl Sterol Oxidase of Liver-Microsomes. *Analytical Biochemistry* **70**, 413-423 (1976).
271. Rahimtula, A.D. & Gaylor, J.L. Partial purification of a microsomal sterol 4 -carboxylic acid decarboxylase. *The Journal of biological chemistry* **247**, 9-15 (1972).
272. Miller, W.L., Kalafer, M.E., Gaylor, J.L. & Delwiche, C.V. Investigation of Component Reactions of Oxidative Sterol Demethylation . Study of Aerobic and Anaerobic Processes. *Biochemistry* **6**, 2673-& (1967).
273. Billheimer, J.T., Alcorn, M. & Gaylor, J.L. Solubilization and Partial-Purification of a Microsomal 3-Ketosteroid Reductase of Cholesterol-Biosynthesis. *Archives of Biochemistry and Biophysics* **211**, 430-438 (1981).
274. Delaunoit, Y., Zhao, H.F., Belanger, A., Labrie, F. & Simard, J. Expression of Liver-Specific Member of the 3-Beta-Hydroxysteroid Dehydrogenase Family, an Isoform Possessing an Almost Exclusive 3-Ketosteroid Reductase-Activity. *Journal of Biological Chemistry* **267**, 4513-4517 (1992).
275. Paik, Y.K., Billheimer, J.T., Magolda, R.L. & Gaylor, J.L. Microsomal-Enzymes of Cholesterol-Biosynthesis from Lanosterol - Solubilization and Purification of Steroid 8-Isomerase. *Journal of Biological Chemistry* **261**, 6470-6477 (1986).
276. Kang, M.K., Kim, C.K., Johng, T.N. & Paik, Y.K. Cholesterol biosynthesis from lanosterol: regulation and purification of rat hepatic sterol 8-isomerase. *J Biochem* **117**, 819-823 (1995).
277. Kawata, S., Trzaskos, J.M. & Gaylor, J.L. Microsomal-Enzymes of Cholesterol-Biosynthesis from Lanosterol - Purification and Characterization of Delta-7-Sterol 5-Desaturase of Rat-Liver Microsomes. *Journal of Biological Chemistry* **260**, 6609-6617 (1985).
278. Moebius, F.F., Fitzky, B.U., Lee, J.N., Paik, Y.K. & Glossman, H. Molecular cloning and expression of the human Delta 7-sterol reductase. *Proceedings of the National Academy of Sciences of the United States of America* **95**, 1899-1902 (1998).
279. Bae, S.H. & Paik, Y.K. Cholesterol biosynthesis from lanosterol: development of a novel assay method and characterization of rat liver microsomal lanosterol Delta(24)-reductase. *Biochemical Journal* **326**, 609-616 (1997).
280. Kaduce, T.L., Schmidt, R.W. & Spector, A.A. Acylcoenzyme-a-Cholesterol Acyltransferase Activity - Solubilization and Reconstitution in Liposomes. *Biochemical and Biophysical Research Communications* **81**, 462-468 (1978).
281. Balasubramaniam, S., Mitropoulos, K.A. & Venkatesan, S. Rat-Liver Acyl-Coa - Cholesterol Acyltransferase. *European journal of biochemistry* **90**, 377-383 (1978).
282. Crabb, D.W., Bosron, W.F. & Li, T.K. Steady-State Kinetic-Properties of Purified Rat-Liver Alcohol-Dehydrogenase - Application to Predicting Alcohol Elimination Rates Invivo. *Archives of Biochemistry and Biophysics* **224**, 299-309 (1983).

283. Koivula, T. & Koivusalo, M. Different Forms of Rat-Liver Aldehyde Dehydrogenase and Their Subcellular-Distribution. *Biochimica Et Biophysica Acta* **397**, 9-23 (1975).
284. Rout, U.K. & Weiner, H. Involvement of Serine-74 in the Enzyme-Coenzyme Interaction of Rat-Liver Mitochondrial Aldehyde Dehydrogenase. *Biochemistry* **33**, 8955-8961 (1994).
285. Jackson, V.N. & Halestrap, A.P. The kinetics, substrate, and inhibitor specificity of the monocarboxylate (lactate) transporter of rat liver cells determined using the fluorescent intracellular pH indicator, 2',7'-bis(carboxyethyl)-5(6)-carboxyfluorescein. *Journal of Biological Chemistry* **271**, 861-868 (1996).
286. Gould, G.W. & Holman, G.D. The Glucose-Transporter Family - Structure, Function and Tissue-Specific Expression. *Biochemical Journal* **295**, 329-341 (1993).
287. Hers, H.G. Aldose Reductase. *Biochimica Et Biophysica Acta* **37**, 120-126 (1960).
288. Endo, S. et al. Characterization of a rat NADPH-dependent aldo-keto reductase (AKR1B13) induced by oxidative stress. *Chemico-Biological Interactions* **178**, 151-157 (2009).
289. Cuatreca.P & Segal, S. Mammalian Galactose Dehydrogenase .2. Properties Substrate Specificity and Developmental Changes. *Journal of Biological Chemistry* **241**, 5910-& (1966).
290. Segal, S. & Cuatreca.P Oxidation of C14galactose by Patients with Congenital Galactosemia - Evidence for a Direct Oxidative Pathway. *American Journal of Medicine* **44**, 340-& (1968).
291. Asada, Y. et al. Dimeric Crystal Structure of Rabbit L-Gulonate 3-Dehydrogenase/lambda-Crystallin: Insights into the Catalytic Mechanism. *Journal of Molecular Biology* **401**, 906-920 (2010).
292. Hickman, J. & Ashwell, G. Purification and Properties of D-Xylulokinase in Liver. *Journal of Biological Chemistry* **232**, 737-748 (1958).
293. Dills, W.L., Parsons, P.D., Westgate, C.L. & Komplin, N.J.A. Assay, Purification, and Properties of Bovine Liver D-Xylulokinase. *Protein Expression and Purification* **5**, 259-265 (1994).
294. Cuatreca.P & Segal, S. Mammalian Galactokinase - Developmental and Adaptive Characteristics in Rat Liver. *Journal of Biological Chemistry* **240**, 2382-& (1965).
295. Chu, X.S., Li, N., Liu, X.J. & Li, D. Functional studies of rat galactokinase. *Journal of Biotechnology* **141**, 142-146 (2009).
296. Bertoli, D. & Segal, S. Developmental Aspects and Some Characteristics of Mammalian Galactose 1-Phosphate Uridyltransferase. *Journal of Biological Chemistry* **241**, 4023-& (1966).
297. Gross, W. & Schnarrenberger, C. Purification and Characterization of a Galactose-1-Phosphate-Udp-Glucose Uridyltransferase from the Red Alga Galdieria-Sulphuraria. *European journal of biochemistry* **234**, 258-263 (1995).
298. Segal, S. & Rogers, S. Nucleotide Inhibition of Mammalian Liver Galactose-I-Phosphate Uridylyltransferase. *Biochimica Et Biophysica Acta* **250**, 351-& (1971).
299. Geeganage, S. & Frey, P.A. Significance of metal ions in galactose-1-phosphate uridylyltransferase: An essential structural zinc and a nonessential structural iron. *Biochemistry* **38**, 13398-13406 (1999).
300. Fan, D.F. & Feingold, D.S. Nucleoside Diphosphate-Sugar 4-Epimerases .I. Uridine Diphosphate Glucose 4-Epimerase of Wheat Germ. *Plant Physiology* **44**, 599-& (1969).
301. Maxwell, E.S. The Enzymic Interconversion of Uridine Diphosphogalactose and Uridine Diphosphoglucose. *Journal of Biological Chemistry* **229**, 139-151 (1957).
302. Gulavita, S.J., Zhang, L.P., Dougherty, J.J. & Dain, J.A. Galactose-1-Phosphatase in Rat-Brain. *Journal of Neurochemistry* **57**, 520-526 (1991).
303. Colville, C.A., Seatter, M.J., Jess, T.J., Gould, G.W. & Thomas, H.M. Kinetic-Analysis of the Liver-Type (Glut2) and Brain-Type (Glut3) Glucose Transporters in Xenopus Oocytes - Substrate Specificities and Effects of Transport Inhibitors. *Biochemical Journal* **290**, 701-706 (1993).
304. Adelman, R.C., Ballard, F.J. & Weinhous.S Purification and Properties of Rat Liver Fructokinase. *Journal of Biological Chemistry* **242**, 3360-& (1967).
305. Parks, R.E., Bengershom, E. & Lardy, H.A. Liver Fructokinase. *Journal of Biological Chemistry* **227**, 231-242 (1957).
306. Lehninger, A.L., Sice, J. & Jensen, E.V. Effect of Substrate Structure on the Aldolase Equilibrium. *Biochimica Et Biophysica Acta* **17**, 285-287 (1955).
307. Cox, T.M., Odonnell, M.W. & Camilleri, M. Isolation and Characterization of a Mutant Liver Aldolase in Adult Hereditary Fructose Intolerance - Identification of the Enzyme Variant by Radioassay in Tissue Biopsy Specimens. *Journal of Clinical Investigation* **72**, 201-213 (1983).
308. Gefflaut, T., Blonski, C., Perie, J. & Willson, M. Class I aldolases: Substrate specificity, mechanism, inhibitors and structural aspects. *Progress in Biophysics & Molecular Biology* **63**, 301-340 (1995).
309. Frandsen, E.K. & Grunnet, N. Kinetic Properties of Triokinase from Rat Liver. *European journal of biochemistry* **23**, 588-& (1971).

310. Rivett, A.J., Smith, I.L. & Tipton, K.F. Purification of the High-Km Aldehyde Reductase from Rat-Brain and Liver and from Ox Brain. *Biochemical Journal* **197**, 473-481 (1981).
311. Sillero, M.A.G., Sillero, A. & Sols, A. Enzymes Involved in Fructose Metabolism in Liver and Glyceraldehyde Metabolic Crossroads. *European journal of biochemistry* **10**, 345-& (1969).
312. Leissing, N. & Mcguinness, E.T. Sorbitol Dehydrogenase from Rat-Liver. *Methods in Enzymology* **89**, 135-140 (1982).
313. Katayama, H., Kitagawa, Y. & Sugimoto, E. Purification of Rat-Liver Glycerate Kinase and Studies of Its Enzymatic and Immunological Properties. *Journal of Biochemistry* **88**, 765-773 (1980).
314. Aires, C.C.P. et al. Studies on the extra-mitochondrial CoA-ester formation of valproic and Delta(4)-valproic acids. *Biochimica Et Biophysica Acta-Molecular and Cell Biology of Lipids* **1771**, 533-543 (2007).
315. Aires, C.C.P. et al. Inhibition of hepatic carnitine palmitoyl-transferase I (CPT IA) by valproyl-CoA as a possible mechanism of valproate-induced steatosis. *Biochemical Pharmacology* **79**, 792-799 (2010).
316. Bulik, S., Holzhutter, H.G. & Berndt, N. The relative importance of kinetic mechanisms and variable enzyme abundances for the regulation of hepatic glucose metabolism - insights from mathematical modeling. *BMC Biol* **14**, 15 (2016).
317. la Fleur, S.E., Kalsbeek, A., Wortel, J., Fekkes, M.L. & Buijs, R.M. A daily rhythm in glucose tolerance: a role for the suprachiasmatic nucleus. *Diabetes* **50**, 1237-1243 (2001).
318. Frangioudakis, G., Gyte, A.C., Loxham, S.J. & Poucher, S.M. The intravenous glucose tolerance test in cannulated Wistar rats: a robust method for the in vivo assessment of glucose-stimulated insulin secretion. *J Pharmacol Toxicol Methods* **57**, 106-113 (2008).
319. Hara, E. & Saito, M. Diurnal changes in plasma glucose and insulin responses to oral glucose load in rats. *Am J Physiol* **238**, E463-466 (1980).
320. Balks, H.J. & Jungermann, K. Regulation of peripheral insulin/glucagon levels by rat liver. *European journal of biochemistry* **141**, 645-650 (1984).
321. Diaz, B. & Blazquez, E. Effect of pinealectomy on plasma glucose, insulin and glucagon levels in the rat. *Horm Metab Res* **18**, 225-229 (1986).
322. Patel, D.G. Lack of glucagon response to hypoglycemia in long-term experimental diabetic rats. *Diabetes* **32**, 55-60 (1983).
323. Wan, C.K. et al. Increased responses of glucagon and glucose production to hypoglycemia with intraperitoneal versus subcutaneous insulin treatment. *Metabolism* **49**, 984-989 (2000).
324. Zhou, H. et al. Regulation of alpha-cell function by the beta-cell during hypoglycemia in Wistar rats: the "switch-off" hypothesis. *Diabetes* **53**, 1482-1487 (2004).
325. Claus, T.H., El-Maghrabi, M.R. & Pilkis, S.J. Modulation of the phosphorylation state of rat liver pyruvate kinase by allosteric effectors and insulin. *The Journal of biological chemistry* **254**, 7855-7864 (1979).
326. Pilkis, S., Schlumpf, J., Pilkis, J. & Claus, T.H. Regulation of phosphofructokinase activity by glucagon in isolated rat hepatocytes. *Biochem Biophys Res Commun* **88**, 960-967 (1979).
327. Schudt, C. Regulation of glycogen synthesis in rat-hepatocyte cultures by glucose, insulin and glucocorticoids. *European journal of biochemistry* **97**, 155-160 (1979).
328. El-Maghrabi, M.R., Claus, T.H., Pilkis, J., Fox, E. & Pilkis, S.J. Regulation of rat liver fructose 2,6-bisphosphatase. *The Journal of biological chemistry* **257**, 7603-7607 (1982).
329. Bartrons, R., Hue, L., Van Schaftingen, E. & Hers, H.G. Hormonal control of fructose 2,6-bisphosphate concentration in isolated rat hepatocytes. *The Biochemical journal* **214**, 829-837 (1983).
330. Hartmann, H., Probst, I., Jungermann, K. & Creutzfeldt, W. Inhibition of glycogenolysis and glycogen phosphorylase by insulin and proinsulin in rat hepatocyte cultures. *Diabetes* **36**, 551-555 (1987).
331. Syed, N.A. & Khandelwal, R.L. Reciprocal regulation of glycogen phosphorylase and glycogen synthase by insulin involving phosphatidylinositol-3 kinase and protein phosphatase-1 in HepG2 cells. *Mol Cell Biochem* **211**, 123-136 (2000).
332. De Gasquet, P., Griglio, S., Pequignot-Planche, E. & Malewiak, M.I. Diurnal changes in plasma and liver lipids and lipoprotein lipase activity in heart and adipose tissue in rats fed a high and low fat diet. *J Nutr* **107**, 199-212 (1977).
333. Yamamoto, H., Nagai, K. & Nakagawa, H. Role of SCN in daily rhythms of plasma glucose, FFA, insulin and glucagon. *Chronobiol Int* **4**, 483-491 (1987).
334. Djordjevic, J. et al. The Effect of Fasting on the Diurnal Rhythm of Rat Acth and Corticosterone Secretion. *Archives of Biological Sciences* **60**, 541-546 (2008).
335. Spector, A.A. Fatty-Acid Binding to Plasma Albumin. *Journal of Lipid Research* **16**, 165-179 (1975).

336. Rose, R. & Klemcke, H.G. Relationship between Plasma Albumin Concentration and Plasma Volume in 5 Inbred Rat Strains. *Journal of the American Association for Laboratory Animal Science* **54**, 459-464 (2015).
337. Benavides, A., Siches, M. & Llobera, M. Circadian rhythms of lipoprotein lipase and hepatic lipase activities in intermediate metabolism of adult rat. *Am J Physiol* **275**, R811-817 (1998).
338. Stotz, E. & Bessey, O.A. The blood lactate-pyruvate relation and its use in experimental thiamine deficiency in pigeons. *Journal of Biological Chemistry* **143**, 625-631 (1942).
339. Dauchy, R.T. et al. Dark-phase light contamination disrupts circadian rhythms in plasma measures of endocrine physiology and metabolism in rats. *Comp Med* **60**, 348-356 (2010).
340. Dauchy, R.T. et al. Effects of spectral transmittance through standard laboratory cages on circadian metabolism and physiology in nude rats. *J Am Assoc Lab Anim Sci* **52**, 146-156 (2013).
341. Jeyaraj, D. et al. Klf15 orchestrates circadian nitrogen homeostasis. *Cell Metab* **15**, 311-323 (2012).
342. Minami, Y. et al. Measurement of internal body time by blood metabolomics. *Proc Natl Acad Sci U S A* **106**, 9890-9895 (2009).
343. Fernstrom, J.D., Larin, F. & Wurtman, R.J. Daily variations in the concentrations of individual amino acids in rat plasma. *Life Sci I* **10**, 813-819 (1971).
344. Weisiger, R., Gollan, J. & Ockner, R. Receptor for Albumin on the Liver-Cell Surface May Mediate Uptake of Fatty-Acids and Other Albumin-Bound Substances. *Science* **211**, 1048-1050 (1981).
345. Chambaz, J., Guillouzo, A., Cardot, P., Pepin, D. & Bereziat, G. Essential Fatty-Acid Uptake and Esterification in Primary Culture of Rat Hepatocytes. *Biochimica Et Biophysica Acta* **878**, 310-319 (1986).
346. Clark, C.M., Jr. & Scow, R.O. Effects of fasting and hypophysectomy on FFA uptake and ketone body production by the isolated, perfused rat liver. *Diabetes* **19**, 924-929 (1970).
347. Ishii-Iwamoto, E.L., Ferrarese, M.L., Constantin, J., Salgueiro-Pagadigorria, C. & Bracht, A. Effects of norepinephrine on the metabolism of fatty acids with different chain lengths in the perfused rat liver. *Mol Cell Biochem* **205**, 13-23 (2000).
348. Krebs, H.A., Wallace, P.G., Hems, R. & Freedland, R.A. Rates of ketone-body formation in the perfused rat liver. *The Biochemical journal* **112**, 595-600 (1969).
349. McGarry, J., Wright, P.H. & Foster, D.W. Hormonal control of ketogenesis. Rapid activation of hepatic ketogenic capacity in fed rats by anti-insulin serum and glucagon. *J Clin Invest* **55**, 1202-1209 (1975).
350. Goh, E.H. & Heimberg, M. Effects of free fatty acids on activity of hepatic microsomal 3-hydroxy-3-methylglutaryl coenzyme A reductase and on secretion of triglyceride and cholesterol by liver. *The Journal of biological chemistry* **252**, 2822-2826 (1977).
351. Solerargilaga, C. & Heimberg, M. Comparison of Metabolism of Free Fatty-Acid by Isolated Perfused Livers from Male and Female Rats. *Journal of Lipid Research* **17**, 605-615 (1976).
352. Windmueller, H.G. & Spaeth, A.E. De novo synthesis of fatty acid in perfused rat liver as a determinant of plasma lipoprotein production. *Arch Biochem Biophys* **122**, 362-369 (1967).
353. Brunengraber, H., Boutry, M. & Lowenstein, J.M. Fatty acid and 3- -hydroxysterol synthesis in the perfused rat liver. Including measurements on the production of lactate, pyruvate, -hydroxy-butyrate, and acetoacetate by the fed liver. *The Journal of biological chemistry* **248**, 2656-2669 (1973).
354. Sestoft, L. & Fleron, P. Kinetics of glycerol uptake by the perfused rat liver. Membrane transport, phosphorylation and effect on NAD redox level. *Biochim Biophys Acta* **375**, 462-471 (1975).
355. Simonen, P.P., Gylling, H.K. & Miettinen, T.A. Diabetes contributes to cholesterol metabolism regardless of obesity. *Diabetes care* **25**, 1511-1515 (2002).
356. Saheki, T. & Katunuma, N. Analysis of regulatory factors for urea synthesis by isolated perfused rat liver. I. Urea synthesis with ammonia and glutamine as nitrogen sources. *J Biochem* **77**, 659-669 (1975).
357. Kashiwagura, T., Wilson, D.F. & Erecinska, M. Oxygen dependence of cellular metabolism: the effect of O2 tension on gluconeogenesis and urea synthesis in isolated rat hepatocytes. *J Cell Physiol* **120**, 13-18 (1984).
358. Aw, T.Y. & Jones, D.P. Secondary bioenergetic hypoxia. Inhibition of sulfation and glucuronidation reactions in isolated hepatocytes at low O2 concentration. *The Journal of biological chemistry* **257**, 8997-9004 (1982).
359. Jones, D.P. & Mason, H.S. Gradients of O2 concentration in hepatocytes. *The Journal of biological chemistry* **253**, 4874-4880 (1978).
360. Exton, J.H. & Park, C.R. Control of Gluconeogenesis in the Perfused Liver of Normal and Adrenalectomized Rats. *The Journal of biological chemistry* **240**, 955-957 (1965).

361. Exton, J.H. & Park, C.R. Control of gluconeogenesis in liver. I. General features of gluconeogenesis in the perfused livers of rats. *The Journal of biological chemistry* **242**, 2622-2636 (1967).
362. Scholz, R., Hansen, W. & Thurman, R.G. Interaction of mixed-function oxidation with biosynthetic processes. 1. Inhibition of gluconeogenesis by aminopyrine in perfused rat liver. *European journal of biochemistry* **38**, 64-72 (1973).
363. Holness, M.J., MacLennan, P.A., Palmer, T.N. & Sugden, M.C. The disposition of carbohydrate between glycogenesis, lipogenesis and oxidation in liver during the starved-to-fed transition. *The Biochemical journal* **252**, 325-330 (1988).
364. Holness, M.J., Palmer, T.N., Worrall, E.B. & Sugden, M.C. Hepatic carbon flux after re-feeding in the glycogen-storage-disease (gsd/gsd) rat. *The Biochemical journal* **248**, 969-972 (1987).
365. Niewoehner, C.B. & Nuttall, F.Q. Relationship of hepatic glucose uptake to intrahepatic glucose concentration in fasted rats after glucose load. *Diabetes* **37**, 1559-1566 (1988).
366. Friedmann, B., Goodman, E.H., Jr. & Weinhouse, S. Effects of glucose feeding, cortisol, and insulin on liver glycogen synthesis in the rat. *Endocrinology* **81**, 486-496 (1967).
367. Sestoft, L. & Fleron, P. Determination of the kinetic constants of fructose transport and phosphorylation in the perfused rat liver. *Biochim Biophys Acta* **345**, 27-38 (1974).
368. Vilstrup, H., Keiding, S. & Vendsborg, P.B. Kinetics of galactose uptake by perfused rat livers: applicability of a family of models. *J Theor Biol* **101**, 335-344 (1983).
369. Lindros, K.O., Vihma, R. & Forsander, O.A. Utilization and metabolic effects of acetaldehyde and ethanol in the perfused rat liver. *The Biochemical journal* **126**, 945-952 (1972).
370. Kashiwagi, T., Ji, S., Lemasters, J.J. & Thurman, R.G. Rates of alcohol dehydrogenase-dependent ethanol metabolism in periportal and pericentral regions of the perfused rat liver. *Mol Pharmacol* **21**, 438-443 (1982).
371. Scholz, R. & Nohl, H. Mechanism of the stimulatory effect of fructose on ethanol oxidation in perfused rat liver. *European journal of biochemistry* **63**, 449-458 (1976).
372. Lindros, K.O., Oshino, N., Parrilla, R. & Williamson, J.R. Characteristics of ethanol and acetaldehyde oxidation on flavin and pyridine nucleotide fluorescence changes in perfused rat liver. *The Journal of biological chemistry* **249**, 7956-7963 (1974).
373. Reichardt, J.K.V., Packman, S. & Woo, S.L.C. Molecular Characterization of 2 Galactosemia Mutations - Correlation of Mutations with Highly Conserved Domains in Galactose-I-Phosphate Uridyl Transferase. *American Journal of Human Genetics* **49**, 860-867 (1991).
374. Timson, D.J. Functional analysis of disease-causing mutations in human UDP-galactose 4-epimerase. *Febs Journal* **272**, 6170-6177 (2005).
375. Edge, S.B. & Compton, C.C. The American Joint Committee on Cancer: the 7th edition of the AJCC cancer staging manual and the future of TNM. *Ann Surg Oncol* **17**, 1471-1474 (2010).
376. Cox, J. & Mann, M. MaxQuant enables high peptide identification rates, individualized p.p.b.-range mass accuracies and proteome-wide protein quantification. *Nat Biotechnol* **26**, 1367-1372 (2008).
377. Cox, J. et al. Andromeda: a peptide search engine integrated into the MaxQuant environment. *J Proteome Res* **10**, 1794-1805 (2011).
378. Cox, J. et al. Accurate proteome-wide label-free quantification by delayed normalization and maximal peptide ratio extraction, termed MaxLFQ. *Mol Cell Proteomics* **13**, 2513-2526 (2014).
