# Supplementary material for: HEPATOKIN1 is a biochemistry-based model of liver metabolism for applications in medicine and pharmacology
Source: Nat Commun. 2018 Jun 19;9:2386. doi: 10.1038/s41467-018-04720-9 (PMC6008457; doi:10.1038/s41467-018-04720-9)
Supplement: Supplementary file 3 — Description of Additional Supplementary Files [file 41467_2018_4720_MOESM3_ESM.pdf]

Description of Additional Supplementary Files

File Name: Supplementary Data 1
Description: Computed and measured metabolite concentrations.

File Name: Supplementary Data 2
Description: Absolute values of the time-average control coefficients.

File Name: Supplementary Data 3
Description: Fold-change of enzyme abundances (tumor vs. hepatocyte)

File Name: Supplementary Data 4
Description: Sensitivity coefficients (infinitesimal, finite) and elasticity coefficients
