# Supplementary material for: HEPATOKIN1 is a biochemistry-based model of liver metabolism for applications in medicine and pharmacology
Source: Nat Commun. 2018 Jun 19;9:2386. doi: 10.1038/s41467-018-04720-9 (PMC6008457; doi:10.1038/s41467-018-04720-9)
Supplement: Supplementary file 4 — Supplementary Data 1 [file 41467_2018_4720_MOESM4_ESM.pdf]

Supplementary Information to

A Biochemistry-Based Model of Liver Metabolism for Applications in Medicine and Pharmacology
N. Berndt et al.

Supplementary Data 1 – Computed and measured metabolite concentrations

Identifier	Name	Min conc. observed	Max conc. observed	compartment	citation	Min conc. simulated	Max conc. simulated
$acac$	Acetoacetate	0.06317763	1.60283991	cell	(Krebs, 1966) (Brosnan et al, 1970) (Veech et al, 1972) (Greenbaum et al, 1971) (Lagunas et al, 1970) (Rawat, 1968) (Veech et al, 1973) (Williamson et al, 1967) (Baquer et al, 1976) (Casazza et al, 1986) (Casazza & Veech, 1986a) (Parrilla et al, 1975) (Williamson et al, 1969) (Schofield et al, 1987)	9.19E-02	2.33E-01
$acac_{cyt}$				cytosol		9.08E-02	2.16E-01
$acac_{mito}$				mitochondrion		6.38E-03	9.98E-02
$acetate$	Acetate	1.05746036	1.05746036	cell	(Murthy & Steiner, 1973) (Knowles et al, 1974) (Guynn & Veech, 1974) (Richards et al, 1975)	5.66E-03	9.64E-03
$acetate_{cyt}$				cytosol		4.85E-03	8.26E-03
$acetate_{mito}$				mitochondrion		4.85E-03	8.26E-03
$acglu$	Acetylglutamate	0.01637939	0.32056798	cell	(Saheki et al, 1977) (Shigesada & Tatibana, 1971) (Zollner, 1981) (Saheki et al, 1978)	1.88E-01	3.23E-01
$acglu_{cyt}$				cytosol	(Shigesada & Tatibana, 1971)	1.61E-01	2.75E-01
$acglu_{mito}$				mitochondrion	(Shigesada & Tatibana, 1971)	1.66E-01	2.83E-01
$acoa$	Acetyl coenzyme A	0.02620702	0.27222539	cell	(Menahan et al, 1981) (Start & Newsholme, 1968) (Siess et al, 1978) (Greenbaum et al, 1971) (Kondrup & Grunnet, 1973) (Lagunas et al, 1970) (Rawat, 1968) (Veech et al, 1973) (Albe et al, 1990) (Baquer et al, 1976) (Siess et al, 1977) (Parrilla et al, 1975) (Williamson et al, 1969) (Krahenbuhl & Brass, 1991)	3.75E-02	1.11E-01

$acoa_{cyt}$		0.03332383	1.36	cytosol	(Siess et al, 1978) (Siess et al, 1978) (Garland et al, 1965) (Siess et al, 1977)	1.81E-02	6.11E-02
$acoa_{mito}$		0.006402	2.74	mitochondrion	(Siess et al, 1976) (Siess et al, 1978) (Matsuishi et al, 1991) (Garland et al, 1965) (Siess et al, 1977) (Latipaa et al, 1986) (Lopes-Cardozo & van den Bergh, 1974a) (Eaton et al, 1994)	1.15E-01	3.02E-01
adp	Adenosine diphosphate	1.13251754	4.60962719	cell	(Hems & Brosnan, 1970) (Veech et al, 1972) (Start & Newsholme, 1968) (Siess et al, 1978) (Greenbaum et al, 1971) (Lagunas et al, 1970) (Elbers et al, 1974) (Veech et al, 1973) (Albe et al, 1990) (Aw et al, 1987) (Kauffman et al, 1977) (Baquer et al, 1976) (Casazza et al, 1986) (Keppler et al, 1969) (Jackson et al, 1980) (Siess et al, 1977) (Keiding, 1973) (Parrilla et al, 1975) (Sestoft, 1974) (Guynn et al, 1974) (Woods & Krebs, 1973) (Williamson et al, 1969) (Siess et al, 1978)	2.06E+00	3.71E+00
adp_{cyt}		0.2899039	3.7	cytosol	(Tischler et al, 1977) (Elbers et al, 1974) (Aw et al, 1987) (Siess et al, 1977) (Soboll et al, 1978)	1.17E+00	2.32E+00
adp_{mito}		0.3655542	15.7122574	mitochondrion	(Siess et al, 1978) (Tischler et al, 1977) (Zuurendonk & Tager, 1974) (Elbers et al, 1974) (Aw et al, 1987) (Siess et al, 1977) (Soboll et al, 1978) (Siess, 1985)	5.31E+00	8.35E+00

akg	*Alpha*-ketoglutarate	0.06565143	1.60099261	cell	(Krebs, 1966) (Brosnan et al, 1970) (Veech et al, 1972) (Siess et al, 1978) (Greenbaum et al, 1971) (Frohman et al, 1951) (Parrilla et al, 1975) (Spydevold et al, 1973) (Rawat, 1968) (Veech et al, 1973) (Albe et al, 1990) (Guynn et al, 1986) (Williamson et al, 1967) (Kauffman et al, 1977) (Baquer et al, 1976) (Casazza et al, 1986) (Casazza & Veech, 1986a) (Casazza & Veech, 1986b) (Siess et al, 1977) (Woods & Krebs, 1973) (Williamson et al, 1969)	2.82E-01	1.97E+00
akg_{cyt}		0.04636359	1.23	cytosol	(Siess et al, 1978) (Greenbaum et al, 1971) (Spydevold et al, 1973) (Baquer et al, 1976) (Siess et al, 1977) (Parrilla et al, 1975) (Groen et al, 1982)	1.91E-01	1.36E+00
akg_{mito}		0.01	3.2	mitochondrion	(Siess et al, 1978) (Greenbaum et al, 1971) (Spydevold et al, 1973) (Baquer et al, 1976) (Siess et al, 1977) (Parrilla et al, 1975) (Groen et al, 1982)	5.45E-01	3.67E+00
ala	Alanine	0.46798246	8.63427632	cell	(Brosnan et al, 1970) (Greenbaum et al, 1971) (Parrilla et al, 1975) (Parrilla, 1978) (Ohta et al, 1995) (Albe et al, 1990) (Guynn et al, 1986) (Baquer et al, 1976) (Saheki et al, 1975)	1.76E-01	3.97E-01
ala_{cyt}		0.29	0.29	cytosol	(Groen et al, 1982)	1.76E-01	3.97E-01

amp	Adenosine monophosphate	0.3041886	1.74089474	cell	(Hems & Brosnan, 1970) (Veech et al, 1972) (Start & Newsholme, 1968) (Siess et al, 1978) (Greenbaum et al, 1971) (Lagunas et al, 1970) (Elbers et al, 1974) (Veech et al, 1973) (Kauffman et al, 1977) (Baquer et al, 1976) (Casazza et al, 1986) (Keppler et al, 1969) (Jackson et al, 1980) (Jackson et al, 1976) (Siess et al, 1977) (Keiding, 1973) (Parrilla et al, 1975) (Sestoft, 1974) (Guynn et al, 1974) (Woods & Krebs, 1973) (Williamson et al, 1969)	2.08E-01	1.21E+00
amp_{cyt}		0.03	0.7	cytosol	(Elbers et al, 1974) (Soboll et al, 1978)	2.08E-01	1.21E+00
$apoB$	Apolipoprotein B	126.132244	316.776389	cell	(Cartwright & Higgins, 1992)	1.91E-01	2.52E-01
arg	Arginine	0.04211842	0.07019737	cell	(Saheki et al, 1977) (Saheki et al, 1975)	3.15E-03	5.67E-03
arg_{cyt}				cytosol		2.70E-03	4.86E-03
arg_{mito}				mitochondrion		2.70E-03	4.86E-03
$argsucc$	Argininosuccinate	1.16995614	1.16995614	cell	(O & Choy, 1993)	6.76E-03	1.16E-02
$argsucc_{cyt}$		0.13	0.13	cytosol	(O & Choy, 1993)	6.76E-03	1.16E-02
asp	Aspartate	0.47	7.32	cell	(Brosnan et al, 1970) (Zakim et al, 1967) (Greenbaum et al, 1971) (Parrilla et al, 1975) (Parrilla, 1978) (Lagunas et al, 1970) (Spydevold et al, 1973) (Ohta et al, 1995) (Rawat, 1968) (Veech et al, 1973) (Albe et al, 1990) (Baquer et al, 1976) (Siess et al, 1977) (Saheki et al, 1975) (Zollner, 1981)	3.38E-02	2.34E-01

asp_{cyt}		0.66921491	17.09	cytosol	(Siess et al, 1978) (Greenbaum et al, 1971) (Spydevold et al, 1973) (Aw et al, 1987) (Baquer et al, 1976) (Siess et al, 1977) (Soboll et al, 1976) (Parrilla et al, 1975)	3.36E-02	2.33E-01
asp_{mito}		0.0358512	7.53	mitochondrion	(Siess et al, 1978) (Greenbaum et al, 1971) (Spydevold et al, 1973) (Ohta et al, 1995) (Aw et al, 1987) (Baquer et al, 1976) (Siess et al, 1977) (Soboll et al, 1976) (Parrilla et al, 1975)	1.21E-03	2.89E-03
atp	Adenosine triphosphate	1.00616228	9.26644737	cell	(Hems & Brosnan, 1970) (Veech et al, 1972) (Veech et al, 1970) (Start & Newsholme, 1968) (Siess et al, 1978) (Greenbaum et al, 1971) (Lagunas et al, 1970) (Elbers et al, 1974) (Rawat, 1968) (Veech et al, 1973) (Albe et al, 1990) (Aw et al, 1987) (Kauffman et al, 1977) (Baquer et al, 1976) (Casazza et al, 1986) (Keppler et al, 1969) (Jackson et al, 1980) (Keppler et al, 1974) (Jackson et al, 1976) (Weber et al, 1985) (Siess et al, 1977) (Keiding, 1973) (Parrilla et al, 1975) (Sestoft, 1974) (Berman et al, 1978) (Guynn et al, 1974) (Woods & Krebs, 1973)	4.74E+00	7.40E+00
atp_{cyt}		2.094521	7.35977096	cytosol	(Siess et al, 1978) (Tischler et al, 1977) (Elbers et al, 1974) (Aw et al, 1987) (Siess et al, 1977) (Soboll et al, 1978)	4.47E+00	6.62E+00

atp_{mito}		1.4	16.98	mitochondrion	(Siess et al, 1978) (Tischler et al, 1977) (Elbers et al, 1974) (Aw et al, 1987) (Siess et al, 1977) (Soboll et al, 1978)	1.65E+00	4.69E+00
$bhbut$	*Beta*-hydroxybutyrate	0.17783333	4.25864035	cell	(Krebs, 1966) (Brosnan et al, 1970) (Veech et al, 1972) (Lagunas et al, 1970) (Rawat, 1968) (Veech et al, 1973) (Williamson et al, 1967) (Baquer et al, 1976) (Casazza et al, 1986) (Parrilla et al, 1975) (Williamson et al, 1969) (Schofield et al, 1987)	9.19E-02	2.17E-01
$bhbut_{cyt}$	*Beta*-hydroxybutyrate			cytosol		7.45E-02	1.36E-01
$bhbut_{mito}$				mitochondrion		9.92E-02	4.97E-01
$bpg13$	1,3 Bisphosphoglycerate			Cell		2.47E-04	8.71E-04
$bpg13_{cyt}$				Cytosol		2.47E-04	8.71E-04
$c10coa_{cyt}$	Decanoyl coenzyme A			cytosol		1.25E-03	1.95E-03
$c10coa_{mito}$		0	0.009603	mitochondrion	(Eaton et al, 1994)	5.39E-08	2.11E-07
$c12coa_{cyt}$	Lauryl coenzyme A			cytosol		1.25E-03	1.95E-03
$c12coa_{mito}$		0.0083226	0.0211266	mitochondrion	(Eaton et al, 1994)	5.26E-08	2.06E-07
$c14coa_{cyt}$	Myristoyl coenzyme A			cytosol		1.25E-03	1.95E-03
$c14coa_{mito}$		0.025608	0.147246	mitochondrion	(Eaton et al, 1994)	2.10E-07	8.20E-07
$c16car$	Palmitoylcarnitine	0.02807895	0.43522368	cell	(Kondrup & Grunnet, 1973) (Schofield et al, 1987) (Krahenbuhl & Brass, 1991) (Greenbaum et al, 1971)	1.85E-04	7.42E-04
$c16car_{cyt}$				cytosol		6.04E-05	2.42E-04
$c16car_{mito}$		0.083226	0.176055	mitochondrion	(Eaton et al, 1994)	7.49E-04	3.00E-03

$c16coa$	Palmitoyl coenzyme A	0.01801732	0.33928728	cell	(Greenbaum et al, 1971) (Kondrup & Grunnet, 1973) (Lagunas et al, 1970) (Baquer et al, 1976) (Parrilla et al, 1975) (Williamson et al, 1969) (Krahenbuhl & Brass, 1991) (Chen et al, 1992) (Ellis et al, 2000)	7.47E-03	2.52E-02
$c16coa_{cyt}$				cytosol		7.47E-03	2.52E-02
$c16coa_{er}$				ER		0.00E+00	0.00E+00
$c16coa_{mito}$		0.09603	0.403326	mitochondrion	(Lopes-Cardozo & van den Bergh, 1974b) (Eaton et al, 1994)	3.10E-07	1.21E-06
$c16_{cyt}$	Palmitate			cytosol		5.79E-06	1.36E-05
$c4coa_{cyt}$	Butyryl coenzyme A			cytosol		2.99E-04	4.69E-04
$c4coa_{mito}$		0	0	mitochondrion	(Watmough et al, 1989)	1.56E-08	6.09E-08
$c5coa_{mito}$	Valoryl coenzym A			mitochondrion		0.00E+00	3.06E-26
$c6coa_{cyt}$	Hexanoyl coenzyme A			cytosol		1.69E-03	2.66E-03
$c6coa_{mito}$		0	0	mitochondrion	(Watmough et al, 1989)	2.61E-08	1.02E-07
$c8coa_{cyt}$	Octanoyl coenzyme A			cytosol		3.11E-03	4.88E-03
$c8coa_{mito}$		0	0.0048015	mitochondrion	(Eaton et al, 1994)	9.35E-09	3.65E-08
car	Carnitine	0.27142982	0.54051974	cell	(Kondrup & Grunnet, 1973) (Krahenbuhl & Brass, 1991)	9.16E-01	9.16E-01
car_{cyt}				cytosol		4.00E-01	4.00E-01
car_{mito}		2	5	mitochondrion	(Indiveri et al, 1994)	3.10E+00	3.10E+00
$chol$	Cholesterol	5.82245843	43.4614788	cell	(Fushimi et al, 2006) (Avoy et al, 1965) (Endo, 1980) (Ide et al, 1978) (Purushothama et al, 1994) (Hariharan et al, 1996) (Hariharan et al, 1992) (Purushothama et al, 1995)	6.22E-06	1.76E-05
$chol_{er}$				ER		1.50E-05	4.23E-05

ce	Cholesterol ester	0.3318302	7.63209454	cell	(Andersen et al, 1979) (Ide et al, 1978)	1.35E-01	3.17E-01
ce_{er}				ER		3.09E-03	4.61E-03
ce_{ld}				Lipid Droplet		1.34E-01	3.15E-01
ce_{vldl}				VLDL		1.07E-05	1.55E-05
cit	Citrate	0.11291005	1.53434496	cell	(Veech et al, 1972) (Zakim et al, 1967) (Start & Newsholme, 1968) (Siess et al, 1978) (Greenbaum et al, 1971) (Spencer & Lowenstein, 1967) (Potter & Busch, 1950) (Gordon, 1961) (Frohman et al, 1951) (Parmeggiani & Bowman, 1963) (Kalkhoff et al, 1966) (Lagunas et al, 1970) (Elbers et al, 1974) (Spydevold et al, 1973) (Rawat, 1968) (Veech et al, 1973) (Albe et al, 1990) (Guynn et al, 1986) (Aw et al, 1987) (Baquer et al, 1976) (Siess et al, 1977) (Parrilla et al, 1975) (Woods & Krebs, 1973) (Williamson et al, 1969) (Schofield et al, 1987)	2.17E-01	2.95E+00
cit_{cyt}		0.0263549	2.11	cytosol	(Siess et al, 1976) (Siess et al, 1978) (Greenbaum et al, 1971) (Elbers et al, 1974) (Spydevold et al, 1973) (Aw et al, 1987) (Baquer et al, 1976) (Siess et al, 1977) (Soboll et al, 1976) (Parrilla et al, 1975)	1.39E-01	1.92E+00
cit_{mito}		0.2253504	11.82	mitochondrion	(Siess et al, 1976) (Siess et al, 1978) (Greenbaum et al, 1971) (Elbers et al, 1974) (Spydevold et al, 1973) (Aw et al, 1987) (Baquer et al, 1976) (Siess et al, 1977) (Soboll et al, 1976) (Parrilla et al, 1975)	4.68E-01	6.20E+00

cl	Chloride	22.1	75.504386	cell	(Claret & Mazet, 1972) (Williams et al, 1971b) (Williams et al, 1971a) (Berthon et al, 1980) (Claret et al, 1973)	8.01E+00	8.01E+00
cl_{cyt}		9,3	14,2	cytosol	(Williams et al, 1971a)	8.00E+00	8.00E+00
cl_{mito}		1	46	mitochondrion	(Werkheiser & Bartley, 1957) (Amoore & Bartley, 1958)	4.15E-02	4.60E-02
cmp	Carbamoyl phosphate	0.02480307	0.0374386	cell	(Pausch et al, 1985)	4.48E-03	6.20E-03
cmp_{mito}				mitochondrion		2.69E-02	3.72E-02
$co2_{cyt}$	Carbon dioxide			cytosol		5.00E+00	5.00E+00
$co2_{mito}$	Carbon dioxide			mitochondrion		5.00E+00	5.00E+00
coa	Coenzyme A	0.05124408	1.01420347	cell	(Zakim et al, 1967) (Siess et al, 1978) (Greenbaum et al, 1971) (Kondrup & Grunnet, 1973) (Lagunas et al, 1970) (Menahan et al, 1981) (Veech et al, 1973) (Albe et al, 1990) (Baquer et al, 1976) (Siess et al, 1977) (Parrilla et al, 1975) (Williamson et al, 1969) (Krahenbuhl & Brass, 1991)	3.45E-01	4.58E-01
coa_{cyt}		0.00982763	0.1	cytosol	(Siess et al, 1978) (Garland et al, 1965) (Siess et al, 1977)	5.73E-02	1.36E-01
coa_{mito}		0.006402	3.08	mitochondrion	(Siess et al, 1978) (Garland et al, 1965) (Siess et al, 1977) (Latipaa et al, 1986) (Lopes-Cardozo & van den Bergh, 1974a) (Lopes-Cardozo & van den Bergh, 1974b)	1.72E+00	1.94E+00
ctl	Citrulline	0.06785746	0.23399123	cell	(Saheki et al, 1977) (Saheki & Katunuma, 1975) (Saheki et al, 1978)	6.26E-02	1.39E-01
ctl_{cyt}				cytosol		2.32E-04	1.14E-03
ctl_{mito}				mitochondrion		1.78E-02	3.39E-02
$cytc$	cytochrome c	0.0121638	0.0121638	cell	(Krahenbuhl et al, 1991)	2.50E-02	2.50E-02

$cytc_{ox_{mm}}$	Oxidized cytochrome c			Membrane		4.00E-01	4.21E-01
$cytc_{red_{mm}}$	Reduced cytochrome c			Membrane		7.88E-02	9.97E-02
$dmemcoa_{mito}$	D-Methyl-Malonylcoa			mitochondrion		2.65E-04	6.62E-04
dag	Diacylglycerol			cell		1.28E+00	1.34E+00
dag_{er}				ER		4.86E-03	8.20E-03
dag_{ld}				Lipid droplet		1.28E+00	1.34E+00
$ddimdihtmasca$				cell		5.37E-08	5.69E-08
$ddimdihtmasca_{er}$				ER		1.30E-07	1.37E-07
$ddimkdihtmas$				cell		4.73E-07	5.01E-07
$ddimkdihtmas_{er}$				ER		1.14E-06	1.21E-06
$ddimktmas$				Cell		1.41E-07	1.53E-07
$ddimktmas_{er}$				ER		3.40E-07	3.68E-07
$ddimtmasca$				Cell		1.60E-08	1.73E-08
$ddimtmasca_{er}$				ER		3.86E-08	4.18E-08
$desmo$	Desmosterol			Cell		9.21E-08	9.96E-08
$desmo_{er}$				ER		2.22E-07	2.40E-07
$dhap$	Dihydroxyacetone phosphate	0.02620702	0.32758772	cell	(Veech et al, 1972) (Greenbaum et al, 1971) (Zakim et al, 1967) (Veech et al, 1970) (Veech et al, 1969) (Kalkhoff et al, 1966) (Parrilla, 1978) (Lagunas et al, 1970) (Rawat, 1968) (Veech et al, 1973) (Albe et al, 1990) (Guynn et al, 1986) (Kauffman et al, 1977) (Baquer et al, 1976) (Casazza et al, 1986) (Casazza & Veech, 1986b) (Sestoft, 1974) (Woods & Krebs, 1973) (Williamson et al, 1969)	1.79E-02	5.14E-02
$dhap_{cyt}$				cytosol		1.79E-02	5.14E-02

$dhchol_{er}$	Dehydrocholesterol			ER		5.34E-07	5.66E-07
$dhdesmo_{er}$				ER		4.07E-08	4.40E-08
$dhlath_{er}$	Dihydrolanosterol			ER		9.33E-08	1.00E-07
$dihffmas_{er}$				ER		1.49E-07	1.62E-07
$dihlan_{er}$				ER		3.03E-07	3.20E-07
$dihtmas_{er}$				ER		1.57E-06	1.65E-06
$dmdihtmas_{er}$				ER		1.57E-06	1.65E-06
$dmdihtmasca_{er}$				ER		1.30E-07	1.37E-07
$dmkdihtmas_{er}$				ER		1.14E-06	1.21E-06
$dmktmas_{er}$				ER		3.40E-07	3.68E-07
$dmpp_{cyt}$				ER		2.49E-06	2.49E-06
$dmtmas_{er}$				ER		4.68E-07	5.04E-07
$dmtmasca_{er}$				ER		3.86E-08	4.18E-08
$ec10coa_{mito}$	Enoyl-decanoyl coenzyme A	0	0	mitochondrion	(Watmough et al, 1989)	1.22E-09	3.83E-09
$ec12coa_{mito}$	Enoyl-lauryl coenzyme A	0	0.0048015	mitochondrion	(Eaton et al, 1994)	1.50E-09	4.80E-09
$ec14coa_{mito}$	Enoyl-myristoyl coenzyme A	0.0006402	0.0166452	mitochondrion	(Eaton et al, 1994)	1.78E-09	5.75E-09
$ec16coa_{mito}$	Enoyl-palmitoyl coenzyme A	0.009603	0.09603	mitochondrion	(Eaton et al, 1994)	1.36E-09	4.31E-09
$ec4coa_{mito}$	Enoyl-butyryl coenzyme A	0	0	mitochondrion	(Watmough et al, 1989)	3.97E-07	1.10E-06
$ec5_{coa_{mito}}$	Enoyl-valeryl coenzyme A			mitochondrion		0.00E+00	8.92E-29
$ec6coa_{mito}$	Enoyl-hexanoyl coenzyme A	0	0	mitochondrion	(Watmough et al, 1989)	1.09E-09	3.41E-09
$ec8coa_{mito}$	Enoyl-octanoyl coenzyme A	0	0	mitochondrion	(Watmough et al, 1989)	1.18E-09	3.70E-09
$evalcoa_{mito}$	Enoyl-valproyl coenzyme A			mitochondrion		0.00E+00	9.90E-22

$e4p$	Erythrose 4-phosphate	0.00467982	0.00935965	cell	(Greenbaum et al, 1971) (Casazza & Veech, 1986a)	7.79E-04	1.81E-03
$e4p_{cyt}$				cytosol		7.79E-04	1.81E-03
$ethanol_{cyt}$	Ethanol			cytosol		0.00E+00	7.06E-40
$etffad_{mito}$	Electron-transferring flavoprotein - FAD			mitochondrion		1.92E-01	1.98E-01
$etffadh2_{mito}$	Electron-transferring flavoprotein – $FADH_2$			mitochondrion		2.01E-03	7.83E-03
$etfq_{mito}$	Electron-transferring flavoprotein - Q			mitochondrion		2.00E-01	2.00E-01
$etfqh2_{mito}$	Electron-transferring flavoprotein – QH_2			mitochondrion		2.32E-04	3.69E-04
$ffmas$				cell		1.92E-07	2.04E-07
$ffmas_{er}$				ER		4.64E-07	4.93E-07
fpp	farnesylpyrophosphate			cell		2.88E-06	2.96E-06
fpp_{cyt}		0.01263553	0.01263553	cytosol	(Keller, 1996)	2.88E-06	2.96E-06
fru_{cyt}	fructose			cytosol		0.00E+00	0.00E+00
$fru16bp$	Fructose-1,6-bisphosphate	0.00934516	0.09359649	cell	(Hems & Brosnan, 1970) (Zakim et al, 1967) (Greenbaum et al, 1971) (Veech et al, 1969) (Kalkhoff et al, 1966) (Lagunas et al, 1970) (Rawat, 1968) (Albe et al, 1990) (Kauffman et al, 1977) (Baquer et al, 1976) (Parrilla et al, 1975) (Sestoft, 1974) (Woods & Krebs, 1973) (Williamson et al, 1969)	1.18E-04	1.24E-03

$fru16bp_{cyt}$				cytosol		1.18E-04	1.24E-03
$fru1p$	Fructose-1-phosphate	1.12315789	5.8497807	cell	(Sestoft, 1974)	0.00E+00	0.00E+00
$fru1p_{cyt}$				cytosol		0.00E+00	0.00E+00
$fru26bp$	Fructose-2,6-bisphosphate	0.0025739	0.02433509	cell	(Fushimi et al, 2001) (Nishimura et al, 1994) (Hue et al, 1981) (Hue et al, 1982) (Neely et al, 1981)	4.23E-04	2.16E-03
$fru26bp_{cyt}$				cytosol		4.23E-04	2.16E-03
$fru6p$	Fructose-6-phosphate	0.02028407	0.23399123	cell	(Zakim et al, 1967) (Start & Newsholme, 1968) (Greenbaum et al, 1971) (Lagunas et al, 1970) (Rawat, 1968) (Albe et al, 1990) (Gardner et al, 1993) (Baquer et al, 1976) (Casazza et al, 1986) (Casazza & Veech, 1986a) (Casazza & Veech, 1986b) (Parrilla et al, 1975) (Sestoft, 1974) (Woods & Krebs, 1973) (Williamson et al, 1969)	6.16E-02	8.97E-02
$fru6p_{cyt}$				cytosol		6.16E-02	8.97E-02
fum	Fumarate	0.108	0.95181511	cell	(Frohman et al, 1951) (Albe et al, 1990)	3.84E-02	6.64E-01
fum_{cyt}				cytosol		2.71E-02	4.61E-01
fum_{mito}		8.4280308	8.4280308	mitochondrion	(Bellamy, 1962)	6.75E-02	1.22E+00
$g3p$	Glycerol 3-phosphate	0.19269866	1.6379386	cell	(Hems & Brosnan, 1970) (Veech et al, 1972) (Zakim et al, 1967) (Veech et al, 1970) (Greenbaum et al, 1971) (Kalkhoff et al, 1966) (Parrilla et al, 1975) (Parrilla, 1978) (Lagunas et al, 1970) (Rawat, 1968) (Veech et al, 1973) (Albe et al, 1990) (Baquer et al, 1976) (Casazza & Veech, 1986b) (Sestoft, 1974) (Woods & Krebs, 1973) (Williamson et al, 1969)	1.62E-01	4.28E-01

$g3p_{cyt}$				cytosol		1.62E-01	4.28E-01
gal	Galactose	0	0	cell	(Keiding, 1973) (Berman et al, 1978)	7.77E-05	1.14E-04
gal_{cyt}				cytosol		7.77E-05	1.14E-04
$gal1p$	Galactose 1-phosphate	0.21059211	0.53817982	cell	(Keiding, 1973) (Berman et al, 1978)	2.35E-03	3.44E-03
$gal1p_{cyt}$				cytosol		2.35E-03	3.44E-03
$galactitol_{cyt}$	Galactitol			cytosol		1.41E-07	2.07E-07
$galactonate_{cyt}$	Galactonate			cytosol		5.18E-08	7.62E-08
gdp	Guanosine diphosphate	0.18485307	0.39076535	cell	(Jackson et al, 1980)	2.08E-01	4.43E-01
gdp_{cyt}				cytosol		1.63E-01	3.73E-01
gdp_{mito}				mitochondrion		2.65E-01	4.17E-01
glc	Glucose	0.37438596	20.5912281	cell	(Greenbaum et al, 1971) (Kalkhoff et al, 1966) (Rawat, 1968) (Albe et al, 1990) (Casazza & Veech, 1986a) (Casazza & Veech, 1986b) (Keppler & Decker, 1969) (Keiding, 1973) (Sestoft, 1974) (Berman et al, 1978) (Woods & Krebs, 1973)	1.05E+01	1.13E+01
glc_{cyt}				cytosol		7.45E+00	8.02E+00
glc_{er}				ER		7.45E+00	8.02E+00
$glc1p$	Glucose 1-phosphate	0.0115909	0.25739035	cell	(Greenbaum et al, 1971) (Albe et al, 1990) (Gardner et al, 1993) (Keppler & Decker, 1969) (Parrilla et al, 1975) (Berman et al, 1978) (Guynn et al, 1974) (Woods & Krebs, 1973)	1.23E-02	1.81E-02
$glc1p_{cyt}$				cytosol		1.23E-02	1.81E-02

$glc6p$	Glucose 6-phosphate	0.01965526	0.75345175	cell	(Hems & Brosnan, 1970) (Veech et al, 1972) (Zakim et al, 1967) (Start & Newsholme, 1968) (Greenbaum et al, 1971) (Kalkhoff et al, 1966) (Parrilla et al, 1975) (Parrilla, 1978) (Lagunas et al, 1970) (Rawat, 1968) (Veech et al, 1973) (Albe et al, 1990) (Gardner et al, 1993) (Kauffman et al, 1977) (Baquer et al, 1976) (Casazza et al, 1986) (Casazza & Veech, 1986a) (Casazza & Veech, 1986b) (Siess et al, 1977) (Sestoft, 1974) (Berman et al, 1978) (Guynn et al, 1974) (Woods & Krebs, 1973) (Williamson et al, 1969)	2.81E-01	4.15E-01
$glc6p_{cyt}$		0.14	0.14	cytosol	(Siess et al, 1977)	1.99E-01	2.94E-01
$glc6p_{er}$				ER		1.99E-01	2.93E-01
gln	Glutamine	5.11	10.3424123	cell	(Albe et al, 1990) (Saheki et al, 1975)	4.55E+00	5.74E+00
gln_{cyt}				cytosol		4.12E+00	5.13E+00
gln_{mito}				mitochondrion		2.58E+00	3.67E+00
glu	Glutamate	2.02028026	12.190943	cell	(Brosnan et al, 1970) (Veech et al, 1972) (Siess et al, 1978) (Greenbaum et al, 1971) (Parrilla et al, 1975) (Parrilla, 1978) (Lagunas et al, 1970) (Spydevold et al, 1973) (Ohta et al, 1995) (Rawat, 1968) (Veech et al, 1973) (Albe et al, 1990) (Guynn et al, 1986) (Williamson et al, 1967) (Kauffman et al, 1977) (Baquer et al, 1976) (Siess et al, 1977) (Saheki et al, 1975) (Zollner, 1981) (Woods & Krebs, 1973)	4.26E+00	1.01E+01

glu_{cyt}		2.2	11.91	cytosol	(Krebs, 1966) (Siess et al, 1978) (Greenbaum et al, 1971) (Spydevold et al, 1973) (Aw et al, 1987) (Baquer et al, 1976) (Siess et al, 1977) (Soboll et al, 1976) (Parrilla et al, 1975) (Groen et al, 1982)	2.56E+00	6.11E+00
glu_{mito}		0.90	26.69	mitochondrion	(Siess et al, 1978) (Greenbaum et al, 1971) (Spydevold et al, 1973) (Aw et al, 1987) (Bellamy, 1962) (Baquer et al, 1976) (Siess et al, 1977) (Soboll et al, 1976) (Zollner, 1981) (Parrilla et al, 1975) (Groen et al, 1982)	1.02E+01	2.40E+01
$glyc$	Glycerol	0.07	0.28078947	cell	(Greenbaum et al, 1971) (Sestoft, 1974)	7.62E-03	1.21E-02
$glyc_{cyt}$				cytosol		7.62E-03	1.21E-02
$glycerate_{cyt}$				cytosol		0.00E+00	0.00E+00
$glyglc$	Glycogen	9.94462719	711.333333	cell	(Start & Newsholme, 1968) (Kalkhoff et al, 1966) (Rawat, 1968) (Hornbrook et al, 1966)	2.00E+01	1.81E+02
gpp	Geranoylpyrophosphate			Cell		7.91E-06	7.92E-06
gpp_{cyt}				cytosol		7.91E-06	7.92E-06
gra_{cyt}	Glyceraldehyde			cytosol		0.00E+00	0.00E+00
$grap$	Glyceraldehyde-3-phosphate	0.00156774	0.016	cell	(Veech et al, 1970) (Greenbaum et al, 1971) (Veech et al, 1969) (Lagunas et al, 1970) (Rawat, 1968) (Albe et al, 1990) (Baquer et al, 1976) (Casazza et al, 1986) (Casazza & Veech, 1986a) (Casazza & Veech, 1986b) (Woods & Krebs, 1973) (Williamson et al, 1969)	8.15E-04	2.34E-03
$grap_{cyt}$	glyceraldehyde-3-phosphate			cytosol		8.15E-04	2.34E-03

gtp	Guanosine triphosphate	0.50448509	0.86810746	cell	(Jackson et al, 1980) (Keppler et al, 1974) (Jackson et al, 1976) (Weber et al, 1985)	6.41E-01	8.76E-01
gtp_{cyt}				cytosol		6.27E-01	8.37E-01
gtp_{mito}				mitochondrion		8.31E-02	2.35E-01
h_{cyt}	Proton			cytosol		1.00E-04	1.00E-04
h_{mito}				mitochondrion		2.47E-05	2.58E-05
$hco3_{cyt}$	Bicarbonate					2.50E+01	2.50E+01
$hco3_{mito}$	Bicarbonate					2.50E+01	2.50E+01
$hmgcoa$	3-Hydroxy-3-methyl-glutaryl coenzyme A	0.0080961	0.01202715	cell	(Fushimi et al, 2006)	1.09E-02	2.99E-02
$hmgcoa_{cyt}$				cytosol		1.05E-02	2.58E-02
$hmgcoa_{mito}$				mitochondrion		2.16E-03	2.54E-02
ipp	Isopentenyl pyrophosphate			cell		1.61E-05	1.71E-05
ipp_{cyt}				cytosol		1.61E-05	1.71E-05
$isocit$	Isocitrate	0.029	0.06592323	cell	(Albe et al, 1990) (Veech et al, 1972) (Greenbaum et al, 1971) (Frohman et al, 1951) (Spydevold et al, 1973) (Veech et al, 1973) (Guynn et al, 1986) (Casazza et al, 1986) (Casazza & Veech, 1986a) (Casazza & Veech, 1986b) (Parrilla et al, 1975)	7.80E-03	1.03E-01
$isocit_{mito}$		0.042	0.042	mitochondrion	(Parrilla et al, 1975)	4.68E-02	6.20E-01
$kc10coa_{mito}$	*Beta*-ketoacyl-decanoyl coenzyme A	0	0	mitochondrion	(Watmough et al, 1989) (Bartlett & Eaton, 2004)	1.33E-09	5.20E-09
$kc12coa_{mito}$	*Beta*-ketoacyl-lauryl coenzyme A	0	0	mitochondrion	(Watmough et al, 1989) (Bartlett & Eaton, 2004)	8.87E-10	3.48E-09

$kc14coa_{mito}$	*Beta*-ketoacyl-myristoyl coenzyme A	0	0	mitochondrion	(Watmough et al, 1989) (Bartlett & Eaton, 2004)	1.39E-09	5.47E-09
$kc16coa_{mito}$	*Beta*-ketoacyl-palmitoyl coenzyme A	0	0	mitochondrion	(Watmough et al, 1989) (Bartlett & Eaton, 2004)	1.38E-09	5.42E-09
$kc4coa$	Acetoacetyl coenzyme A	0.00060838	0.00060838	cell	(Menahan et al, 1981)	7.46E-07	1.16E-05
$kc4coa_{cyt}$				cytosol		2.83E-07	8.29E-06
$kc4coa_{mito}$				mitochondrion		2.71E-06	2.01E-05
$kc5_{coa_{mito}}$	*Beta*-ketoacyl-pentanoyl coenzyme A			mitochondrion		0.00E+00	1.68E-27
$kc6_{coa_{mito}}$	*Beta*-ketoacyl-hexanoyl coenzyme A	0	0	mitochondrion	(Watmough et al, 1989) (Bartlett & Eaton, 2004)	4.34E-09	1.70E-08
$kc8_{coa_{mito}}$	*Beta*-ketoacyl-octanoyl coenzyme A	0	0	mitochondrion	(Watmough et al, 1989) (Bartlett & Eaton, 2004)	1.99E-09	7.80E-09
k	Potassium	113	235.161184	cell	(Macfarlane & Spencer, 1953) (Claret & Mazet, 1972) (Williams et al, 1971b) (Williams et al, 1971a) (Berthon et al, 1980)	1.61E+02	1.62E+02
k_{cyt}		79.4144502	171	cytosol	(Aw et al, 1987) (Macfarlane & Spencer, 1953) (Williams et al, 1971b) (Williams et al, 1971a) (Claret-Berthon et al, 1977)	1.40E+02	1.40E+02
k_{mito}		7	142	mitochondrion	(Aw et al, 1987) (Werkheiser & Bartley, 1957) (Amoore & Bartley, 1958) (Macfarlane & Spencer, 1953) (Harris & van Dam, 1968)	1.29E+02	1.30E+02

$kvalcoa_{mito}$	*Beta*-ketoacyl-valpoyl coenzyme A			mitochondrion		0.00E+00	3.03E-24
kga	3-Dehydro-L-gulonate			cell		2.56E-07	3.78E-07
kga_{cyt}				cytosol		2.56E-07	3.78E-07
$lc10coa_{mito}$	*Beta*-hydroxyacyl-decanoyl coenzyme A	0	0	mitochondrion	(Watmough et al, 1989)	1.64E-09	4.97E-09
$lc12coa_{mito}$	*Beta*-hydroxyacyl-lauryl coenzyme A	0	0.0073623	mitochondrion	(Eaton et al, 1994)	1.64E-09	4.96E-09
$lc14coa_{mito}$	*Beta*-hydroxyacyl-myristoyl coenzyme A	0.0006402	0.009603	mitochondrion	(Eaton et al, 1994)	1.64E-09	4.97E-09
$lc16coa_{mito}$	*Beta*-hydroxyacyl-palmitoyl coenzyme A	0.003201	0.038412	mitochondrion	(Eaton et al, 1994)	1.64E-09	4.97E-09
$lc4coa_{mito}$	*Beta*-hydroxyacyl-butyryl coenzyme A	0	0	mitochondrion	(Watmough et al, 1989)	9.86E-08	2.74E-07
$lc5coa_{mito}$	*Beta*-hydroxyacyl-valeryl coenzyme A			mitochondrion		0.00E+00	1.77E-28
$lc6coa_{mito}$	*Beta*-hydroxyacyl-hexanoyl coenzyme A	0	0	mitochondrion	(Watmough et al, 1989)	1.65E-09	4.99E-09
$lc8coa_{mito}$	*Beta*-hydroxyacyl-octanoyl coenzyme A	0	0	mitochondrion	(Watmough et al, 1989)	1.64E-09	4.98E-09
$lmemcoa_{mito}$	L-metyl-malonyl coenzyme A			mitochondrion		2.65E-04	6.62E-04

$lvalcoa_{mito}$	*Beta*-hydroxyacyl-valproyl coenzyme A			mitochondrion		0.00E+00	1.98E-21
lac	Lactate	0.34630702	6.17736842	cell	(Krebs, 1966) (Hems & Brosnan, 1970) (Brosnan et al, 1970) (Veech et al, 1972) (Start & Newsholme, 1968) (Greenbaum et al, 1971) (Frohman et al, 1951) (Kalkhoff et al, 1966) (Parrilla, 1978) (Lagunas et al, 1970) (Rawat, 1968) (Veech et al, 1973) (Albe et al, 1990) (Guynn et al, 1986) (Williamson et al, 1967) (Kauffman et al, 1977) (Sies & Kandel, 1970) (Baquer et al, 1976) (Casazza et al, 1986) (Casazza & Veech, 1986a) (Casazza & Veech, 1986b) (Keiding, 1973) (Berman et al, 1978) (Woods & Krebs, 1973) (Williamson et al, 1969)	1.08E+00	2.36E+00
lac_{cyt}				cytosol		1.08E+00	2.36E+00
lan	Lanosterol			cell		2.44E-07	2.58E-07
lan_{er}				ER		5.89E-07	6.23E-07
$lath$	Lathosterol			cell		2.63E-07	2.78E-07
$lath_{er}$				ER		6.34E-07	6.70E-07
lpa	Lysophosphatidic acid	0.14039474	0.14039474	cell	(Das & Hajra, 1989)	5.15E-04	8.35E-04
lpa_{er}				ER		1.24E-03	2.02E-03
mag_{ld}	Monoacylglycerol			Lipid droplet		1.31E-02	2.58E-02

mal	Malate	0.18344912	4.50967105	cell	(Brosnan et al, 1970) (Veech et al, 1972) (Zakim et al, 1967) (Siess et al, 1978) (Greenbaum et al, 1971) (Frohman et al, 1951) (Kalkhoff et al, 1966) (Parrilla et al, 1975) (Lagunas et al, 1970) (Elbers et al, 1974) (Spydevold et al, 1973) (Rawat, 1968) (Veech et al, 1973) (Albe et al, 1990) (Guynn et al, 1986) (Aw et al, 1987) (Kauffman et al, 1977) (Sies & Kandel, 1970) (Baquer et al, 1976) (Casazza et al, 1986) (Casazza & Veech, 1986a) (Casazza & Veech, 1986b) (Siess et al, 1977) (Zollner, 1981) (Woods & Krebs, 1973) (Williamson et al, 1969) (Mallette et al, 1969)	1.48E-01	2.75E+00
mal_{cyt}		0.034	3.01	cytosol	(Siess et al, 1976) (Siess et al, 1978) (Greenbaum et al, 1971) (Elbers et al, 1974) (Spydevold et al, 1973) (Aw et al, 1987) (Baquer et al, 1976) (Siess et al, 1977) (Soboll et al, 1976) (Parrilla et al, 1975)	1.01E-01	1.90E+00
mal_{mito}		0.0403326	5.36672921	mitochondrion	(Siess et al, 1976) (Siess et al, 1978) (Greenbaum et al, 1971) (Elbers et al, 1974) (Spydevold et al, 1973) (Aw et al, 1987) (Baquer et al, 1976) (Siess et al, 1977) (Soboll et al, 1976) (Parrilla et al, 1975)	2.83E-01	5.12E+00
$malcoa$	Malonyl coenzyme A	0.00067857	0.01754934	cell	(McGarry et al, 1983) (Schofield et al, 1987)	5.45E-04	2.42E-03
$malcoa_{cyt}$		0.000032	0.000032	cytosol	(Nagashima et al, 2015)	5.14E-04	2.27E-03
$malcoa2_{imm}$						6.23E-04	2.93E-03

mev	Mevalonate			cell		1.70E-05	1.79E-05
mev_{cyt}				cytosol		1.70E-05	1.79E-05
$mev5p$	Mevalonate-5-phosphate			Cell		3.89E-06	4.05E-06
$mev5p_{cyt}$				cytosol		3.89E-06	4.05E-06
$mev5pp$	Mevalonate-5-pyrophosphate			Cell		1.05E-06	1.09E-06
$mev5pp_{cyt}$				cytosol		1.05E-06	1.09E-06
na	Sodium	16.4	78.6210526	cell	(Macfarlane & Spencer, 1953) (Claret & Mazet, 1972) (Williams et al, 1971b) (Williams et al, 1971a) (Berthon et al, 1980) (Claret et al, 1973)	2.85E+01	2.86E+01
na_{cyt}		20.5	34.3447964	cytosol	(Macfarlane & Spencer, 1953) (Williams et al, 1971b) (Williams et al, 1971a) (Claret-Berthon et al, 1977)	2.70E+01	2.70E+01
na_{mito}		1.3	17.3557251	mitochondrion	(Werkheiser & Bartley, 1957) (Amoore & Bartley, 1958) (Macfarlane & Spencer, 1953)	9.15E+00	9.45E+00
nad	Nicotinamide adenine dinucleotide	1.097	2.55361944	cell	(Brosnan et al, 1970) (Greenbaum et al, 1971) (Kalkhoff et al, 1966) (Lagunas et al, 1970) (Albe et al, 1990) (Sies & Kandel, 1970) (Baquer et al, 1976) (Williamson et al, 1969)	1.13E+00	1.13E+00
nad_{cyt}				cytosol		1.13E+00	1.13E+00
nad_{mito}		0.00752503	0.00915043	mitochondrion	(Birt & Bartley, 1960a) (Birt & Bartley, 1960b)	1.24E-02	2.56E-02
$nadh$	Reduced nicotinamide adenine dinucleotide	0.13257088	0.44458333	cell	(Brosnan et al, 1970) (Kalkhoff et al, 1966) (Lagunas et al, 1970) (Sies & Kandel, 1970) (Williamson et al, 1969)	7.14E-03	9.15E-03

$nadh_{cyt}$		0.00457522	0.00469562	cytosol		2.74E-03	3.23E-03
$nadh_{mito}$				mitochondrion		2.44E-02	3.76E-02
$nadp$	Nicotinamide adenine dinucleotide phosphate	0.09172456	0.85874781	cell	(Greenbaum et al, 1971) (Kalkhoff et al, 1966) (Lagunas et al, 1970) (Albe et al, 1990) (Sies & Kandel, 1970) (Baquer et al, 1976) (Williamson et al, 1969)	1.12E-01	2.38E-01
$nadp_{cyt}$				cytosol		1.12E-01	2.38E-01
$nadp_{mito}$		0.00198661	1.78	mitochondrion	(Birt & Bartley, 1960a) (Birt & Bartley, 1960b) (Giulivi, 1998)	1.52E-03	3.24E-03
$nadph$	Reduced nicotinamide adenine dinucleotide phosphate			cell		1.10E+00	1.22E+00
$nadph_{cyt}$				cytosol		7.62E-01	8.88E-01
$nadph_{mito}$				mitochondrion		2.00E+00	2.00E+00
$nh3$	Ammonia	0.40480482	2.22291667	cell	(Brosnan et al, 1970) (Spydevold et al, 1973) (Rawat, 1968) (Veech et al, 1973) (Albe et al, 1990) (Guynn et al, 1986) (Williamson et al, 1967) (Baquer et al, 1976) (Parrilla et al, 1975) (Saheki et al, 1978) (Woods & Krebs, 1973)	1.35E-01	1.76E-01
$nh3_{cyt}$				cytosol		1.26E-01	1.64E-01
$nh3_{mito}$		0.014	0.035	mitochondrion	(Groen et al, 1982)	5.55E-02	7.41E-02
$o2_{cyt}$ $[mmHg]$	Oxygen			cytosol		1.37E+02	1.61E+02

oaa	Oxaloacetate	0.00920027	0.07513516	cell	(Siess et al, 1978) (Eschenbrenner & Guynn, 1976) (Spydevold et al, 1973) (Frohman et al, 1951) (Parrilla et al, 1975) (Parrilla, 1978) (Lagunas et al, 1970) (Rawat, 1968) (Albe et al, 1990) (Siess et al, 1977) (Williamson et al, 1969)	3.70E-04	7.45E-03
oaa_{cyt}		0.00289772	0.035/50.6	cytosol	(Siess et al, 1976) (Siess et al, 1978) (Spydevold et al, 1973) (Baquer et al, 1976) (Siess et al, 1977) (Parrilla et al, 1975)	3.68E-04	7.44E-03
oaa_{mito}		0.00008	0.0623	mitochondrion	(Siess et al, 1976) (Siess et al, 1978) (Spydevold et al, 1973) (Baquer et al, 1976) (Siess et al, 1977) (Parrilla et al, 1975)	1.18E-05	6.61E-05
orn	Ornithine	0.41182456	6.84452421	cell	(Saheki et al, 1977) (Saheki & Katunuma, 1975) (Saheki et al, 1975) (Saheki et al, 1978)	2.67E+00	2.68E+00
orn_{cyt}				cytosol		2.52E+00	2.53E+00
orn_{mito}				mitochondrion		8.88E-01	9.04E-01
p	Phosphate	4.7	14.7882456	cell	(Hems & Brosnan, 1970) (Veech et al, 1972) (Veech et al, 1970) (Greenbaum et al, 1971) (Veech et al, 1973) (Albe et al, 1990) (Guynn et al, 1986) (Aw et al, 1987) (Casazza & Veech, 1986a) (Keppler & Decker, 1969) (Sestoft, 1974) (Guynn et al, 1974) (Woods & Krebs, 1973)	1.28E+01	1.61E+01
p_{cyt}		2.8	10	cytosol	(Aw et al, 1987) (Soboll et al, 1978)	8.17E+00	1.02E+01
p_{er}				ER		8.18E+00	1.02E+01
p_{mito}		0.9	109/12.6	mitochondrion	(Aw et al, 1987) (Soboll et al, 1978) (Werkheiser & Bartley, 1957) (Amoore & Bartley, 1958)	7.51E+00	1.02E+01

pa	Phosphatidate	0.29959742	1.01114131	cell	(Bocckino et al, 1987)	2.32E-02	3.15E-02
pa_{er}				ER		5.60E-02	7.59E-02
pep	Phosphoenolpyruvate	0.04913816	0.74409211	cell	(Hems & Brosnan, 1970) (Veech et al, 1972) (Greenbaum et al, 1971) (Kalkhoff et al, 1966) (Parrilla et al, 1975) (Parrilla, 1978) (Lagunas et al, 1970) (Spydevold et al, 1973) (Veech et al, 1973) (Albe et al, 1990) (Guynn et al, 1986) (Baquer et al, 1976) (Woods & Krebs, 1973) (Williamson et al, 1969) (Mallette et al, 1969)	2.39E-02	2.27E-01
pep_{cyt}		0.31	0.31	cytosol	(Siess et al, 1977)	2.39E-02	2.27E-01
pep_{mito}		2.05	2.05	mitochondrion	(Siess et al, 1977)	1.24E-04	1.27E-03
$pg2$	2-Phosphoglycerate	0.02456908	0.19655263	cell	(Hems & Brosnan, 1970) (Greenbaum et al, 1971) (Parrilla, 1978) (Lagunas et al, 1970) (Veech et al, 1973) (Albe et al, 1990) (Baquer et al, 1976) (Woods & Krebs, 1973) (Williamson et al, 1969) (Mallette et al, 1969)	1.40E-02	1.34E-01
$pg2_{cyt}$				cytosol		1.40E-02	1.34E-01
$pg3$	3-Phosphoglycerate	0.20123246	0.84002851	cell	(Hems & Brosnan, 1970) (Veech et al, 1972) (Veech et al, 1970) (Greenbaum et al, 1971) (Kalkhoff et al, 1966) (Parrilla et al, 1975) (Parrilla, 1978) (Lagunas et al, 1970) (Veech et al, 1973) (Albe et al, 1990) (Guynn et al, 1986) (Baquer et al, 1976) (Siess et al, 1977) (Woods & Krebs, 1973)	8.11E-02	7.75E-01
$pg3_{cyt}$				cytosol		8.11E-02	7.75E-01

$pg6$	6-Phosphogluconate	0.01099759	0.05264803	cell	(Greenbaum et al, 1971) (Lagunas et al, 1970) (Albe et al, 1990) (Kauffman et al, 1977) (Baquer et al, 1976) (Casazza et al, 1986) (Casazza & Veech, 1986a) (Casazza & Veech, 1986b)	6.16E-02	6.62E-02
$pg6_{cyt}$				cytosol		6.16E-02	6.62E-02
$pgl6$	6-Phosphogluconolactone			cell		9.39E-03	2.18E-02
$pgl6_{cyt}$				cytosol		9.39E-03	2.18E-02
pp	Pyrophosphate	0.014	0.022	cell	(Albe et al, 1990)	1.94E-03	2.16E-03
pp_{cyt}		0.0053818	0.00889167	cytosol	(Guynn et al, 1974)	1.94E-03	2.16E-03
$propcoa_{mito}$	Propionyl coenzyme A			mitochondrion		0.00E+00	2.03E-24
pyr	Pyruvate	0.02339912	0.76789691	cell	(Krebs, 1966) (Hems & Brosnan, 1970) (Brosnan et al, 1970) (Veech et al, 1972) (Zakim et al, 1967) (Veech et al, 1970) (Start & Newsholme, 1968) (Greenbaum et al, 1971) (Frohman et al, 1951) (Kalkhoff et al, 1966) (Parrilla et al, 1975) (Parrilla, 1978) (Lagunas et al, 1970) (Rawat, 1968) (Veech et al, 1973) (Albe et al, 1990) (Aw et al, 1987) (Williamson et al, 1967) (Kauffman et al, 1977) (Sies & Kandel, 1970) (Baquer et al, 1976) (Casazza et al, 1986) (Casazza & Veech, 1986a) (Casazza & Veech, 1986b) (Siess et al, 1977) (Keiding, 1973) (Woods & Krebs, 1973) (Williamson et al, 1969)	6.44E-02	1.49E-01
pyr_{cyt}		0.32	0.64	cytosol	(Aw et al, 1987) (Siess et al, 1977)	4.40E-02	1.03E-01

pyr_{mito}		1.08	1.6	mitochondrion	(Siess et al, 1978) (Aw et al, 1987) (Siess et al, 1977)	1.23E-01	2.76E-01
q_{mm}	Ubiquinone			Membrane		8.14E-02	8.71E-02
$qh2_{mm}$	Ubiquinol			Membrane		1.29E-02	1.86E-02
$r5p$	Ribose 5-phosphate	0.00350987	0.01193355	cell	(Casazza & Veech, 1986a) (Casazza & Veech, 1986b)	1.16E-02	2.02E-02
$r5p_{cyt}$				cytosol		1.16E-02	2.02E-02
$ru5p$	Ribulose 5-phosphate	0.0079557	0.04890417	cell	(Kauffman et al, 1977) (Casazza et al, 1986) (Casazza & Veech, 1986c) (Casazza & Veech, 1986a) (Casazza & Veech, 1986b)	3.73E-03	6.47E-03
$ru5p_{cyt}$				cytosol		3.73E-03	6.47E-03
$s7p$	Sedoheptulose 7-phosphate	0.05966776	0.1041261	cell	(Greenbaum et al, 1971) (Baquer et al, 1976) (Casazza & Veech, 1986a)	9.18E-02	1.24E-01
$s7p_{cyt}$				cytosol		9.18E-02	1.24E-01
ser_{cyt}	serine			cytosol		2.62E-01	3.54E-01
sqe	Squalene-epoxide			Cell		4.34E-07	4.61E-07
sqe_{cyt}				cytosol		4.34E-07	4.61E-07
squ	Squalene			Cell		4.91E-09	5.21E-09
squ_{cyt}				cytosol		4.91E-09	5.21E-09
suc	Succinate	0.19887095	1.068	cell	(Frohman et al, 1951) (Albe et al, 1990) (Williamson et al, 1969)	4.03E-02	1.30E-01
suc_{mito}				mitochondrion		2.42E-01	7.77E-01
$succoa$	Succinyl coenzyme A	0.0212932	0.06270965	cell	(Krahenbuhl & Brass, 1991)	1.02E-03	2.54E-03
$succoa_{mito}$		0.1	0.16	mitochondrion	(Matsuishi et al, 1991)	6.09E-03	1.52E-02
tag	Triacylglycerol	1.005114	119.422862	cell	(Fushimi et al, 2006) (Tijburg et al, 1991) (Hariharan et al, 1996) (Purushothama et al, 1995)	1.31E+01	1.45E+01
tag_{er}				ER		3.42E-02	8.43E-02
tag_{LD}				Lipid droplet		1.31E+01	1.45E+01
tag_{VLDL}		0.36	0.36	VLDL	(Tijburg et al, 1991)	1.01E-04	2.36E-04

$tmas$				Cell		1.94E-07	2.09E-07
$tmas_{er}$				ER		4.68E-07	5.04E-07
udp	Uridine diphosphate	0.14039474	0.49138158	cell	(Jackson et al, 1980) (Hitchings, 1974) (Keppler et al, 1974) (Jackson et al, 1976)	1.86E-01	5.24E-01
udp_{cyt}				cytosol		1.86E-01	5.24E-01
$udpgal$	Uridine diphosphate galactose	0.04679825	0.21059211	cell	(Keiding, 1973) (Keppler et al, 1970) (Berman et al, 1978)	1.83E-01	2.78E-01
$udpgal_{cyt}$				cytosol		1.83E-01	2.78E-01
$udpglc$	Uridine diphosphate glucose	0.33	1.0295614	cell	(Albe et al, 1990) (Keppler & Decker, 1969) (Keppler et al, 1969) (Jackson et al, 1980) (Jackson et al, 1976) (Keiding, 1973) (Keppler et al, 1970) (Guynn et al, 1974)	5.69E-01	8.64E-01
$udpglc_{cyt}$				cytosol		5.69E-01	8.64E-01
$urea$	Urea	4.91381579	58.9657895	cell	(Saheki et al, 1977) (Saheki et al, 1975) (Saheki et al, 1978)	1.94E+01	3.05E+01
$urea_{cyt}$				cytosol		1.94E+01	3.05E+01
utp	Uridine triphosphate	0.60837719	1.15591667	cell	(Jackson et al, 1980) (Hitchings, 1974) (Keppler et al, 1974) (Jackson et al, 1976) (Weber et al, 1985) (Guynn et al, 1974)	8.19E-01	1.05E+00
utp_{cyt}				cytosol		8.19E-01	1.05E+00
val_{cyt}	Valproate			cytosol		0.00E+00	0.00E+00
$valcar_{cyt}$	Carnitylvalproate			cytosol		0.00E+00	3.09E-21
$valcar_{mito}$				mitochondrion		0.00E+00	2.46E-20
$valcoa_{cyt}$	Valproyl coenzym A			cytosol		0.00E+00	0.00E+00
$valcoa_{mito}$				mitochondrion		0.00E+00	2.89E-20

$v_{mm}\ [mV]$	mitochondrial membrane potential					-1.33E+02	-1.30E+02
$x5p$	Xylulose 5-phosphate	0.00889167	0.07651513	cell	(Kauffman et al, 1977) (Fushimi et al, 2001) (Baquer et al, 1976) (Casazza et al, 1986) (Casazza & Veech, 1986c) (Casazza & Veech, 1986a) (Casazza & Veech, 1986b) (Woods & Krebs, 1973)	5.50E-03	9.51E-03
$x5p_{cyt}$				cytosol		5.50E-03	9.51E-03
xyl	Xylulose			cell		1.21E-06	2.42E-06
xyl_{cyt}				cytosol		1.21E-06	2.42E-06
zym	Zymosterol			Cell		7.43E-08	8.04E-08
zym_{er}				ER		1.79E-07	1.94E-07
$zymostenol$	Zymostenol			Cell		3.38E-07	3.58E-07
$zymostenol_{er}$				ER		8.16E-07	8.64E-07

Factors used for the conversion of experimental data from various sources

conversion	values	reference
Dry weight / wet weight	3.23 - 4.94	(Albe et al, 1990) (Parrilla et al, 1975) (Tischler et al, 1977)
Vol_mito	0.2 ml/g_dw	(Tischler et al, 1977)
Vol_cyt	2 ml/g_dw	(Tischler et al, 1977)
Vol_cyt	0.456 ml_cytosol/ml_ww	(Bolender & Weibel, 1973)
Vol_mito	0.237 ml_cytosol/ml_ww	(Bolender & Weibel, 1973)
Vol_er	0.189 ml_cytosol/ml_ww	(Bolender & Weibel, 1973)
Dichte	1.067 g/ml	(Bolender & Weibel, 1973)
Cellular protein density	313 mg/ml	(Albe et al, 1990)
Protein content / cell	1.8 mg/10^6	(Berthon et al, 1980)
Protein content / cell	1.2 -2.4 µg/10^3	(Uhal & Roehrig,

		1982)
Mitochondrial protein / g_ww	60 -90 mg/g_ww	(Brass & Ruff, 1992) (Forestier et al, 1997)
Extramitochondrial protein / g_ww	130 mg/ g_ww	(Brass & Ruff, 1992)

References

Albe KR, Butler MH, Wright BE (1990) Cellular concentrations of enzymes and their substrates. *J Theor Biol* **143:** 163-195

Amoore JE, Bartley W (1958) The permeability of isolated rat-liver mitochondria to sucrose, sodium chloride and potassium chloride at 0 degrees. *Biochem J* **69:** 223-236

Andersen JM, Turley SD, Dietschy JM (1979) Low and high density lipoproteins and chylomicrons as regulators of rate of cholesterol synthesis in rat liver in vivo. *Proc Natl Acad Sci U S A* **76:** 165-169

Avoy DR, Swyryd EA, Gould RG (1965) Effects of Alpha-P-Chlorophenoxyisobutyryl Ethyl Ester (Cpib) with and without Androsterone on Cholesterol Biosynthesis in Rat Liver. *J Lipid Res* **6:** 369-376

Aw TY, Andersson BS, Jones DP (1987) Mitochondrial transmembrane ion distribution during anoxia. *Am J Physiol* **252:** C356-361

Baquer NZ, Cascales M, McLean P, Greenbaum AL (1976) Effects of thyroid hormone deficiency on the distribution of hepatic metabolites and control of pathways of carbohydrate metabolism in liver and adipose tissue of the rat. *Eur J Biochem* **68:** 403-413

Bartlett K, Eaton S (2004) Mitochondrial beta-oxidation. *European Journal of Biochemistry* **271:** 462-469

Bellamy D (1962) The endogenous citric acid-cycle intermediates and amino acids of mitochondria. *Biochem J* **82:** 218-224

Berman WF, Rogers SR, Bautista JO, Segal S (1978) Galactose and glucose metabolism in the isolated perfused suckling and adult rat liver. *Metabolism* **27:** 1721-1731

Berthon B, Claret M, Mazet JL, Poggioli J (1980) Volume- and temperature-dependent permeabilities in isolated rat liver cells. *J Physiol* **305:** 267-277

Birt LM, Bartley W (1960a) The behaviour of pyridine nucleotides of mitochondria in a 'saline medium'. *Biochem J* **76:** 328-341

Birt LM, Bartley W (1960b) The pyridine nucleotide metabolism of mitochondria incubated with and without added substrates and metabolic inhibitors. *Biochem J* **76:** 427-438

Bocckino SB, Blackmore PF, Wilson PB, Exton JH (1987) Phosphatidate accumulation in hormone-treated hepatocytes via a phospholipase D mechanism. *J Biol Chem* **262:** 15309-15315

Bolender RP, Weibel ER (1973) A morphometric study of the removal of phenobarbital-induced membranes from hepatocytes after cessation of threatment. *J Cell Biol* **56:** 746-761

Brass EP, Ruff LJ (1992) Rat hepatic coenzyme A is redistributed in response to mitochondrial acyl-coenzyme A accumulation. *J Nutr* **122:** 2094-2100

Brosnan JT, Krebs HA, Williamson DH (1970) Effects of ischaemia on metabolite concentrations in rat liver. *Biochem J* **117:** 91-96

Cartwright IJ, Higgins JA (1992) Quantification of apolipoprotein B-48 and B-100 in rat liver endoplasmic reticulum and Golgi fractions. *Biochem J* **285 (Pt 1):** 153-159

Casazza JP, Schaffer WT, Veech RL (1986) The effect of dehydroepiandrosterone on liver metabolites. *J Nutr* **116:** 304-310

Casazza JP, Veech RL (1986a) The content of pentose-cycle intermediates in liver in starved, fed ad libitum and meal-fed rats. *Biochem J* **236:** 635-641

Casazza JP, Veech RL (1986b) The interdependence of glycolytic and pentose cycle intermediates in ad libitum fed rats. *J Biol Chem* **261:** 690-698

Casazza JP, Veech RL (1986c) The Measurement of Xylulose 5-Phosphate, Ribulose 5-Phosphate, and Combined Sedoheptulose 7-Phosphate and Ribose 5-Phosphate in Liver-Tissue. *Analytical Biochemistry* **159:** 243-248

Chen MT, Kaufman LN, Spennetta T, Shrago E (1992) Effects of high fat-feeding to rats on the interrelationship of body weight, plasma insulin, and fatty acyl-coenzyme A esters in liver and skeletal muscle. *Metabolism* **41:** 564-569

Claret-Berthon B, Claret M, Mazet JL (1977) Fluxes and distribution of calcium in rat liver cells: kinetic analysis and identification of pools. *J Physiol* **272:** 529-552

Claret B, Claret M, Mazet JL (1973) Ionic transport and membrane potential of rat liver cells in normal and low-chloride solutions. *J Physiol* **230:** 87-101

Claret M, Mazet JL (1972) Ionic fluxes and permeabilities of cell membranes in rat liver. *J Physiol* **223:** 279-295

Das AK, Hajra AK (1989) Quantification, Characterization and Fatty-Acid Composition of Lysophosphatidic Acid in Different Rat-Tissues. *Lipids* **24:** 329-333

Eaton S, Turnbull DM, Bartlett K (1994) Redox control of beta-oxidation in rat liver mitochondria. *Eur J Biochem* **220:** 671-681

Elbers R, Heldt HW, Schmucker P, Soboll S, Wiese H (1974) Measurement of the ATP/ADP ratio in mitochondria and in the extramitochondrial compartment by fractionation of freeze-stopped liver tissue in non-aqueous media. *Hoppe Seylers Z Physiol Chem* **355:** 378-393

Ellis BA, Poynten A, Lowy AJ, Furler SM, Chisholm DJ, Kraegen EW, Cooney GJ (2000) Long-chain acyl-CoA esters as indicators of lipid metabolism and insulin sensitivity in rat and human muscle. *Am J Physiol Endocrinol Metab* **279:** E554-560

Endo A (1980) Monacolin K, a new hypocholesterolemic agent that specifically inhibits 3-hydroxy-3-methylglutaryl coenzyme A reductase. *J Antibiot (Tokyo)* **33:** 334-336

Eschenbrenner E, Guynn RW (1976) Measurement of oxaloacetate in tissue extracts by enzymatic cycling. *Anal Biochem* **72:** 220-229

Forestier M, Solioz M, Isbeki F, Talos C, Reichen J, Krahenbuhl S (1997) Hepatic mitochondrial proliferation in rats with secondary biliary cirrhosis: time course and mechanisms. *Hepatology* **26:** 386-391

Frohman CE, Orten JM, Smith AH (1951) Levels of acids of the citric acid cycle in tissues of normal and diabetic rats. *J Biol Chem* **193:** 803-807

Fushimi T, Suruga K, Oshima Y, Fukiharu M, Tsukamoto Y, Goda T (2006) Dietary acetic acid reduces serum cholesterol and triacylglycerols in rats fed a cholesterol-rich diet. *Br J Nutr* **95:** 916-924

Fushimi T, Tayama K, Fukaya M, Kitakoshi K, Nakai N, Tsukamoto Y, Sato Y (2001) Acetic acid feeding enhances glycogen repletion in liver and skeletal muscle of rats. *J Nutr* **131:** 1973-1977

Gardner LB, Liu Z, Barrett EJ (1993) The role of glucose-6-phosphatase in the action of insulin on hepatic glucose production in the rat. *Diabetes* **42:** 1614-1620

Garland PB, Shepherd D, Yates DW (1965) Steady-state concentrations of coenzyme A, acetyl-coenzyme A and long-chain fatty acyl-coenzyme A in rat-liver mitochondria oxidizing palmitate. *Biochem J* **97:** 587-594

Giulivi C (1998) Functional implications of nitric oxide produced by mitochondria in mitochondrial metabolism. *Biochem J* **332 (Pt 3):** 673-679

Gordon EE (1961) The metabolism of citrate-C-14 in normal and in fluoroinhibitor-poisoned rats. *J Clin Invest* **40:** 1719-1726

Greenbaum AL, Gumaa KA, McLean P (1971) The distribution of hepatic metabolites and the control of the pathways of carbohydrate metabolism in animals of different dietary and hormonal status. *Arch Biochem Biophys* **143:** 617-663

Groen AK, Sips HJ, Vervoorn RC, Tager JM (1982) Intracellular compartmentation and control of alanine metabolism in rat liver parenchymal cells. *Eur J Biochem* **122:** 87-93

Guynn RW, Merrill DK, Lund K (1986) The reactions of the phosphorylated pathway of L-serine biosynthesis: thermodynamic relationships in rat liver in vivo. *Arch Biochem Biophys* **245:** 204-211

Guynn RW, Veech RL (1974) Direct enzymic determination of acetate in tissue extracts in the presence of labile acetate esters. *Anal Biochem* **61:** 6-15

Guynn RW, Veloso D, Lawson JW, Veech RL (1974) The concentration and control of cytoplasmic free inorganic pyrophosphate in rat liver in vivo. *Biochem J* **140:** 369-375

Hariharan K, Purushothama S, Murthy KN, Raina PL (1992) Effect of Long-Term Feeding of Palm Oil on Growth and Lipid-Metabolism in Rats. *Nutrition Research* **12:** 867-877

Hariharan K, Purushothama S, Raina PL (1996) Studies on red palm oil: Effect of partial supplementation of saturated fats upon lipids and lipoproteins. *Nutrition Research* **16:** 1381-1392

Harris EJ, van Dam K (1968) Changes of total water and sucrose space accompanying induced ion uptake or phosphate swelling of rat liver mitochondria. *Biochem J* **106:** 759-766

Hems DA, Brosnan JT (1970) Effects of ischaemia on content of metabolites in rat liver and kidney in vivo. *Biochem J* **120:** 105-111

Hitchings GH (1974) Indications for control mechanisms in purine and pyrimidine biosynthesis as revealed by studies with inhibitors. *Adv Enzyme Regul* **12:** 121-129

Hornbrook KR, Burch HB, Lowry OH (1966) The effects of adrenalectomy and hydrocortisone on rat liver metabolites and glycogen synthetase activity. *Mol Pharmacol* **2:** 106-116

Hue L, Blackmore PF, Exton JH (1981) Fructose 2,6-bisphosphate. Hormonal regulation and mechanism of its formation in liver. *J Biol Chem* **256:** 8900-8903

Hue L, Blackmore PF, Shikama H, Robinson-Steiner A, Exton JH (1982) Regulation of fructose-2,6-bisphosphate content in rat hepatocytes, perfused hearts, and perfused hindlimbs. *J Biol Chem* **257:** 4308-4313

Ide T, Okamatsu H, Sugano M (1978) Regulation by dietary fats of 3-hydroxy-3-methylglutaryl-Coenzyme A reductase in rat liver. *J Nutr* **108:** 601-612

Indiveri C, Tonazzi A, Palmieri F (1994) The reconstituted carnitine carrier from rat liver mitochondria: evidence for a transport mechanism different from that of the other mitochondrial translocators. *Biochim Biophys Acta* **1189:** 65-73

Jackson RC, Boritzki TJ, Morris HP, Weber G (1976) Purine and pyrimidine ribonucleotide contents of rat liver and hepatoma 3924A and the effect of ischemia. *Life Sci* **19:** 1531-1536

Jackson RC, Lui MS, Boritzki TJ, Morris HP, Weber G (1980) Purine and pyrimidine nucleotide patterns of normal, differentiating, and regenerating liver and of hepatomas in rats. *Cancer Res* **40:** 1286-1291

Kalkhoff RK, Hornbrook KR, Burch HB, Kipnis DM (1966) Studies of the metabolic effects of acute insulin deficiency. II. Changes in hepatic glycolytic and krebs-cycle intermediates and pyridine nucleotides. *Diabetes* **15:** 451-456

Kauffman FC, Evans RK, Thurman RG (1977) Alterations in nicotinamide and adenine nucleotide systems during mixed-function oxidation of p-nitroanisole in perfused livers from normal and phenobarbital-treated rats. *Biochem J* **166:** 583-592

Keiding S (1973) Galactose elimination capacity in the rat. *Scand J Clin Lab Invest* **31:** 319-325

Keller RK (1996) Squalene synthase inhibition alters metabolism of nonsterols in rat liver. *Biochimica Et Biophysica Acta-Lipids and Lipid Metabolism* **1303:** 169-179

Keppler D, Decker K (1969) Studies on the mechanism of galactosamine-1-phosphate and its inhibition of UDP-glucose pyrophosphorylase. *Eur J Biochem* **10:** 219-225

Keppler D, Frohlich J, Reutter W, Wieland O, Decker K (1969) Changes in uridine nucleotides during liver perfusion with D-galactosamine. *FEBS Lett* **4:** 278-280

Keppler D, Rudigier J, Decker K (1970) Enzymic determination of uracil nucleotides in tissues. *Anal Biochem* **38:** 105-114

Keppler DO, Pausch J, Decker K (1974) Selective uridine triphosphate deficiency induced by D-galactosamine in liver and reversed by pyrimidine nucleotide precursors. Effect on ribonucleic acid synthesis. *The Journal of biological chemistry* **249:** 211-216

Knowles SE, Jarrett IG, Filsell OH, Ballard FJ (1974) Production and utilization of acetate in mammals. *Biochem J* **142:** 401-411

Kondrup J, Grunnet N (1973) The effect of acute and prolonged ethanol treatment on the contents of coenzyme A, carnitine and their derivatives in rat liver. *Biochem J* **132:** 373-379

Krahenbuhl S, Brass EP (1991) Fuel homeostasis and carnitine metabolism in rats with secondary biliary cirrhosis. *Hepatology* **14:** 927-934

Krahenbuhl S, Chang M, Brass EP, Hoppel CL (1991) Decreased activities of ubiquinol:ferricytochrome c oxidoreductase (complex III) and ferrocytochrome c:oxygen oxidoreductase (complex IV) in liver mitochondria from rats with hydroxycobalamin[c-lactam]-induced methylmalonic aciduria. *J Biol Chem* **266:** 20998-21003

Krebs HA (1966) The regulation of the release of ketone bodies by the liver. *Adv Enzyme Regul* **4:** 339-354

Lagunas R, McLean P, Greenbaum AL (1970) The effect of raising the NAD+ content on the pathways of carbohydrate metabolism and lipogenesis in rat liver. *Eur J Biochem* **15:** 179-190

Latipaa PM, Karki TT, Hiltunen JK, Hassinen IE (1986) Regulation of palmitoylcarnitine oxidation in isolated rat liver mitochondria. Role of the redox state of NAD(H). *Biochim Biophys Acta* **875:** 293-300

Lopes-Cardozo M, van den Bergh SG (1974a) Ketogenesis in isolated rat liver mitochondria. II. Factors affecting the rate of beta-oxidation. *Biochim Biophys Acta* **357:** 43-52

Lopes-Cardozo M, van den Bergh SG (1974b) Ketogenesis in isolated rat liver mitochondria. III. Relationship with the rate of beta-oxidation. *Biochim Biophys Acta* **357:** 53-62

Macfarlane MG, Spencer AG (1953) Changes in the water, sodium and potassium content of rat-liver mitochondria during metabolism. *Biochem J* **54:** 569-575

Mallette LE, Exton JH, Park (1969) Effects of glucagon on amino acid transport and utilization in the perfused rat liver. *J Biol Chem* **244:** 5724-5728

Matsuishi T, Stumpf DA, Chrislip K (1991) The effect of malate on propionate mitochondrial toxicity. *Biochem Med Metab Biol* **46:** 177-184

McGarry JD, Mills SE, Long CS, Foster DW (1983) Observations on the affinity for carnitine, and malonyl-CoA sensitivity, of carnitine palmitoyltransferase I in animal and human tissues. Demonstration of the presence of malonyl-CoA in non-hepatic tissues of the rat. *Biochem J* **214:** 21-28

Menahan LA, Hron WT, Hinkelman DG, Miziorko HM (1981) Interrelationships between 3-hydroxy-3-methylglutaryl-CoA synthase, acetoacetyl-CoA and ketogenesis. *Eur J Biochem* **119:** 287-294

Murthy VK, Steiner G (1973) Hepatic acetate levels in relation to altered lipid metabolism. *Metabolism* **22:** 81-84

Nagashima S, Yagyu H, Tozawa R, Tazoe F, Takahashi M, Kitamine T, Yamamuro D, Sakai K, Sekiya M, Okazaki H, Osuga J, Honda A, Ishibashi S (2015) Plasma cholesterol-lowering and transient liver dysfunction in mice lacking squalene synthase in the liver. *Journal of Lipid Research* **56:** 998-1005

Neely P, El-Maghrabi MR, Pilkis SJ, Claus TH (1981) Effect of diabetes, insulin, starvation, and refeeding on the level of rat hepatic fructose 2,6-bisphosphate. *Diabetes* **30:** 1062-1064

Nishimura M, Fedorov S, Uyeda K (1994) Glucose-stimulated synthesis of fructose 2,6-bisphosphate in rat liver. Dephosphorylation of fructose 6-phosphate, 2-kinase:fructose 2,6-bisphosphatase and activation by a sugar phosphate. *J Biol Chem* **269:** 26100-26106

O KM, Choy PC (1993) Effects of Fasting on Phosphatidylcholine Biosynthesis in Hamster Liver - Regulation of Cholinephosphotransferase Activity by Endogenous Argininosuccinate. *Biochemical Journal* **289:** 727-733

Ohta J, Ubuka T, Kodama H, Sugahara K, Yao K, Masuoka N, Kinuta M (1995) Increase in cystathionine content in rat liver mitochondria after D,L-propargylglycine administration. *Amino Acids* **9:** 111-122

Parmeggiani A, Bowman RH (1963) Regulation of Phosphofructokinase Activity by Citrate in Normal and Diabetic Muscle. *Biochem Biophys Res Commun* **12:** 268-273

Parrilla R (1978) The effect of starvation in the rat on metabolite concentrations in blood, liver and skeletal muscle. *Pflugers Arch* **374:** 9-14

Parrilla R, Jimenez I, Ayuso-Parrilla MS (1975) Glucagon and insulin control of gluconeogenesis in the perfused isolated rat liver. Effects on cellular metabolite distribution. *Eur J Biochem* **56:** 375-383

Pausch J, Rasenack J, Haussinger D, Gerok W (1985) Hepatic carbamoyl phosphate metabolism. Role of cytosolic and mitochondrial carbamoyl phosphate in de novo pyrimidine synthesis. *Eur J Biochem* **150:** 189-194

Potter VR, Busch H (1950) Citric acid content of normal and tumor tissues in vivo following injection of fluoroacetate. *Cancer Res* **10:** 353-356

Purushothama S, Narasimhamurthy K, Raina PL, Hariharan K (1994) A Study of Plasma and Liver Lipid Profile of Rats Fed Palm Oil or Safflower Oil Along with Cholesterol. *Nutrition Research* **14:** 255-269

Purushothama S, Raina PL, Hariharan K (1995) Effect of Long-Term Feeding of Rice Bran Oil Upon Lipids and Lipoproteins in Rats. *Molecular and Cellular Biochemistry* **146:** 63-69

Rawat AK (1968) Effects of ethanol infusion on the redox state and metabolite levels in rat liver in vivo. *Eur J Biochem* **6:** 585-592

Richards RG, Mendenhall CL, MacGee J (1975) A simple, rapid method for measurement of acetate in tissue and serum. *J Lipid Res* **16:** 395-397

Saheki T, Katsunuma T, Sase M (1977) Regulation of urea synthesis in rat liver. Changes of ornithine and acetylglutamate concentrations in the livers of rats subjected to dietary transitions. *J Biochem* **82:** 551-558

Saheki T, Katunuma N (1975) Analysis of regulatory factors for urea synthesis by isolated perfused rat liver. I. Urea synthesis with ammonia and glutamine as nitrogen sources. *J Biochem* **77:** 659-669

Saheki T, Ohkubo T, Katsunuma T (1978) Regulation of urea synthesis in rat liver. Increase in the concentrations of ornithine and acetylglutamate in rat liver in response to urea synthesis stimulated by the injection of an ammonium salt. *J Biochem* **84:** 1423-1430

Saheki T, Tsuda M, Tanaka T, katunuma N (1975) Analysis of regulatory factors for urea synthesis by isolated perfused rat liver. II. Comparison of urea synthesis in livers of rats subjected to different dietary conditions. *J Biochem* **77:** 671-678

Schofield PS, Sugden MC, Corstorphine CG, Zammit VA (1987) Altered interactions between lipogenesis and fatty acid oxidation in regenerating rat liver. *Biochem J* **241:** 469-474

Sestoft L (1974) Regulation of fructose metabolism in the perfused rat liver. Interrelation with inorganic phosphate, glucose, ketone body and ethanol metabolism. *Biochim Biophys Acta* **343:** 1-16

Shigesada K, Tatibana M (1971) Role of acetylglutamate in ureotelism. I. Occurrence and biosynthesis of acetylglutamate in mouse and rat tissues. *J Biol Chem* **246:** 5588-5595

Sies H, Kandel M (1970) Positive increase of redox potential of the extramitochondrial NADP(H) system by mixed function oxidations in hemoglobin-free perfused rat liver. *FEBS Lett* **9:** 205-208

Siess EA (1985) Stimulation by 3-hydroxybutyrate of pyruvate carboxylation in mitochondria from rat liver. *Eur J Biochem* **152:** 131-136

Siess EA, Brocks DG, Lattke HK, Wieland OH (1977) Effect of glucagon on metabolite compartmentation in isolated rat liver cells during gluconeogenesis from lactate. *Biochem J* **166:** 225-235

Siess EA, Brocks DG, Wieland OH (1976) Subcellular distribution of key metabolites in isolated liver cells from fasted rats. *FEBS Lett* **69:** 265-271

Siess EA, Brocks DG, Wieland OH (1978) Distribution of metabolites between the cytosolic and mitochondrial compartments of hepatocytes isolated from fed rats. *Hoppe Seylers Z Physiol Chem* **359:** 785-798

Soboll S, Scholz R, Freisl M, Elbers R, Heldt HW (1976) *Use of Isolated Liver Cells and Kidney Tubules in Metabolic Studies*, Amsterdam, Oxford and New York: North-Holland/American Elsevier.

Soboll S, Scholz R, Heldt HW (1978) Subcellular metabolite concentrations. Dependence of mitochondrial and cytosolic ATP systems on the metabolic state of perfused rat liver. *Eur J Biochem* **87:** 377-390

Spencer AF, Lowenstein JM (1967) Citrate content of liver and kidney of rat in various metabolic states and in fluoroacetate poisoning. *Biochem J* **103:** 342-348

Spydevold OS, Greenbaum AL, McLean P (1973) Effect of quinolinic acid on the distribution of hepatic metabolites. *Biochem Biophys Res Commun* **54:** 1581-1587

Start C, Newsholme EA (1968) The effects of starvation and alloxan-diabetes on the contents of citrate and other metabolic intermediates in rat liver. *Biochem J* **107:** 411-415

Tijburg LB, Nyathi CB, Meijer GW, Geelen MJ (1991) Biosynthesis and secretion of triacylglycerol in rat liver after partial hepatectomy. *Biochem J* **277 (Pt 3):** 723-728

Tischler ME, Hecht P, Williamson JR (1977) Determination of mitochondrial/cytosolic metabolite gradients in isolated rat liver cells by cell disruption. *Arch Biochem Biophys* **181:** 278-293

Uhal BD, Roehrig KL (1982) Effect of dietary state on hepatocyte size. *Biosci Rep* **2:** 1003-1007

Veech RL, Guynn R, Veloso D (1972) The time-course of the effects of ethanol on the redox and phosphorylation states of rat liver. *Biochem J* **127:** 387-397

Veech RL, Raijman L, Dalziel K, Krebs HA (1969) Disequilibrium in the triose phosphate isomerase system in rat liver. *Biochem J* **115:** 837-842

Veech RL, Raijman L, Krebs HA (1970) Equilibrium relations between the cytoplasmic adenine nucleotide system and nicotinamide-adenine nucleotide system in rat liver. *Biochem J* **117:** 499-503

Veech RL, Veloso D, Mehlman MA (1973) Thiamin deficiency: liver metabolite levels and redox and phosphorylation states in thiamin-deficient rats. *J Nutr* **103:** 267-272

Watmough NJ, Turnbull DM, Sherratt HSA, Bartlett K (1989) Measurement of the Acyl-Coa Intermediates of Beta-Oxidation by Hplc with Online Radiochemical and Photodiode-Array Detection - Application to the Study of [U-C-14]Hexadecanoate Oxidation by Intact Rat-Liver Mitochondria. *Biochemical Journal* **262:** 261-269

Weber G, Lui MS, Jayaram HN, Pillwein K, Natsumeda Y, Faderan MA, Reardon MA (1985) Regulation of purine and pyrimidine metabolism by insulin and by resistance to tiazofurin. *Adv Enzyme Regul* **23:** 81-99

Werkheiser WC, Bartley W (1957) The study of steady-state concentrations of internal solutes of mitochondria by rapid centrifugal transfer to a fixation medium. *Biochem J* **66:** 79-91

Williams JA, Withrow CD, Woodbury DM (1971a) Effects of CO2 on transmembrane potentials of rat liver and muscle in vivo. *J Physiol* **215:** 539-555

Williams JA, Withrow CD, Woodbury DM (1971b) Effects of ouabain and diphenylhydantoin on transmembrane potentials, intracellular electrolytes, and cell pH of rat muscle and liver in vivo. *J Physiol* **212:** 101-115

Williamson DH, Lund P, Krebs HA (1967) The redox state of free nicotinamide-adenine dinucleotide in the cytoplasm and mitochondria of rat liver. *Biochem J* **103:** 514-527

Williamson JR, Scholz R, Browning ET, Thurman RG, Fukami MH (1969) Metabolic effects of ethanol in perfused rat liver. *J Biol Chem* **244:** 5044-5054

Woods HF, Krebs HA (1973) Xylitol metabolism in the isolated perfused rat liver. *Biochem J* **134:** 437-443

Zakim D, Pardini RS, Herman RH, Sauberli.He (1967) Mechanism for Differential Effects of High Carbohydrate Diets on Lipigenesis in Rat Liver. *Biochimica Et Biophysica Acta* **144:** 242-&

Zollner H (1981) Regulation of urea synthesis. The effect of ammonia on the N-acetylglutamate content of isolated rat liver cells. *Biochim Biophys Acta* **676:** 170-176

Zuurendonk PF, Tager JM (1974) Rapid separation of particulate components and soluble cytoplasm of isolated rat-liver cells. *Biochim Biophys Acta* **333:** 393-399
