# Supplementary material for: HEPATOKIN1 is a biochemistry-based model of liver metabolism for applications in medicine and pharmacology
Source: Nat Commun. 2018 Jun 19;9:2386. doi: 10.1038/s41467-018-04720-9 (PMC6008457; doi:10.1038/s41467-018-04720-9)
Supplement: Supplementary file 5 — Supplementary Data 2 [file 41467_2018_4720_MOESM5_ESM.pdf]

Supplementary Information to

A Biochemistry-Based Model of Liver Metabolism for Applications in Medicine and Pharmacology
N. Berndt et al.

Supplementary Data 2 Absolute values of the time-average control coefficients

The regulatory impact of each of the 209 enzymes on 24 different metabolic liver functions was assessed by the time-average control coefficient (see also equation (6) in Methods)

$$\langle R \rangle_T = \frac{\int_0^T \left| Y_{pert} - Y_{ref} \right| dt}{\int_0^T \left| Y_{ref} \right| dt}$$

where Y_{ref} and Y_{pert} denote the time-dependent changes of model variable Y in the absence (ref) and presence (pert) of a perturbation and T is the time interval of interest. For a better comparison of the regulatory effect exerted by the various enzymes on a specific liver function we computed the relative values shown in Fig. 5 of the main document by dividing positive absolute $\langle R \rangle_{24h}$ values by the maximal positive value in the respective column of the table below and negative $\langle R \rangle_{24h}$ values by the absolute value of the minimal column value. This normalization yields values between 1 and -1 without shifting the “0”-point. Enzyme numbers in the first column are identical with those in Supplementary Table 1.

Absolute $\langle R \rangle_{24h}$ values

# enzyme	glucose exchange flux	lactate exchange flux	pyruvate exchange flux	glycerol exchange flux	ffa uptake	acetoacetate flux	ß-hydoxy-buturate flux	oxygen consumption rate	nh3 uptake	Gln exchange flux	Glu exchange flux	Ser exchange flux	Ala exchange flux	urea production	acetate flux	vldl export	Glycogen content	TAG content	cholesterol synthesis	fatty acid synthesis	Mitochondrial membrane potential	ATP/ADP	NAD/NADH	NADP/NADPH
1	1.8E-02	-7.9E-03	-1.5E-03	9.8E-04	-4.2E-02	5.6E-02	3.3E-02	-4.3E-04	4.8E-04	-2.7E-03	-6.6E-03	4.1E-06	1.1E-03	8.1E-03	9.3E-03	-3.1E-02	1.1E-02	-2.8E-02	-1.1E-04	6.2E-03	1.6E-04	-1.1E-02	1.6E-03	3.3E-03
2	2.9E-03	-1.3E-03	-2.9E-04	1.9E-04	-7.7E-03	1.0E-02	5.8E-03	-7.5E-05	8.3E-05	-4.9E-04	-1.1E-03	1.9E-06	1.9E-04	1.3E-03	1.6E-03	-5.5E-03	2.4E-03	-4.9E-03	-1.9E-05	1.0E-03	7.6E-06	-1.9E-03	3.2E-04	4.9E-04
3	1.4E-02	-8.3E-03	-1.2E-03	6.7E-04	-3.0E-02	3.5E-02	2.1E-02	-2.5E-04	2.9E-04	-1.7E-03	-4.0E-03	-1.3E-06	6.9E-04	5.0E-03	6.1E-03	-2.2E-02	8.0E-03	-2.0E-02	-1.5E-05	4.4E-03	4.7E-05	-6.3E-03	1.0E-03	2.5E-03
4	1.6E-02	-9.3E-03	-1.4E-03	7.3E-04	-3.3E-02	3.9E-02	2.4E-02	-2.8E-04	3.3E-04	-1.9E-03	-4.6E-03	2.0E-06	7.7E-04	5.7E-03	7.1E-03	-2.4E-02	8.9E-03	-2.3E-02	-3.1E-05	4.9E-03	6.9E-05	-7.2E-03	1.2E-03	2.8E-03

5	2.2E-03	-1.5E-03	-2.5E-04	1.1E-04	-5.0E-03	6.2E-03	3.5E-03	-3.2E-05	3.9E-05	-3.0E-04	-6.9E-04	7.4E-07	1.1E-04	8.2E-04	1.0E-03	-3.6E-03	2.2E-03	-3.4E-03	-1.1E-05	6.8E-04	-2.0E-05	-1.2E-03	2.4E-04	3.4E-04
6	-8.2E-03	4.4E-02	5.6E-03	-6.2E-04	-1.3E-02	-1.4E-02	3.6E-02	-3.7E-03	-1.7E-03	4.0E-03	1.3E-02	-1.0E-07	-2.5E-03	-1.3E-02	4.8E-03	1.9E-02	-1.9E-03	1.7E-02	1.0E-03	-6.8E-03	6.2E-04	2.8E-02	-4.4E-03	-4.4E-03
7	-1.5E-04	-8.7E-05	-5.0E-05	-4.4E-06	7.9E-06	3.5E-04	-1.8E-05	7.3E-06	6.3E-06	-3.0E-05	-5.2E-05	-1.9E-07	-1.6E-06	2.1E-05	6.7E-05	7.0E-06	4.9E-04	1.1E-06	-6.8E-06	-3.0E-05	-2.0E-05	-1.7E-04	6.5E-05	-4.0E-05
8	-1.9E-04	-9.6E-05	-3.7E-05	-4.4E-06	-3.5E-06	3.7E-04	-1.3E-04	8.8E-06	1.2E-05	-3.5E-05	-5.8E-05	2.4E-06	1.1E-05	-5.3E-06	-4.3E-05	2.4E-05	6.7E-04	1.8E-05	-6.7E-06	-1.4E-05	-1.6E-05	-2.2E-04	4.8E-05	-2.7E-05
9	-2.3E-04	-1.0E-04	-4.3E-05	-7.4E-06	3.0E-07	8.6E-04	1.3E-04	8.0E-06	1.7E-05	-8.1E-05	-1.4E-04	-2.6E-06	2.0E-05	1.1E-04	1.0E-04	3.5E-05	7.1E-04	2.3E-05	-1.0E-05	2.0E-05	-1.1E-05	-3.9E-04	5.9E-05	5.4E-06
10	-2.4E-04	-1.5E-04	-6.3E-05	-8.8E-06	-2.4E-05	5.8E-04	1.7E-05	1.1E-05	4.0E-06	-4.3E-05	-6.8E-05	-1.6E-07	7.1E-06	1.4E-04	9.7E-05	2.8E-05	9.6E-04	3.6E-05	-1.0E-05	-2.6E-05	-2.5E-05	-2.6E-04	8.1E-05	-4.4E-05
11	-2.7E-04	-4.5E-04	-3.0E-05	-1.4E-05	-5.6E-05	1.1E-03	1.1E-04	2.4E-05	-2.5E-05	-6.9E-05	7.8E-05	1.5E-06	7.0E-05	-1.2E-04	9.8E-05	4.4E-05	1.2E-03	5.5E-05	-2.0E-05	1.4E-05	-2.0E-05	-4.9E-04	9.3E-06	-1.5E-05
12	-2.5E-04	-1.7E-04	-4.5E-05	-1.4E-05	-3.6E-05	8.4E-04	6.5E-05	1.1E-05	2.0E-05	-6.4E-05	-1.6E-04	-9.6E-07	2.3E-05	1.4E-04	5.3E-05	3.3E-05	1.0E-03	3.7E-05	-2.0E-05	4.8E-07	-1.2E-05	-4.1E-04	6.0E-05	-2.4E-05
13	-2.8E-04	-1.5E-04	-3.8E-05	-1.1E-05	-3.9E-05	5.7E-04	-1.7E-04	1.5E-05	2.0E-05	-4.1E-05	-1.0E-04	2.5E-07	1.9E-05	5.9E-05	-6.6E-05	2.6E-05	1.1E-03	3.6E-05	-1.6E-05	-1.2E-05	-1.6E-05	-3.3E-04	5.5E-05	-3.8E-05
14	-3.1E-04	-3.1E-04	-1.2E-04	-1.9E-05	-2.6E-05	3.5E-03	1.5E-03	2.7E-05	2.7E-05	-7.5E-05	-1.2E-04	-1.6E-06	6.0E-06	1.6E-04	-8.7E-05	2.4E-05	1.1E-03	4.0E-05	-1.2E-05	-4.3E-05	-4.6E-05	-5.3E-04	1.8E-04	-6.5E-05
15	-2.1E-04	-1.6E-04	-4.9E-05	-6.0E-06	-3.8E-05	5.6E-04	-3.9E-05	9.3E-06	1.3E-05	-4.1E-05	-8.3E-05	5.5E-07	1.3E-05	9.7E-05	1.8E-05	3.0E-05	1.0E-03	4.3E-05	-1.0E-06	3.2E-05	-1.7E-05	-2.9E-04	5.9E-05	1.7E-05
16	-1.1E-04	-1.3E-04	-4.8E-05	-9.8E-06	-6.1E-06	-1.6E-04	-2.8E-04	1.1E-05	-5.2E-06	-4.1E-06	7.8E-05	-2.1E-06	-1.8E-05	-5.0E-05	-2.3E-05	-8.0E-06	5.7E-04	1.8E-06	-9.6E-06	-1.1E-04	-3.5E-05	8.7E-06	6.5E-05	-1.2E-04
17	-2.7E-04	-1.8E-04	-6.1E-05	1.0E-06	-3.8E-05	8.9E-04	7.6E-05	1.2E-05	1.4E-05	-8.1E-05	-1.6E-04	4.0E-07	1.9E-05	1.2E-04	8.3E-05	3.5E-05	1.1E-03	4.2E-05	-1.5E-05	9.1E-06	-1.6E-05	-4.1E-04	7.6E-05	-1.4E-05
18	-2.2E-04	-1.9E-04	-6.0E-05	-8.8E-06	-1.8E-05	4.2E-04	-1.1E-04	1.3E-05	7.5E-06	-5.9E-05	-5.5E-05	1.7E-06	5.4E-06	8.7E-05	2.7E-05	2.0E-05	9.7E-04	2.5E-05	-1.5E-05	-5.6E-05	-2.7E-05	-2.3E-04	7.5E-05	-7.5E-05
19	-2.1E-04	-1.9E-04	-5.4E-05	-1.5E-05	-8.2E-06	2.3E-04	-2.0E-04	1.4E-05	5.3E-06	-4.6E-05	-1.4E-05	-2.5E-07	-3.4E-06	1.1E-05	-6.8E-06	2.1E-05	1.1E-03	2.5E-05	-1.0E-05	-6.4E-05	-2.9E-05	-1.5E-04	6.9E-05	-7.9E-05
20	-1.6E-04	-1.2E-04	-4.3E-05	-1.1E-06	-2.5E-05	3.1E-04	-1.7E-04	9.7E-06	1.1E-05	-4.5E-05	-3.4E-05	1.5E-06	1.4E-06	-1.4E-06	-2.3E-05	8.9E-06	6.9E-04	2.0E-05	-5.1E-06	-2.3E-05	-2.3E-05	-2.1E-04	5.9E-05	-3.7E-05
21	-4.9E-03	-9.3E-03	-7.8E-03	4.9E-04	1.2E-03	2.0E-01	1.7E-01	1.4E-04	2.4E-04	-8.9E-03	-1.7E-02	7.3E-07	-1.3E-03	3.1E-02	6.1E-02	-1.0E-03	5.1E-04	-1.2E-03	-1.7E-03	-3.5E-03	1.8E-04	-1.8E-02	1.1E-02	-4.2E-03
22	3.5E-03	-1.3E-02	-7.6E-03	3.9E-04	6.2E-03	-1.8E-02	-2.7E-02	1.6E-04	-6.1E-04	1.1E-03	4.3E-03	5.0E-07	-2.6E-03	-1.2E-03	-9.1E-03	-5.3E-03	-1.6E-03	-5.7E-03	-6.7E-03	-3.0E-02	5.5E-04	-3.5E-02	1.0E-02	-3.3E-02
23	-1.4E-04	-1.3E-04	-5.2E-05	-1.2E-05	-7.1E-06	2.2E-04	-6.8E-05	1.0E-05	1.2E-06	-2.7E-05	3.5E-06	-1.7E-07	-9.9E-06	7.6E-06	7.7E-05	7.5E-06	6.5E-04	1.3E-05	-6.0E-06	-6.0E-05	-2.7E-05	-9.4E-05	7.4E-05	-6.9E-05
24	-1.3E-02	4.4E-02	-1.8E-02	3.1E-03	-1.7E-03	-5.5E-02	-5.2E-02	-4.2E-03	-3.0E-03	7.0E-03	2.6E-02	1.7E-06	-1.2E-02	-1.4E-02	-2.4E-02	-6.6E-04	-1.9E-02	-7.6E-04	-1.6E-03	2.1E-02	2.2E-03	-4.1E-02	2.8E-02	2.2E-02

25	5.3E-03	-5.3E-03	1.1E-02	-1.4E-03	-2.1E-03	-5.1E-02	2.9E-04	6.5E-04	2.8E-03	-4.8E-03	-2.1E-02	-1.5E-07	1.4E-02	3.2E-03	-5.7E-03	2.4E-03	1.2E-02	2.8E-03	4.7E-03	6.8E-03	-9.1E-04	3.8E-02	-1.5E-02	8.3E-03
26	0.0E+00	0.0E+00	0.0E+00	0.0E+00	0.0E+00	0.0E+00	0.0E+00	0.0E+00	0.0E+00	0.0E+00	0.0E+00	0.0E+00	0.0E+00	0.0E+00	0.0E+00	0.0E+00	0.0E+00	0.0E+00	0.0E+00	0.0E+00	0.0E+00	0.0E+00	0.0E+00	0.0E+00
27	-2.4E-04	-2.0E-04	-5.5E-05	4.5E-06	-4.8E-05	1.4E-03	5.0E-04	7.3E-06	2.0E-05	-1.4E-04	-2.7E-04	5.4E-07	3.5E-05	3.4E-04	2.8E-04	2.6E-05	9.7E-04	3.6E-05	-2.8E-05	2.2E-05	-2.8E-06	-5.4E-04	6.7E-05	-4.4E-06
28	5.4E-05	-1.1E-03	-7.1E-05	-1.4E-05	1.5E-04	1.1E-03	4.5E-04	5.5E-05	2.8E-05	-1.5E-04	-2.6E-04	1.0E-06	7.2E-05	2.1E-04	3.7E-04	-1.3E-04	9.8E-04	-1.6E-04	-1.5E-04	-1.1E-03	-3.8E-05	-1.7E-04	7.2E-06	-1.2E-03
29	-2.4E-04	-1.1E-04	-4.4E-05	-1.1E-05	-2.3E-05	8.4E-04	9.7E-05	1.0E-05	1.8E-05	-3.1E-05	-1.3E-04	9.9E-07	1.8E-05	8.7E-05	8.4E-05	3.2E-05	8.8E-04	3.3E-05	-1.2E-05	1.0E-05	-1.2E-05	-3.7E-04	6.3E-05	-1.0E-05
30	-1.8E-04	-1.9E-04	-3.1E-05	-2.0E-05	-1.1E-05	1.2E-04	-2.4E-04	1.7E-05	3.4E-06	-3.3E-05	1.3E-06	-4.0E-08	-8.2E-06	4.4E-06	-4.2E-05	9.5E-06	7.3E-04	1.5E-05	-1.6E-05	-9.7E-05	-2.8E-05	-2.0E-04	4.2E-05	-1.2E-04
31	-2.3E-04	-1.3E-04	-5.1E-05	-9.3E-06	-2.6E-05	5.1E-04	-4.5E-05	1.1E-05	8.0E-06	-6.1E-05	-5.6E-05	1.9E-06	1.1E-05	9.3E-05	4.0E-05	3.0E-05	9.3E-04	3.9E-05	-7.7E-06	-1.4E-05	-2.1E-05	-2.6E-04	6.7E-05	-3.0E-05
32	-1.8E-04	-1.1E-04	-3.8E-05	-1.4E-05	-3.6E-06	6.6E-04	1.1E-04	6.3E-06	1.3E-05	-4.1E-05	-9.4E-05	-5.3E-07	8.6E-06	1.7E-04	1.1E-04	3.6E-05	7.4E-04	3.2E-05	-1.9E-06	2.7E-05	-1.4E-05	-2.9E-04	5.8E-05	1.7E-05
33	-1.5E-04	3.3E-04	2.7E-04	-5.0E-06	-3.0E-04	1.0E-02	5.8E-03	-8.9E-05	2.0E-04	-7.4E-04	-2.4E-03	2.0E-06	4.4E-04	2.5E-03	1.5E-03	3.4E-04	-2.4E-04	3.0E-04	-9.5E-05	1.3E-03	4.0E-04	-3.6E-03	-4.3E-04	1.3E-03
34	-2.4E-03	3.7E-03	-2.9E-04	-2.1E-04	-1.7E-04	-1.2E-02	-3.8E-03	-5.4E-05	-2.3E-04	1.0E-03	3.5E-03	-3.4E-08	-5.6E-04	-3.9E-03	-1.2E-03	2.6E-04	5.0E-03	3.2E-04	5.1E-04	-4.2E-05	-1.1E-03	8.1E-03	7.6E-04	-9.7E-05
35	2.8E-03	8.3E-03	5.0E-03	4.3E-04	-9.1E-03	3.1E-01	1.9E-01	-5.0E-03	4.9E-03	-2.0E-02	-8.1E-02	-2.8E-07	1.4E-02	8.1E-02	4.7E-02	9.4E-03	-3.5E-02	7.4E-03	-6.5E-03	4.2E-02	1.5E-02	-1.4E-01	-9.5E-03	4.0E-02
36	-2.3E-02	3.5E-02	-1.3E-02	-2.3E-03	4.6E-03	-4.0E-01	-1.6E-01	-4.5E-04	-9.5E-03	3.3E-02	9.8E-02	1.2E-07	-1.8E-02	-1.1E-01	-3.7E-02	-3.9E-03	6.2E-02	-2.6E-03	7.3E-03	-2.9E-02	-2.4E-02	1.8E-01	2.4E-02	-2.8E-02
37	2.0E-02	-4.4E-02	-2.0E-03	2.2E-03	2.0E-03	1.0E-01	2.5E-02	-5.8E-03	1.6E-03	-1.0E-02	-3.3E-02	1.1E-06	3.6E-03	4.0E-02	9.7E-03	-3.0E-03	-3.1E-02	-3.6E-03	-5.6E-03	-3.5E-03	-1.3E-02	-7.4E-02	-1.8E-03	-3.2E-03
38	3.6E-03	-2.0E-02	3.2E-03	-4.2E-04	-5.1E-04	1.4E-02	4.2E-03	9.2E-04	7.1E-04	5.7E-03	6.6E-03	-4.2E-06	2.9E-03	-2.0E-02	2.8E-03	7.1E-04	-1.0E-03	6.8E-04	-6.2E-04	7.6E-04	-6.9E-04	-1.1E-02	-6.0E-03	4.6E-04
39	2.5E-02	-5.5E-02	8.3E-04	2.6E-03	1.1E-04	2.3E-01	9.2E-02	-8.3E-03	3.9E-03	-1.9E-02	-6.4E-02	-1.6E-06	9.6E-03	7.2E-02	2.8E-02	-1.9E-04	-4.9E-02	-1.4E-03	-8.6E-03	1.0E-02	-1.2E-02	-1.2E-01	-7.6E-03	9.8E-03
40	1.5E-01	-4.0E-01	-2.4E-02	1.1E-02	2.2E-02	3.0E-01	-9.3E-02	-6.2E-02	2.8E-03	-2.5E-02	-1.0E-01	5.1E-07	7.9E-03	1.2E-01	1.4E-02	-3.0E-02	-1.5E-01	-3.1E-02	-3.7E-02	-7.4E-02	3.6E-02	-2.9E-01	2.3E-03	-6.8E-02
41	6.2E-05	-8.9E-04	3.4E-05	-6.5E-06	3.2E-06	6.5E-04	-1.5E-04	-9.9E-05	5.6E-06	-5.2E-05	-1.4E-04	-8.4E-07	1.8E-05	1.5E-04	6.5E-05	-8.5E-06	4.7E-04	-1.0E-05	-4.1E-05	-1.2E-04	3.1E-05	-5.4E-04	-1.2E-04	-1.3E-04
42	1.3E-01	-4.1E-01	3.7E-02	2.2E-03	7.1E-03	1.6E-01	-4.4E-02	-5.6E-02	8.1E-04	-1.5E-02	-5.8E-02	9.6E-07	7.8E-03	6.6E-02	1.2E-02	-9.8E-03	-9.8E-02	-1.1E-02	-1.7E-02	-3.7E-02	2.5E-02	-1.8E-01	-8.5E-02	-3.4E-02
43	-1.3E-04	-1.2E-04	-5.4E-05	-2.1E-06	-1.6E-05	8.9E-05	-1.8E-04	1.1E-05	8.3E-07	-4.5E-05	5.6E-06	3.8E-06	-3.1E-06	-1.9E-05	9.6E-06	-9.4E-06	6.1E-04	3.0E-06	-9.2E-06	-7.3E-05	-2.8E-05	-5.9E-05	6.8E-05	-8.6E-05
44	1.1E-03	-2.0E-03	-1.9E-04	-1.4E-04	-1.5E-04	-1.0E-02	-1.0E-03	1.6E-06	-2.3E-04	7.2E-04	1.9E-03	-1.3E-06	-3.2E-04	-2.4E-03	-5.9E-04	3.5E-05	-2.0E-02	1.3E-04	2.5E-04	3.0E-04	-1.8E-04	4.8E-03	-7.3E-05	3.7E-04
45	-3.0E-02	6.8E-02	-6.1E-04	-3.4E-03	-4.9E-03	-1.3E-01	-7.4E-03	9.2E-04	-2.7E-03	1.2E-02	3.5E-02	-1.7E-06	-5.6E-03	-4.1E-02	-8.7E-03	5.9E-03	4.6E-02	6.3E-03	7.2E-03	1.0E-02	-2.0E-03	1.1E-01	6.8E-03	9.4E-03
46	-6.1E-05	-9.1E-05	-2.2E-05	-3.2E-06	-1.9E-05	2.7E-04	3.9E-05	-7.5E-07	6.2E-06	-1.0E-05	-5.5E-05	4.2E-06	9.0E-06	7.6E-06	3.6E-05	6.7E-06	4.4E-04	1.4E-05	-5.9E-06	-1.3E-06	-3.0E-06	-1.3E-04	1.9E-05	-9.2E-06

47	-2.3E-02	4.6E-02	9.2E-04	-2.4E-03	-4.6E-03	-4.0E-02	1.6E-02	-1.8E-03	-7.1E-04	5.0E-03	1.5E-02	2.9E-06	-1.8E-03	-1.9E-02	-9.1E-04	5.6E-03	2.9E-02	5.7E-03	4.7E-03	1.3E-02	-7.6E-03	6.1E-02	2.2E-03	1.2E-02
48	-5.3E-03	1.7E-02	-5.9E-04	5.0E-05	-5.9E-04	1.0E-01	2.3E-02	2.0E-04	1.9E-03	-7.0E-03	-2.0E-02	3.8E-06	3.5E-03	2.3E-02	9.4E-03	2.3E-03	-7.4E-03	1.5E-03	-1.8E-03	4.2E-03	5.1E-04	-4.8E-02	3.5E-03	2.9E-03
49	-8.4E-02	1.1E-01	1.0E-02	2.1E-03	2.4E-03	-1.6E-01	-4.0E-02	-1.9E-03	-3.1E-03	1.2E-02	3.5E-02	-1.4E-07	-6.1E-03	-4.0E-02	-1.9E-02	-5.2E-03	-7.8E-03	-4.7E-03	4.6E-03	-9.4E-03	-4.3E-04	8.2E-02	-4.0E-03	-4.6E-03
50	-1.0E-03	8.0E-04	4.6E-05	2.2E-05	2.0E-05	-1.9E-03	-6.3E-04	-8.2E-06	-4.0E-05	1.1E-04	4.4E-04	9.5E-07	-8.3E-05	-5.2E-04	-2.0E-04	-6.8E-05	8.4E-04	-6.0E-05	3.0E-05	-2.2E-04	-3.7E-05	9.3E-04	6.7E-06	-1.9E-04
51	4.8E-06	-2.0E-04	-8.1E-05	-1.5E-05	-8.0E-06	4.8E-04	-2.8E-06	1.7E-05	4.4E-06	-8.3E-06	-4.5E-05	-7.2E-08	4.0E-06	2.0E-05	6.1E-05	3.3E-05	6.6E-04	2.7E-05	-2.0E-06	8.6E-06	-2.8E-05	-1.6E-04	9.3E-05	-1.6E-05
52	6.7E-02	-5.6E-02	-1.3E-02	-6.1E-03	-8.1E-03	1.7E-01	6.1E-02	3.3E-03	3.1E-03	-1.1E-02	-2.7E-02	5.4E-07	5.2E-03	3.1E-02	2.3E-02	1.5E-02	4.9E-02	1.3E-02	6.3E-04	2.7E-02	-1.0E-03	-3.9E-02	1.4E-02	2.0E-02
53	-2.9E-04	-1.7E-04	-6.7E-05	-1.7E-05	-3.7E-05	8.0E-04	3.0E-05	1.2E-05	1.5E-06	-6.1E-05	-1.2E-04	-3.1E-07	1.8E-05	1.2E-04	7.4E-05	3.9E-05	1.2E-03	4.8E-05	-8.0E-06	1.9E-05	-2.1E-05	-3.9E-04	8.3E-05	-2.1E-06
54	-2.1E-04	-1.2E-04	-6.5E-05	-6.9E-06	-1.4E-05	7.9E-04	1.9E-04	9.5E-06	1.3E-05	-6.3E-05	-1.1E-04	9.6E-07	-2.0E-06	1.1E-04	2.1E-04	1.4E-05	7.5E-04	1.9E-05	-1.3E-05	-3.6E-05	-2.3E-05	-2.9E-04	9.4E-05	-5.3E-05
55	4.8E-04	-5.8E-04	-2.3E-04	-8.1E-05	-1.7E-04	3.4E-03	9.9E-04	5.8E-05	5.7E-05	-2.2E-04	-5.6E-04	1.5E-07	1.0E-04	5.5E-04	3.8E-04	2.6E-04	1.0E-03	2.4E-04	-3.8E-05	2.9E-04	-3.1E-05	-9.9E-04	3.1E-04	2.5E-04
56	-3.9E-02	3.2E-02	7.8E-03	3.6E-03	4.8E-03	-7.4E-02	-2.7E-02	-2.2E-03	-1.6E-03	5.1E-03	9.3E-03	2.8E-06	-2.0E-03	-1.2E-02	-1.0E-02	-8.2E-03	8.8E-03	-8.2E-03	-1.7E-03	-1.7E-02	7.9E-04	1.0E-02	-8.0E-03	-1.9E-02
57	4.1E-02	-2.8E-02	-8.9E-03	-5.0E-03	-6.2E-03	6.8E-02	3.1E-02	2.5E-03	1.4E-03	-4.2E-03	-6.1E-03	2.3E-06	1.6E-03	8.5E-03	1.1E-02	1.0E-02	-3.5E-03	1.0E-02	2.8E-03	2.1E-02	-1.2E-03	4.0E-03	1.0E-02	2.3E-02
58	-1.1E-02	1.6E-02	9.4E-04	5.2E-05	1.0E-04	-7.6E-03	-3.8E-03	-1.1E-04	-2.9E-05	4.5E-04	2.7E-03	-1.3E-06	-5.1E-04	-2.5E-03	-1.6E-03	-1.7E-04	9.3E-03	-8.4E-05	8.8E-04	2.5E-04	-1.1E-04	5.8E-03	1.1E-04	3.4E-05
59	2.4E-03	2.0E-02	-3.6E-03	-2.7E-03	-3.8E-03	5.0E-02	1.8E-02	1.3E-03	1.2E-03	-3.1E-03	-3.4E-03	3.4E-06	8.6E-04	5.6E-03	5.6E-03	6.6E-03	1.6E-02	6.4E-03	2.6E-03	1.3E-02	-7.7E-04	3.9E-03	7.4E-03	1.3E-02
60	3.9E-04	1.3E-03	-4.0E-04	-1.9E-04	-3.3E-04	9.0E-03	2.6E-03	1.2E-04	1.7E-04	-5.4E-04	-1.3E-03	5.6E-06	2.1E-04	1.5E-03	8.6E-04	6.5E-04	6.2E-04	5.2E-04	-2.5E-05	5.6E-04	-4.9E-05	-1.5E-03	8.2E-04	4.0E-04
61	-1.1E-04	-1.1E-04	-3.4E-05	-8.8E-06	-7.3E-06	1.8E-04	-6.9E-05	6.0E-06	5.8E-07	-2.2E-05	-1.4E-05	-1.9E-07	1.2E-06	2.0E-05	2.1E-05	4.0E-06	3.7E-04	3.9E-06	-6.5E-06	-3.0E-05	-1.7E-05	-1.1E-04	4.0E-05	-3.9E-05
62	-1.7E-04	-1.8E-04	-4.2E-05	-1.1E-06	-6.4E-06	2.7E-04	-1.2E-04	7.7E-06	4.4E-06	-3.4E-05	-1.3E-05	6.4E-06	6.0E-06	-4.3E-05	6.7E-06	-7.8E-07	6.7E-04	1.4E-06	-7.2E-09	-3.2E-05	-2.4E-05	1.1E-04	4.3E-05	-4.6E-05
63	-2.3E-04	-1.4E-04	-3.2E-05	-1.1E-05	-4.4E-05	9.7E-04	1.9E-04	4.5E-06	1.9E-05	-8.0E-05	-1.9E-04	1.1E-06	3.6E-05	1.8E-04	6.4E-05	4.8E-05	9.6E-04	5.7E-05	-6.2E-06	7.6E-05	2.3E-06	-2.8E-04	2.8E-05	5.9E-05
64	-2.9E-04	-2.7E-04	-4.7E-05	-3.6E-05	-6.4E-05	1.2E-03	2.4E-04	1.2E-05	2.4E-05	-8.3E-05	-2.1E-04	-3.3E-06	3.9E-05	9.8E-05	1.0E-04	3.9E-05	1.5E-03	5.3E-05	-2.8E-05	2.8E-05	-2.9E-06	-5.3E-04	5.2E-05	-4.9E-06
65	-1.6E-04	-1.2E-04	-5.7E-05	-5.6E-06	-2.1E-05	2.7E-04	-1.1E-04	9.0E-06	4.4E-06	-3.3E-05	-3.7E-05	6.3E-09	3.1E-06	-1.8E-05	1.6E-05	1.2E-05	7.3E-04	2.1E-05	-6.6E-06	-3.5E-05	-2.3E-05	-9.8E-05	6.4E-05	-5.0E-05
66	-1.7E-03	4.7E-03	-3.0E-03	4.6E-04	1.1E-03	2.7E-02	1.1E-02	-7.8E-05	-1.5E-04	-2.0E-03	-1.4E-02	-4.6E-07	2.5E-03	1.2E-02	6.5E-03	-1.9E-03	-1.2E-02	-2.3E-03	-6.3E-03	-8.7E-03	6.8E-04	-3.2E-02	4.4E-03	-1.1E-02
67	-9.0E-04	1.7E-03	-8.1E-05	3.7E-05	-3.0E-04	1.0E-02	2.7E-03	-5.7E-05	2.6E-04	-6.8E-04	-1.6E-03	8.1E-09	6.1E-04	1.3E-03	1.4E-03	2.4E-04	1.4E-03	2.6E-04	3.3E-05	1.8E-03	-8.7E-05	5.1E-03	2.5E-04	1.7E-03
68	-2.2E-04	-2.0E-04	-5.2E-	-1.0E-05	-6.2E-06	2.9E-04	-1.1E-04	1.2E-05	6.8E-06	-2.2E-05	-1.7E-05	-6.3E-07	-2.5E-07	-6.1E-05	5.2E-06	1.6E-05	9.9E-04	1.2E-05	-1.5E-05	-7.0E-05	-2.5E-05	-1.1E-04	5.8E-05	-9.2E-05

			05																					
69	-1.5E-02	3.0E-02	-3.1E-02	3.4E-03	6.1E-03	-3.3E-01	-1.0E-01	-6.2E-03	-6.0E-03	2.8E-02	7.5E-02	-8.1E-08	-1.9E-02	-7.9E-02	-3.9E-02	-1.0E-02	3.1E-02	-8.5E-03	7.6E-03	-1.8E-02	-1.1E-03	1.6E-01	4.6E-02	-1.3E-02
70	-2.1E-04	-1.2E-04	-6.3E-05	-6.3E-06	-8.9E-06	4.5E-04	9.7E-06	1.2E-05	1.1E-06	-3.4E-05	-4.0E-05	2.4E-06	-5.1E-06	7.8E-05	1.2E-04	1.4E-05	7.9E-04	2.2E-05	-8.7E-06	-5.3E-05	-2.4E-05	-1.6E-04	8.2E-05	-6.5E-05
71	1.1E-02	9.9E-02	-7.4E-02	-1.7E-03	-2.2E-03	-1.1E-01	-1.2E-02	1.5E-04	-2.8E-03	9.0E-03	2.4E-02	-6.1E-07	-1.6E-03	-3.2E-02	-7.5E-03	1.4E-03	9.8E-03	1.9E-03	4.0E-03	2.7E-04	-1.1E-03	6.7E-02	-2.6E-02	1.1E-03
72	-4.2E-03	7.7E-03	-8.2E-03	9.5E-04	1.5E-03	-8.3E-02	-2.4E-02	-1.7E-03	-1.6E-03	5.7E-03	1.8E-02	-4.0E-07	-2.7E-03	-2.0E-02	-1.1E-02	-2.4E-03	7.6E-03	-1.9E-03	1.9E-03	-2.9E-03	-9.7E-05	2.9E-02	1.3E-02	-1.5E-03
73	0.0E+00	0.0E+00	0.0E+00	0.0E+00	0.0E+00	0.0E+00	0.0E+00	0.0E+00	0.0E+00	0.0E+00	0.0E+00	0.0E+00	0.0E+00	0.0E+00	0.0E+00	0.0E+00	0.0E+00	0.0E+00	0.0E+00	0.0E+00	0.0E+00	0.0E+00	0.0E+00	0.0E+00
74	-1.8E-04	-1.2E-04	-5.5E-05	-6.6E-06	5.3E-06	2.7E-04	-3.6E-05	1.5E-05	1.0E-06	-1.6E-05	4.7E-05	-2.7E-07	-3.5E-05	-1.1E-04	1.3E-04	2.1E-06	6.4E-04	6.7E-06	-5.1E-06	-1.0E-04	-3.4E-05	9.5E-05	7.5E-05	-1.2E-04
75	-2.8E-04	-1.7E-04	-6.9E-05	-8.8E-06	-3.1E-05	1.1E-03	1.7E-04	1.2E-05	2.4E-05	-7.7E-05	-1.6E-04	2.4E-06	1.8E-05	1.7E-04	1.7E-04	1.7E-05	1.1E-03	2.2E-05	-2.5E-05	-3.6E-05	-2.3E-05	-4.1E-04	9.8E-05	-6.3E-05
76	-1.4E-04	-4.3E-04	3.9E-05	-2.2E-05	-5.5E-05	5.8E-04	-1.3E-05	1.9E-05	-1.9E-06	-8.1E-05	-1.5E-04	5.5E-07	2.6E-05	2.2E-05	4.8E-05	2.6E-05	1.2E-03	4.9E-05	-5.3E-05	2.2E-05	-2.1E-05	-3.9E-04	-6.9E-05	9.9E-04
77	-1.4E-04	-1.0E-04	-4.4E-05	-1.2E-05	-1.6E-05	2.4E-04	-8.9E-05	7.9E-06	-4.2E-06	-3.6E-05	-3.1E-05	1.1E-07	9.4E-07	1.9E-06	1.1E-05	7.4E-06	6.1E-04	1.4E-05	-8.2E-06	-3.6E-05	-1.7E-05	-1.3E-04	4.9E-05	-4.8E-05
78	-1.1E-03	1.7E-03	-1.6E-04	2.7E-05	-1.8E-04	4.5E-03	1.5E-03	-3.6E-05	1.0E-04	-3.4E-04	-7.7E-04	8.2E-07	3.8E-04	4.9E-04	9.3E-04	1.9E-04	1.8E-03	2.2E-04	-6.3E-05	1.2E-03	-4.9E-05	1.9E-03	3.5E-04	1.1E-03
79	-1.6E-04	-6.7E-05	-4.2E-05	1.1E-06	3.5E-06	5.1E-05	-2.1E-04	8.3E-06	-1.0E-06	1.7E-05	1.5E-05	2.9E-07	-1.6E-06	-9.7E-06	-4.5E-05	1.9E-05	5.4E-04	1.6E-05	-4.4E-06	-4.2E-05	-2.1E-05	-9.6E-05	4.9E-05	-5.0E-05
80	-2.2E-04	-1.3E-04	-6.4E-05	-7.9E-06	-1.7E-05	4.8E-04	-5.3E-05	1.1E-05	1.8E-06	-4.0E-05	-7.2E-05	-1.7E-06	3.9E-06	7.2E-05	4.9E-05	2.3E-05	8.8E-04	2.9E-05	-1.0E-05	-3.2E-05	-2.5E-05	-2.5E-04	7.7E-05	-4.9E-05
81	2.9E-03	-5.3E-03	-4.0E-04	-2.7E-04	-2.6E-04	-2.1E-02	-2.5E-03	2.4E-05	-4.6E-04	1.5E-03	4.1E-03	1.2E-06	-6.9E-04	-4.9E-03	-1.1E-03	-2.4E-05	-4.1E-02	2.7E-04	5.5E-04	6.6E-04	-2.9E-04	1.1E-02	-2.6E-04	8.8E-04
82	9.5E-03	-1.6E-02	-1.0E-03	-1.1E-03	-8.3E-04	-9.9E-02	-1.3E-02	3.4E-04	-2.1E-03	7.4E-03	2.0E-02	1.4E-06	-3.1E-03	-2.4E-02	-5.9E-03	-6.8E-05	-1.5E-01	1.0E-03	2.3E-03	1.7E-03	-6.3E-04	5.5E-02	-1.3E-03	2.5E-03
83	-2.0E-02	3.7E-02	1.1E-03	3.7E-04	-2.2E-05	9.3E-02	1.2E-02	-1.5E-05	2.2E-03	-7.1E-03	-1.6E-02	2.4E-06	2.5E-03	2.0E-02	4.8E-03	1.4E-03	2.2E-01	6.8E-04	-4.3E-04	1.2E-03	3.2E-04	-3.7E-02	2.9E-03	2.7E-04
84	1.0E-04	-1.8E-03	7.7E-04	-1.1E-04	-2.1E-04	-2.9E-03	8.2E-04	1.1E-04	-9.5E-05	2.3E-04	7.1E-04	3.7E-06	-3.2E-06	-1.0E-03	-3.6E-05	2.1E-04	1.6E-03	2.1E-04	1.5E-04	1.9E-04	-8.5E-05	2.3E-03	-1.4E-03	1.6E-04
85	-2.6E-04	-1.8E-04	-5.9E-05	-1.7E-05	-2.5E-05	4.3E-04	-2.1E-04	1.4E-05	-3.4E-08	-4.3E-05	-5.9E-05	1.8E-06	6.6E-06	2.6E-05	-3.1E-05	2.3E-05	1.1E-03	3.0E-05	-9.0E-06	-2.8E-05	-3.0E-05	-2.8E-04	8.0E-05	-4.9E-05
86	3.6E-03	-1.5E-02	8.7E-03	-1.2E-03	-1.9E-03	-2.3E-02	7.6E-03	1.2E-03	-6.5E-04	1.9E-03	5.5E-03	2.8E-06	2.2E-04	-8.1E-03	4.8E-05	2.1E-03	5.7E-03	2.1E-03	1.7E-03	3.5E-03	-6.9E-04	1.9E-02	-1.4E-02	3.6E-03
87	-1.3E-04	-1.6E-04	-1.0E-05	-1.2E-05	-1.6E-05	7.6E-05	-2.0E-04	1.4E-05	4.7E-06	-5.6E-06	5.3E-05	2.9E-07	-4.1E-05	-6.9E-05	-3.5E-05	1.3E-05	6.7E-04	1.8E-05	-3.0E-06	-3.6E-05	-3.1E-05	-1.1E-04	8.4E-06	-4.9E-05
88	-2.4E-04	-1.8E-04	7.9E-05	1.6E-05	-3.4E-05	1.3E-03	3.9E-04	-2.7E-05	3.6E-05	-1.0E-04	-3.2E-04	3.6E-06	6.8E-05	2.5E-04	2.8E-05	3.4E-05	4.9E-04	3.3E-05	-1.1E-05	1.5E-04	4.0E-05	-5.9E-04	-1.3E-04	1.4E-04
89	1.8E-02	-6.7E-02	1.5E-02	-1.1E-03	-1.5E-03	-1.7E-02	7.7E-04	-7.6E-03	-8.0E-04	1.1E-03	2.1E-03	3.2E-06	7.3E-04	-4.1E-03	3.4E-04	1.3E-03	-6.5E-03	1.2E-03	4.4E-04	-5.4E-04	1.9E-03	4.5E-03	-2.8E-02	-3.5E-04

90	-4.8E-04	3.0E-04	-7.4E-05	-1.3E-05	4.1E-05	-1.8E-03	-9.6E-04	2.2E-05	-1.3E-05	1.0E-04	4.3E-04	-1.6E-08	-5.8E-05	-5.2E-04	-4.0E-04	1.3E-04	8.1E-04	1.8E-04	-3.6E-03	3.1E-05	-1.1E-05	9.5E-04	1.3E-04	1.3E-01
91	-1.7E-04	-1.2E-04	-4.8E-05	-6.5E-06	-1.4E-05	5.6E-04	-1.3E-05	4.8E-06	1.3E-05	-7.8E-05	-1.1E-04	6.9E-07	1.2E-05	9.7E-05	4.4E-05	1.3E-05	6.1E-04	1.6E-05	-9.8E-06	7.0E-08	-2.5E-05	-3.6E-04	6.8E-05	-1.2E-05
92	8.8E-06	-1.3E-04	-4.6E-05	-1.4E-05	5.0E-07	2.8E-04	-7.7E-05	-7.0E-06	4.6E-06	-3.6E-05	-8.3E-05	-1.2E-06	-3.6E-06	7.7E-05	2.5E-05	1.1E-05	3.5E-04	9.6E-06	1.0E-06	1.6E-06	-4.6E-05	-4.1E-04	5.6E-05	3.6E-06
93	-2.0E-04	-1.2E-04	-5.3E-05	-3.2E-05	-2.8E-05	3.4E-04	-8.2E-05	1.1E-05	3.7E-06	-1.8E-05	-2.6E-05	-4.5E-06	-1.5E-06	3.5E-05	3.9E-05	1.5E-05	8.6E-04	2.8E-05	-8.6E-06	-3.4E-05	-2.5E-05	-1.8E-04	6.9E-05	-5.0E-05
94	-2.3E-04	-1.4E-04	-4.7E-05	-7.4E-06	-2.8E-05	9.6E-04	2.1E-04	8.7E-06	1.5E-05	-7.3E-05	-1.6E-04	2.1E-07	2.1E-05	1.5E-04	1.4E-04	3.4E-05	8.5E-04	3.6E-05	-2.1E-05	-4.4E-06	-9.6E-06	-4.1E-04	6.1E-05	-2.4E-05
95	-1.8E-04	-1.9E-04	-5.8E-05	-8.2E-06	-4.4E-05	1.1E-03	1.8E-04	4.5E-06	2.4E-05	-1.0E-04	-2.3E-04	6.9E-07	2.2E-05	2.1E-04	1.3E-04	2.0E-05	9.1E-04	3.2E-05	-2.6E-05	-1.0E-05	-2.5E-05	-5.9E-04	7.5E-05	-3.3E-05
96	-1.8E-04	-1.8E-04	-4.6E-05	-1.0E-05	-3.1E-05	3.8E-04	-9.5E-05	1.2E-05	1.2E-05	-3.4E-05	-4.8E-05	-1.5E-06	1.2E-06	5.2E-05	2.9E-05	1.0E-05	9.5E-04	2.4E-05	-1.4E-05	-5.0E-05	-2.4E-05	-2.1E-04	6.6E-05	-6.9E-05
97	-2.9E-04	-1.9E-04	-5.2E-05	-8.3E-06	-3.9E-05	8.8E-04	-2.9E-05	1.3E-05	2.0E-05	-8.2E-05	-1.7E-04	-3.2E-07	2.5E-05	1.7E-04	8.6E-06	3.7E-05	1.2E-03	4.2E-05	-1.8E-05	3.6E-06	-1.7E-05	-4.6E-04	7.0E-05	-2.2E-05
98	7.1E-04	-4.0E-03	-4.5E-05	-1.0E-04	7.0E-04	1.2E-04	-6.0E-04	1.9E-04	4.0E-05	-7.4E-05	-2.4E-04	3.8E-06	1.5E-04	7.0E-06	-1.5E-04	-5.5E-04	1.7E-03	-6.2E-04	-6.1E-04	-4.5E-03	-8.6E-05	2.3E-04	-3.0E-04	-4.8E-03
99	1.8E-03	-1.9E-02	3.3E-04	-7.5E-04	5.1E-03	-1.9E-04	-4.8E-03	1.3E-03	3.2E-04	-2.3E-04	-1.1E-04	4.3E-06	9.3E-04	-1.3E-03	-2.2E-03	-3.6E-03	1.2E-02	-4.4E-03	-9.4E-03	-3.1E-02	-5.5E-04	4.0E-03	-2.0E-03	-3.5E-02
100	-1.1E-03	-1.1E-02	2.6E-03	-7.9E-04	9.3E-04	-1.7E-02	9.0E-04	7.3E-04	-8.5E-05	9.7E-04	3.1E-03	1.5E-06	5.5E-04	-5.4E-03	-1.9E-03	-5.1E-03	1.2E-02	-5.0E-03	1.3E-02	-5.0E-02	-6.0E-04	1.8E-02	-5.0E-03	-5.0E-02
101	2.5E-03	-1.2E-02	-1.4E-03	1.2E-04	2.9E-03	1.7E-03	-7.4E-03	8.2E-04	2.5E-04	-5.1E-04	-1.9E-03	1.5E-06	4.3E-04	1.8E-03	-1.4E-03	-4.2E-03	1.9E-03	-4.2E-03	-2.2E-04	1.4E-03	-1.7E-04	-4.7E-03	9.4E-04	9.3E-04
102	-2.9E-03	1.2E-02	1.4E-03	-1.5E-04	-3.1E-03	-1.0E-03	7.7E-03	-8.4E-04	-2.6E-04	4.9E-04	2.0E-03	-3.2E-07	-4.5E-04	-1.9E-03	1.5E-03	4.4E-03	1.2E-04	4.4E-03	2.1E-04	-1.5E-03	1.4E-04	4.6E-03	-8.6E-04	-1.0E-03
103	-1.4E-04	-2.0E-03	2.5E-04	-1.5E-04	1.9E-04	-6.0E-04	-1.5E-05	1.4E-04	3.8E-05	3.7E-07	1.7E-04	4.4E-06	1.3E-04	-5.5E-04	-2.1E-04	-6.7E-04	3.2E-03	-7.3E-04	2.1E-03	-7.3E-03	-1.2E-04	1.4E-03	-5.1E-04	-7.4E-03
104	1.9E-03	3.2E-02	-2.1E-03	-8.7E-02	1.5E-03	-4.0E-02	-1.3E-02	-6.9E-04	-6.6E-04	2.7E-03	7.5E-03	-1.3E-06	-1.7E-03	-8.3E-03	-4.8E-03	-2.5E-03	-3.9E-04	-2.2E-03	4.3E-04	-3.6E-03	4.2E-05	1.5E-02	6.1E-03	-2.6E-03
105	2.7E-04	7.8E-03	-5.7E-04	-2.2E-02	3.7E-04	-9.3E-03	-3.2E-03	-1.6E-04	-1.5E-04	6.3E-04	1.8E-03	-1.5E-06	-4.1E-04	-2.0E-03	-1.2E-03	-6.0E-04	9.5E-04	-5.2E-04	1.0E-04	-9.2E-04	-1.3E-05	3.5E-03	1.6E-03	-6.9E-04
106	-7.0E-02	5.6E-02	5.1E-03	2.5E-03	1.4E-02	-2.0E-01	-8.3E-02	1.5E-03	-2.6E-03	1.2E-02	3.3E-02	-8.1E-07	-5.3E-03	-4.0E-02	-2.7E-02	-2.4E-02	8.9E-04	-2.2E-02	3.4E-03	-1.2E-02	-1.3E-03	8.1E-02	-2.4E-03	-5.7E-03
107	-2.6E-04	-1.9E-04	-6.5E-05	-1.1E-05	-3.0E-05	3.9E-04	-2.1E-04	1.5E-05	9.9E-06	-1.7E-05	-4.1E-05	2.4E-08	3.2E-06	2.3E-05	-1.1E-05	1.7E-05	1.1E-03	3.0E-05	-9.0E-06	-3.9E-05	-3.4E-05	-2.7E-04	8.8E-05	-5.9E-05
108	-2.1E-04	-2.0E-04	-6.3E-05	-2.3E-05	-1.2E-05	6.3E-04	-2.0E-05	1.0E-05	7.0E-06	-3.9E-05	-1.1E-04	-2.6E-06	8.7E-06	-3.4E-05	5.5E-05	1.4E-05	1.2E-03	1.4E-05	-2.3E-05	-5.7E-05	-2.7E-05	-3.4E-04	7.2E-05	-7.9E-05
109	-9.5E-05	-9.0E-05	-3.4E-05	-5.4E-06	-4.5E-06	-1.6E-05	-1.4E-04	8.4E-06	-2.7E-07	-1.9E-05	-3.4E-06	1.5E-07	-3.6E-06	-1.4E-05	-2.8E-05	-1.2E-05	4.1E-04	-1.1E-05	-2.2E-05	-1.0E-04	-1.7E-05	-1.4E-05	3.4E-05	-1.2E-04
110	-4.7E-03	1.1E-03	-1.3E-03	-3.2E-04	-4.1E-04	9.3E-03	8.6E-04	3.4E-04	-8.8E-02	3.2E-02	-9.6E-03	-5.7E-07	1.7E-04	3.9E-02	1.8E-03	7.9E-04	4.5E-03	7.6E-04	8.8E-05	1.4E-03	-2.2E-04	1.7E-04	2.2E-03	1.1E-03
111	-1.1E-04	-4.0E-03	1.1E-03	-2.4E-04	-3.8E-04	-3.4E-03	7.5E-04	2.4E-04	-1.0E-02	-1.7E-02	1.9E-02	5.1E-06	1.7E-03	1.6E-02	1.7E-03	4.6E-04	2.3E-03	5.0E-04	4.1E-04	9.3E-04	-1.9E-04	3.8E-03	-1.9E-03	9.4E-04

112	-3.3E-04	-6.1E-04	2.8E-04	-1.1E-04	-1.3E-04	-1.8E-03	2.4E-04	7.1E-05	1.4E-04	-5.7E-03	5.8E-03	-7.4E-06	4.0E-04	2.8E-03	5.3E-04	1.5E-04	1.3E-03	1.7E-04	1.5E-04	2.0E-04	-8.4E-05	1.8E-03	-4.5E-04	2.0E-04
113	-8.1E-05	-1.6E-04	-1.3E-06	-1.9E-05	-1.7E-05	-8.0E-04	-4.0E-04	2.1E-05	8.7E-06	-7.8E-04	9.7E-04	-7.0E-07	3.3E-05	2.2E-04	-4.0E-05	5.9E-06	4.1E-04	2.3E-05	1.8E-05	-9.3E-05	-4.6E-05	4.2E-04	-1.6E-05	-9.5E-05
114	1.1E-03	-1.2E-02	1.5E-03	-2.6E-04	-4.4E-04	5.9E-03	2.9E-03	4.7E-04	-2.0E-03	-3.1E-04	1.2E-02	-2.2E-06	2.1E-03	-1.2E-02	1.2E-03	5.9E-04	7.8E-04	5.9E-04	9.2E-05	1.2E-03	-8.4E-05	-1.7E-03	-3.2E-03	1.2E-03
115	2.1E-03	-2.1E-02	2.7E-03	-4.5E-04	-7.9E-04	1.3E-02	5.0E-03	8.6E-04	-3.7E-03	4.4E-04	2.0E-02	1.6E-06	4.0E-03	-2.2E-02	2.4E-03	1.1E-03	3.6E-04	1.0E-03	1.6E-04	1.9E-03	-2.2E-04	-3.5E-03	-5.9E-03	1.7E-03
116	-3.7E-04	-3.0E-04	-9.5E-05	-1.2E-05	-6.1E-06	2.5E-04	-4.1E-04	2.5E-05	9.7E-07	-4.1E-05	1.8E-05	-1.2E-06	-6.4E-06	-1.6E-04	-5.4E-05	2.2E-05	1.7E-03	2.3E-05	-1.4E-05	-9.8E-05	-4.9E-05	-2.0E-04	1.2E-04	-1.3E-04
117	-2.7E-03	1.0E-02	-2.8E-03	2.8E-04	3.9E-04	-1.3E-02	-1.1E-02	-3.9E-04	-5.9E-04	-6.8E-03	-1.0E-02	3.0E-06	-2.1E-03	2.4E-02	6.5E-02	-5.4E-04	2.2E-03	-4.9E-04	-1.7E-04	-7.7E-04	7.2E-05	2.0E-04	5.0E-03	-7.8E-04
118	-1.9E-04	-6.3E-04	-1.3E-05	-1.8E-05	-1.4E-05	5.5E-04	-2.0E-04	3.1E-05	3.3E-05	1.4E-04	2.0E-04	2.6E-08	7.7E-05	-6.2E-04	1.0E-03	4.5E-05	7.7E-04	3.9E-05	-1.1E-05	3.7E-06	-2.7E-05	-3.7E-04	-2.9E-05	-1.3E-05
119	1.2E-03	-1.6E-02	1.2E-03	-2.1E-04	-4.2E-04	6.7E-03	-9.1E-04	6.2E-04	8.3E-04	6.0E-03	7.8E-03	3.9E-06	2.4E-03	-2.1E-02	3.5E-02	6.2E-04	-1.1E-03	5.7E-04	-8.6E-05	1.2E-03	-1.1E-04	-4.8E-03	-3.1E-03	1.1E-03
120	-1.8E-04	-7.9E-05	-4.3E-05	-1.2E-05	-1.5E-05	3.6E-04	-8.8E-05	9.4E-06	1.5E-05	-3.8E-05	-3.7E-05	-3.0E-06	-2.0E-06	9.8E-06	3.3E-05	1.1E-05	6.2E-04	1.6E-05	-5.7E-06	-3.8E-05	-2.1E-05	-1.8E-04	6.4E-05	-5.1E-05
121	-3.1E-04	-8.6E-06	-1.0E-04	-5.0E-07	1.6E-05	9.4E-04	9.4E-05	9.6E-06	1.2E-05	-9.1E-05	-1.1E-04	8.9E-08	5.0E-06	9.5E-05	-1.7E-03	6.7E-06	1.2E-03	1.0E-05	-7.1E-05	-2.4E-04	-2.0E-05	-3.8E-04	1.5E-04	-2.8E-04
122	-5.6E-03	4.4E-02	-5.3E-03	7.7E-04	1.3E-03	-3.1E-02	-1.0E-02	-1.7E-03	-2.5E-03	-2.0E-02	-2.6E-02	2.9E-06	-7.5E-03	6.9E-02	-3.3E-03	-1.9E-03	4.9E-03	-1.7E-03	-6.1E-06	-3.3E-03	2.8E-04	1.1E-02	1.2E-02	-3.1E-03
123	-1.5E-04	-2.5E-04	-4.1E-05	7.7E-06	-2.5E-05	2.8E-04	-1.7E-04	1.3E-05	4.0E-06	-1.8E-05	-3.6E-05	1.7E-06	3.4E-06	-1.1E-04	-3.0E-05	-6.0E-06	1.0E-03	7.2E-06	-2.2E-05	-7.2E-05	-2.9E-05	-2.2E-04	5.1E-05	-9.5E-05
124	-2.0E-04	-1.0E-04	-4.0E-05	8.7E-06	-8.1E-06	5.2E-04	-1.9E-05	9.5E-06	6.2E-07	-3.1E-05	-7.0E-05	1.4E-06	1.1E-05	8.5E-05	3.6E-05	2.3E-05	6.1E-04	1.9E-05	-9.4E-06	-1.2E-05	-1.8E-05	-2.6E-04	5.8E-05	-2.6E-05
125	-2.7E-04	-1.8E-04	-4.0E-05	-5.3E-06	-5.3E-05	8.5E-04	-7.1E-06	1.0E-05	2.4E-05	-6.7E-05	-1.7E-04	-4.4E-07	3.2E-05	1.5E-04	-3.3E-05	4.1E-05	1.1E-03	5.3E-05	-1.6E-05	4.2E-05	-7.0E-06	-4.5E-04	4.4E-05	1.9E-05
126	-2.6E-04	-2.6E-04	-4.9E-05	-3.4E-06	-3.7E-05	1.0E-03	1.7E-04	9.7E-06	1.8E-05	-1.0E-04	-2.0E-04	2.5E-09	3.4E-05	7.6E-05	8.7E-05	3.3E-05	1.4E-03	3.8E-05	-2.8E-05	1.7E-06	-1.0E-05	-4.9E-04	5.3E-05	-2.8E-05
127	-2.1E-04	-8.6E-04	8.8E-05	-2.5E-05	-5.4E-05	1.9E-03	7.6E-04	3.9E-05	6.0E-05	3.1E-04	4.0E-04	-7.4E-07	1.5E-04	-1.4E-03	-4.0E-03	8.0E-05	1.6E-03	8.0E-05	-1.1E-05	2.8E-05	-2.6E-05	-4.7E-04	-2.0E-04	-2.9E-06
128	-1.1E-04	-1.1E-04	-3.7E-05	-2.1E-05	-3.9E-05	2.9E-04	-7.1E-05	7.6E-06	5.8E-06	-1.6E-05	-4.2E-05	4.0E-07	-7.9E-07	4.3E-05	1.9E-05	-4.6E-06	6.5E-04	1.9E-05	-6.1E-06	-1.8E-05	-1.9E-05	-1.7E-04	5.0E-05	-3.0E-05
129	-2.6E-04	-1.3E-04	-5.9E-05	-1.3E-05	-1.6E-05	6.4E-04	-1.5E-05	1.2E-05	8.8E-06	-4.3E-05	-9.0E-05	-1.2E-06	9.5E-06	9.9E-05	5.6E-05	3.3E-05	9.6E-04	3.5E-05	-9.7E-06	-1.4E-05	-2.1E-05	-3.1E-04	7.5E-05	-3.0E-05
130	-1.6E-04	-1.0E-04	-4.1E-05	8.7E-07	-1.2E-05	4.2E-04	-8.6E-06	8.3E-06	8.5E-06	-7.4E-06	-5.9E-05	-2.4E-07	5.5E-06	1.0E-04	5.2E-05	1.2E-05	6.1E-04	1.5E-05	-1.1E-05	-3.2E-05	-1.8E-05	-2.1E-04	5.5E-05	-4.5E-05
131	-2.8E-03	2.6E-03	-5.3E-03	6.0E-04	9.8E-04	2.7E-02	-2.1E-04	-3.3E-04	3.8E-03	-2.5E-02	3.0E-02	1.9E-06	-4.3E-03	1.4E-02	-1.2E-03	-1.0E-03	-3.1E-03	-1.2E-03	-2.0E-03	-2.6E-03	3.7E-04	-1.8E-02	7.8E-03	-3.2E-03
132	3.8E-03	-2.8E-02	1.9E-02	-3.3E-03	-5.1E-03	-2.7E-03	1.9E-02	4.4E-03	-4.3E-03	3.7E-02	9.4E-02	-4.7E-06	-8.6E-02	-9.5E-03	9.6E-03	6.7E-03	1.3E-02	6.7E-03	3.8E-03	1.3E-02	-2.0E-03	2.6E-02	-2.9E-02	1.3E-02
133	-1.8E-03	2.0E-02	4.1E-03	-7.1E-04	-1.0E-03	-4.3E-03	5.4E-04	4.5E-04	1.2E-02	3.1E-02	-5.9E-03	-8.1E-02	1.2E-04	3.1E-02	1.0E-03	1.3E-03	6.4E-03	1.4E-03	8.0E-04	2.1E-03	-3.7E-04	8.7E-03	-3.9E-03	1.9E-03

134	-6.8E-04	5.2E-03	1.0E-03	-2.0E-04	-3.0E-04	-4.0E-04	3.4E-04	1.3E-04	3.3E-03	8.1E-03	-1.6E-03	-2.2E-02	4.8E-05	8.3E-03	4.6E-04	3.8E-04	2.7E-03	4.0E-04	2.1E-04	5.9E-04	-1.2E-04	2.1E-03	-9.8E-04	5.0E-04
135	-1.0E-04	-1.2E-04	-5.3E-05	-3.2E-06	-6.4E-06	-4.1E-04	-4.8E-04	1.2E-05	-1.4E-05	-2.0E-03	5.0E-05	8.2E-07	-2.8E-05	-1.6E-04	-1.2E-04	-2.1E-05	5.8E-04	-5.1E-06	-5.0E-06	-1.1E-04	-3.9E-05	6.8E-05	7.2E-05	-1.2E-04
136	-2.0E-03	1.1E-02	-3.6E-03	4.8E-04	7.9E-04	1.3E-02	-2.1E-03	-4.8E-04	-9.7E-03	6.0E-02	-5.6E-02	2.6E-06	-4.6E-03	-2.6E-02	-3.6E-03	-9.1E-04	-1.8E-03	-9.6E-04	-1.1E-03	-2.1E-03	3.3E-04	-1.0E-02	6.1E-03	-2.3E-03
137	1.5E-02	-4.4E-03	-1.6E-03	2.6E-03	3.5E-02	3.4E-02	2.2E-02	-3.6E-04	2.5E-04	-1.5E-03	-3.6E-03	1.3E-06	5.6E-04	4.4E-03	6.2E-03	5.3E-02	9.5E-03	-4.8E-02	2.5E-05	4.3E-03	1.1E-04	-5.2E-03	1.9E-03	2.4E-03
138	-2.4E-03	2.4E-03	7.7E-05	-1.3E-04	2.8E-03	-4.9E-03	-2.4E-03	4.9E-05	-5.3E-05	3.1E-04	9.4E-04	4.1E-07	-1.6E-04	-1.1E-03	-8.0E-04	2.2E-03	1.4E-03	2.8E-03	1.4E-04	-2.9E-04	-6.6E-05	2.2E-03	1.1E-04	-1.4E-04
139	-1.2E-02	8.6E-04	1.3E-03	-2.5E-03	-3.8E-02	-2.4E-02	-1.9E-02	3.6E-04	-1.0E-04	8.4E-04	1.7E-03	-5.1E-06	-2.6E-04	-2.5E-03	-4.9E-03	-5.5E-02	-7.6E-03	4.6E-02	-2.6E-04	-3.8E-03	-6.7E-05	9.9E-04	-1.8E-03	-2.1E-03
140	1.3E-02	-1.8E-03	-1.4E-03	2.5E-03	3.7E-02	2.8E-02	1.9E-02	-3.2E-04	1.7E-04	-1.1E-03	-2.4E-03	8.6E-07	3.6E-04	3.1E-03	5.3E-03	5.5E-02	9.4E-03	-4.6E-02	1.8E-04	3.9E-03	3.2E-05	-2.5E-03	2.0E-03	2.2E-03
141	-1.3E-02	7.6E-04	1.2E-03	-2.5E-03	-3.8E-02	-2.4E-02	-1.9E-02	3.6E-04	-9.5E-05	8.5E-04	1.7E-03	2.0E-06	-2.5E-04	-2.6E-03	-4.9E-03	-5.5E-02	-6.9E-03	4.6E-02	-2.6E-04	-3.8E-03	-6.8E-05	8.7E-04	-1.8E-03	-2.1E-03
142	-2.1E-04	-2.1E-04	-5.0E-05	-1.7E-05	-2.4E-05	2.6E-04	-2.9E-04	1.3E-05	9.0E-06	-4.1E-05	-2.5E-05	-3.1E-07	3.2E-06	-1.1E-04	-8.3E-05	3.0E-05	1.2E-03	3.9E-05	7.0E-07	-6.0E-06	-3.0E-05	-2.3E-04	6.6E-05	-2.0E-05
143	3.6E-04	-8.9E-04	-8.3E-05	-2.4E-06	7.0E-05	-8.2E-04	-6.6E-04	2.6E-05	3.9E-07	2.4E-05	8.4E-05	2.2E-06	-2.0E-05	-7.1E-05	-7.2E-05	6.1E-05	9.2E-04	1.1E-01	6.1E-06	1.6E-06	-3.7E-05	8.3E-05	3.1E-05	6.1E-06
144	-2.7E-04	-1.4E-04	-7.6E-05	-5.6E-06	-1.4E-05	6.8E-04	-2.2E-05	1.1E-05	-1.2E-06	-7.2E-05	-1.0E-04	2.7E-06	1.3E-05	1.1E-04	6.6E-05	2.8E-05	9.7E-04	-4.7E-03	-1.1E-05	-1.9E-05	-2.5E-05	-3.4E-04	8.7E-05	-3.7E-05
145	-2.6E-04	-1.4E-04	-4.1E-05	-1.0E-05	-2.1E-05	4.8E-04	-1.7E-04	1.1E-05	1.5E-05	-4.6E-05	-8.1E-05	1.5E-06	1.6E-05	6.0E-05	-5.9E-05	3.7E-05	1.0E-03	8.1E-06	-1.2E-05	-1.1E-05	-2.0E-05	-3.2E-04	5.5E-05	-3.0E-05
146	-1.9E-04	2.0E-05	-3.5E-05	1.3E-06	-1.3E-06	3.7E-04	6.8E-05	5.4E-06	2.3E-06	-2.9E-05	-5.2E-05	1.3E-06	7.0E-06	8.0E-05	7.4E-05	2.0E-05	4.7E-04	-5.6E-04	-1.0E-05	-2.7E-05	-1.5E-05	-1.7E-04	5.6E-05	-3.6E-05
147	-1.4E-03	-7.5E-03	-1.7E-03	4.0E-05	8.6E-06	9.5E-02	7.8E-02	2.6E-04	4.9E-04	-9.7E-04	-3.1E-03	3.9E-08	6.1E-04	3.0E-03	-3.3E-03	1.6E-04	-4.1E-04	1.3E-04	-3.1E-04	8.0E-04	3.9E-06	-8.0E-03	2.0E-03	6.8E-04
148	-1.7E-04	-9.8E-05	-6.3E-05	-1.3E-05	-1.8E-05	1.1E-03	3.3E-04	1.1E-05	6.8E-06	-4.2E-05	-7.7E-05	-1.2E-06	-6.0E-06	1.0E-04	2.3E-04	1.5E-06	5.4E-04	1.3E-05	-5.7E-06	-1.7E-05	-2.2E-05	-2.7E-04	9.9E-05	-2.9E-05
149	-2.8E-04	-1.9E-04	-5.3E-05	-1.5E-05	-4.1E-05	8.2E-04	9.7E-06	1.3E-05	1.5E-05	-8.0E-05	-1.3E-04	2.1E-07	2.4E-05	1.7E-04	4.2E-05	3.9E-05	1.1E-03	4.7E-05	-1.8E-05	-6.7E-07	-1.6E-05	-4.1E-04	6.9E-05	-2.5E-05
150	-9.0E-05	4.3E-04	5.5E-05	-2.0E-05	-2.7E-05	5.3E-02	-5.5E-02	3.9E-05	-4.0E-05	1.3E-04	3.8E-04	-4.3E-07	-3.5E-05	-4.9E-04	-1.1E-04	6.5E-07	5.4E-04	1.5E-05	3.0E-05	-8.9E-05	-4.9E-05	8.7E-04	-4.6E-05	-9.4E-05
151	-1.8E-04	-6.7E-04	-1.5E-04	-8.8E-06	1.4E-05	-5.2E-02	5.3E-02	-1.6E-05	3.1E-05	-1.1E-04	-2.4E-04	9.4E-07	-6.4E-07	3.4E-04	1.2E-04	6.0E-06	7.0E-04	1.5E-05	-4.3E-05	-8.7E-05	-1.9E-05	-8.8E-04	1.8E-04	-1.0E-04
152	-8.3E-05	-9.0E-05	-4.2E-05	-3.2E-06	-2.4E-05	2.5E-04	-1.9E-04	8.4E-06	1.1E-06	-1.0E-05	-1.9E-07	1.1E-06	-2.8E-06	-3.7E-05	-2.3E-05	-1.6E-05	4.2E-04	-2.9E-06	-3.5E-06	-3.8E-05	-2.7E-05	-9.2E-05	5.4E-05	-4.9E-05
153	-3.8E-05	-2.6E-05	-1.6E-05	-1.3E-05	-9.6E-06	1.9E-04	-9.0E-05	6.8E-07	2.7E-06	-1.9E-05	-4.0E-05	-7.5E-07	2.0E-06	4.6E-05	4.8E-05	1.0E-05	2.4E-04	1.6E-05	-3.2E-06	2.5E-06	-4.1E-07	-6.7E-05	1.4E-05	-1.1E-06
154	-2.8E-04	3.2E-04	-2.5E-04	3.3E-06	2.3E-04	-1.3E-02	5.3E-04	-6.0E-06	1.9E-05	-1.4E-04	-2.3E-04	-7.2E-08	-1.1E-05	3.3E-04	3.2E-04	-1.6E-04	1.5E-03	-1.7E-04	-3.6E-04	-1.2E-03	-8.3E-06	-8.5E-04	4.0E-04	-1.3E-03
155	-2.6E-04	-1.6E-04	-6.3E-	-7.7E-06	-2.1E-05	6.3E-04	-2.0E-05	1.2E-05	8.3E-06	-7.3E-05	-9.0E-05	4.5E-07	1.3E-05	8.1E-05	5.2E-05	3.6E-05	1.0E-03	4.3E-05	-6.8E-05	2.3E-06	-2.2E-05	-3.2E-04	7.7E-05	-2.3E-05

			05																					
156	-1.4E-03	1.7E-03	4.6E-05	-8.2E-05	3.0E-04	-1.6E-04	-1.0E-03	-1.9E-05	-5.5E-05	1.4E-04	5.0E-04	-2.4E-06	-1.0E-04	-6.6E-04	-2.2E-04	1.6E-03	5.0E-04	1.7E-03	-1.8E-02	3.4E-03	-3.3E-05	8.2E-04	8.4E-05	7.9E-04
157	-2.1E-04	-3.6E-04	-1.1E-06	-1.4E-05	-1.2E-04	4.0E-03	-4.3E-06	1.8E-05	5.4E-06	-1.7E-05	-9.7E-05	-1.8E-06	3.2E-05	-7.0E-05	-2.5E-05	-1.9E-05	1.2E-03	-1.6E-05	1.2E-03	8.7E-05	-1.3E-05	-1.5E-04	-3.2E-05	2.5E-04
158	-4.9E-03	1.7E-03	6.8E-04	-3.3E-04	2.6E-04	-1.6E-02	-4.6E-03	2.5E-04	-1.3E-04	8.5E-04	2.3E-03	-1.8E-09	-1.6E-04	-3.3E-03	-2.1E-03	3.0E-03	3.9E-03	3.7E-03	-8.4E-02	2.5E-03	-2.0E-04	8.0E-03	-9.3E-04	-1.0E-02
159	-2.4E-04	-1.3E-04	-4.5E-05	1.9E-06	-8.0E-06	6.1E-04	-2.0E-05	1.0E-05	1.4E-05	-1.7E-05	-8.8E-05	-8.4E-07	1.5E-05	1.9E-05	2.3E-05	3.1E-05	8.6E-04	2.5E-05	-7.5E-06	6.9E-06	-1.7E-05	-3.1E-04	6.0E-05	-8.8E-06
160	-1.1E-04	-7.8E-05	-5.2E-05	-6.4E-06	1.4E-05	-2.7E-04	-3.4E-04	1.1E-05	-5.4E-06	-7.1E-06	9.9E-05	-5.7E-07	-2.5E-05	-8.7E-05	-3.6E-05	-2.7E-06	3.9E-04	-3.7E-06	1.6E-07	-9.7E-05	-3.7E-05	5.4E-05	7.1E-05	-1.0E-04
161	-2.1E-04	-9.6E-05	-3.4E-05	-6.1E-06	-1.3E-05	3.7E-04	-1.7E-04	9.6E-06	1.1E-05	-5.9E-06	-4.1E-05	2.2E-06	1.1E-05	3.9E-05	-4.2E-05	3.1E-05	7.5E-04	3.0E-05	-5.9E-06	-1.1E-05	-2.1E-05	-2.6E-04	5.3E-05	-2.4E-05
162	-1.3E-04	-1.1E-04	-6.0E-05	4.0E-07	2.1E-05	-6.9E-04	-6.9E-04	1.4E-05	-1.2E-05	1.1E-05	1.7E-04	-1.0E-06	-3.0E-05	-2.3E-04	-2.0E-04	-1.3E-05	5.3E-04	-1.3E-05	3.8E-06	-1.3E-04	-5.4E-05	1.1E-04	7.8E-05	-1.3E-04
163	-1.5E-04	-1.1E-04	-3.9E-05	1.8E-06	-4.1E-05	3.2E-04	-1.3E-04	8.0E-06	3.3E-07	-3.8E-05	-4.9E-05	7.4E-06	5.0E-06	-2.6E-05	-2.4E-05	1.3E-05	7.2E-04	3.4E-05	-1.7E-06	5.3E-06	-1.9E-05	-2.1E-04	5.1E-05	-7.8E-06
164	-2.7E-04	-1.7E-04	-5.5E-05	-1.2E-05	-2.7E-05	6.0E-04	-8.7E-05	1.2E-05	1.1E-05	-7.2E-05	-9.5E-05	5.0E-07	1.8E-05	1.2E-04	-4.0E-06	4.1E-05	1.1E-03	4.6E-05	-1.2E-05	-9.6E-06	-1.9E-05	-3.3E-04	6.6E-05	-2.8E-05
165	-1.5E-04	-1.2E-04	-4.8E-05	-4.2E-06	-2.6E-05	2.3E-04	-1.1E-04	8.7E-06	6.1E-07	-3.0E-05	-3.5E-05	3.1E-06	6.2E-06	4.9E-05	-6.9E-07	8.4E-06	7.2E-04	2.2E-05	-7.7E-06	-3.3E-05	-2.0E-05	-1.4E-04	5.5E-05	-4.6E-05
166	-1.5E-04	-1.6E-04	-4.8E-05	-6.0E-06	-1.5E-05	-5.7E-05	-2.8E-04	1.1E-05	-3.8E-06	-1.4E-05	2.1E-05	2.7E-07	-2.6E-06	3.8E-06	-8.3E-05	1.2E-05	9.2E-04	2.2E-05	-6.6E-06	-5.4E-05	-2.6E-05	-6.9E-05	5.0E-05	-6.6E-05
167	-2.0E-04	-9.1E-05	-6.4E-05	-4.1E-06	-1.6E-05	6.7E-04	1.0E-04	7.2E-06	-6.6E-06	-5.3E-05	-9.1E-05	3.3E-06	4.1E-06	1.1E-04	1.4E-04	2.8E-05	7.3E-04	3.0E-05	1.3E-06	1.8E-05	-2.0E-05	-2.8E-04	8.2E-05	6.5E-06
168	-1.5E-04	-1.3E-04	-4.6E-05	-9.8E-06	-2.0E-05	3.3E-04	-2.9E-05	9.7E-06	2.7E-06	6.0E-06	-2.6E-05	1.8E-07	-2.3E-06	6.2E-05	7.2E-05	5.8E-06	6.5E-04	1.6E-05	-1.8E-05	-7.9E-05	-2.4E-05	-1.5E-04	6.2E-05	-9.5E-05
169	-1.7E-04	-1.3E-04	-4.6E-05	-7.7E-06	-8.2E-06	2.2E-04	-1.2E-04	9.4E-06	-1.3E-06	-3.2E-05	-2.4E-05	1.4E-06	4.2E-07	-2.6E-06	1.3E-05	2.0E-05	7.8E-04	2.4E-05	-9.2E-06	-4.9E-05	-2.3E-05	-1.4E-04	6.0E-05	-6.1E-05
170	-1.4E-04	-1.4E-04	-5.0E-05	-8.4E-06	-4.7E-05	3.6E-04	-6.8E-05	9.8E-06	7.7E-06	-3.0E-05	-3.7E-05	-5.3E-07	3.9E-06	2.2E-05	3.7E-05	5.5E-06	8.7E-04	2.8E-05	-8.3E-06	-2.0E-05	-2.1E-05	-1.9E-04	6.3E-05	-3.7E-05
171	-3.0E-04	-2.7E-04	-7.7E-05	-2.1E-05	-4.6E-05	1.2E-03	2.5E-04	1.5E-05	2.2E-05	-1.1E-04	-1.7E-04	-5.2E-07	1.7E-05	8.8E-05	2.2E-04	2.9E-05	1.6E-03	4.6E-05	-1.8E-05	4.7E-06	-2.0E-05	-4.5E-04	1.0E-04	-2.3E-05
172	-1.3E-04	-1.2E-04	-4.4E-05	-6.6E-06	-7.6E-06	5.1E-05	-2.5E-04	9.5E-06	4.5E-06	-2.1E-05	8.1E-06	7.7E-07	-4.9E-06	-3.2E-05	-6.3E-05	2.2E-06	6.4E-04	6.9E-06	-2.4E-06	-3.2E-05	-2.6E-05	-1.0E-04	5.5E-05	-4.3E-05
173	-3.0E-04	-1.8E-04	-7.4E-05	-9.3E-06	-2.8E-05	8.0E-04	9.6E-05	1.5E-05	9.9E-06	-5.3E-05	-8.9E-05	1.6E-06	1.0E-05	5.8E-05	1.5E-04	3.1E-05	1.2E-03	3.9E-05	-1.5E-05	-3.3E-05	-2.2E-05	-3.2E-04	9.3E-05	-5.6E-05
174	-1.2E-04	-4.8E-05	-3.6E-05	-3.2E-06	-1.5E-06	2.6E-04	-5.8E-05	7.6E-06	1.1E-06	-1.7E-05	-6.5E-06	1.7E-06	-3.7E-06	8.0E-06	4.3E-05	-7.1E-08	2.3E-04	-4.7E-07	-1.1E-05	-5.9E-05	-2.1E-05	-1.4E-04	5.3E-05	-6.8E-05
175	-1.8E-04	-1.3E-04	-5.1E-05	-1.7E-05	-7.6E-06	3.6E-04	-1.1E-04	1.1E-05	9.7E-06	-1.5E-05	-3.8E-05	1.5E-06	-2.7E-07	-8.9E-06	3.3E-05	8.4E-06	7.2E-04	9.9E-06	-8.9E-06	-4.6E-05	-2.8E-05	-2.0E-04	7.4E-05	-6.0E-05

176	-2.7E-04	-2.1E-04	-5.7E-05	-1.2E-05	-3.5E-05	8.1E-04	1.0E-05	1.3E-05	1.4E-05	-4.8E-05	-1.3E-04	-1.5E-06	2.1E-05	1.3E-04	5.7E-05	2.7E-05	1.1E-03	3.4E-05	-2.6E-05	-4.1E-05	-2.2E-05	-4.0E-04	7.1E-05	-6.6E-05
177	-1.8E-04	-1.5E-04	-5.6E-05	-5.2E-06	-2.0E-05	4.2E-04	-1.3E-05	9.6E-06	2.6E-06	-4.6E-05	-5.4E-05	2.0E-07	-2.0E-06	7.5E-05	8.0E-05	2.1E-05	8.7E-04	2.9E-05	-6.3E-06	-3.0E-05	-2.3E-05	-1.9E-04	7.0E-05	-4.3E-05
178	-2.6E-04	-1.5E-04	-4.4E-05	-1.9E-05	-5.5E-05	1.2E-03	2.7E-04	7.8E-06	2.1E-05	-1.0E-04	-2.3E-04	-1.9E-07	4.0E-05	2.2E-04	1.1E-04	4.6E-05	1.1E-03	5.7E-05	-1.7E-05	6.6E-05	2.1E-06	-5.3E-04	4.8E-05	4.2E-05
179	-4.7E-05	-2.6E-05	-9.6E-06	-8.1E-06	-1.0E-05	2.5E-04	6.1E-05	8.5E-07	8.6E-06	-7.0E-07	-6.6E-05	1.7E-06	6.6E-06	6.8E-05	1.8E-05	1.3E-05	2.0E-04	1.8E-05	-5.2E-06	5.4E-06	8.6E-07	-1.1E-04	8.5E-06	8.1E-07
180	0.0E+00	0.0E+00	0.0E+00	0.0E+00	0.0E+00	0.0E+00	0.0E+00	0.0E+00	0.0E+00	0.0E+00	0.0E+00	0.0E+00	0.0E+00	0.0E+00	0.0E+00	0.0E+00	0.0E+00	0.0E+00	0.0E+00	0.0E+00	0.0E+00	0.0E+00	0.0E+00	0.0E+00
181	0.0E+00	0.0E+00	0.0E+00	0.0E+00	0.0E+00	0.0E+00	0.0E+00	0.0E+00	0.0E+00	0.0E+00	0.0E+00	0.0E+00	0.0E+00	0.0E+00	0.0E+00	0.0E+00	0.0E+00	0.0E+00	0.0E+00	0.0E+00	0.0E+00	0.0E+00	0.0E+00	0.0E+00
182	-1.1E-04	-6.6E-05	-1.4E-05	-8.1E-06	-5.0E-06	9.7E-05	-2.0E-04	4.1E-06	8.6E-06	1.0E-05	-3.7E-05	7.7E-08	5.6E-06	-4.4E-06	-9.8E-05	3.6E-05	4.9E-04	3.4E-05	1.1E-05	4.4E-05	-1.2E-05	-1.6E-04	2.4E-05	4.3E-05
183	-6.8E-05	-9.3E-05	-4.0E-05	-3.0E-07	-1.4E-05	-1.7E-04	-2.8E-04	8.4E-06	-1.8E-06	-9.5E-06	5.4E-05	2.2E-06	-1.8E-05	-1.2E-04	-4.2E-05	-6.4E-06	4.8E-04	8.4E-06	3.3E-06	-5.2E-05	-2.8E-05	1.9E-05	5.7E-05	-5.7E-05
184	4.9E-05	-1.8E-04	2.2E-05	-1.9E-06	-2.9E-05	-8.7E-04	-6.0E-04	9.3E-06	-2.8E-06	3.4E-05	1.6E-04	-8.1E-07	-1.8E-05	-2.3E-04	1.6E-03	1.2E-05	-1.5E-04	1.6E-05	6.1E-05	1.2E-04	-3.2E-05	2.0E-04	-4.4E-05	1.4E-04
185	-1.0E-04	-1.2E-04	-4.2E-05	-5.4E-06	2.6E-06	-1.7E-04	-3.0E-04	1.0E-05	-6.5E-06	7.8E-06	5.2E-05	5.1E-07	-1.4E-05	-8.7E-05	-5.6E-05	-1.5E-05	5.3E-04	-1.3E-05	-1.3E-05	-1.1E-04	-3.1E-05	6.4E-06	5.2E-05	-1.2E-04
186	-1.8E-04	-1.0E-04	-5.3E-05	-1.5E-05	-1.2E-05	5.0E-04	3.0E-05	8.0E-06	4.3E-06	-2.8E-05	-7.4E-05	-3.3E-07	1.0E-05	6.7E-05	6.6E-05	2.2E-05	6.9E-04	2.1E-05	-9.9E-06	-1.5E-05	-1.5E-05	-2.3E-04	6.0E-05	-2.9E-05
187	-1.5E-04	-8.8E-05	-3.3E-05	-2.2E-05	-2.4E-05	3.0E-04	-3.3E-05	7.5E-06	4.1E-09	-2.3E-05	-5.6E-05	-4.2E-06	-2.0E-06	7.8E-06	2.8E-05	1.9E-05	6.7E-04	2.9E-05	-9.1E-06	-2.6E-05	-1.3E-05	-1.5E-04	4.5E-05	-3.8E-05
188	0.0E+00	0.0E+00	0.0E+00	0.0E+00	0.0E+00	0.0E+00	0.0E+00	0.0E+00	0.0E+00	0.0E+00	0.0E+00	0.0E+00	0.0E+00	0.0E+00	0.0E+00	0.0E+00	0.0E+00	0.0E+00	0.0E+00	0.0E+00	0.0E+00	0.0E+00	0.0E+00	0.0E+00
189	-2.9E-04	-2.2E-04	-6.2E-05	-9.0E-06	-2.3E-05	7.9E-04	1.1E-05	1.3E-05	1.9E-05	-8.1E-05	-1.3E-04	1.1E-06	2.1E-05	1.0E-04	6.2E-05	3.8E-05	1.3E-03	4.1E-05	-1.5E-05	-8.6E-06	-2.1E-05	-3.9E-04	8.0E-05	-3.0E-05
190	0.0E+00	0.0E+00	0.0E+00	0.0E+00	0.0E+00	0.0E+00	0.0E+00	0.0E+00	0.0E+00	0.0E+00	0.0E+00	0.0E+00	0.0E+00	0.0E+00	0.0E+00	0.0E+00	0.0E+00	0.0E+00	0.0E+00	0.0E+00	0.0E+00	0.0E+00	0.0E+00	0.0E+00
191	-2.6E-04	-1.7E-04	-5.0E-05	-1.3E-05	-3.2E-05	8.0E-04	3.6E-05	1.2E-05	2.2E-05	-7.6E-05	-1.5E-04	3.3E-07	1.8E-05	1.1E-04	5.4E-05	3.3E-05	1.1E-03	4.0E-05	-1.5E-05	2.5E-06	-1.6E-05	-3.9E-04	6.9E-05	-1.8E-05
192	-3.0E-04	-2.4E-04	-6.4E-05	-1.6E-05	-3.0E-05	6.2E-04	-1.2E-04	1.5E-05	1.2E-05	-4.4E-05	-9.4E-05	-8.8E-07	2.0E-05	8.6E-05	-1.2E-05	4.0E-05	1.4E-03	4.6E-05	-1.4E-05	-1.4E-05	-2.4E-05	-3.5E-04	7.5E-05	-3.6E-05
193	-1.1E-04	-9.5E-05	-5.0E-05	-3.1E-06	-2.9E-05	2.2E-04	-9.6E-05	8.6E-06	2.4E-06	-3.0E-05	-1.1E-05	-3.8E-07	-5.1E-06	-3.7E-05	3.1E-05	-1.9E-06	5.9E-04	1.5E-05	-6.4E-06	-4.7E-05	-2.5E-05	-1.2E-04	6.4E-05	-6.0E-05
194	-2.4E-04	-1.1E-04	-4.9E-05	-1.9E-05	-1.6E-05	6.3E-04	-2.6E-05	1.1E-05	1.5E-05	-5.3E-05	-9.2E-05	-3.0E-06	9.2E-06	-5.5E-05	4.1E-05	3.2E-05	9.5E-04	3.3E-05	-1.3E-05	-2.2E-05	-1.8E-05	-3.0E-04	6.9E-05	-3.7E-05
195	-1.8E-04	-1.0E-04	-5.3E-05	-1.5E-05	-1.2E-05	5.0E-04	3.0E-05	8.0E-06	4.3E-06	-2.8E-05	-7.4E-05	-3.3E-07	1.0E-05	6.7E-05	6.6E-05	2.2E-05	6.9E-04	2.1E-05	-9.9E-06	-1.5E-05	-1.5E-05	-2.3E-04	6.0E-05	-2.9E-05
196	0.0E+00	0.0E+00	0.0E+00	0.0E+00	0.0E+00	0.0E+00	0.0E+00	0.0E+00	0.0E+00	0.0E+00	0.0E+00	0.0E+00	0.0E+00	0.0E+00	0.0E+00	0.0E+00	0.0E+00	0.0E+00	0.0E+00	0.0E+00	0.0E+00	0.0E+00	0.0E+00	0.0E+00
197	-2.7E-04	1.1E-04	-6.1E-05	-1.0E-05	-8.5E-06	-1.8E-04	-2.1E-04	1.2E-05	-1.2E-05	-9.0E-06	1.0E-04	-8.0E-07	-1.9E-05	-1.6E-04	-4.6E-05	3.6E-05	8.1E-04	3.3E-05	2.6E-05	1.9E-05	-3.1E-05	1.7E-04	8.4E-05	6.5E-06

198	0.0E+00	0.0E+00	0.0E+00	0.0E+00	0.0E+00	0.0E+00	0.0E+00	0.0E+00	0.0E+00	0.0E+00	0.0E+00	0.0E+00	0.0E+00	0.0E+00	0.0E+00	0.0E+00	0.0E+00	0.0E+00	0.0E+00	0.0E+00	0.0E+00	0.0E+00	0.0E+00	0.0E+00
199	0.0E+00	0.0E+00	0.0E+00	0.0E+00	0.0E+00	0.0E+00	0.0E+00	0.0E+00	0.0E+00	0.0E+00	0.0E+00	0.0E+00	0.0E+00	0.0E+00	0.0E+00	0.0E+00	0.0E+00	0.0E+00	0.0E+00	0.0E+00	0.0E+00	0.0E+00	0.0E+00	0.0E+00
200	0.0E+00	0.0E+00	0.0E+00	0.0E+00	0.0E+00	0.0E+00	0.0E+00	0.0E+00	0.0E+00	0.0E+00	0.0E+00	0.0E+00	0.0E+00	0.0E+00	0.0E+00	0.0E+00	0.0E+00	0.0E+00	0.0E+00	0.0E+00	0.0E+00	0.0E+00	0.0E+00	0.0E+00
201	0.0E+00	0.0E+00	0.0E+00	0.0E+00	0.0E+00	0.0E+00	0.0E+00	0.0E+00	0.0E+00	0.0E+00	0.0E+00	0.0E+00	0.0E+00	0.0E+00	0.0E+00	0.0E+00	0.0E+00	0.0E+00	0.0E+00	0.0E+00	0.0E+00	0.0E+00	0.0E+00	0.0E+00
202	0.0E+00	0.0E+00	0.0E+00	0.0E+00	0.0E+00	0.0E+00	0.0E+00	0.0E+00	0.0E+00	0.0E+00	0.0E+00	0.0E+00	0.0E+00	0.0E+00	0.0E+00	0.0E+00	0.0E+00	0.0E+00	0.0E+00	0.0E+00	0.0E+00	0.0E+00	0.0E+00	0.0E+00
203	0.0E+00	0.0E+00	0.0E+00	0.0E+00	0.0E+00	0.0E+00	0.0E+00	0.0E+00	0.0E+00	0.0E+00	0.0E+00	0.0E+00	0.0E+00	0.0E+00	0.0E+00	0.0E+00	0.0E+00	0.0E+00	0.0E+00	0.0E+00	0.0E+00	0.0E+00	0.0E+00	0.0E+00
204	0.0E+00	0.0E+00	0.0E+00	0.0E+00	0.0E+00	0.0E+00	0.0E+00	0.0E+00	0.0E+00	0.0E+00	0.0E+00	0.0E+00	0.0E+00	0.0E+00	0.0E+00	0.0E+00	0.0E+00	0.0E+00	0.0E+00	0.0E+00	0.0E+00	0.0E+00	0.0E+00	0.0E+00
205	0.0E+00	0.0E+00	0.0E+00	0.0E+00	0.0E+00	0.0E+00	0.0E+00	0.0E+00	0.0E+00	0.0E+00	0.0E+00	0.0E+00	0.0E+00	0.0E+00	0.0E+00	0.0E+00	0.0E+00	0.0E+00	0.0E+00	0.0E+00	0.0E+00	0.0E+00	0.0E+00	0.0E+00
206	0.0E+00	0.0E+00	0.0E+00	0.0E+00	0.0E+00	0.0E+00	0.0E+00	0.0E+00	0.0E+00	0.0E+00	0.0E+00	0.0E+00	0.0E+00	0.0E+00	0.0E+00	0.0E+00	0.0E+00	0.0E+00	0.0E+00	0.0E+00	0.0E+00	0.0E+00	0.0E+00	0.0E+00
207	-2.5E-04	-1.3E-04	-3.8E-05	-1.0E-05	-2.4E-05	7.6E-04	9.0E-06	1.1E-05	2.1E-05	-7.3E-05	-1.3E-04	4.9E-07	1.6E-05	1.0E-04	3.9E-05	3.3E-05	9.2E-04	3.6E-05	-1.7E-05	-1.5E-05	-1.5E-05	-3.7E-04	5.9E-05	-3.5E-05
208	-1.2E-04	-1.3E-04	-2.6E-05	-1.1E-05	-3.8E-05	2.8E-04	-1.6E-04	7.4E-06	1.0E-05	-4.4E-05	-5.0E-05	3.4E-06	1.5E-05	5.9E-05	-7.2E-05	1.4E-07	6.4E-04	1.6E-05	-1.4E-05	-2.3E-05	-1.7E-05	-2.3E-04	3.4E-05	-4.1E-05
209	-2.2E-04	-1.4E-04	-5.0E-05	-1.8E-05	-3.7E-05	8.8E-04	1.8E-04	9.6E-06	1.2E-05	-2.8E-05	-1.5E-04	-8.5E-07	1.3E-05	1.4E-04	1.4E-04	4.0E-05	9.2E-04	4.9E-05	-8.2E-06	2.5E-05	-9.9E-06	-3.5E-04	6.8E-05	7.9E-06

Relative MDE values

#	glucose exchange flux	lactate exchange flux	pyruvate exchange flux	glycerol exchange flux	ffa uptake	acetoacetate flux	ß-hydroxy-buturate flux	oxygen consumption rate	nh3 uptake	Gln exchange flux	Glu exchange flux	Ser exchange flux	Ala exchange flux	urea production	acetate flux	vldl export	Glycogen content	TAG content	cholesterol synthesis	fatty acid synthesis	Mitochondrial membrane potential	ATP/ADP	NAD/NADH	NADP/NADPH

1	0.12	-0.02	-0.02	0.09	-1.00	0.18	0.17	-0.01	0.04	-0.11	-0.06	0.56	0.08	0.07	0.14	-0.55	0.05	-0.57	0.00	0.15	0.00	-0.04	0.03	0.02
2	0.02	0.00	0.00	0.02	-0.18	0.03	0.03	0.00	0.01	-0.02	-0.01	0.25	0.01	0.01	0.02	-0.10	0.01	-0.10	0.00	0.02	0.00	-0.01	0.01	0.00
3	0.09	-0.02	-0.02	0.06	-0.70	0.11	0.11	0.00	0.02	-0.07	-0.04	0.00	0.05	0.04	0.10	-0.39	0.04	-0.42	0.00	0.11	0.00	-0.02	0.02	0.02
4	0.10	-0.02	-0.02	0.07	-0.77	0.13	0.13	0.00	0.03	-0.08	-0.04	0.27	0.05	0.05	0.11	-0.43	0.04	-0.47	0.00	0.12	0.00	-0.02	0.03	0.02
5	0.01	0.00	0.00	0.01	-0.12	0.02	0.02	0.00	0.00	-0.01	-0.01	0.10	0.01	0.01	0.02	-0.07	0.01	-0.07	0.00	0.02	0.00	0.00	0.01	0.00
6	-0.10	0.39	0.15	-0.01	-0.31	-0.03	0.19	-0.06	-0.02	0.07	0.13	0.00	-0.03	-0.12	0.07	0.34	-0.01	0.16	0.08	-0.09	0.02	0.15	-0.05	-0.06
7	0.00	0.00	0.00	0.00	0.00	0.00	0.00	0.00	0.00	0.00	0.00	0.00	0.00	0.00	0.00	0.00	0.00	0.00	0.00	0.00	0.00	0.00	0.00	0.00
8	0.00	0.00	0.00	0.00	0.00	0.00	0.00	0.00	0.00	0.00	0.00	0.33	0.00	0.00	0.00	0.00	0.00	0.00	0.00	0.00	0.00	0.00	0.00	0.00
9	0.00	0.00	0.00	0.00	0.00	0.00	0.00	0.00	0.00	0.00	0.00	0.00	0.00	0.00	0.00	0.00	0.00	0.00	0.00	0.00	0.00	0.00	0.00	0.00
10	0.00	0.00	0.00	0.00	0.00	0.00	0.00	0.00	0.00	0.00	0.00	0.00	0.00	0.00	0.00	0.00	0.00	0.00	0.00	0.00	0.00	0.00	0.00	0.00
11	0.00	0.00	0.00	0.00	0.00	0.00	0.00	0.01	0.00	0.00	0.00	0.20	0.01	0.00	0.00	0.00	0.01	0.00	0.00	0.00	0.00	0.00	0.00	0.00
12	0.00	0.00	0.00	0.00	0.00	0.00	0.00	0.00	0.00	0.00	0.00	0.00	0.00	0.00	0.00	0.00	0.00	0.00	0.00	0.00	0.00	0.00	0.00	0.00
13	0.00	0.00	0.00	0.00	0.00	0.00	0.00	0.00	0.00	0.00	0.00	0.03	0.00	0.00	0.00	0.00	0.00	0.00	0.00	0.00	0.00	0.00	0.00	0.00
14	0.00	0.00	0.00	0.00	0.00	0.01	0.01	0.01	0.00	0.00	0.00	0.00	0.00	0.00	0.00	0.00	0.00	0.00	0.00	0.00	0.00	0.00	0.00	0.00
15	0.00	0.00	0.00	0.00	0.00	0.00	0.00	0.00	0.00	0.00	0.00	0.07	0.00	0.00	0.00	0.00	0.00	0.00	0.00	0.00	0.00	0.00	0.00	0.00
16	0.00	0.00	0.00	0.00	0.00	0.00	0.00	0.00	0.00	0.00	0.00	0.00	0.00	0.00	0.00	0.00	0.00	0.00	0.00	0.00	0.00	0.00	0.00	0.00
17	0.00	0.00	0.00	0.00	0.00	0.00	0.00	0.00	0.00	0.00	0.00	0.05	0.00	0.00	0.00	0.00	0.01	0.00	0.00	0.00	0.00	0.00	0.00	0.00
18	0.00	0.00	0.00	0.00	0.00	0.00	0.00	0.00	0.00	0.00	0.00	0.22	0.00	0.00	0.00	0.00	0.00	0.00	0.00	0.00	0.00	0.00	0.00	0.00
19	0.00	0.00	0.00	0.00	0.00	0.00	0.00	0.00	0.00	0.00	0.00	0.00	0.00	0.00	0.00	0.00	0.01	0.00	0.00	0.00	0.00	0.00	0.00	0.00
20	0.00	0.00	0.00	0.00	0.00	0.00	0.00	0.00	0.00	0.00	0.00	0.20	0.00	0.00	0.00	0.00	0.00	0.00	0.00	0.00	0.00	0.00	0.00	0.00
21	-0.06	-0.02	-0.11	0.05	0.03	0.63	0.90	0.03	0.02	-0.36	-0.16	0.10	-0.02	0.26	0.94	-0.02	0.00	-0.03	-0.02	-0.05	0.01	-0.06	0.23	-0.06
22	0.02	-0.03	-0.10	0.04	0.16	-0.04	-0.17	0.04	-0.01	0.02	0.04	0.07	-0.03	-0.01	-0.23	-0.10	-0.01	-0.12	-0.08	-0.41	0.02	-0.12	0.22	-0.48
23	0.00	0.00	0.00	0.00	0.00	0.00	0.00	0.00	0.00	0.00	0.00	0.00	0.00	0.00	0.00	0.00	0.00	0.00	0.00	0.00	0.00	0.00	0.00	0.00
24	-0.15	0.39	-0.24	0.30	-0.04	-0.14	-0.34	-0.07	-0.03	0.12	0.27	0.24	-0.14	-0.13	-0.62	-0.01	-0.13	-0.02	-0.02	0.50	0.06	-0.14	0.61	0.17
25	0.03	-0.01	0.29	-0.02	-0.05	-0.13	0.00	0.15	0.23	-0.19	-0.20	0.00	0.97	0.03	-0.15	0.04	0.05	0.03	0.38	0.16	-0.04	0.21	-0.18	0.06
26	0.00	0.00	0.00	0.00	0.00	0.00	0.00	0.00	0.00	0.00	0.00	0.00	0.00	0.00	0.00	0.00	0.00	0.00	0.00	0.00	0.00	0.00	0.00	0.00
27	0.00	0.00	0.00	0.00	0.00	0.00	0.00	0.00	0.00	-0.01	0.00	0.07	0.00	0.00	0.00	0.00	0.00	0.00	0.00	0.00	0.00	0.00	0.00	0.00
28	0.00	0.00	0.00	0.00	0.00	0.00	0.00	0.01	0.00	-0.01	0.00	0.14	0.01	0.00	0.01	0.00	0.00	0.00	0.00	-0.01	0.00	0.00	0.00	-0.02
29	0.00	0.00	0.00	0.00	0.00	0.00	0.00	0.00	0.00	0.00	0.00	0.13	0.00	0.00	0.00	0.00	0.00	0.00	0.00	0.00	0.00	0.00	0.00	0.00

30	0.00	0.00	0.00	0.00	0.00	0.00	0.00	0.00	0.00	0.00	0.00	0.00	0.00	0.00	0.00	0.00	0.00	0.00	0.00	0.00	0.00	0.00	0.00	0.00
31	0.00	0.00	0.00	0.00	0.00	0.00	0.00	0.00	0.00	0.00	0.00	0.25	0.00	0.00	0.00	0.00	0.00	0.00	0.00	0.00	0.00	0.00	0.00	0.00
32	0.00	0.00	0.00	0.00	0.00	0.00	0.00	0.00	0.00	0.00	0.00	0.00	0.00	0.00	0.00	0.00	0.00	0.00	0.00	0.00	0.00	0.00	0.00	0.00
33	0.00	0.00	0.01	0.00	-0.01	0.03	0.03	0.00	0.02	-0.03	-0.02	0.27	0.03	0.02	0.02	0.01	0.00	0.00	0.00	0.03	0.01	-0.01	-0.01	0.01
34	-0.03	0.03	0.00	0.00	0.00	-0.03	-0.02	0.00	0.00	0.02	0.04	0.00	-0.01	-0.04	-0.03	0.00	0.02	0.00	0.04	0.00	-0.05	0.04	0.02	0.00
35	0.02	0.07	0.14	0.04	-0.21	1.00	1.00	-0.08	0.40	-0.80	-0.77	0.00	1.00	0.66	0.73	0.17	-0.23	0.07	-0.08	1.00	0.43	-0.47	-0.11	0.30
36	-0.27	0.31	-0.18	-0.03	0.12	-1.00	-1.00	-0.01	-0.11	0.56	1.00	0.02	-0.21	-1.00	-0.96	-0.07	0.28	-0.05	0.58	-0.39	-1.00	1.00	0.51	-0.41
37	0.13	-0.11	-0.03	0.21	0.05	0.34	0.13	-0.09	0.13	-0.40	-0.31	0.14	0.26	0.32	0.15	-0.05	-0.20	-0.07	-0.07	-0.05	-0.55	-0.25	-0.02	-0.05
38	0.02	-0.05	0.09	0.00	-0.01	0.05	0.02	0.21	0.06	0.10	0.07	0.00	0.21	-0.19	0.04	0.01	-0.01	0.01	-0.01	0.02	-0.03	-0.04	-0.07	0.00
39	0.17	-0.13	0.02	0.24	0.00	0.74	0.48	-0.13	0.32	-0.76	-0.61	0.00	0.68	0.58	0.43	0.00	-0.32	-0.03	-0.10	0.24	-0.49	-0.41	-0.09	0.07
40	1.00	-0.98	-0.33	1.00	0.58	0.98	-0.59	-1.00	0.23	-1.00	-1.00	0.07	0.57	1.00	0.22	-0.54	-1.00	-0.65	-0.44	-1.00	1.00	-1.00	0.05	-1.00
41	0.00	0.00	0.00	0.00	0.00	0.00	0.00	0.00	0.00	0.00	0.00	0.00	0.00	0.00	0.00	0.00	0.00	0.00	0.00	0.00	0.00	0.00	0.00	0.00
42	0.86	-1.00	1.00	0.21	0.19	0.51	-0.28	-0.91	0.07	-0.62	-0.55	0.13	0.56	0.53	0.19	-0.18	-0.64	-0.22	-0.20	-0.50	0.70	-0.60	-1.00	-0.50
43	0.00	0.00	0.00	0.00	0.00	0.00	0.00	0.00	0.00	0.00	0.00	0.51	0.00	0.00	0.00	0.00	0.00	0.00	0.00	0.00	0.00	0.00	0.00	0.00
44	0.01	0.00	0.00	0.00	0.00	-0.02	-0.01	0.00	0.00	0.01	0.02	0.00	0.00	-0.02	-0.02	0.00	-0.13	0.00	0.02	0.01	-0.01	0.03	0.00	0.00
45	-0.36	0.60	-0.01	-0.04	-0.11	-0.32	-0.05	0.21	-0.03	0.19	0.36	0.00	-0.06	-0.37	-0.22	0.11	0.21	0.06	0.58	0.24	-0.09	0.61	0.15	0.07
46	0.00	0.00	0.00	0.00	0.00	0.00	0.00	0.00	0.00	0.00	0.00	0.56	0.00	0.00	0.00	0.00	0.00	0.00	0.00	0.00	0.00	0.00	0.00	0.00
47	-0.27	0.41	0.02	-0.03	-0.11	-0.10	0.08	-0.03	-0.01	0.08	0.16	0.39	-0.02	-0.17	-0.02	0.10	0.13	0.05	0.38	0.30	-0.32	0.34	0.05	0.09
48	-0.06	0.15	-0.01	0.00	-0.01	0.32	0.12	0.04	0.16	-0.28	-0.19	0.51	0.25	0.18	0.15	0.04	-0.05	0.01	-0.02	0.10	0.01	-0.16	0.08	0.02
49	-1.00	1.00	0.27	0.20	0.07	-0.40	-0.26	-0.03	-0.04	0.21	0.36	0.00	-0.07	-0.37	-0.48	-0.09	-0.05	-0.10	0.36	-0.13	-0.02	0.45	-0.05	-0.07
50	-0.01	0.01	0.00	0.00	0.00	0.00	0.00	0.00	0.00	0.00	0.00	0.13	0.00	0.00	-0.01	0.00	0.00	0.00	0.00	0.00	0.00	0.01	0.00	0.00
51	0.00	0.00	0.00	0.00	0.00	0.00	0.00	0.00	0.00	0.00	0.00	0.00	0.00	0.00	0.00	0.00	0.00	0.00	0.00	0.00	0.00	0.00	0.00	0.00
52	0.44	-0.14	-0.18	-0.07	-0.19	0.54	0.32	0.76	0.26	-0.44	-0.25	0.07	0.37	0.25	0.35	0.26	0.22	0.12	0.05	0.64	-0.04	-0.13	0.31	0.15
53	0.00	0.00	0.00	0.00	0.00	0.00	0.00	0.00	0.00	0.00	0.00	0.00	0.00	0.00	0.00	0.00	0.01	0.00	0.00	0.00	0.00	0.00	0.00	0.00
54	0.00	0.00	0.00	0.00	0.00	0.00	0.00	0.00	0.00	0.00	0.00	0.13	0.00	0.00	0.00	0.00	0.00	0.00	0.00	0.00	0.00	0.00	0.00	0.00
55	0.00	0.00	0.00	0.00	0.00	0.01	0.01	0.01	0.00	-0.01	-0.01	0.02	0.01	0.00	0.01	0.00	0.00	0.00	0.00	0.01	0.00	0.00	0.01	0.00
56	-0.47	0.28	0.21	0.35	0.13	-0.18	-0.17	-0.04	-0.02	0.08	0.09	0.37	-0.02	-0.11	-0.26	-0.15	0.04	-0.17	-0.02	-0.23	0.02	0.06	-0.09	-0.27
57	0.27	-0.07	-0.12	-0.06	-0.15	0.22	0.16	0.57	0.12	-0.17	-0.06	0.32	0.11	0.07	0.17	0.19	-0.02	0.09	0.23	0.50	-0.05	0.02	0.22	0.17
58	-0.13	0.14	0.03	0.00	0.00	-0.02	-0.02	0.00	0.00	0.01	0.03	0.00	-0.01	-0.02	-0.04	0.00	0.04	0.00	0.07	0.01	0.00	0.03	0.00	0.00

59	0.02	0.18	-0.05	-0.03	-0.09	0.16	0.09	0.30	0.10	-0.12	-0.03	0.46	0.06	0.05	0.09	0.12	0.07	0.06	0.20	0.30	-0.03	0.02	0.16	0.10
60	0.00	0.01	-0.01	0.00	-0.01	0.03	0.01	0.03	0.01	-0.02	-0.01	0.75	0.01	0.01	0.01	0.01	0.00	0.00	0.00	0.01	0.00	-0.01	0.02	0.00
61	0.00	0.00	0.00	0.00	0.00	0.00	0.00	0.00	0.00	0.00	0.00	0.00	0.00	0.00	0.00	0.00	0.00	0.00	0.00	0.00	0.00	0.00	0.00	0.00
62	0.00	0.00	0.00	0.00	0.00	0.00	0.00	0.00	0.00	0.00	0.00	0.86	0.00	0.00	0.00	0.00	0.00	0.00	0.00	0.00	0.00	0.00	0.00	0.00
63	0.00	0.00	0.00	0.00	0.00	0.00	0.00	0.00	0.00	0.00	0.00	0.14	0.00	0.00	0.00	0.00	0.00	0.00	0.00	0.00	0.00	0.00	0.00	0.00
64	0.00	0.00	0.00	0.00	0.00	0.00	0.00	0.00	0.00	0.00	0.00	0.00	0.00	0.00	0.00	0.00	0.01	0.00	0.00	0.00	0.00	0.00	0.00	0.00
65	0.00	0.00	0.00	0.00	0.00	0.00	0.00	0.00	0.00	0.00	0.00	0.00	0.00	0.00	0.00	0.00	0.00	0.00	0.00	0.00	0.00	0.00	0.00	0.00
66	-0.02	0.04	-0.04	0.04	0.03	0.09	0.06	0.00	0.00	-0.08	-0.13	0.00	0.18	0.10	0.10	-0.03	-0.08	-0.05	-0.08	-0.12	0.02	-0.11	0.09	-0.17
67	-0.01	0.02	0.00	0.00	-0.01	0.03	0.01	0.00	0.02	-0.03	-0.02	0.00	0.04	0.01	0.02	0.00	0.01	0.00	0.00	0.04	0.00	0.03	0.01	0.01
68	0.00	0.00	0.00	0.00	0.00	0.00	0.00	0.00	0.00	0.00	0.00	0.00	0.00	0.00	0.00	0.00	0.00	0.00	0.00	0.00	0.00	0.00	0.00	0.00
69	-0.18	0.26	-0.42	0.32	0.16	-0.82	-0.67	-0.10	-0.07	0.46	0.76	0.00	-0.22	-0.73	-1.00	-0.18	0.14	-0.18	0.61	-0.24	-0.05	0.85	1.00	-0.19
70	0.00	0.00	0.00	0.00	0.00	0.00	0.00	0.00	0.00	0.00	0.00	0.32	0.00	0.00	0.00	0.00	0.00	0.00	0.00	0.00	0.00	0.00	0.00	0.00
71	0.07	0.88	-1.00	-0.02	-0.05	-0.28	-0.08	0.03	-0.03	0.15	0.25	0.00	-0.02	-0.30	-0.19	0.03	0.04	0.02	0.31	0.01	-0.05	0.37	-0.31	0.01
72	-0.05	0.07	-0.11	0.09	0.04	-0.21	-0.15	-0.03	-0.02	0.10	0.18	0.00	-0.03	-0.19	-0.28	-0.04	0.03	-0.04	0.15	-0.04	0.00	0.16	0.27	-0.02
73	0.00	0.00	0.00	0.00	0.00	0.00	0.00	0.00	0.00	0.00	0.00	0.00	0.00	0.00	0.00	0.00	0.00	0.00	0.00	0.00	0.00	0.00	0.00	0.00
74	0.00	0.00	0.00	0.00	0.00	0.00	0.00	0.00	0.00	0.00	0.00	0.00	0.00	0.00	0.00	0.00	0.00	0.00	0.00	0.00	0.00	0.00	0.00	0.00
75	0.00	0.00	0.00	0.00	0.00	0.00	0.00	0.00	0.00	0.00	0.00	0.32	0.00	0.00	0.00	0.00	0.00	0.00	0.00	0.00	0.00	0.00	0.00	0.00
76	0.00	0.00	0.00	0.00	0.00	0.00	0.00	0.00	0.00	0.00	0.00	0.07	0.00	0.00	0.00	0.00	0.01	0.00	0.00	0.00	0.00	0.00	0.00	0.01
77	0.00	0.00	0.00	0.00	0.00	0.00	0.00	0.00	0.00	0.00	0.00	0.02	0.00	0.00	0.00	0.00	0.00	0.00	0.00	0.00	0.00	0.00	0.00	0.00
78	-0.01	0.01	0.00	0.00	0.00	0.01	0.01	0.00	0.01	-0.01	-0.01	0.11	0.03	0.00	0.01	0.00	0.01	0.00	0.00	0.03	0.00	0.01	0.01	0.01
79	0.00	0.00	0.00	0.00	0.00	0.00	0.00	0.00	0.00	0.00	0.00	0.04	0.00	0.00	0.00	0.00	0.00	0.00	0.00	0.00	0.00	0.00	0.00	0.00
80	0.00	0.00	0.00	0.00	0.00	0.00	0.00	0.00	0.00	0.00	0.00	0.00	0.00	0.00	0.00	0.00	0.00	0.00	0.00	0.00	0.00	0.00	0.00	0.00
81	0.02	-0.01	-0.01	0.00	-0.01	-0.05	-0.02	0.01	-0.01	0.02	0.04	0.17	-0.01	-0.04	-0.03	0.00	-0.27	0.00	0.04	0.02	-0.01	0.06	0.00	0.01
82	0.06	-0.04	-0.01	-0.01	-0.02	-0.24	-0.08	0.08	-0.02	0.12	0.20	0.19	-0.04	-0.22	-0.15	0.00	-0.98	0.01	0.19	0.04	-0.03	0.30	-0.01	0.02
83	-0.24	0.33	0.03	0.03	0.00	0.30	0.06	0.00	0.18	-0.28	-0.15	0.33	0.18	0.16	0.07	0.02	1.00	0.01	-0.01	0.03	0.01	-0.13	0.06	0.00
84	0.00	0.00	0.02	0.00	0.00	-0.01	0.00	0.02	0.00	0.00	0.01	0.50	0.00	-0.01	0.00	0.00	0.01	0.00	0.01	0.00	0.00	0.01	-0.02	0.00
85	0.00	0.00	0.00	0.00	0.00	0.00	0.00	0.00	0.00	0.00	0.00	0.24	0.00	0.00	0.00	0.00	0.00	0.00	0.00	0.00	0.00	0.00	0.00	0.00
86	0.02	-0.04	0.24	-0.01	-0.05	-0.06	0.04	0.26	-0.01	0.03	0.06	0.38	0.02	-0.07	0.00	0.04	0.03	0.02	0.14	0.08	-0.03	0.10	-0.16	0.03
87	0.00	0.00	0.00	0.00	0.00	0.00	0.00	0.00	0.00	0.00	0.00	0.04	0.00	0.00	0.00	0.00	0.00	0.00	0.00	0.00	0.00	0.00	0.00	0.00

88	0.00	0.00	0.00	0.00	0.00	0.00	0.00	0.00	0.00	0.00	0.00	0.49	0.00	0.00	0.00	0.00	0.00	0.00	0.00	0.00	0.00	0.00	0.00	0.00
89	0.12	-0.16	0.42	-0.01	-0.03	-0.04	0.00	-0.12	-0.01	0.02	0.02	0.44	0.05	-0.04	0.01	0.02	-0.04	0.01	0.03	-0.01	0.05	0.02	-0.33	-0.01
90	-0.01	0.00	0.00	0.00	0.00	0.00	-0.01	0.01	0.00	0.00	0.00	0.00	0.00	0.00	-0.01	0.00	0.00	0.00	-0.04	0.00	0.00	0.01	0.00	1.00
91	0.00	0.00	0.00	0.00	0.00	0.00	0.00	0.00	0.00	0.00	0.00	0.09	0.00	0.00	0.00	0.00	0.00	0.00	0.00	0.00	0.00	0.00	0.00	0.00
92	0.00	0.00	0.00	0.00	0.00	0.00	0.00	0.00	0.00	0.00	0.00	0.00	0.00	0.00	0.00	0.00	0.00	0.00	0.00	0.00	0.00	0.00	0.00	0.00
93	0.00	0.00	0.00	0.00	0.00	0.00	0.00	0.00	0.00	0.00	0.00	0.00	0.00	0.00	0.00	0.00	0.00	0.00	0.00	0.00	0.00	0.00	0.00	0.00
94	0.00	0.00	0.00	0.00	0.00	0.00	0.00	0.00	0.00	0.00	0.00	0.03	0.00	0.00	0.00	0.00	0.00	0.00	0.00	0.00	0.00	0.00	0.00	0.00
95	0.00	0.00	0.00	0.00	0.00	0.00	0.00	0.00	0.00	0.00	0.00	0.09	0.00	0.00	0.00	0.00	0.00	0.00	0.00	0.00	0.00	0.00	0.00	0.00
96	0.00	0.00	0.00	0.00	0.00	0.00	0.00	0.00	0.00	0.00	0.00	0.00	0.00	0.00	0.00	0.00	0.00	0.00	0.00	0.00	0.00	0.00	0.00	0.00
97	0.00	0.00	0.00	0.00	0.00	0.00	0.00	0.00	0.00	0.00	0.00	0.00	0.00	0.00	0.00	0.00	0.01	0.00	0.00	0.00	0.00	0.00	0.00	0.00
98	0.00	-0.01	0.00	0.00	0.02	0.00	0.00	0.04	0.00	0.00	0.00	0.51	0.01	0.00	0.00	-0.01	0.01	-0.01	-0.01	-0.06	0.00	0.00	0.00	-0.07
99	0.01	-0.05	0.01	-0.01	0.14	0.00	-0.03	0.29	0.03	-0.01	0.00	0.58	0.07	-0.01	-0.06	-0.07	0.05	-0.09	-0.11	-0.42	-0.02	0.02	-0.02	-0.51
100	-0.01	-0.03	0.07	-0.01	0.02	-0.04	0.00	0.17	0.00	0.02	0.03	0.20	0.04	-0.05	-0.05	-0.09	0.05	-0.10	1.00	-0.68	-0.03	0.10	-0.06	-0.73
101	0.02	-0.03	-0.02	0.01	0.08	0.01	-0.05	0.19	0.02	-0.02	-0.02	0.20	0.03	0.01	-0.03	-0.08	0.01	-0.09	0.00	0.03	-0.01	-0.02	0.02	0.01
102	-0.04	0.11	0.04	0.00	-0.07	0.00	0.04	-0.01	0.00	0.01	0.02	0.00	-0.01	-0.02	0.02	0.08	0.00	0.04	0.02	-0.02	0.00	0.03	-0.01	-0.02
103	0.00	0.00	0.01	0.00	0.01	0.00	0.00	0.03	0.00	0.00	0.00	0.59	0.01	-0.01	-0.01	-0.01	0.01	-0.02	0.17	-0.10	-0.01	0.01	-0.01	-0.11
104	0.01	0.29	-0.03	-1.00	0.04	-0.10	-0.08	-0.01	-0.01	0.05	0.08	0.00	-0.02	-0.08	-0.12	-0.05	0.00	-0.05	0.03	-0.05	0.00	0.08	0.13	-0.04
105	0.00	0.07	-0.01	-0.25	0.01	-0.02	-0.02	0.00	0.00	0.01	0.02	0.00	0.00	-0.02	-0.03	-0.01	0.00	-0.01	0.01	-0.01	0.00	0.02	0.03	-0.01
106	-0.84	0.50	0.14	0.23	0.38	-0.50	-0.53	0.35	-0.03	0.20	0.33	0.00	-0.06	-0.36	-0.69	-0.44	0.00	-0.46	0.27	-0.16	-0.06	0.44	-0.03	-0.08
107	0.00	0.00	0.00	0.00	0.00	0.00	0.00	0.00	0.00	0.00	0.00	0.00	0.00	0.00	0.00	0.00	0.01	0.00	0.00	0.00	0.00	0.00	0.00	0.00
108	0.00	0.00	0.00	0.00	0.00	0.00	0.00	0.00	0.00	0.00	0.00	0.00	0.00	0.00	0.00	0.00	0.01	0.00	0.00	0.00	0.00	0.00	0.00	0.00
109	0.00	0.00	0.00	0.00	0.00	0.00	0.00	0.00	0.00	0.00	0.00	0.02	0.00	0.00	0.00	0.00	0.00	0.00	0.00	0.00	0.00	0.00	0.00	0.00
110	-0.06	0.01	-0.02	0.00	-0.01	0.03	0.00	0.08	-1.00	0.53	-0.09	0.00	0.01	0.32	0.03	0.01	0.02	0.01	0.01	0.03	-0.01	0.00	0.05	0.01
111	0.00	-0.01	0.03	0.00	-0.01	-0.01	0.00	0.06	-0.12	-0.70	0.20	0.68	0.12	0.13	0.03	0.01	0.01	0.00	0.03	0.02	-0.01	0.02	-0.02	0.01
112	0.00	0.00	0.01	0.00	0.00	0.00	0.00	0.02	0.01	-0.23	0.06	0.00	0.03	0.02	0.01	0.00	0.01	0.00	0.01	0.00	0.00	0.01	-0.01	0.00
113	0.00	0.00	0.00	0.00	0.00	0.00	0.00	0.00	0.00	-0.03	0.01	0.00	0.00	0.00	0.00	0.00	0.00	0.00	0.00	0.00	0.00	0.00	0.00	0.00
114	0.01	-0.03	0.04	0.00	-0.01	0.02	0.02	0.11	-0.02	-0.01	0.12	0.00	0.15	-0.11	0.02	0.01	0.00	0.01	0.01	0.03	0.00	-0.01	-0.04	0.01
115	0.01	-0.05	0.07	-0.01	-0.02	0.04	0.03	0.20	-0.04	0.01	0.20	0.21	0.28	-0.20	0.04	0.02	0.00	0.01	0.01	0.04	-0.01	-0.01	-0.07	0.01
116	0.00	0.00	0.00	0.00	0.00	0.00	0.00	0.01	0.00	0.00	0.00	0.00	0.00	0.00	0.00	0.00	0.01	0.00	0.00	0.00	0.00	0.00	0.00	0.00

117	-0.03	0.09	-0.04	0.03	0.01	-0.03	-0.07	-0.01	-0.01	-0.27	-0.10	0.40	-0.02	0.19	1.00	-0.01	0.01	-0.01	0.00	-0.01	0.00	0.00	0.11	-0.01
118	0.00	0.00	0.00	0.00	0.00	0.00	0.00	0.01	0.00	0.00	0.00	0.00	0.01	-0.01	0.02	0.00	0.00	0.00	0.00	0.00	0.00	0.00	0.00	0.00
119	0.01	-0.04	0.03	0.00	-0.01	0.02	-0.01	0.14	0.07	0.10	0.08	0.53	0.17	-0.19	0.54	0.01	-0.01	0.01	0.00	0.03	0.00	-0.02	-0.04	0.01
120	0.00	0.00	0.00	0.00	0.00	0.00	0.00	0.00	0.00	0.00	0.00	0.00	0.00	0.00	0.00	0.00	0.00	0.00	0.00	0.00	0.00	0.00	0.00	0.00
121	0.00	0.00	0.00	0.00	0.00	0.00	0.00	0.00	0.00	0.00	0.00	0.01	0.00	0.00	-0.04	0.00	0.01	0.00	0.00	0.00	0.00	0.00	0.00	0.00
122	-0.07	0.39	-0.07	0.07	0.04	-0.08	-0.07	-0.03	-0.03	-0.79	-0.25	0.39	-0.09	0.56	-0.09	-0.03	0.02	-0.04	0.00	-0.05	0.01	0.06	0.26	-0.05
123	0.00	0.00	0.00	0.00	0.00	0.00	0.00	0.00	0.00	0.00	0.00	0.23	0.00	0.00	0.00	0.00	0.00	0.00	0.00	0.00	0.00	0.00	0.00	0.00
124	0.00	0.00	0.00	0.00	0.00	0.00	0.00	0.00	0.00	0.00	0.00	0.19	0.00	0.00	0.00	0.00	0.00	0.00	0.00	0.00	0.00	0.00	0.00	0.00
125	0.00	0.00	0.00	0.00	0.00	0.00	0.00	0.00	0.00	0.00	0.00	0.00	0.00	0.00	0.00	0.00	0.01	0.00	0.00	0.00	0.00	0.00	0.00	0.00
126	0.00	0.00	0.00	0.00	0.00	0.00	0.00	0.00	0.00	0.00	0.00	0.00	0.00	0.00	0.00	0.00	0.01	0.00	0.00	0.00	0.00	0.00	0.00	0.00
127	0.00	0.00	0.00	0.00	0.00	0.01	0.00	0.01	0.00	0.01	0.00	0.00	0.01	-0.01	-0.10	0.00	0.01	0.00	0.00	0.00	0.00	0.00	0.00	0.00
128	0.00	0.00	0.00	0.00	0.00	0.00	0.00	0.00	0.00	0.00	0.00	0.05	0.00	0.00	0.00	0.00	0.00	0.00	0.00	0.00	0.00	0.00	0.00	0.00
129	0.00	0.00	0.00	0.00	0.00	0.00	0.00	0.00	0.00	0.00	0.00	0.00	0.00	0.00	0.00	0.00	0.00	0.00	0.00	0.00	0.00	0.00	0.00	0.00
130	0.00	0.00	0.00	0.00	0.00	0.00	0.00	0.00	0.00	0.00	0.00	0.00	0.00	0.00	0.00	0.00	0.00	0.00	0.00	0.00	0.00	0.00	0.00	0.00
131	-0.03	0.02	-0.07	0.06	0.03	0.09	0.00	-0.01	0.31	-0.99	0.31	0.25	-0.05	0.11	-0.03	-0.02	-0.02	-0.02	-0.02	-0.04	0.01	-0.06	0.17	-0.05
132	0.02	-0.07	0.51	-0.04	-0.12	-0.01	0.10	1.00	-0.05	0.61	0.96	0.00	-1.00	-0.09	0.15	0.12	0.06	0.06	0.30	0.30	-0.09	0.14	-0.34	0.09
133	-0.02	0.18	0.11	-0.01	-0.02	-0.01	0.00	0.10	1.00	0.51	-0.06	-1.00	0.01	0.25	0.02	0.02	0.03	0.01	0.06	0.05	-0.02	0.05	-0.05	0.01
134	-0.01	0.05	0.03	0.00	-0.01	0.00	0.00	0.03	0.27	0.14	-0.02	-0.27	0.00	0.07	0.01	0.01	0.01	0.00	0.02	0.01	0.00	0.01	-0.01	0.00
135	0.00	0.00	0.00	0.00	0.00	0.00	0.00	0.00	0.00	-0.08	0.00	0.11	0.00	0.00	0.00	0.00	0.00	0.00	0.00	0.00	0.00	0.00	0.00	0.00
136	-0.02	0.09	-0.05	0.05	0.02	0.04	-0.01	-0.01	-0.11	1.00	-0.53	0.35	-0.05	-0.24	-0.09	-0.02	-0.01	-0.02	-0.01	-0.03	0.01	-0.03	0.13	-0.03
137	0.10	-0.01	-0.02	0.25	0.92	0.11	0.11	-0.01	0.02	-0.06	-0.03	0.18	0.04	0.04	0.10	0.96	0.04	-1.00	0.00	0.10	0.00	-0.02	0.04	0.02
138	-0.03	0.02	0.00	0.00	0.07	-0.01	-0.02	0.01	0.00	0.01	0.01	0.06	0.00	-0.01	-0.02	0.04	0.01	0.03	0.01	0.00	0.00	0.01	0.00	0.00
139	-0.15	0.01	0.03	-0.03	-0.89	-0.06	-0.12	0.08	0.00	0.01	0.02	0.00	0.00	-0.02	-0.12	-1.00	-0.05	0.42	0.00	-0.05	0.00	0.01	-0.02	-0.03
140	0.08	0.00	-0.02	0.24	1.00	0.09	0.10	-0.01	0.01	-0.05	-0.02	0.12	0.03	0.03	0.08	1.00	0.04	-0.95	0.01	0.09	0.00	-0.01	0.04	0.02
141	-0.15	0.01	0.03	-0.03	-0.88	-0.06	-0.12	0.08	0.00	0.01	0.02	0.27	0.00	-0.02	-0.13	-1.00	-0.05	0.42	0.00	-0.05	0.00	0.00	-0.02	-0.03
142	0.00	0.00	0.00	0.00	0.00	0.00	0.00	0.00	0.00	0.00	0.00	0.00	0.00	0.00	0.00	0.00	0.01	0.00	0.00	0.00	0.00	0.00	0.00	0.00
143	0.00	0.00	0.00	0.00	0.00	0.00	0.00	0.01	0.00	0.00	0.00	0.30	0.00	0.00	0.00	0.00	0.00	1.00	0.00	0.00	0.00	0.00	0.00	0.00
144	0.00	0.00	0.00	0.00	0.00	0.00	0.00	0.00	0.00	0.00	0.00	0.37	0.00	0.00	0.00	0.00	0.00	-0.10	0.00	0.00	0.00	0.00	0.00	0.00
145	0.00	0.00	0.00	0.00	0.00	0.00	0.00	0.00	0.00	0.00	0.00	0.20	0.00	0.00	0.00	0.00	0.00	0.00	0.00	0.00	0.00	0.00	0.00	0.00

146	0.00	0.00	0.00	0.00	0.00	0.00	0.00	0.00	0.00	0.00	0.00	0.17	0.00	0.00	0.00	0.00	0.00	-0.01	0.00	0.00	0.00	0.00	0.00	0.00
147	-0.02	-0.02	-0.02	0.00	0.00	0.30	0.40	0.06	0.04	-0.04	-0.03	0.01	0.04	0.02	-0.09	0.00	0.00	0.00	0.00	0.02	0.00	-0.03	0.04	0.01
148	0.00	0.00	0.00	0.00	0.00	0.00	0.00	0.00	0.00	0.00	0.00	0.00	0.00	0.00	0.00	0.00	0.00	0.00	0.00	0.00	0.00	0.00	0.00	0.00
149	0.00	0.00	0.00	0.00	0.00	0.00	0.00	0.00	0.00	0.00	0.00	0.03	0.00	0.00	0.00	0.00	0.01	0.00	0.00	0.00	0.00	0.00	0.00	0.00
150	0.00	0.00	0.00	0.00	0.00	0.17	-0.35	0.01	0.00	0.00	0.00	0.00	0.00	0.00	0.00	0.00	0.00	0.00	0.00	0.00	0.00	0.00	0.00	0.00
151	0.00	0.00	0.00	0.00	0.00	-0.13	0.27	0.00	0.00	0.00	0.00	0.13	0.00	0.00	0.00	0.00	0.00	0.00	0.00	0.00	0.00	0.00	0.00	0.00
152	0.00	0.00	0.00	0.00	0.00	0.00	0.00	0.00	0.00	0.00	0.00	0.15	0.00	0.00	0.00	0.00	0.00	0.00	0.00	0.00	0.00	0.00	0.00	0.00
153	0.00	0.00	0.00	0.00	0.00	0.00	0.00	0.00	0.00	0.00	0.00	0.00	0.00	0.00	0.00	0.00	0.00	0.00	0.00	0.00	0.00	0.00	0.00	0.00
154	0.00	0.00	0.00	0.00	0.01	-0.03	0.00	0.00	0.00	-0.01	0.00	0.00	0.00	0.00	0.00	0.00	0.01	0.00	0.00	-0.02	0.00	0.00	0.01	-0.02
155	0.00	0.00	0.00	0.00	0.00	0.00	0.00	0.00	0.00	0.00	0.00	0.06	0.00	0.00	0.00	0.00	0.00	0.00	0.00	0.00	0.00	0.00	0.00	0.00
156	-0.02	0.02	0.00	0.00	0.01	0.00	-0.01	0.00	0.00	0.00	0.01	0.00	0.00	-0.01	-0.01	0.03	0.00	0.02	-0.21	0.08	0.00	0.00	0.00	0.01
157	0.00	0.00	0.00	0.00	0.00	0.01	0.00	0.00	0.00	0.00	0.00	0.00	0.00	0.00	0.00	0.00	0.01	0.00	0.09	0.00	0.00	0.00	0.00	0.00
158	-0.06	0.01	0.02	0.00	0.01	-0.04	-0.03	0.06	0.00	0.01	0.02	0.00	0.00	-0.03	-0.05	0.05	0.02	0.03	-1.00	0.06	-0.01	0.04	-0.01	-0.15
159	0.00	0.00	0.00	0.00	0.00	0.00	0.00	0.00	0.00	0.00	0.00	0.00	0.00	0.00	0.00	0.00	0.00	0.00	0.00	0.00	0.00	0.00	0.00	0.00
160	0.00	0.00	0.00	0.00	0.00	0.00	0.00	0.00	0.00	0.00	0.00	0.00	0.00	0.00	0.00	0.00	0.00	0.00	0.00	0.00	0.00	0.00	0.00	0.00
161	0.00	0.00	0.00	0.00	0.00	0.00	0.00	0.00	0.00	0.00	0.00	0.30	0.00	0.00	0.00	0.00	0.00	0.00	0.00	0.00	0.00	0.00	0.00	0.00
162	0.00	0.00	0.00	0.00	0.00	0.00	0.00	0.00	0.00	0.00	0.00	0.00	0.00	0.00	-0.01	0.00	0.00	0.00	0.00	0.00	0.00	0.00	0.00	0.00
163	0.00	0.00	0.00	0.00	0.00	0.00	0.00	0.00	0.00	0.00	0.00	1.00	0.00	0.00	0.00	0.00	0.00	0.00	0.00	0.00	0.00	0.00	0.00	0.00
164	0.00	0.00	0.00	0.00	0.00	0.00	0.00	0.00	0.00	0.00	0.00	0.07	0.00	0.00	0.00	0.00	0.01	0.00	0.00	0.00	0.00	0.00	0.00	0.00
165	0.00	0.00	0.00	0.00	0.00	0.00	0.00	0.00	0.00	0.00	0.00	0.41	0.00	0.00	0.00	0.00	0.00	0.00	0.00	0.00	0.00	0.00	0.00	0.00
166	0.00	0.00	0.00	0.00	0.00	0.00	0.00	0.00	0.00	0.00	0.00	0.04	0.00	0.00	0.00	0.00	0.00	0.00	0.00	0.00	0.00	0.00	0.00	0.00
167	0.00	0.00	0.00	0.00	0.00	0.00	0.00	0.00	0.00	0.00	0.00	0.44	0.00	0.00	0.00	0.00	0.00	0.00	0.00	0.00	0.00	0.00	0.00	0.00
168	0.00	0.00	0.00	0.00	0.00	0.00	0.00	0.00	0.00	0.00	0.00	0.02	0.00	0.00	0.00	0.00	0.00	0.00	0.00	0.00	0.00	0.00	0.00	0.00
169	0.00	0.00	0.00	0.00	0.00	0.00	0.00	0.00	0.00	0.00	0.00	0.19	0.00	0.00	0.00	0.00	0.00	0.00	0.00	0.00	0.00	0.00	0.00	0.00
170	0.00	0.00	0.00	0.00	0.00	0.00	0.00	0.00	0.00	0.00	0.00	0.00	0.00	0.00	0.00	0.00	0.00	0.00	0.00	0.00	0.00	0.00	0.00	0.00
171	0.00	0.00	0.00	0.00	0.00	0.00	0.00	0.00	0.00	0.00	0.00	0.00	0.00	0.00	0.00	0.00	0.01	0.00	0.00	0.00	0.00	0.00	0.00	0.00
172	0.00	0.00	0.00	0.00	0.00	0.00	0.00	0.00	0.00	0.00	0.00	0.10	0.00	0.00	0.00	0.00	0.00	0.00	0.00	0.00	0.00	0.00	0.00	0.00
173	0.00	0.00	0.00	0.00	0.00	0.00	0.00	0.00	0.00	0.00	0.00	0.22	0.00	0.00	0.00	0.00	0.01	0.00	0.00	0.00	0.00	0.00	0.00	0.00
174	0.00	0.00	0.00	0.00	0.00	0.00	0.00	0.00	0.00	0.00	0.00	0.23	0.00	0.00	0.00	0.00	0.00	0.00	0.00	0.00	0.00	0.00	0.00	0.00

175	0.00	0.00	0.00	0.00	0.00	0.00	0.00	0.00	0.00	0.00	0.00	0.21	0.00	0.00	0.00	0.00	0.00	0.00	0.00	0.00	0.00	0.00	0.00	0.00
176	0.00	0.00	0.00	0.00	0.00	0.00	0.00	0.00	0.00	0.00	0.00	0.00	0.00	0.00	0.00	0.00	0.01	0.00	0.00	0.00	0.00	0.00	0.00	0.00
177	0.00	0.00	0.00	0.00	0.00	0.00	0.00	0.00	0.00	0.00	0.00	0.03	0.00	0.00	0.00	0.00	0.00	0.00	0.00	0.00	0.00	0.00	0.00	0.00
178	0.00	0.00	0.00	0.00	0.00	0.00	0.00	0.00	0.00	0.00	0.00	0.00	0.00	0.00	0.00	0.00	0.00	0.00	0.00	0.00	0.00	0.00	0.00	0.00
179	0.00	0.00	0.00	0.00	0.00	0.00	0.00	0.00	0.00	0.00	0.00	0.23	0.00	0.00	0.00	0.00	0.00	0.00	0.00	0.00	0.00	0.00	0.00	0.00
180	0.00	0.00	0.00	0.00	0.00	0.00	0.00	0.00	0.00	0.00	0.00	0.00	0.00	0.00	0.00	0.00	0.00	0.00	0.00	0.00	0.00	0.00	0.00	0.00
181	0.00	0.00	0.00	0.00	0.00	0.00	0.00	0.00	0.00	0.00	0.00	0.00	0.00	0.00	0.00	0.00	0.00	0.00	0.00	0.00	0.00	0.00	0.00	0.00
182	0.00	0.00	0.00	0.00	0.00	0.00	0.00	0.00	0.00	0.00	0.00	0.01	0.00	0.00	0.00	0.00	0.00	0.00	0.00	0.00	0.00	0.00	0.00	0.00
183	0.00	0.00	0.00	0.00	0.00	0.00	0.00	0.00	0.00	0.00	0.00	0.29	0.00	0.00	0.00	0.00	0.00	0.00	0.00	0.00	0.00	0.00	0.00	0.00
184	0.00	0.00	0.00	0.00	0.00	0.00	0.00	0.00	0.00	0.00	0.00	0.00	0.00	0.00	0.02	0.00	0.00	0.00	0.00	0.00	0.00	0.00	0.00	0.00
185	0.00	0.00	0.00	0.00	0.00	0.00	0.00	0.00	0.00	0.00	0.00	0.07	0.00	0.00	0.00	0.00	0.00	0.00	0.00	0.00	0.00	0.00	0.00	0.00
186	0.00	0.00	0.00	0.00	0.00	0.00	0.00	0.00	0.00	0.00	0.00	0.00	0.00	0.00	0.00	0.00	0.00	0.00	0.00	0.00	0.00	0.00	0.00	0.00
187	0.00	0.00	0.00	0.00	0.00	0.00	0.00	0.00	0.00	0.00	0.00	0.00	0.00	0.00	0.00	0.00	0.00	0.00	0.00	0.00	0.00	0.00	0.00	0.00
188	0.00	0.00	0.00	0.00	0.00	0.00	0.00	0.00	0.00	0.00	0.00	0.00	0.00	0.00	0.00	0.00	0.00	0.00	0.00	0.00	0.00	0.00	0.00	0.00
189	0.00	0.00	0.00	0.00	0.00	0.00	0.00	0.00	0.00	0.00	0.00	0.15	0.00	0.00	0.00	0.00	0.01	0.00	0.00	0.00	0.00	0.00	0.00	0.00
190	0.00	0.00	0.00	0.00	0.00	0.00	0.00	0.00	0.00	0.00	0.00	0.00	0.00	0.00	0.00	0.00	0.00	0.00	0.00	0.00	0.00	0.00	0.00	0.00
191	0.00	0.00	0.00	0.00	0.00	0.00	0.00	0.00	0.00	0.00	0.00	0.04	0.00	0.00	0.00	0.00	0.00	0.00	0.00	0.00	0.00	0.00	0.00	0.00
192	0.00	0.00	0.00	0.00	0.00	0.00	0.00	0.00	0.00	0.00	0.00	0.00	0.00	0.00	0.00	0.00	0.01	0.00	0.00	0.00	0.00	0.00	0.00	0.00
193	0.00	0.00	0.00	0.00	0.00	0.00	0.00	0.00	0.00	0.00	0.00	0.00	0.00	0.00	0.00	0.00	0.00	0.00	0.00	0.00	0.00	0.00	0.00	0.00
194	0.00	0.00	0.00	0.00	0.00	0.00	0.00	0.00	0.00	0.00	0.00	0.00	0.00	0.00	0.00	0.00	0.00	0.00	0.00	0.00	0.00	0.00	0.00	0.00
195	0.00	0.00	0.00	0.00	0.00	0.00	0.00	0.00	0.00	0.00	0.00	0.00	0.00	0.00	0.00	0.00	0.00	0.00	0.00	0.00	0.00	0.00	0.00	0.00
196	0.00	0.00	0.00	0.00	0.00	0.00	0.00	0.00	0.00	0.00	0.00	0.00	0.00	0.00	0.00	0.00	0.00	0.00	0.00	0.00	0.00	0.00	0.00	0.00
197	0.00	0.00	0.00	0.00	0.00	0.00	0.00	0.00	0.00	0.00	0.00	0.00	0.00	0.00	0.00	0.00	0.00	0.00	0.00	0.00	0.00	0.00	0.00	0.00
198	0.00	0.00	0.00	0.00	0.00	0.00	0.00	0.00	0.00	0.00	0.00	0.00	0.00	0.00	0.00	0.00	0.00	0.00	0.00	0.00	0.00	0.00	0.00	0.00
199	0.00	0.00	0.00	0.00	0.00	0.00	0.00	0.00	0.00	0.00	0.00	0.00	0.00	0.00	0.00	0.00	0.00	0.00	0.00	0.00	0.00	0.00	0.00	0.00
200	0.00	0.00	0.00	0.00	0.00	0.00	0.00	0.00	0.00	0.00	0.00	0.00	0.00	0.00	0.00	0.00	0.00	0.00	0.00	0.00	0.00	0.00	0.00	0.00
201	0.00	0.00	0.00	0.00	0.00	0.00	0.00	0.00	0.00	0.00	0.00	0.00	0.00	0.00	0.00	0.00	0.00	0.00	0.00	0.00	0.00	0.00	0.00	0.00
202	0.00	0.00	0.00	0.00	0.00	0.00	0.00	0.00	0.00	0.00	0.00	0.00	0.00	0.00	0.00	0.00	0.00	0.00	0.00	0.00	0.00	0.00	0.00	0.00
203	0.00	0.00	0.00	0.00	0.00	0.00	0.00	0.00	0.00	0.00	0.00	0.00	0.00	0.00	0.00	0.00	0.00	0.00	0.00	0.00	0.00	0.00	0.00	0.00

204	0.00	0.00	0.00	0.00	0.00	0.00	0.00	0.00	0.00	0.00	0.00	0.00	0.00	0.00	0.00	0.00	0.00	0.00	0.00	0.00	0.00	0.00	0.00	0.00
205	0.00	0.00	0.00	0.00	0.00	0.00	0.00	0.00	0.00	0.00	0.00	0.00	0.00	0.00	0.00	0.00	0.00	0.00	0.00	0.00	0.00	0.00	0.00	0.00
206	0.00	0.00	0.00	0.00	0.00	0.00	0.00	0.00	0.00	0.00	0.00	0.00	0.00	0.00	0.00	0.00	0.00	0.00	0.00	0.00	0.00	0.00	0.00	0.00
207	0.00	0.00	0.00	0.00	0.00	0.00	0.00	0.00	0.00	0.00	0.00	0.07	0.00	0.00	0.00	0.00	0.00	0.00	0.00	0.00	0.00	0.00	0.00	0.00
208	0.00	0.00	0.00	0.00	0.00	0.00	0.00	0.00	0.00	0.00	0.00	0.46	0.00	0.00	0.00	0.00	0.00	0.00	0.00	0.00	0.00	0.00	0.00	0.00
209	0.00	0.00	0.00	0.00	0.00	0.00	0.00	0.00	0.00	0.00	0.00	0.00	0.00	0.00	0.00	0.00	0.00	0.00	0.00	0.00	0.00	0.00	0.00	0.00
