# Supplementary material for: HEPATOKIN1 is a biochemistry-based model of liver metabolism for applications in medicine and pharmacology
Source: Nat Commun. 2018 Jun 19;9:2386. doi: 10.1038/s41467-018-04720-9 (PMC6008457; doi:10.1038/s41467-018-04720-9)
Supplement: Supplementary file 6 — Supplementary Data 3 [file 41467_2018_4720_MOESM6_ESM.pdf]

Supplementary Information to

A Biochemistry-Based Model of Liver Metabolism for Applications in Medicine and Pharmacology

N. Berndt et al.

Supplementary Data 3 Fold-change of enzyme abundances in tumor cells versus normal hepatocytes based on proteomics data

#	Enzyme short name	HCC 1 fold change	HCC2 fold change	Adenoma fold change
	Fatty acid uptake			
1	$v_{c16_{cyt}-uptake}^{carrier}$	0.13	0.66	0.31
2	$v_{c16_{cyt}-uptake}^{diffusion}$	1	1	1
3	v_{ACSL1}	0.03	0.25	0.78
4	v_{ACSL4}	0.43	1	1
5	v_{ACSL5}	1	1.50	0.43
	Beta-oxidation			
6	v_{CPT1}	0.19	1	0.75
7	v_{CACT}	0.49	0.37	0.92
8	v_{CPT2}	0.29	0.60	0.83
9	$v_{c4coa-scdh}$	0.12	0.12	0.17
	$v_{c5coa-scdh}$	0.12	0.12	0.17
10	$v_{c6coa-mcdh}$	0.14	0.65	0.31
	$v_{c8coa-mcdh}$	0.14	0.65	0.31
	$v_{c10coa-mcdh}$	0.14	0.65	0.31
	$v_{c12coa-mcdh}$	0.14	0.65	0.31
11	$v_{c10coa-lcdh}$	0.58	1	0.98
	$v_{c12coa-lcdh}$	0.58	1	0.98
	$v_{c14coa-lcdh}$	0.58	1	0.98
	$v_{c16coa-lcdh}$	0.58	1	0.98
12	$v_{ehyd-ec4}$	0.11	0.81	1.02
	$v_{ehyd-ec5}$	0.11	0.81	1.02
	$v_{ehyd-ec6}$	0.11	0.81	1.02
	$v_{ehyd-ec8}$	0.11	0.81	1.02

	$v_{ehyd-ec10}$	0.11	0.81	1.02
	$v_{ehyd-ec12}$	0.11	0.81	1.02
	$v_{ehyd-ec14}$	0.11	0.81	1.02
	$v_{ehyd-ec16}$	0.11	0.81	1.02
13	$v_{3hdh-lc4}$	0.18	0.71	1.10
	$v_{3hdh-lc5}$	0.18	0.71	1.10
	$v_{3hdh-lc6}$	0.18	0.71	1.10
	$v_{3hdh-lc8}$	0.18	0.71	1.10
	$v_{3hdh-lc10}$	0.18	0.71	1.10
	$v_{3hdh-lc12}$	0.18	0.71	1.10
	$v_{3hdh-lc14}$	0.18	0.71	1.10
	$v_{3hdh-lc16}$	0.18	0.71	1.10
14	v_{3kt}^{kc4coa}	0.06	0.62	0.66
15	v_{3kt}^{kc5coa}	0.21	0.34	0.72
	v_{3kt}^{kc6coa}	0.21	0.34	0.72
	v_{3kt}^{kc8coa}	0.21	0.34	0.72
	$v_{3kt}^{kc10coa}$	0.21	0.34	0.72
	$v_{3kt}^{kc12coa}$	0.21	0.34	0.72
	$v_{3kt}^{kc14coa}$	0.21	0.34	0.72
	$v_{3kt}^{kc16coa}$	0.21	0.34	0.72
16	v_{pcc}	0.04	0.24	1.32
17	v_{mmrm}	1	0.13	0.79
18	v_{mmm}	0.10	0.38	0.48
19	$v_{ETF-FAD}$	1	1	1
20	v_{ETF-QO}	1	1	1

	Citric acid cycle			
21	v_{pdhc}	1.41	1.74	3.56
22	v_{cs}	2.21	2.28	3.46
23	v_{ac}	0.38	0.34	1.10
24	v_{idh}	0.95	1.09	2.14
25	v_{kgdhc}	0.27	1.15	1.39
26	$v_{scs-atp}$	0.68	0.73	1.58
27	$v_{scs-gtp}$	0.44	0.52	1.71
28	v_{succdh}	0.44	0.76	0.90
29	v_{fum}	0.48	0.40	1.20
30	$v_{mdh_{mito}}$	0.38	0.73	1.80
31	v_{tdh}	0.29	0.64	0.84
	Mitochondrial electrophysiology and ATP synthesis			
32	$I_{cl_{ed}}$	1	1	1
33	I_{na}^{pump}	1	1	1
34	$I_{na_{ed}}$	1	1	1
35	I_{k}^{pump}	1	1	1
36	$I_{k_{ed}}$	1	1	1
37	v_{F0F1}	0.70	0.53	1.56
38	v_{nex}	1.48	0.85	1.03
39	v_{P-ex}	3.56	0.85	1.13
40	v_{cxI}	1.09	0.61	1.00
41	v_{cxIII}	1.16	0.85	1.29
42	v_{cxIV}	0.63	0.45	1.73

43	$v_{ak_{cyt}}$	0.35	1.36	2.66
44	v_{ppase}	0.76	0.55	1.19
45	$v_{atp-usage}$	1	1	1
46	$v_{O_{2diff}}$	1	1	1
	I_H^{pump}	1	1	1
47	$I_{H_{ed}}$	1	1	1
	Glycolysis			
48	v_{gluT2}	1	0.61	0.22
49	v_{Gk}	1	1	1
50	v_{hk}	1	1	1
51	$v_{glc6pT_{er}}$	1	1	1
52	$v_{glc6pp_{er}}$	1	1	1
53	$v_{glcT_{er}}$	1	1	1
54	$v_{pT_{er}}$	1	1	1
55	v_{gpi}	0.79	0.36	2.96
56	v_{pfk2}	1	1	1
57	v_{fbp2}	1	1	1
58	v_{pfk1}	1.10	1.81	1.07
59	v_{fbp1}	0.04	0.09	0.56
60	v_{ald}	0.0006	0.20	0.73
61	v_{tpi}	1.72	0.59	1.03
62	v_{gapdh}	1.09	0.73	0.97
63	v_{pgk}	0.96	0.97	1.38
64	v_{pgm}	1.46	0.61	0.97
65	v_{eno}	0.83	0.47	0.71

66	v_{pk}	1	1.10	2.19
67	v_{pepck}	1	0.06	1
68	$v_{pepck_{mito}}$	0.01	1.16	0.58
69	v_{pc}	0.01	1.05	0.59
70	v_{ldh}	11.86	1.45	1.15
71	v_{lacT}	1	1	1.19
	v_{pyrT}	1	1	1.19
72	$v_{pyrT_{mito}}$	1	1	1.19
73	v_{malT}	1	1	1
74	$v_{mal-pyrT}$	1	1	1
75	v_{mdh}	0.67	0.38	0.49
76	v_{me}	1	1	1
77	v_{pepT}	1	1	1
78	$v_{ndk_{cyt}}$	1.30	0.80	2.19
	$v_{ndk_{cyt}}^{udp}$	1.30	0.80	2.19
79	$v_{ndk_{mito}}$	1	1	1
	Glycogen metabolism			
80	v_{gpm}	1.79	0.50	1.85
81	v_{upgase}	0.12	0.16	0.41
82	v_{gs}	0.41	0.54	0.71
83	v_{gp}	0.48	0.31	2.75
	Mal-Asp shuttle			
84	$v_{asat_{mito}}$	0.12	0.17	0.77
85	v_{asat}	0.12	0.17	0.81

86	v_{agc}	0.05	1.32	0.94
87	v_{mac}	0.82	1.17	2.63
88	v_{g3pdh}	1	0.55	1.33
89	$v_{g3pdh_{mito}}$	8.22	1.97	1
	PPP			
90	v_{g6pdh}	0.43	1.33	0.61
91	v_{pgls}	0.66	0.52	1.46
92	v_{pgdh}	1.41	0.92	2.64
93	v_{rpe}	1.42	0.81	1
94	v_{rpi}	2.10	1	1
95	v_{taldo}	0.80	0.48	0.71
96	v_{tketo1}	2.29	1.46	2.60
97	v_{tketo2}	2.29	1.46	2.60
	Fatty acid synthesis			
98	$v_{cit-mal}$	1	0.41	2.16
99	$v_{cit-lys}$	4.62	1.21	13.26
100	v_{acc1}	0.54	1.86	3.27
101	v_{acc2}	0.22	1	1
102	v_{mdc2}	1	1	0.73
103	v_{fas-c4}	0.21	1.34	6.29
	v_{fas-c6}	0.21	1.34	6.29
	v_{fas-c8}	0.21	1.34	6.29
	$v_{fas-c10}$	0.21	1.34	6.29
	$v_{fas-c12}$	0.21	1.34	6.29
	$v_{fas-c14}$	0.21	1.34	6.29

	$v_{fas-c16}$	0.21	1.34	6.29
	TAG synthesis			
104	v_{glycT}	1	1	1
105	v_{glycK}	0.21	0.33	0.59
106	v_{gpat}	1	1	1
107	v_{agpat}	2.23	0.44	1
108	v_{pap}	1	1	1
109	v_{dgat}	1	1	1
	Urea synthesis			
110	$v_{nh_3-uptake}$	1	1	1
111	v_{nh_3-diff}	1	1	1
112	$v_{glnT_{mito}}$	1	1	1
113	$v_{glnase-b}$	1	1	1
	$v_{glnase-free}$	1	1	1
114	v_{gdh}	0.35	0.48	1.37
115	$v_{gdh-nadp}$	0.35	0.48	1.37
116	v_{argT}	1	1	1
117	$v_{aglu-syn}$	1	1	1
118	v_{acgluT}	1	1	1
119	$v_{acglu-hyd}$	0.06	0.10	1.28
120	$v_{gluT_{mito}}$	1	1	1
121	$v_{acoa-syn}$	0.16	1.31	6.59
122	v_{cps}	0.0006	0.88	0.42
123	v_{otc}	1	1	0.73
124	v_{ocT}	0.63	0.32	0.89

125	v_{ass}	0.02	0.22	0.50
126	v_{asl}	1	0.30	0.72
127	v_{argase}	1	0.12	1.42
128	v_{ureaT}	1	1	1
129	v_{fmT}	1	1	1
130	v_{aat}	0.51	0.65	0.40
131	v_{gluT}	0.57	1	0.58
132	v_{alnT}	1	1	1
133	v_{serT}	1	1	1
134	v_{sdh}	1	1	1
135	v_{glnT}	1	1	1
136	$v_{gln-syn}$	1	1	1
	VLDL-LD synthesis			
137	$v_{LD-syn-tag}$	1	1	1
138	$v_{LD-syn-ce}$	1	1	1
139	$v_{apoB-syn}$	1	1	1
140	$v_{apoB-deg}$	1	1	1
141	v_{MTP}^{tag}	1	0.67	0.45
	v_{MTP}^{ce}	1	0.67	0.45
142	$v_{VLDL-ex-tag}$	1	1	1
	$v_{VLDL-ex-ce}$	1	1	1
143	v_{ATGL}^{tag}	1	1	1
144	v_{HSL}^{dag}	1	1	1
145	v_{magl}	0.83	1	1.19
146	v_{cee}	0.81	1	1.66

	Ketone body synthesis			
147	$v_{hmg-syn}$	1	0.24	0.49
148	$v_{hmg-lys}$	0.03	0.62	1.35
149	$v_{\beta hdh}$	0.01	0.54	0.76
150	v_{acacT}	1	1	1.19
151	$v_{\beta hbT}$	1	1	1.19
152	$v_{acac-ex}$	1	1	1.19
153	$v_{\beta hb-ex}$	1	1	1.19
	Cholesterol synthesis			
154	$v_{acaccoa-syn}$	0.21	0.76	6.69
155	v_{3kt}^{kc4coa}	0.10	0.20	2.63
156	$v_{hmg-syn-cyt}$	2.52	11.78	58.20
157	v_{hmgl}	0.03	0.62	1.35
158	v_{hmgr}	1	1	1
159	v_{mevk}	1	0.50	1.08
160	v_{pmevk}	2.83	2.15	0.91
161	v_{mdp}	0.94	1.03	5.46
162	v_{ippi}	0.18	0.83	4.83
163	v_{gpps}	1	1	1
164	v_{fpps}	2.52	0.75	5.36
165	$v_{sqs-nadph}$	1	1	1
	$v_{sqs-nadh}$	1	1	1
166	v_{sqe}	1	1	1
167	v_{osc}	1	0.65	2.61

168	$v_{s14dm-lan}$	0.69	1	7.64
	$v_{s14dm-dihlan}$	0.69	1	7.64
169	$v_{s14r-ffmas}$	1	1	1
	$v_{s14r-dihffmas}$	1	1	1
170	$v_{mso-tmas}$	1	1	1
	$v_{mso-dmtmas}$	1	1	1
	$v_{mso-dihtmas}$	1	1	1
	$v_{mso-dmdihtmas}$	1	1	1
171	$v_{casdc-dmtmasca}$	0.03	0.24	5.55
	$v_{casdc-ddimtmasca}$	0.03	0.24	5.55
	$v_{casdc-dmdihtmasca}$	0.03	0.24	5.55
	$v_{casdc-ddimdihtmasca}$	0.03	0.24	5.55
172	$v_{3ksr-dmktmas}$	0.85	1	1
	$v_{3ksr-ddimktmas}$	0.85	1	1
	$v_{3ksr-ddimkdihtmas}$	0.85	1	1
	$v_{3ksr-dmkdihtmas}$	0.85	1	1
173	$v_{s78i-zym}$	1	1	1
	$v_{s78i-zymostenol}$	1	1	1
174	$v_{lathox-lath}$	1	1	1
	$v_{lathox-dhlath}$	1	1	1
175	$v_{s7r-dhchol}$	1	1	1
	$v_{s7r-dhdesm}$	1	1	1
176	$v_{s24r-lan}$	1	1	1.82
	$v_{s24r-ffmas}$	1	1	1.82
	$v_{s24r-tmas}$	1	1	1.82

	$v_{s24r-zym}$	1	1	1.82
	$v_{s24r-dhlath}$	1	1	1.82
	$v_{s24r-dhdesmo}$	1	1	1.82
	$v_{s24r-desmo}$	1	1	1.82
177	v_{acat}	1	1.37	1
	Alcohol metabolism			
178	v_{ethT}	1	1	1
179	v_{adh}	0.17	0.46	0.76
180	v_{alddhI}	1	1	1
181	$v_{alddhII}$	1	1	1
182	v_{aaldT}	1	1	1
183	$v_{alddh_{mito}}$	0.13	0.95	1.44
184	v_{aceT}	1	1	1.19
185	$v_{aceT_{mito}}$	1	1	1.19
	Galactose metabolism			
186	v_{galT}	1	0.61	0.22
187	v_{aldor}	1	1	1
188	v_{galolT}	1	1	1
189	v_{galdh}	1	1	1
190	v_{galaT}	1	1	1
191	v_{guldh}	0.12	0.42	0.57
192	v_{galdc}	0.12	0.42	0.57
193	v_{xylk}	1	1	1
194	v_{galk}	0.53	2.30	0.89
195	v_{galt}	0.06	0.22	0.73

196	v_{gale}	1.18	1.84	23.65
197	v_{gal1pp}	0.37	1.25	2.26
	Fructose metabolism			
198	v_{fruT}	1	0.61	0.22
199	v_{fruk}	1	0.14	0.74
200	v_{aldB}	0.0006	0.20	0.73
201	v_{triok}	1	0.68	0.88
202	v_{aldr}	2.21	2.41	46.46
203	$v_{alddh-gra}$	0.13	0.95	1.44
204	v_{sordh}	0.02	0.78	1.20
205	v_{gck}	1	1	1
206	v_{graT}	1	1	1
	Valproate metabolism			
207	$v_{val-diff}$	1	1	1
208	v_{vcs}	1	1	1
6	$v_{CPT1-val}$	0.19	1	0.75
7	$v_{CACT-val}$	0.49	0.37	0.92
8	$v_{CPT2-val}$	0.29	0.60	0.83
209	$v_{valcoa-bcdh}$	0.03	0.85	0.35
12	$v_{ehyd-evalcoa}$	0.11	0.81	1.02
13	$v_{3hdh-lvalcoa}$	0.18	0.71	1.10
14	$v_{3kt}^{kvalcoa}$	0.21	0.34	0.72
